# HLA allele and haplotype diversity in Central Anatolia: a comparative analysis of donors and transplant candidates across six HLA loci (HLA-A, HLA-B, HLA-C, HLA-DRB1, HLA-DQB1, and HLA-DPB1)

**DOI:** 10.55730/1300-0144.6216

**Published:** 2026-02-16

**Authors:** Emel YANTIR, Eren GÜNDÜZ

**Affiliations:** 1 Department of Immunology, Faculty of Medicine, Eskişehir Osmangazi University, Eskişehir, Turkiye; 2 Division of Hematology, Department of Internal Medicine, Faculty of Medicine, Eskişehir Osmangazi University, Eskişehir, Turkiye

**Keywords:** Human leukocyte antigen, six loci, allele frequency, haplotype frequency, Türkiye, transplantation

## Abstract

**Background/aim:**

Human leukocyte antigen (HLA) polymorphisms play a central role in immune recognition, disease susceptibility, and transplant success. The distribution of HLA alleles and haplotypes varies among ethnic and geographic populations. Despite several regional studies conducted in Türkiye, large-scale and comprehensive investigations comparing HLA profiles between healthy individuals and transplant candidates remain limited. The aim of this study was to compare the distribution of HLA profiles among donors, hematopoietic stem cell transplantation (HSCT) candidates, and solid organ transplant candidates.

**Materials and methods:**

A total of 8472 individuals were included in the study, comprising 6422 donors and 2050 transplant candidates. HLA genotyping was performed using polymerase chain reaction with sequence-specific primers and polymerase chain reaction with sequence-specific oligonucleotide probes. Allele, genotype, and haplotype frequencies were calculated using the PyPop (version 1.2.0) and GENE[RATE] software tools.

**Results:**

The most frequent alleles at the HLA-A, HLA-B, HLA-C, HLA-DRB1, HLA-DQB1, and HLA-DPB1 loci were similar across all groups, with A*02, B*35, C*07, DRB1*11, DQB1*03, and DPB1*04 observed as the predominant alleles. Several alleles were significantly overrepresented in solid organ transplant candidates compared with donors, including A*31, A*33, B*15, B*42, B*51, B*54, DRB1*04, and DRB1*12, whereas DRB1*11 was significantly underrepresented (p < 0.05). A*23 was also observed at a significantly higher frequency in kidney transplant candidates. No significant allele differences were observed between HSCT candidates and donors. Haplotype analysis revealed that the most prevalent six-locus haplotype (A:B:C:DRB1:DQB1:DPB1) was 02~35~04~11~03~04 in donors, whereas 24~35~04~11~03~04 was observed in HSCT candidates and 24~35~04~04~03~04 in solid organ transplant candidates.

**Conclusion:**

To our knowledge, this study represents the largest and most comprehensive analysis of HLA distribution in Türkiye to date and the first to report six-locus HLA haplotypes, including the HLA-DPB1 locus, in transplant candidates. The results indicate that solid organ transplant candidates exhibit significant differences in HLA allele and haplotype frequencies compared with healthy donors. These findings underscore the importance of region-specific HLA data in optimizing donor selection and potentially improving transplant outcomes.

## 1. Introduction

The human leukocyte antigen (HLA) system is a highly polymorphic gene region encoded on the short arm of chromosome 6 in the human genome. As of March 2025, a total of 41,003 alleles are listed in the IMGT/HLA database (version 3.59) [1]. Tissue compatibility is directly associated with graft rejection and posttransplant survival. Traditional evaluations of the HLA-A, HLA-B, and HLA-DRB1 loci are recommended for solid organ transplantation, whereas assessment of six loci is recommended for hematopoietic stem cell transplantation (HSCT) [2].

HLA gene frequencies vary according to ethnic background and geographic distribution. An increasing number of studies worldwide report HLA distribution from different nations and populations. Although studies have reported HLA distributions across different regions of Türkiye, investigations involving large population cohorts remain limited [3–6].

This study was designed as a follow-up to our previously published research on HLA distribution in donors [7]. HLA data from donors were compared with those from HSCT and solid organ transplant candidates. Although the study population was limited to the Central Anatolian region, to our knowledge, this investigation represents the largest and most detailed HLA population genetics analysis conducted in Türkiye to date.

## 2. Materials and methods

### 2.1. Populations

Data from 8472 individuals who underwent HLA typing at the Eskişehir Osmangazi University Hospital Tissue Typing Laboratory between 2001 and 2023 were analyzed. The study population comprised 6422 healthy donors and 2050 transplant candidates, including 1082 HSCT candidates and 968 solid organ transplant candidates. Solid organ transplant candidates consisted of kidney (n = 912), liver (n = 12), and corneal (n = 44) transplant recipients. HSCT was planned for patients diagnosed with leukemia or myelodysplastic syndrome (MDS) (n = 777), lymphoma (n = 117), and other hematologic disorders (n = 188). A total of six HLA loci (HLA-A, HLA-B, HLA-C, HLA-DRB1, HLA-DQB1, and HLA-DPB1) were evaluated using DNA-based molecular typing methods, including sequence-specific oligonucleotide probes (PCR-SSO) and sequence-specific primers (PCR-SSP). Individuals were required to have data for at least three loci (HLA-A, HLA-B, and HLA-DRB1) to be included in the analysis. The proportions of female and male participants were 46.7% and 53.3% among donors, 43.5% and 56.5% among HSCT candidates, and 44.8% and 55.2% among solid organ transplant candidates, respectively. No statistically significant difference in sex distribution was observed among the groups (p > 0.05).

The study was conducted in accordance with the principles of the Declaration of Helsinki and was approved by the Eskişehir Osmangazi University Faculty of Medicine Ethics Committee (approval no: 2026–55).

### 2.2. HLA typing methods

HLA typing was performed using the PCR-SSO and/or PCR-SSP methods as previously described for peripheral blood samples [7]. All HLA data generated prior to 2010 were reannotated according to the current IMGT/HLA nomenclature. All HLA data were validated prior to analysis using the quality control tools implemented in the GENE[RATE] software [8,9].

### 2.3. Statistical analysis

All statistical analyses were conducted using the methods described in our previous study to ensure methodological consistency [7]. The HLA data were used to estimate genotype and allele proportions, haplotype frequencies, the number of common heterozygotes per allele, linkage disequilibrium (LD) parameters, and neutrality using Slatkin’s implementation of the Ewens–Watterson homozygosity test. For each locus set, the population genomics software PyPop: Python for Population Genomics (version 1.0.2) was used [8,10]. A total of 1000 permutations were performed for significance testing. LD was quantified using Hedrick’s D′ and Cramer’s V statistic (Wn), with corresponding p values calculated as multiallelic extensions of the correlation measure. Hardy–Weinberg equilibrium (HWE) was evaluated using the chi-square test implemented in PyPop. Allele and haplotype frequencies across two to six loci were estimated using the expectation maximization (EM) algorithm. Demographic data were analyzed using IBM SPSS Statistics for Windows, version 25.0 (IBM Corp., Armonk, NY, USA). Kappa analysis was performed to assess concordance in HLA allele sequence rankings between groups.

## 3. Results

### 3.1. HLA allele frequencies

**Figure 1:**
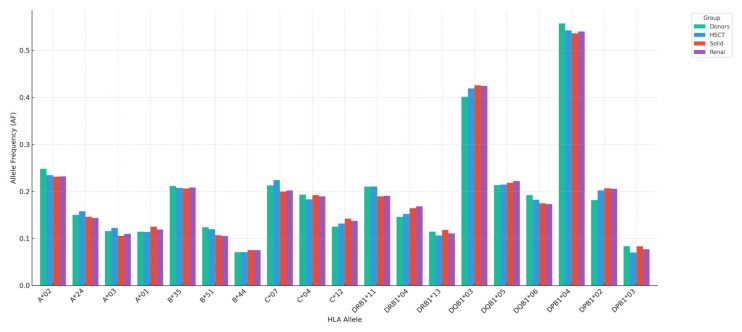
Allele frequencies across all study groups. The bar chart displays allele frequency (AF) values for each HLA allele, with study groups distinguished by different colors. Overall, distribution patterns are comparable across groups, although slight frequency differences are observed for certain alleles. The x-axis lists individual HLA alleles, whereas the y-axis represents the corresponding allele frequencies.

Figure 1 and Table 1 present a comparison of the most frequently observed allele frequencies across all groups. The most frequent alleles were largely comparable between donors and hematopoietic stem cell transplantation (HSCT) candidates. In both groups, the predominant alleles were A*02 at the HLA-A locus, B*35 at the HLA-B locus, C*07 at the HLA-C locus, DRB1*11 at the HLA-DRB1 locus, DQB1*03 at the HLA-DQB1 locus, and DPB1*04 at the HLA-DPB1 locus.

In contrast, solid organ transplant candidates exhibited a slightly different distribution at the HLA-A locus, where A*01 ranked among the three most frequent alleles instead of A*03. Although the leading alleles at the remaining loci were unchanged, several alleles demonstrated statistically significant frequency differences compared with donors. Specifically, A*31, A*33, B*15, B*42, B*51, B*54, DRB1*04, and DRB1*12 were observed at significantly higher frequencies in solid organ transplant candidates, whereas DRB1*11 was observed at a significantly lower frequency.

**Figure 2:**
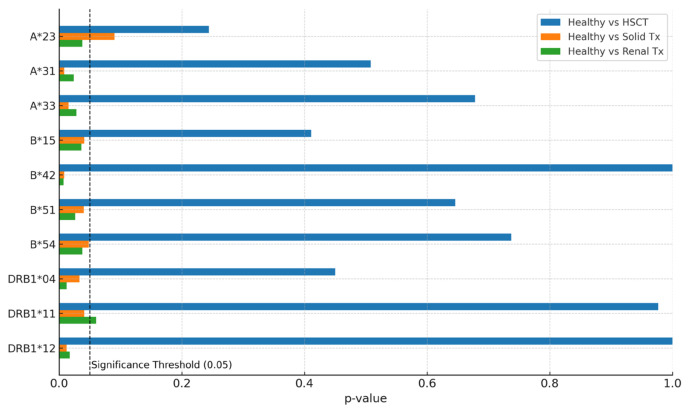
Comparison of p values for statistically significant differences in HLA alleles between groups. Horizontal bar chart illustrating alleles with statistically significant differences (p < 0.05) between healthy donors and transplant candidate groups: hematopoietic stem cell transplantation (HSCT), solid organ transplantation, and kidney transplantation. Each group is represented by a distinct color. Alleles are listed on the y-axis, and the corresponding p values are displayed on the x-axis. The black dashed vertical line indicates the statistical significance threshold (p = 0.05).

When kidney transplant candidates were analyzed separately, A*23 was observed at a significantly higher frequency compared with donors and the other transplant candidate groups. A complete list of allele frequencies for all groups is provided in Supplementary File 1 (Tables S1–S4). Alleles demonstrating statistically significant differences between groups are presented in Figure 2, and the corresponding p values for all compared alleles are provided in Supplementary File 1 (Table S5).

### 3.2. Neutrality testing and Hardy–Weinberg equilibrium analysis

The results of Slatkin’s implementation of the Ewens–Watterson homozygosity test for six HLA loci across all individuals are summarized in Supplementary File 1 (Table S6). Negative and statistically significant Fnd values were observed for all loci except HLA-DPB1, indicating excess heterozygosity and suggesting the presence of balancing selection at these loci.

**Figure 3:**
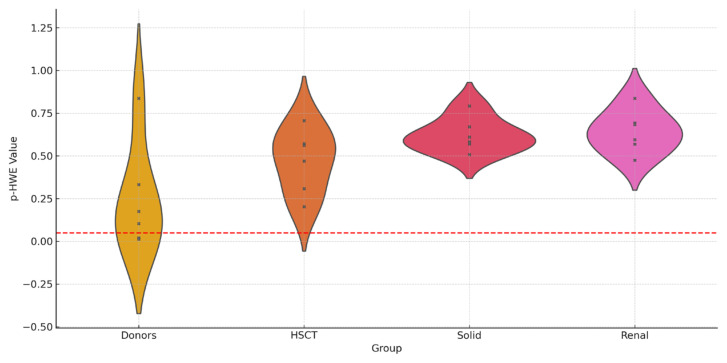
Distribution of Hardy–Weinberg equilibrium (HWE) p values across study groups. The distribution of p values for HWE testing across donors, HSCT candidates, solid organ transplant candidates, and renal transplant candidates is presented. The width of each violin reflects the density of data points across p value levels. The dashed horizontal line represents the statistical significance threshold (p = 0.05). Alleles in the donor group exhibit broader dispersion, with several p values below the significance threshold, whereas transplant candidate groups generally display higher HWE p values, suggesting less deviation from equilibrium.

Supplementary File 1 (Table S7) presents the results of HWE testing, including the ratio of observed to expected heterozygotes and the corresponding p values. No statistically significant deviation from HWE expectations was detected among donors at any locus except HLA-A and HLA-C, where mild deviations were observed (p < 0.05). No significant deviations were observed across all loci in the patient groups (HSCT candidates, solid organ transplant candidates, and renal transplant candidates). The distribution of p values for HWE testing across donors and transplant candidate groups is presented in Figure 3 (violin plot of HWE p values). The violin plot illustrates that the majority of loci in all groups exhibited HWE p values well above the 0.05 significance threshold, indicating overall concordance with HWE expectations. Mild deviations (p < 0.05) were primarily observed among donors, particularly at the HLA-A and HLA-C loci, whereas the HSCT, solid organ, and renal transplant candidate groups demonstrated narrower and more centralized distributions, consistent with genetically stable populations. Supplementary File 1 (Table S8) presents heterozygosity values for the most frequently observed alleles.

### 3.3. Genotype analysis

The most frequently observed genotypes are presented in Supplementary File 1 (Table S9).

### 3.4. Haplotype frequencies

Haplotype frequencies across three to six loci were estimated using the PyPop software. The most frequently observed haplotypes are presented in Table 2, and complete haplotype statistics are provided in Supplementary File 2. Because PyPop is limited to estimating fewer than 5000 haplotypes per analysis, A:B:DRB1 haplotypes in the donor group exceeded this threshold and were therefore reestimated using the GENE[RATE] pipeline to ensure computational accuracy and consistency. A comprehensive list of all estimated haplotypes is available in Supplementary File 2.

**Figure 4:**
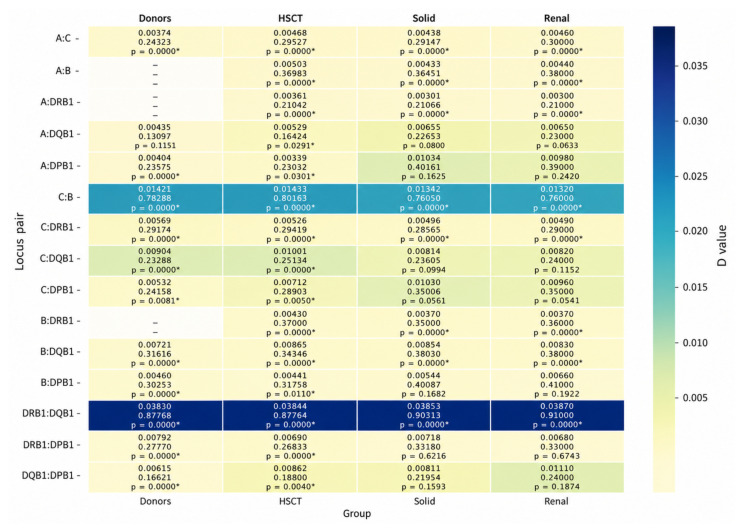
Comparative heat map of pairwise linkage disequilibrium (LD) values across study groups. The heat map summarizes pairwise LD parameters (D, D’, and p values) for selected HLA locus pairs across four groups: donors, HSCT candidates, solid organ transplant candidates, and kidney transplant candidates. Each cell displays LD statistics (D and D’) along with the corresponding p value. Darker colors indicate higher D values and stronger LD between locus pairs. Statistically significant p values (p < 0.05) indicate nonrandom associations between specific HLA loci. Missing data are left blank because the Pypop software cannot evaluate datasets exceeding 5000 haplotypes; consequently, no estimates were obtained for the A:B, B:DRB1, and A:DRB1 haplotypes.

Figure 4 presents a heat map of D′ values for pairwise locus combinations across the four groups. Pairwise LD was assessed using the log-likelihood under linkage equilibrium (L_0_), ln(L_1_), D′, and Wn. All pairwise comparisons were statistically significant (p < 0.05), except for the A:DQB1 locus pair in donors. All locus pairs demonstrated statistically significant LD (p < 0.05) in HSCT candidates. In contrast, the A:DQB1, A:DPB1, C:DQB1, C:DPB1, B:DPB1, DRB1:DPB1, and DQB1:DPB1 locus pairs did not reach statistical significance in solid organ transplant candidates (Supplementary File 1, Table S10).

## 4. Discussion

This study is significant because it provides a comprehensive HLA map that includes allele, genotype, and haplotype distributions across six HLA loci in both donors and transplant candidates. This comprehensive mapping enhances the understanding of genetic diversity within these populations and may inform strategies aimed at improving transplant compatibility and outcomes. Furthermore, it provides a foundation for future research investigating associations between HLA variation and disease susceptibility. Although the study population was limited to individuals from Central Anatolia and may not fully represent the entire population of Türkiye, to our knowledge, this report includes the largest number of cases published to date. Additionally, this study is, to our knowledge, the first to report HLA-DPB1 and six-locus HLA haplotypes in HSCT and solid organ transplant candidates in Türkiye. The absence of subgroup analyses among HSCT candidates represents a limitation and will be addressed in future studies focusing on disease-specific HLA associations.

### 4.1. Allele frequency

The three most frequent alleles were identical across all groups except at the HLA-A locus. The two most frequent alleles at the HLA-A locus were identical (A*02 and A*24); however, the third-ranked allele was A*03 in donors and HSCT candidates, whereas A*01 ranked third in solid organ and renal transplant candidates (Figure 1; Supplementary File 1, Table S4). Compared with previously published national data, our findings are consistent with most reports on allele and haplotype frequencies [3,4,6,11–13]. However, direct comparison with patient subgroups was not feasible because previous studies have not concurrently examined HSCT and solid organ transplant candidates.

No statistically significant differences were observed between HSCT candidates and donors. In contrast, A*31 (p = 0.008), A*33 (p = 0.015), B*15 (p = 0.041), B*42 (p = 0.008), B*51 (p = 0.04), B*54 (p = 0.048), DRB1*04 (p = 0.033), DRB1*11 (p = 0.041), and DRB1*12 (p = 0.012) demonstrated statistically significant frequency differences in solid organ transplant candidates (Figure 1). Solid organ transplant candidates, of whom 94.2% were kidney transplant candidates diagnosed with end-stage renal disease, 1.2% were liver transplant candidates, and 4.5% were corneal transplant candidates, represented a relatively homogeneous group, which may have contributed to the observed consistency in allele frequency patterns.

Kidney transplant candidates were therefore analyzed separately and compared with donors and the overall transplant candidate groups. Statistically significant differences in the allele frequencies of A*23 and DRB1*11 were observed in renal transplant candidates compared with donors. Although A*23 demonstrated a statistically significant difference in renal transplant candidates, no significant difference was observed in the remaining solid organ transplant subgroups. DRB1*11 was observed at a significantly lower frequency in solid organ transplant candidates compared with donors.; however, this difference was not specifically observed in the renal transplant subgroup. In contrast, HSCT candidates represented a more heterogeneous population, comprising various subtypes of leukemia, lymphoma, and other hematologic disorders, including aplastic anemia.

Kappa analysis assessing concordance in allele sequence ranking indicated that, although overall agreement between the two groups was high, locus-specific differences in allele ranking were observed at loci other than HLA-DRB1 and HLA-DQB1. In HSCT candidates, concordance in allele ranking was 80% at the HLA-A locus, 42.9% at HLA-B, 42.1% at HLA-DPB1, and 66.7% at HLA-C. Differences in allele ranking became more pronounced beyond the fifth most frequent allele. Allele ranking patterns at the HLA-DRB1 and HLA-DPB1 loci were comparable (Supplementary File 1, Tables S11–S16).

At the HLA-A locus, the third most frequent allele was A*03 in donors and HSCT candidates, whereas A*01 ranked third in solid organ transplant candidates (Figure 1; Supplementary File 1, Table S11). A*23 was observed at a higher frequency in both patient groups compared with donors. A*33 and A*31 were observed at higher frequencies in solid organ transplant candidates compared with donors and HSCT candidates. In contrast, A*68 was observed at a higher frequency in donors compared with both patient groups. The A*50 allele was detected exclusively in the HSCT candidate group.

At the HLA-B locus, the three most frequent alleles were ranked identically across all groups. B*52 was observed at a higher frequency in HSCT candidates, whereas B*38, B*08, and B*50 were more frequent in donors (Figure 1; Supplementary File 1, Table S12).

At the HLA-C locus, the three most frequent alleles were identical across all groups. C*14 and C*16 were observed at higher frequencies in donors, whereas C*02 and C*04 were more frequent in both patient groups (Figure 1; Supplementary File 1, Table S13).

At the HLA-DRB1 locus, the three most frequent alleles showed identical ranking across groups. DRB1*03 and DRB1*12 were observed at higher frequencies in both patient groups compared with donors (Figure 1; Supplementary File 1, Table S14).

No differences in allele frequency ranking were observed at the HLA-DQB1 locus across groups (Figure 1; Supplementary File 1, Table S15). At the HLA-DPB1 locus, the three most frequent alleles were ranked identically across all groups. In the HSCT candidate group, DPB1*09, DPB1*14, and DPB1*01 were observed at higher frequencies compared with donors, whereas DPB1*13, DPB1*23, DPB1*11, and DPB1*10 were observed at lower frequencies. In solid organ transplant candidates, DPB1*13, DPB1*14, DPB1*23, and DPB1*11 were observed at higher frequencies compared with donors, whereas DPB1*17, DPB1*09, DPB1*01, and DPB1*10 were observed at lower frequencies. Renal transplant candidates and the overall solid organ transplant group demonstrated identical allele ranking pattern (Figure 1; Supplementary File 1, Table S16).

Studies investigating HLA associations in liver and corneal transplantation are less numerous than those in renal transplantation. According to previous reports, different HLA alleles associated with liver or corneal diseases may confer either risk or protective effects. However, because the number of liver and corneal transplant candidates in the study population was insufficient for meaningful polymorphism analysis, these subgroups were not evaluated separately. Numerous studies have investigated which HLA alleles exert protective or adverse effects on kidney function, and various alleles have been documented across populations. Proposed explanations for these variations include differences in ethnic background, population-specific effects of HLA alleles, and distinct HLA associations related to the underlying diseases leading to end-stage renal disease (ESRD). A review of previous studies indicates that the A*31 [14], A*33 [15], B*15 [14,16], B*42 [17], B*51 [17,18], B*54 [19], DRB1*04 [14,19], DRB1*11 [14,20,21], and DRB1*12 [14,16,22–24] alleles, which demonstrated significant differences in the present study, have also been reported in association with ESRD or transplant populations in other cohorts [25]. To the best of our knowledge, the significant difference observed for A*23 in renal transplant candidates has not been previously reported in the literature. Consistent with our findings, A*33 has been reported in Azerbaijan [15], B*42 and B*51 in Brazil [17], and B*51 in Venezuela [18] in Class I HLA analyses. Furthermore, studies from China, Taiwan, Brazil, Pakistan, Indonesia, and Mexico have reported associations between Class II HLA alleles—specifically DRB1*04 [14,19,20,26], DRB1*11 [14,20,21], and DRB1*12 [14,22,23,24,27]—and ESRD. Although some studies from China have reported similar alleles, our findings additionally identified A*33, B*42, and B*51, which were not consistently reported across those cohorts [14,16,19]. In contrast, no overlapping allele associations were identified in comparison with the Romanian study [28]. In a study of living-related kidney transplant recipients and donors in Nepal, high-frequency alleles included A*11, A*24, A*33, B*15, B*35, B*40, DRB1*15, DRB1*12, and DRB1*04 [29].

The HSCT cohort in our study was heterogeneous, including patients with leukemia, lymphoma, and other hematologic diseases. Because disease-specific subgroup analyses were not performed, this heterogeneity represents a limitation of the present study. The overall allele distribution in HSCT candidates was largely comparable to that observed in donors, suggesting that this cohort reflects the regional HLA background. No disease-specific conclusions can be drawn from the present data. Future studies focusing on more homogeneous diagnostic subgroups may clarify potential allele–disease associations. Previous studies have emphasized the relevance of HLA allele and haplotype diversity in HSCT settings, particularly in relation to regional population characteristics [30–39].

### 4.2. Haplotype frequency evaluation

In the present study, the most frequent A:B:C haplotype across all groups was 24~35~04. The most frequent A:B:DRB1 haplotype was identical in donors and HSCT candidates (24~35~11) but differed in solid organ transplant candidates (03~35~04). Previously reported A:B:DRB1 haplotypes in hematologic disease studies from Türkiye include the following: in the Black Sea region, 02~35~11 in Hodgkin lymphoma, 02~51~11 in non-Hodgkin lymphoma [36], 03~51~11 in acute myeloblastic leukemia (AML), and 02~35~01 in acute lymphoblastic leukemia (ALL) [32]; in the Aegean region, 24~35~11 in patients and 01~08~03 in controls [33]; and in the Central Anatolia region, 02~35~13 in ALL patients, 01~08~03 in AML patients, and 02~35~04 in controls [30]. The A:B:DRB1 haplotypes associated with ESRD in the present study differed from those reported in China, Romania, and Indonesia [16,19,28], as well as from haplotypes reported in hematologic disease cohorts from Egypt and Mexico [35,39]. In the present study, the dominant A:B:C:DRB1 haplotype was 24~35~04~11 in donors and HSCT candidates, whereas it was 01~08~07~03 in solid organ transplant candidates (Table 2). The dominant five-locus A:B:C:DRB1:DQB1 haplotype was 24~35~04~11~03 in donors and HSCT candidates, whereas 02~35~04~11~03 was observed in solid organ transplant candidates. In the present study, the haplotype 11~35~04~11~03 ranked third among solid organ transplant candidates but fifteenth among donors. Studies from China reported different haplotype patterns [15,17], whereas studies from Türkiye demonstrated partial similarities [33]. The most frequent six-locus A:B:C:DRB1:DQB1:DPB1 haplotypes were 02~35~04~11~03~04 in donors, 24~35~04~11~03~04 in HSCT candidates, and 24~35~04~04~03~04 in solid organ transplant candidates. The most frequent haplotypes in kidney transplant candidates were A:B:C (11~35~04), A:B:DRB1 (01~08~03), and A:B:C:DRB1 (24~35~04~04), which differed from those observed in the other groups.

Recent studies have emphasized the importance of high-resolution HLA typing and population-specific allele distributions in both HSCT and solid organ transplantation settings [40–43].

This study has several limitations that should be acknowledged. Although the sample size represents the largest HLA dataset reported to date in Türkiye, the study population was restricted to the Central Anatolian region, which may limit the generalizability of the findings to the broader national population. Furthermore, although transplant candidates were categorized into HSCT and solid organ groups, detailed subgroup analyses—particularly within the HSCT cohort—could not be performed because of clinical heterogeneity across diagnoses such as AML, ALL, and MDS. To address this limitation, a follow-up study focusing on more homogeneous HSCT subtypes is planned. Additionally, because the majority of transplant candidates were kidney transplant recipients, meaningful comparisons across other solid organ subgroups, such as liver or corneal transplantation, were limited. Lastly, the use of low-resolution genotyping methods (PCR-SSO and PCR-SSP) may have limited the detection of rare allele subtypes; therefore, future studies employing high-resolution sequencing technologies are warranted to achieve greater allele-level resolution and improved haplotype accuracy.

In conclusion, allele frequencies at six HLA loci (HLA-A, HLA-B, HLA-C, HLA-DRB1, HLA-DQB1, and HLA-DPB1), as well as haplotype frequencies across three-, four-, five-, and six-locus combinations and genotype frequencies, were compared between healthy donors from our region and candidates for HSCT and solid organ transplantation. This study provides a comprehensive comparison that, to our knowledge, has not previously been documented in Türkiye.

In addition, consideration of allele and haplotype distributions during donor selection may contribute to optimizing transplant compatibility and potentially improving long-term transplant outcomes. To advance HLA research, larger and more geographically diverse populations should be included in future investigations. Although the study does not encompass the entire Turkish population, it has the potential to contribute meaningfully to mapping HLA distribution patterns nationwide. These findings may support future research initiatives and enhance the understanding of HLA variation at the national level.

## Supplementary Information





## Figures and Tables

**Table 1:** Most frequent allele frequencies at the HLA-A, HLA-B, HLA-C, HLA-DRB1, HLA-DQB1, and HLA-DPB1 loci across study groups.

Donors			HSCT candidates			Solid tx candidates			Renal tx candidates		
Allele	AF	2n	Allele	AF	2n	Allele	AF	2n	Allele	AF	2n
A*02	0.24821	12,944	A*02	0.23475	2164	A*02	0.23140	1936	A*02	0.23191	1824
A*24	0.15019	12,944	A*24	0.15804	2164	A*24	0.14618	1936	A*24	0.14364	1824
A*03	0.11570	12,944	A*03	0.12246	2164	A*01	*0.12500*	*1936*	A*01	0.11897	1824
A*01	0.11437	12,944	A*01	0.11368	2164	A*03	0.10537	1936	A*03	0.10965	1824
B*35	0.21154	12,944	B*35	0.20749	2164	B*35	0.20610	1936	B*35	0.20833	1824
B*51	0.12348	12,944	B*51	0.11969	2164	B*51	0.10692	1936	B*51	0.10526	1824
B*44	0.07069	12,944	B*44	0.07070	2164	B*44	0.07541	1936	B*44	0.07511	1824
C*07	0.21314	3852	C*07	0.22425	1262	C*07	0.19981	1056	C*07	0.20236	1018
C*04	0.19315	3852	C*04	0.18304	1262	C*04	0.19223	1056	C*04	0.18959	1018
C*12	0.12513	3852	C*12	0.13154	1262	C*12	0.14205	1056	C*12	0.13752	1018
DRB1*11	0.20998	12,944	DRB1*11	0.21026	2164	DRB1*11	0.18957	1936	DRB1*11	0.19079	1824
DRB1*04	0.14575	12,944	DRB1*04	0.15203	2164	DRB1*04	0.16426	1936	DRB1*04	0.16831	1824
DRB1*13	0.11437	12,944	DRB1*13	0.10628	2164	DRB1*13	0.11777	1936	DRB1*13	0.11075	1824
DQB1*03	0.40143	1400	DQB1*03	0.41930	1202	DQB1*03	0.42576	458	DQB1*03	0.42444	450
DQB1*05	0.21357	1400	DQB1*05	0.21464	1202	DQB1*05	0.21834	458	DQB1*05	0.22222	450
DQB1*06	0.19214	1400	DQB1*06	0.18220	1202	DQB1*06	0.17467	458	DQB1*06	0.17333	450
DPB1*04	0.55743	1184	DPB1*04	0.54251	988	DPB1*04	0.53623	276	DPB1*04	0.54044	272
DPB1*02	0.18159	1184	DPB1*02	0.20243	988	DPB1*02	0.20652	276	DPB1*02	0.20588	272
DPB1*03	0.08361	1184	DPB1*03	0.06984	988	DPB1*03	0.08333	276	DPB1*03	0.07721	272

Abbreviations: AF, allele frequency; 2n, allele count; Tx, transplantation; HSCT, hematopoietic stem cell transplantation.

**Table 2:** Most frequent haplotypes across study groups.

Donors		HSCT		Solid Tx candidates		Renal Tx candidates	
Haplotype name	HF	Haplotype name	HF	Haplotype name	HF	Haplotype name	HF
**A:B:C**							
**24~35~04**	**0.03432**	**24~35~04**	**0.04436**	**24~35~04**	**0.03558**	**11~35~04**	**0.03482**
02~35~04	0.03158	03~35~04	0.03037	11~35~04	0.03373	02~35~04	0.03228
03~35~04	0.02599	23~49~07	0.02489	02~35~04	0.02863	24~35~04	0.03107
**A:B.DR**							
**24~35~11**	**0.0163**	**24~35~11**	**0.01979**	**03~35~04**	**0.01084**	**01~08~03**	**0.01747**
02~51~11	0.0135	03~35~11	0.01383	24~07~12	0.00101	02~51~04	0.01487
02~51~04	0.0116	02~51~11	0.01231	02~55~11	0.00101	03~35~04	0.01332
**DRB1:DQB1:DPB1**							
**11~03~04**	**0.14238**	**11~03~04**	**0.13522**	**11~03~04**	**0.12284**	**11~03~04**	**0.12404**
15~06~04	0.05765	04~03~04	0.06252	04~03~04	0.07992	04~03~04	0.08201
13~06~04	0.05546	15~06~04	0.05457	03~02~04	0.06390	03~02~04	0.06529
**A:B:C:DRB1**							
**24~35~04~11**	**0.0155**	**24~35~04~11**	**0.01888**	**01~08~07~03**	**0.01678**	**24~35~04~04**	**0.01681**
02~35~04~11	0.01278	23~49~07~11	0.01471	24~35~04~11	0.01548	01~08~07~03	0.01656
23~49~07~11	0.01154	03~35~04~11	0.01373	24~35~04~04	0.01406	02~35~04~11	0.01573
**A:B:C:DRB1:DQB1**							
**24~35~04~11~03**	**0.0251**	**24~35~04~11~03**	**0.03138**	**02~35~04~11~03**	**0.02374**	**02~35~04~11~03**	**0.02928**
02~35~04~11~03	0.0232	23~49~07~11~03	0.01385	24~35~04~04~03	0.01991	24~35~04~04~03	0.01958
02~35~04~04~03	0.01514	02~13~06~07~02	0.01316	01~08~07~03~02	0.0177	01~08~07~03~02	0.01577
**A:B:C:DRB1:DQB1:DPB1**							
**02~35~04~11~03~04**	**0.02693**	**24~35~04~11~03~04**	**0.01911**	**24~35~04~04~03~04**	**0.02612**	**24~35~04~04~03~04**	**0.02652**
24~35~04~11~03~04	0.01430	02~35~04~04~03~02	0.01418	11~35~04~11~03~04	0.01866	11~35~04~11~03~04	0.01894
11~52~12~15~06~04	0.01331	23~49~07~11~03~04	0.01280	24~13~06~07~02~04	0.01493	01~08~07~03~02~04	0.01515

Abbreviations: HF, haplotype frequency; Tx, transplantation; HSCT, hematopoietic stem cell transplantation.
