# Erratum to “HLA allele and haplotype diversity in Central Anatolia: a comparative analysis of donors and transplant candidates across six HLA loci (HLA-A, HLA-B, HLA-C, HLA-DRB1, HLA-DQB1, and HLA-DPB1)” [Turkish Journal of Medical Sciences 56 (3) 2026 817–827]

**DOI:** 10.55730/1300-0144.6380

**Published:** 2026-08-01

**Authors:** Emel YANTIR, Eren GÜNDÜZ

**Affiliations:** 1 Department of Immunology, Faculty of Medicine, Eskişehir Osmangazi University, Eskişehir, Turkiye; 2 Division of Hematology, Department of Internal Medicine, Faculty of Medicine, Eskişehir Osmangazi University, Eskişehir, Turkiye

In Article No. 56-3-22, entitled “HLA Allele and Haplotype Diversity in Central Anatolia: A Comparative Analysis of Donors and Transplant Candidates across Six HLA Loci (HLA-A, HLA-B, HLA-C, HLA-DRB1, HLA-DQB1, and HLA-DPB1)”, an error was identified in the DOI of the Aperta link provided in the Supplementary Materials section, as the final digit of the DOI was inadvertently omitted.

The correct and complete version of the link is provided below on a separate page.

https://aperta.ulakbim.gov.tr/records/287387

