# Supplementary material for: HLA allele and haplotype diversity in Central Anatolia: a comparative analysis of donors and transplant candidates across six HLA loci (HLA-A, HLA-B, HLA-C, HLA-DRB1, HLA-DQB1, and HLA-DPB1)
Source: Turk J Med Sci. 2026 Feb 16;56(3):817–27. doi: 10.55730/1300-0144.6216 (PMC13398592; doi:10.55730/1300-0144.6216)
Supplement: Supplementary file 1 [file Supplementfile_1_controled.docx]

**HLA allele and haplotype diversity in Central Anatolia: a comparative analysis of donors and transplant candidates across six HLA loci (HLA-A, HLA-B, HLA-C, HLA-DRB1, HLA-DQB1, and HLA-DPB1)**

**Emel YANTIR^1,^*, Eren GÜNDÜZ^2^**

^1^Department of Immunology, Faculty of Medicine, Eskisehir Osmangazi University, Eskisehir, Turkiye

^2^Division of Hematology, Department of Internal Medicine, Faculty of Medicine, Eskisehir Osmangazi University, Eskisehir, Turkiye

***Correspondence:** emel.yantir@ogu.edu.tr

ORCIDs:

Emel YANTIR: <https://orcid.org/0000-0002-4965-8730>

Eren GÜNDÜZ: <https://orcid.org/0000-0001-7455-2949>

**Abstract**

**Background/aim:** Human leukocyte antigen (HLA) polymorphisms play a central role in immune recognition, disease susceptibility, and transplant success. The distribution of HLA alleles and haplotypes varies among ethnic and geographic populations. Despite several regional studies conducted in Türkiye, large-scale and comprehensive investigations comparing HLA profiles between healthy individuals and transplant candidates remain limited. The aim of this study was to compare the distribution of HLA profiles among donors, hematopoietic stem cell transplantation (HSCT) candidates, and solid organ transplant candidates.

**Materials and methods:** A total of 8472 individuals were included in the study, comprising 6422 donors and 2050 transplant candidates. HLA genotyping was performed using polymerase chain reaction with sequence-specific primers and polymerase chain reaction with sequence-specific oligonucleotide probes. Allele, genotype, and haplotype frequencies were calculated using the PyPop (version 1.2.0) and GENE[RATE] software tools.

**Results:** The most frequent alleles at the HLA-A, HLA-B, HLA-C, HLA-DRB1, HLA-DQB1, and HLA-DPB1 loci were similar across all groups, with A*02, B*35, C*07, DRB1*11, DQB1*03, and DPB1*04 observed as the predominant alleles. Several alleles were significantly overrepresented in solid organ transplant candidates compared with donors, including A*31, A*33, B*15, B*42, B*51, B*54, DRB1*04, and DRB1*12, whereas DRB1*11 was significantly underrepresented (p < 0.05). A*23 was also observed at a significantly higher frequency in kidney transplant candidates. No significant allele differences were observed between HSCT candidates and donors. Haplotype analysis revealed that the most prevalent six-locus haplotype (A:B:C:DRB1:DQB1:DPB1) was 02~35~04~11~03~04 in donors, whereas 24~35~04~11~03~04 was observed in HSCT candidates and 24~35~04~04~03~04 in solid organ transplant candidates.

**Conclusion:** To our knowledge, this study represents the largest and most comprehensive analysis of HLA distribution in Türkiye to date and the first to report six-locus HLA haplotypes, including the HLA-DPB1 locus, in transplant candidates. The results indicate that solid organ transplant candidates exhibit significant differences in HLA allele and haplotype frequencies compared with healthy donors. These findings underscore the importance of region-specific HLA data in optimizing donor selection and potentially improving transplant outcomes.

**Key words:** Human leukocyte antigen, six loci, allele frequency, haplotype frequency, Türkiye, transplantation

1. Introduction

The human leukocyte antigen (HLA) system is a highly polymorphic gene region encoded on the short arm of chromosome 6 in the human genome. As of March 2025, a total of 41,003 alleles are listed in the IMGT/HLA database (version 3.59) [1]. Tissue compatibility is directly associated with graft rejection and posttransplant survival. Traditional evaluations of the HLA-A, HLA-B, and HLA-DRB1 loci are recommended for solid organ transplantation, whereas assessment of six loci is recommended for hematopoietic stem cell transplantation (HSCT) [2].

HLA gene frequencies vary according to ethnic background and geographic distribution. An increasing number of studies worldwide report HLA distribution from different nations and populations. Although studies have reported HLA distributions across different regions of Türkiye, investigations involving large population cohorts remain limited [3–6].

This study was designed as a follow-up to our previously published research on HLA distribution in donors [7]. HLA data from donors were compared with those from HSCT and solid organ transplant candidates. Although the study population was limited to the Central Anatolian region, to our knowledge, this investigation represents the largest and most detailed HLA population genetics analysis conducted in Türkiye to date.

1. Materials and methods
   1. Populations

Data from 8472 individuals who underwent HLA typing at the Eskişehir Osmangazi University Hospital Tissue Typing Laboratory between 2001 and 2023 were analyzed. The study population comprised 6422 healthy donors and 2050 transplant candidates, including 1082 HSCT candidates and 968 solid organ transplant candidates. Solid organ transplant candidates consisted of kidney (n = 912), liver (n = 12), and corneal (n = 44) transplant recipients. HSCT was planned for patients diagnosed with leukemia or myelodysplastic syndrome (MDS) (n = 777), lymphoma (n = 117), and other hematologic disorders (n = 188). A total of six HLA loci (HLA-A, HLA-B, HLA-C, HLA-DRB1, HLA-DQB1, and HLA-DPB1) were evaluated using DNA-based molecular typing methods, including sequence-specific oligonucleotide probes (PCR-SSO) and sequence-specific primers (PCR-SSP). Individuals were required to have data for at least three loci (HLA-A, HLA-B, and HLA-DRB1) to be included in the analysis. The proportions of female and male participants were 46.7% and 53.3% among donors, 43.5% and 56.5% among HSCT candidates, and 44.8% and 55.2% among solid organ transplant candidates, respectively. No statistically significant difference in sex distribution was observed among the groups (p > 0.05).

The study was conducted in accordance with the principles of the Declaration of Helsinki and was approved by the Eskişehir Osmangazi University Faculty of Medicine Ethics Committee (approval no: 2026–55).

- 1. HLA typing methods

HLA typing was performed using the PCR-SSO and/or PCR-SSP methods as previously described for peripheral blood samples [7]. All HLA data generated prior to 2010 were reannotated according to the current IMGT/HLA nomenclature. All HLA data were validated prior to analysis using the quality control tools implemented in the GENE[RATE] software [8,9].

- 1. Statistical analysis

All statistical analyses were conducted using the methods described in our previous study to ensure methodological consistency [7]. The HLA data were used to estimate genotype and allele proportions, haplotype frequencies, the number of common heterozygotes per allele, linkage disequilibrium (LD) parameters, and neutrality using Slatkin’s implementation of the Ewens–Watterson homozygosity test. For each locus set, the population genomics software PyPop: Python for Population Genomics (version 1.0.2) was used [8,10]. A total of 1000 permutations were performed for significance testing. LD was quantified using Hedrick’s D′ and Cramer’s V statistic (Wn), with corresponding p values calculated as multiallelic extensions of the correlation measure. Hardy–Weinberg equilibrium (HWE) was evaluated using the chi-square test implemented in PyPop. Allele and haplotype frequencies across two to six loci were estimated using the expectation maximization (EM) algorithm. Demographic data were analyzed using IBM SPSS Statistics for Windows, version 25.0 (IBM Corp., Armonk, NY, USA). Kappa analysis was performed to assess concordance in HLA allele sequence rankings between groups.

1. Results
   1. HLA allele frequencies

Figure 1 and Table 1 present a comparison of the most frequently observed allele frequencies across all groups. The most frequent alleles were largely comparable between donors and hematopoietic stem cell transplantation (HSCT) candidates. In both groups, the predominant alleles were A*02 at the HLA-A locus, B*35 at the HLA-B locus, C*07 at the HLA-C locus, DRB1*11 at the HLA-DRB1 locus, DQB1*03 at the HLA-DQB1 locus, and DPB1*04 at the HLA-DPB1 locus.

In contrast, solid organ transplant candidates exhibited a slightly different distribution at the HLA-A locus, where A*01 ranked among the three most frequent alleles instead of A*03. Although the leading alleles at the remaining loci were unchanged, several alleles demonstrated statistically significant frequency differences compared with donors. Specifically, A*31, A*33, B*15, B*42, B*51, B*54, DRB1*04, and DRB1*12 were observed at significantly higher frequencies in solid organ transplant candidates, whereas DRB1*11 was observed at a significantly lower frequency.

When kidney transplant candidates were analyzed separately, A*23 was observed at a significantly higher frequency compared with donors and the other transplant candidate groups. A complete list of allele frequencies for all groups is provided in Supplementary File 1 (Tables S1–S4). Alleles demonstrating statistically significant differences between groups are presented in Figure 2, and the corresponding p values for all compared alleles are provided in Supplementary File 1 (Table S5).

- 1. Neutrality testing and Hardy–Weinberg equilibrium analysis

The results of Slatkin’s implementation of the Ewens–Watterson homozygosity test for six HLA loci across all individuals are summarized in Supplementary File 1 (Table S6). Negative and statistically significant Fnd values were observed for all loci except HLA-DPB1, indicating excess heterozygosity and suggesting the presence of balancing selection at these loci.

Supplementary File 1 (Table S7) presents the results of HWE testing, including the ratio of observed to expected heterozygotes and the corresponding p values. No statistically significant deviation from HWE expectations was detected among donors at any locus except HLA-A and HLA-C, where mild deviations were observed (p < 0.05). No significant deviations were observed across all loci in the patient groups (HSCT candidates, solid organ transplant candidates, and renal transplant candidates). The distribution of p values for HWE testing across donors and transplant candidate groups is presented in Figure 3 (violin plot of HWE p values). The violin plot illustrates that the majority of loci in all groups exhibited HWE p values well above the 0.05 significance threshold, indicating overall concordance with HWE expectations. Mild deviations (p < 0.05) were primarily observed among donors, particularly at the HLA-A and HLA-C loci, whereas the HSCT, solid organ, and renal transplant candidate groups demonstrated narrower and more centralized distributions, consistent with genetically stable populations. Supplementary File 1 (Table S8) presents heterozygosity values for the most frequently observed alleles.

- 1. Genotype analysis

The most frequently observed genotypes are presented in Supplementary File 1 (Table S9).

- 1. Haplotype frequencies

Haplotype frequencies across three to six loci were estimated using the PyPop software. The most frequently observed haplotypes are presented in Table 2, and complete haplotype statistics are provided in Supplementary File 2. Because PyPop is limited to estimating fewer than 5000 haplotypes per analysis, A:B:DRB1 haplotypes in the donor group exceeded this threshold and were therefore reestimated using the GENE[RATE] pipeline to ensure computational accuracy and consistency. A comprehensive list of all estimated haplotypes is available in Supplementary File 2.

Figure 4 presents a heat map of D′ values for pairwise locus combinations across the four groups. Pairwise LD was assessed using the log-likelihood under linkage equilibrium (L_0_), ln(L_1_), D′, and Wn. All pairwise comparisons were statistically significant (p < 0.05), except for the A:DQB1 locus pair in donors. All locus pairs demonstrated statistically significant LD (p < 0.05) in HSCT candidates. In contrast, the A:DQB1, A:DPB1, C:DQB1, C:DPB1, B:DPB1, DRB1:DPB1, and DQB1:DPB1 locus pairs did not reach statistical significance in solid organ transplant candidates (Supplementary File 1, Table S10).

1. Discussion

This study is significant because it provides a comprehensive HLA map that includes allele, genotype, and haplotype distributions across six HLA loci in both donors and transplant candidates. This comprehensive mapping enhances the understanding of genetic diversity within these populations and may inform strategies aimed at improving transplant compatibility and outcomes. Furthermore, it provides a foundation for future research investigating associations between HLA variation and disease susceptibility. Although the study population was limited to individuals from Central Anatolia and may not fully represent the entire population of Türkiye, to our knowledge, this report includes the largest number of cases published to date. Additionally, this study is, to our knowledge, the first to report HLA-DPB1 and six-locus HLA haplotypes in HSCT and solid organ transplant candidates in Türkiye. The absence of subgroup analyses among HSCT candidates represents a limitation and will be addressed in future studies focusing on disease-specific HLA associations.

- 1. Allele frequency

The three most frequent alleles were identical across all groups except at the HLA-A locus. The two most frequent alleles at the HLA-A locus were identical (A*02 and A*24); however, the third-ranked allele was A*03 in donors and HSCT candidates, whereas A*01 ranked third in solid organ and renal transplant candidates (Figure 1; Supplementary File 1, Table S4). Compared with previously published national data, our findings are consistent with most reports on allele and haplotype frequencies [3,4,6,11–13]. However, direct comparison with patient subgroups was not feasible because previous studies have not concurrently examined HSCT and solid organ transplant candidates.

No statistically significant differences were observed between HSCT candidates and donors. In contrast, A*31 (p = 0.008), A*33 (p = 0.015), B*15 (p = 0.041), B*42 (p = 0.008), B*51 (p = 0.04), B*54 (p = 0.048), DRB1*04 (p = 0.033), DRB1*11 (p = 0.041), and DRB1*12 (p = 0.012) demonstrated statistically significant frequency differences in solid organ transplant candidates (Figure 1). Solid organ transplant candidates, of whom 94.2% were kidney transplant candidates diagnosed with end-stage renal disease, 1.2% were liver transplant candidates, and 4.5% were corneal transplant candidates, represented a relatively homogeneous group, which may have contributed to the observed consistency in allele frequency patterns.

Kidney transplant candidates were therefore analyzed separately and compared with donors and the overall transplant candidate groups. Statistically significant differences in the allele frequencies of A*23 and DRB1*11 were observed in renal transplant candidates compared with donors. Although A*23 demonstrated a statistically significant difference in renal transplant candidates, no significant difference was observed in the remaining solid organ transplant subgroups. DRB1*11 was observed at a significantly lower frequency in solid organ transplant candidates compared with donors.; however, this difference was not specifically observed in the renal transplant subgroup. In contrast, HSCT candidates represented a more heterogeneous population, comprising various subtypes of leukemia, lymphoma, and other hematologic disorders, including aplastic anemia.

Kappa analysis assessing concordance in allele sequence ranking indicated that, although overall agreement between the two groups was high, locus-specific differences in allele ranking were observed at loci other than HLA-DRB1 and HLA-DQB1. In HSCT candidates, concordance in allele ranking was 80% at the HLA-A locus, 42.9% at HLA-B, 42.1% at HLA-DPB1, and 66.7% at HLA-C. Differences in allele ranking became more pronounced beyond the fifth most frequent allele. Allele ranking patterns at the HLA-DRB1 and HLA-DPB1 loci were comparable (Supplementary File 1, Tables S11–S16).

At the HLA-A locus, the third most frequent allele was A*03 in donors and HSCT candidates, whereas A*01 ranked third in solid organ transplant candidates (Figure 1; Supplementary File 1, Table S11). A*23 was observed at a higher frequency in both patient groups compared with donors. A*33 and A*31 were observed at higher frequencies in solid organ transplant candidates compared with donors and HSCT candidates. In contrast, A*68 was observed at a higher frequency in donors compared with both patient groups. The A*50 allele was detected exclusively in the HSCT candidate group.

At the HLA-B locus, the three most frequent alleles were ranked identically across all groups. B*52 was observed at a higher frequency in HSCT candidates, whereas B*38, B*08, and B*50 were more frequent in donors (Figure 1; Supplementary File 1, Table S12).

At the HLA-C locus, the three most frequent alleles were identical across all groups. C*14 and C*16 were observed at higher frequencies in donors, whereas C*02 and C*04 were more frequent in both patient groups (Figure 1; Supplementary File 1, Table S13).

At the HLA-DRB1 locus, the three most frequent alleles showed identical ranking across groups. DRB1*03 and DRB1*12 were observed at higher frequencies in both patient groups compared with donors (Figure 1; Supplementary File 1, Table S14).

No differences in allele frequency ranking were observed at the HLA-DQB1 locus across groups (Figure 1; Supplementary File 1, Table S15). At the HLA-DPB1 locus, the three most frequent alleles were ranked identically across all groups. In the HSCT candidate group, DPB1*09, DPB1*14, and DPB1*01 were observed at higher frequencies compared with donors, whereas DPB1*13, DPB1*23, DPB1*11, and DPB1*10 were observed at lower frequencies. In solid organ transplant candidates, DPB1*13, DPB1*14, DPB1*23, and DPB1*11 were observed at higher frequencies compared with donors, whereas DPB1*17, DPB1*09, DPB1*01, and DPB1*10 were observed at lower frequencies. Renal transplant candidates and the overall solid organ transplant group demonstrated identical allele ranking pattern (Figure 1; Supplementary File 1, Table S16).

Studies investigating HLA associations in liver and corneal transplantation are less numerous than those in renal transplantation. According to previous reports, different HLA alleles associated with liver or corneal diseases may confer either risk or protective effects. However, because the number of liver and corneal transplant candidates in the study population was insufficient for meaningful polymorphism analysis, these subgroups were not evaluated separately. Numerous studies have investigated which HLA alleles exert protective or adverse effects on kidney function, and various alleles have been documented across populations. Proposed explanations for these variations include differences in ethnic background, population-specific effects of HLA alleles, and distinct HLA associations related to the underlying diseases leading to end-stage renal disease (ESRD). A review of previous studies indicates that the A*31 [14], A*33 [15], B*15 [14,16], B*42 [17], B*51 [17,18], B*54 [19], DRB1*04 [14,19], DRB1*11 [14,20,21], and DRB1*12 [14,16,22–24] alleles, which demonstrated significant differences in the present study, have also been reported in association with ESRD or transplant populations in other cohorts [25]. To the best of our knowledge, the significant difference observed for A*23 in renal transplant candidates has not been previously reported in the literature. Consistent with our findings, A*33 has been reported in Azerbaijan [15], B*42 and B*51 in Brazil [17], and B*51 in Venezuela [18] in Class I HLA analyses. Furthermore, studies from China, Taiwan, Brazil, Pakistan, Indonesia, and Mexico have reported associations between Class II HLA alleles—specifically DRB1*04 [14,19,20,26], DRB1*11 [14,20,21], and DRB1*12 [14,22,23,24,27]—and ESRD. Although some studies from China have reported similar alleles, our findings additionally identified A*33, B*42, and B*51, which were not consistently reported across those cohorts [14,16,19]. In contrast, no overlapping allele associations were identified in comparison with the Romanian study [28]. In a study of living-related kidney transplant recipients and donors in Nepal, high-frequency alleles included A*11, A*24, A*33, B*15, B*35, B*40, DRB1*15, DRB1*12, and DRB1*04 [29].

The HSCT cohort in our study was heterogeneous, including patients with leukemia, lymphoma, and other hematologic diseases. Because disease-specific subgroup analyses were not performed, this heterogeneity represents a limitation of the present study. The overall allele distribution in HSCT candidates was largely comparable to that observed in donors, suggesting that this cohort reflects the regional HLA background. No disease-specific conclusions can be drawn from the present data. Future studies focusing on more homogeneous diagnostic subgroups may clarify potential allele–disease associations. Previous studies have emphasized the relevance of HLA allele and haplotype diversity in HSCT settings, particularly in relation to regional population characteristics [30–39].

- 1. Haplotype frequency evaluation

In the present study, the most frequent A:B:C haplotype across all groups was 24~35~04. The most frequent A:B:DRB1 haplotype was identical in donors and HSCT candidates (24~35~11) but differed in solid organ transplant candidates (03~35~04). Previously reported A:B:DRB1 haplotypes in hematologic disease studies from Türkiye include the following: in the Black Sea region, 02~35~11 in Hodgkin lymphoma, 02~51~11 in non-Hodgkin lymphoma [36], 03~51~11 in acute myeloblastic leukemia (AML), and 02~35~01 in acute lymphoblastic leukemia (ALL) [32]; in the Aegean region, 24~35~11 in patients and 01~08~03 in controls [33]; and in the Central Anatolia region, 02~35~13 in ALL patients, 01~08~03 in AML patients, and 02~35~04 in controls [30]. The A:B:DRB1 haplotypes associated with ESRD in the present study differed from those reported in China, Romania, and Indonesia [16,19,28], as well as from haplotypes reported in hematologic disease cohorts from Egypt and Mexico [35,39]. In the present study, the dominant A:B:C:DRB1 haplotype was 24~35~04~11 in donors and HSCT candidates, whereas it was 01~08~07~03 in solid organ transplant candidates (Table 2). The dominant five-locus A:B:C:DRB1:DQB1 haplotype was 24~35~04~11~03 in donors and HSCT candidates, whereas 02~35~04~11~03 was observed in solid organ transplant candidates. In the present study, the haplotype 11~35~04~11~03 ranked third among solid organ transplant candidates but fifteenth among donors. Studies from China reported different haplotype patterns [15,17], whereas studies from Türkiye demonstrated partial similarities [33]. The most frequent six-locus A:B:C:DRB1:DQB1:DPB1 haplotypes were 02~35~04~11~03~04 in donors, 24~35~04~11~03~04 in HSCT candidates, and 24~35~04~04~03~04 in solid organ transplant candidates. The most frequent haplotypes in kidney transplant candidates were A:B:C (11~35~04), A:B:DRB1 (01~08~03), and A:B:C:DRB1 (24~35~04~04), which differed from those observed in the other groups.

Recent studies have emphasized the importance of high-resolution HLA typing and population-specific allele distributions in both HSCT and solid organ transplantation settings [40–43].

This study has several limitations that should be acknowledged. Although the sample size represents the largest HLA dataset reported to date in Türkiye, the study population was restricted to the Central Anatolian region, which may limit the generalizability of the findings to the broader national population. Furthermore, although transplant candidates were categorized into HSCT and solid organ groups, detailed subgroup analyses—particularly within the HSCT cohort—could not be performed because of clinical heterogeneity across diagnoses such as AML, ALL, and MDS. To address this limitation, a follow-up study focusing on more homogeneous HSCT subtypes is planned. Additionally, because the majority of transplant candidates were kidney transplant recipients, meaningful comparisons across other solid organ subgroups, such as liver or corneal transplantation, were limited. Lastly, the use of low-resolution genotyping methods (PCR-SSO and PCR-SSP) may have limited the detection of rare allele subtypes; therefore, future studies employing high-resolution sequencing technologies are warranted to achieve greater allele-level resolution and improved haplotype accuracy.

In conclusion, allele frequencies at six HLA loci (HLA-A, HLA-B, HLA-C, HLA-DRB1, HLA-DQB1, and HLA-DPB1), as well as haplotype frequencies across three-, four-, five-, and six-locus combinations and genotype frequencies, were compared between healthy donors from our region and candidates for HSCT and solid organ transplantation. This study provides a comprehensive comparison that, to our knowledge, has not previously been documented in Türkiye.

In addition, consideration of allele and haplotype distributions during donor selection may contribute to optimizing transplant compatibility and potentially improving long-term transplant outcomes. To advance HLA research, larger and more geographically diverse populations should be included in future investigations. Although the study does not encompass the entire Turkish population, it has the potential to contribute meaningfully to mapping HLA distribution patterns nationwide. These findings may support future research initiatives and enhance the understanding of HLA variation at the national level.

**Acknowledgments**

The authors thank Dr. Ertuğrul Çolak for his assistance with the statistical analysis.

Conflict of interest

The authors declare no conflict of interest. All authors have approved the final version of the manuscript and agree to its publication. The study was conducted in accordance with the principles of the Declaration of Helsinki and was approved by the Eskişehir Osmangazi University Faculty of Medicine Ethics Committee (approval no: 2026–55). The datasets supporting the conclusions of this article are included within the article and its Supplementary Files. Additional datasets are not publicly available due to ethical and privacy considerations. This research received no external funding.

References

1. Robinson J, Barker DJ, Georgiou X, Cooper MA, Flicek P et al. IPD-IMGT/HLA Database. Nucleic Acids Res. 2020; 48(D1): 948-955. https://doi.org/10.1093/nar/gkz950

2. Sureda A, Corbacioglu S, Greco R, Kröger N, Carreras E. The EBMT Handbook: Hematopoietic Cell Transplantation and Cellular Therapies Cham, Switzerland: Springer; 2024. https://doi.org/10.1007/978-3-031-44080-9

3. Uyar FA, Dorak MT, Saruhan-Direskeneli G. Human leukocyte antigen-A, -B and -C alleles and human leukocyte antigen haplotypes in Turkey: relationship to other populations. Tissue Antigens 2004; 64 (2): 180-187. https://doi.org/10.1111/j.1399-0039.2004.00258.x

4. Baştürk B, Kantaroğlu B, Kavuzlu M, Sarıtürk Ç. The most common HLA alleles and anti-HLA antibodies to know for virtual cross-match. Experimental and Clinical Transplantation 2016; 14 (Suppl 3): 53-55. https://doi.org/10.6002/ect.tondtdtd2016.P5

5. Saruhan-Direskeneli G, Uyar FA, Bakar Ş, Eraksoy M. Molecular analysis of HLA-DRB1, -DQA1 and -DQB1 polymorphism in Turkey. Tissue Antigens 2008; 55 (2): 171-174. https://doi.org/10.1034/j.1399-0039.2000.550211.x

6. Mete E. Doku tiplendirme laboratuvarına gönderilmiş olan örneklerde HLA antijenlerinin dağılımının araştırılması (in Turkish). Pamukkale Tıp Dergisi 2021; 14 (3): 742 - 746. https://doi.org/10.31362/patd.943791

7. Yantır E, Gündüz E, Çolak E. HLA alleles, genotype and haplotype analyzes from Central Anatolia region of Turkey. Balkan Medical Journal. 2023; 40 (5): 358-366. https://doi.org/10.4274/balkanmedj.galenos.2023.2023-4-55

8. Sanchez-Mazas A, Vidan-Jeras B, Nunes JM, Fischer G, Little A-M et al. Strategies to work with HLA data in human populations for histocompatibility, clinical transplantation, epidemiology and population genetics: HLA-NET methodological recommendations. International Journal of Immunogenetics 2012; 39 (6): 459-476. https://doi.org/10.1111/j.1744-313X.2012.01113.x

9. Nunes JM, Buhler S, Roessli D, Sanchez-Mazas A: HLA-net 2013 collaboration. The HLA-net GENE[RATE] pipeline for effective HLA data analysis and its application to 145 population samples from Europe and neighbouring areas. Tissue Antigens 2014; 83 (5): 307-323. https://doi.org/10.1111/tan.12356

10. Lancaster AK, Single RM, Solberg OD, Nelson MP, Thomson G. PyPop update—a software pipeline for large-scale multilocus population genomics. Tissue Antigens 2007; 69 (s1): 192-197. https://doi.org/10.1111/j.1399-0039.2006.00769.x

11. Balkan E, Yaşar E, Doğan H. The diagnosis of human leukocyte antigen class I and class II allel in Eastern Anatolia region. Van Medical Journal 2019; 26 (2): 162-166. https://dx.doi.org/10.5505/vtd.2019.42042

12. Pala FS, Tabakçıoğlu K, Algüneş Ç, Kurt Ömürlü İ. Evaluation of Frequencies of HLA-A, B and DR in Thracian Population and Examination of its Relationship with Balkan Populations. Trakya Üniversitesi Tıp Fakültesi Dergisi 2008; 25 (3): 189-195. https://izlik.org/JA78KD99WR

13. Pingel J, Solloch UV, Hofmann JA, Lange V, Ehninger G et al. High-resolution HLA haplotype frequencies of stem cell donors in Germany with foreign parentage: How can they be used to improve unrelated donor searches? Human Immunology 2013; 74 (3): 330-340. https://doi.org/10.1016/j.humimm.2012.10.029

14. Pan Q, Ma X, Chen H, Fan S, Wang X et al. A single center study of protective and susceptible HLA alleles and haplotypes with end-stage renal disease in China. Human Immunology 2019; 80 (11): 943-947. https://doi.org/10.1016/j.humimm.2019.09.001

15. Davood P, Farhadi N, Najafizadeh M. Protective and susceptible HLA class I genes in patients with end-stage renal disease. Research Journal of Biological Sciences 2008; 3 (11): 1344-1346. https://doi.org/

16. Pei Y, Li H, Huang C, Qin Y, Sun X. Associations between end stage renal disease and HLA polymorphisms in the Guangxi Zhuang population. Scientific Reports 2024; 14 (1): 21765. https://doi.org/10.1038/s41598-024-72688-2

17. Yamakawa RH, Saito PK, da Silva Junior WV, de Mattos LC, Borelli SD. Polymorphism of Leukocyte and Erythrocyte Antigens in Chronic Kidney Disease Patients in Southern Brazil. Plos One 2014; 9 (1): e84456. https://doi.org/10.1371/journal.pone.0084456

18. Rivera P S, Márquez G, Cipriani AM, Hassanhi M, Villalobos CC et al. HLA class I association with progression to end-stage renal disease in patients from Zulia, Venezuela. Inmunología 2012; 31 (2): 37-42. https://doi.org/10.1016/j.inmuno.2011.12.001

19. Cao Q, Xie D, Liu J, Zou H, Zhang Y et al. HLA polymorphism and susceptibility to end-stage renal disease in Cantonese patients awaiting kidney transplantation. Plos One 2014; 9 (6): e90869. https://doi.org/10.1371/journal.pone.0090869

20. Hernández-Rivera JCH, Salazar-Mendoza M, Pérez-López MJ, González-Ramos J, Espinoza-Pérez R et al. Most common HLA alleles associated with risk and/or protection in chronic kidney disease of undetermined etiology (in Spanish). Gaceta Médica de México 2019; 155 (3): 243-248. https://doi.org/10.24875/gmm.19005033

21. Crispim JC, Mendes-Júnior CT, Wastowski IJ, Palomino GM, Saber LT et al. HLA Polymorphisms as Incidence Factor in the Progression to End-Stage Renal Disease in Brazilian Patients Awaiting Kidney Transplant. Transplantation Proceedings 2008; 40 (5): 1333-1336. https://doi.org/10.1016/j.transproceed.2008.02.086

22. Noureen N, Shah FA, Lisec J, Usman H, Khalid M et al. Revisiting the association between human leukocyte antigen and end-stage renal disease. Plos One; 15 (9): e0238878. https://doi.org/10.1371/journal.pone.0238878

23. Shi X, Han W, Ding J. The impact of human leukocyte antigen mismatching on graft survival and mortality in adult renal transplantation: A protocol for a systematic review and meta-analysis. Medicine (Baltimore) 2017; 96 (49): e8899. https://doi.org/10.1097/md.0000000000008899

24. Shao LN, Yang Y, Zhang ST, Zhou SH, Duan Y et al. Association between the polymorphism of HLA and ESRD in Dalian Han population located in north of China. Immunological Investigations 2018; 47 (2): 212-219. https://doi.org/10.1080/08820139.2017.1416397

25. Lowe M, Jervis S, Payton A, Poulton K, Worthington J et al. Systematic review of associations between HLA and renal function. International Journal of Immunogenetics 2022; 49 (1): 46-62. https://doi.org/10.1111/iji.12566

26. Chang DY, Luo H, Zhou XJ, Chen M, Zhao MH. Association of HLA genes with clinical outcomes of ANCA-associated vasculitis. Clinical Journal of the American Society of Nephrology 2012; 7 (8): 1293-1299. https://doi.org/10.2215/cjn.13071211

27. Susianti H, Djatmiko DP, Adi Widana IK, Tandio DA, Sutrisnani CS et al. Evaluation of Human Leukocyte Antigen Class I and Class II in End-Stage Renal Disease Occurrence in Indonesian Transplantation Patients. International Journal of Nephrology 2021; 4219822, 6 pages. https://doi.org/10.1155/2021/4219822

28. Iancu Loga LI, Dican L, Chiorean AD, Chelaru VF, Elec FI et al. Association between Human Leukocyte Antigen and End-Stage Renal Disease in Patients from Transylvania, Romania. International Journal of Molecular Sciences 2023; 24 (17): 13383. https://doi.org/10.3390/ijms241713383

29. Tuladhar A, Shrestha S, Raut PP, Bhandari P, Shrestha P. HLA antigen distribution in renal transplant recipients and donors. Journal of Nepal Health Research Council 2013; 11 (25): 289-292. https://elibrary.nhrc.gov.np/bitstream/20.500.14356/1877/1/407-Article%20Text-490-1-10-20140207.pdf

30. Patiroglu T, Akar HH. Relationships of Human Leukocyte Antigen-A, -B, -DRB1 Alleles, and Haplotypes in 129 Ethnic Turkish Patients with Acute Myeloblastic Leukemia. Laboratory Medicine 2015; 46 (3): 195-199. https://doi.org/10.1309/lml8dsrktfuo27rm

31. Patıroğlu T, Akar HH. The Frequency of HLA-A, HLA-B, and HLA-DRB1 Alleles in Patients with Acute Lymphoblastic Leukemia in the Turkish Population: A Case-Control Study. Turkish Journal of Hematology 2016; 33 (4): 339-345. https://doi.org/10.4274/tjh.2016.0102

32. Uçar F, Sönmez M, Erkut N, Balcı M, Yücel B et al. Relation of HLA-A, -B, -DRB1 Alleles and Haplotypes in Patients with Acute Leukemia: A Case Control Study. Archives of Medical Research 2011; 42 (4): 305-310. https://doi.org/10.1016/j.arcmed.2011.06.003

33. Güleç RD, Arslan FD. Frequencies of HLA Alleles in Patients with Acute Lymphoblastic and Myeloid Leukemia. Medical Science and Discovery 2023; 10 (8): 539-545. https://doi.org/10.36472/msd.v10i8.994

34. ElNahass Y, Mekky N, Abdelfattah NM, Abdelfattah R, Samra M et al. HLA alleles, haplotypes frequencies, and their association with hematological disorders: a report from 1550 families whose patients underwent allogeneic bone marrow transplantation in Egypt. Immunogenetics 2024; 76 (4): 243-60. https://doi.org/10.1007/s00251-024-01343-x

35. Li YM, Li YX, Li DY, Zhou Y, An L et al. Investigation of HLA susceptibility alleles and genotypes with hematological disease among Chinese Han population. Plos One 2024; 19 (4): e0281698. https://doi.org/10.1371/journal.pone.0281698

36. Uçar F, Sönmez M, Ermantaş N, Özbaş HM, Cansız A et al. The associations of HLA-A, -B, DRB1 alleles and haplotypes in Turkish lymphoma patients. Gene 2016; 586 (2): 263-267. https://doi.org/10.1016/j.gene.2016.04.017

37. Sayad A, Akbari MT, Mehdizadeh M, Movafagh A, Hajifathali A. The association of HLA-class I and class II with Hodgkin's lymphoma in Iranian patients. BioMed Research International 2014; 231236, 5 pages. https://doi.org/10.1155/2014/231236

38. Nathalang O, Tatsumi N, Hino M, Prayoonwiwat W, Yamane T et al. HLA class II polymorphism in Thai patients with non-Hodgkin's lymphoma. European Journal of Immunogenetics 1999; 26 (6): 389-392. https://doi.org/10.1046/j.1365-2370.1999.00177.x

39. Bello–López JM, Cisneros CB, Martínez-Albarrán A. HLA analysis of Mexican candidates for bone marrow transplantation and probability of finding compatible related donors. Transfusion and Apheresis Science 2018; 57 (1): 82-87. https://doi.org/10.1016/j.transci.2017.12.004

40. Bezstarosti S, Heidt S. The Progress and Challenges of Implementing HLA Molecular Matching in Clinical Practice. Transpl. Int. 2025; 38:14716. https://doi.org/10.3389/ti.2025.14716

41. Abraham G, Kute V, Prasad. N, Daniel D, Tapiawala S. Immunological Challenges in Organ Transplantation. Singapore: Springer; 2025. https://doi.org/10.1007/978-981-95-1533-2

42. Lachmann N, Pruß A. HLA in Transplantation: Challenges and Perspectives. Transfus Med Hemother. 2024 Jun 3;51(3):129-130. https://doi.org/10.1159/000538982

43. An L, Affdal A, Ballesteros F, Malo MF, Cochran-Mavrikakis SL et al. HLA Experts' Perspectives on Precision Medicine and Molecular Matching in Kidney Transplantation: A Qualitative Study. Can J Kidney Health Dis. 2026; 13: 20543581251412195. https://doi.org/10.1177/20543581251412195


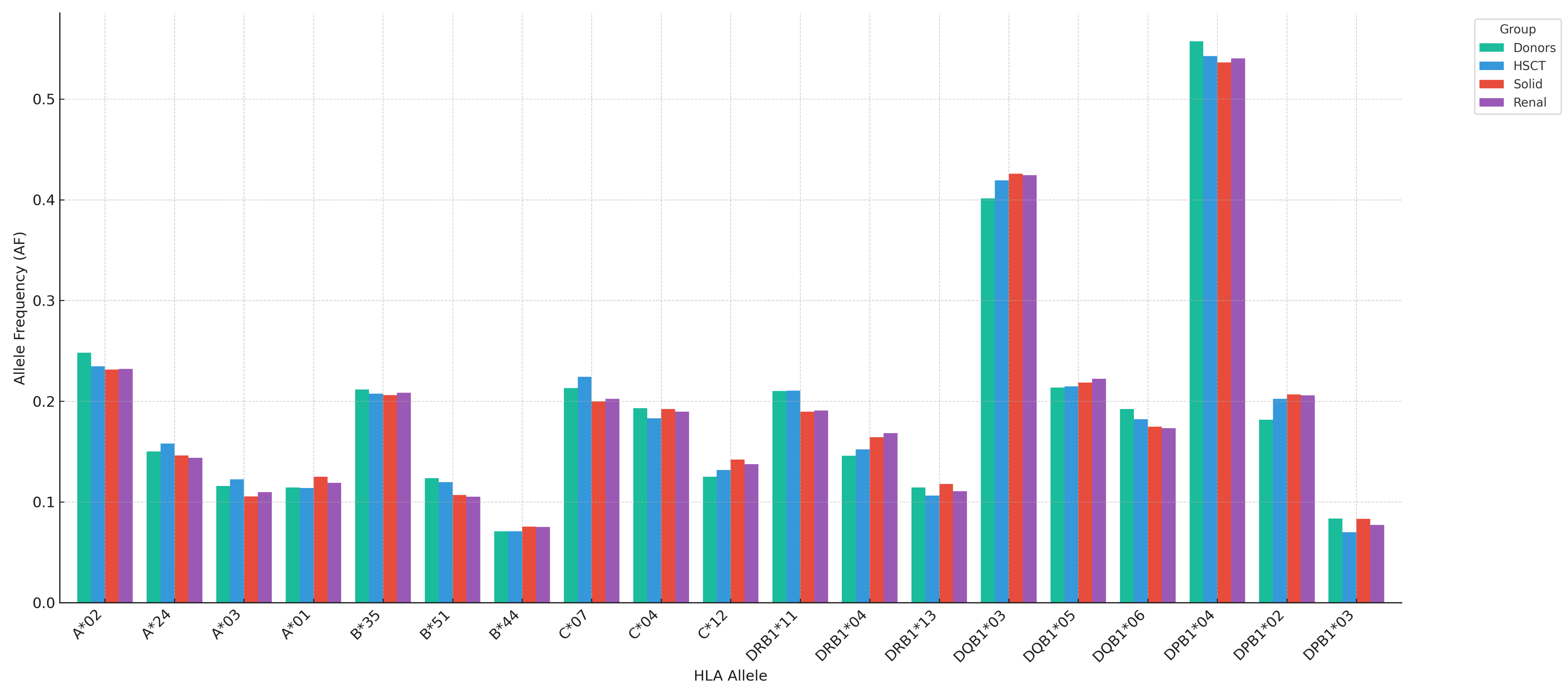


**Figure 1.** Allele frequencies across all study groups. The bar chart displays allele frequency (AF) values for each HLA allele, with study groups distinguished by different colors. Overall, distribution patterns are comparable across groups, although slight frequency differences are observed for certain alleles. The x-axis lists individual HLA alleles, whereas the y-axis represents the corresponding allele frequencies.


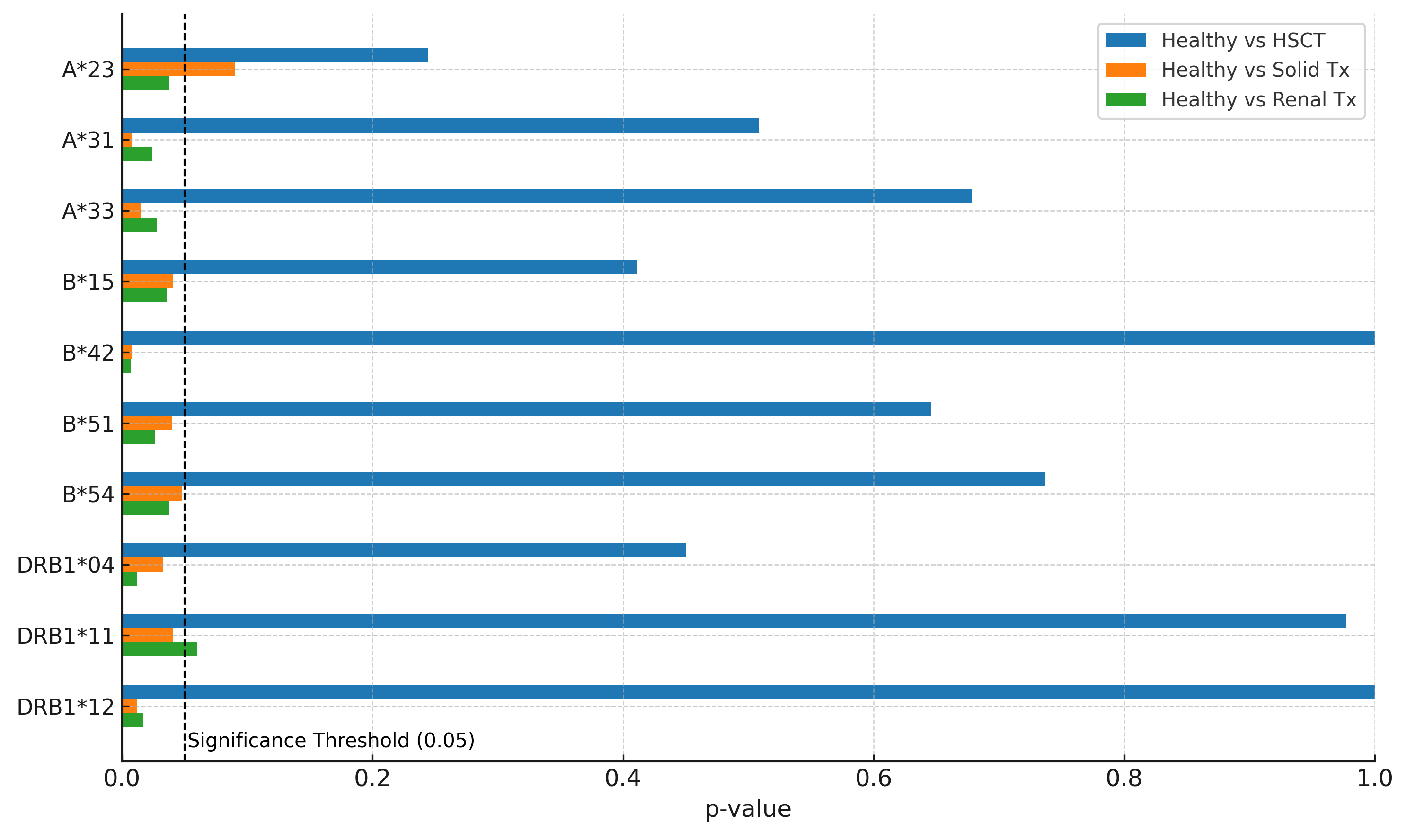


**Figure 2.** Comparison of p values for statistically significant differences in HLA alleles between groups. Horizontal bar chart illustrating alleles with statistically significant differences (p < 0.05) between healthy donors and transplant candidate groups: hematopoietic stem cell transplantation (HSCT), solid organ transplantation, and kidney transplantation. Each group is represented by a distinct color. Alleles are listed on the y-axis, and the corresponding p values are displayed on the x-axis. The black dashed vertical line indicates the statistical significance threshold (p = 0.05).


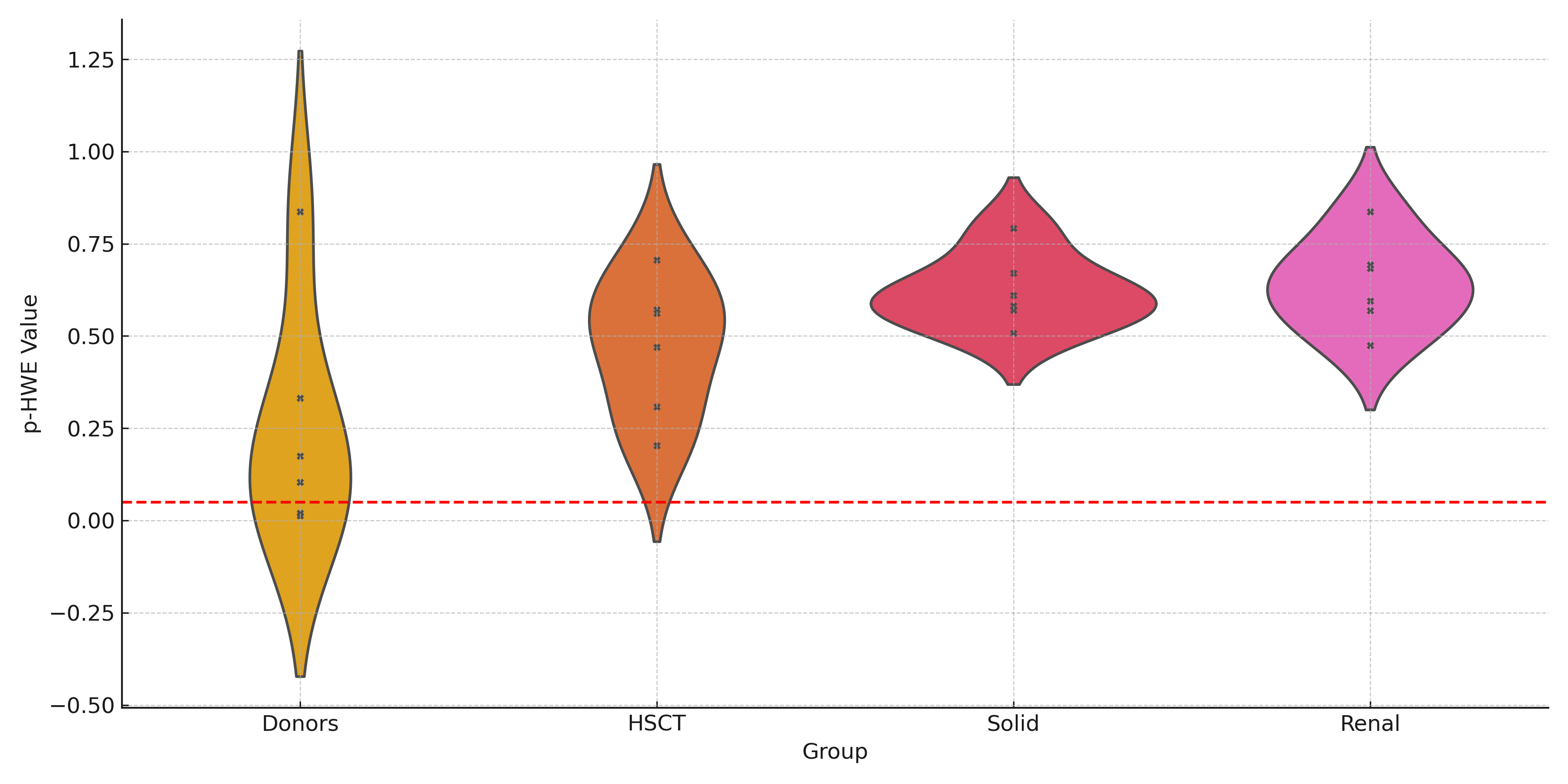


**Figure 3.** Distribution of Hardy–Weinberg equilibrium (HWE) p values across study groups. The distribution of p values for HWE testing across donors, HSCT candidates, solid organ transplant candidates, and renal transplant candidates is presented. The width of each violin reflects the density of data points across p value levels. The dashed horizontal line represents the statistical significance threshold (p = 0.05). Alleles in the donor group exhibit broader dispersion, with several p values below the significance threshold, whereas transplant candidate groups generally display higher HWE p values, suggesting less deviation from equilibrium.


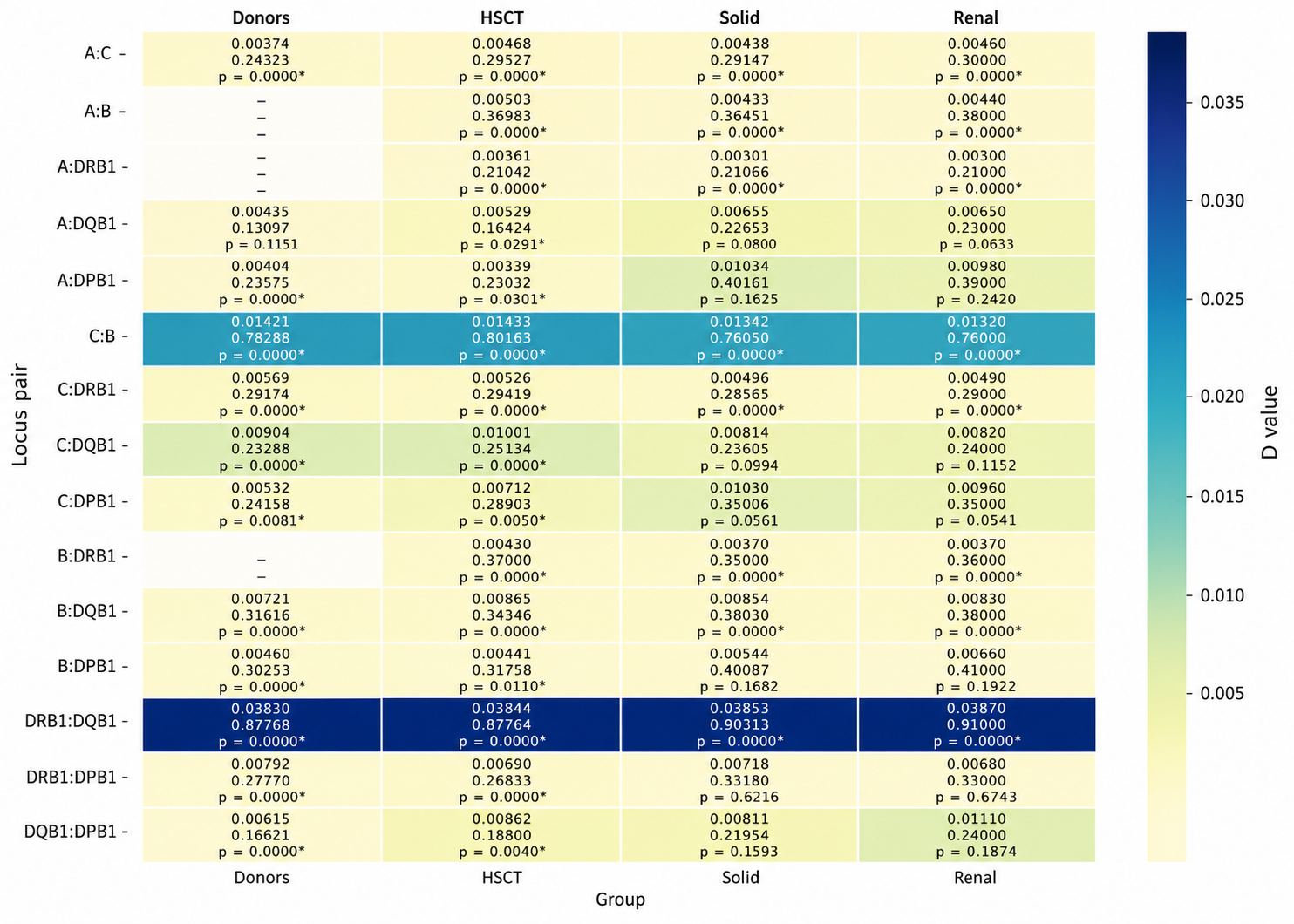


**Figure 4.** Comparative heat map of pairwise linkage disequilibrium (LD) values across study groups. The heat map summarizes pairwise LD parameters (D, D', and p values) for selected HLA locus pairs across four groups: donors, HSCT candidates, solid organ transplant candidates, and kidney transplant candidates. Each cell displays LD statistics (D and D') along with the corresponding p value. Darker colors indicate higher D values and stronger LD between locus pairs. Statistically significant p values (p < 0.05) indicate nonrandom associations between specific HLA loci. Missing data are left blank because the Pypop software cannot evaluate datasets exceeding 5000 haplotypes; consequently, no estimates were obtained for the A:B, B:DRB1, and A:DRB1 haplotypes.

**Table 1.** Most frequent allele frequencies at the HLA-A, HLA-B, HLA-C, HLA-DRB1, HLA-DQB1, and HLA-DPB1 loci across study groups.

| **Donors** | | | **HSCT candidates** | | | **Solid tx candidates** | | | **Renal tx candidates** | | |
| --- | --- | --- | --- | --- | --- | --- | --- | --- | --- | --- | --- |
| **Allele** | **AF** | **2n** | **Allele** | **AF** | **2n** | **Allele** | **AF** | **2n** | **Allele** | **AF** | **2n** |
| **A*02** | 0.24821 | 12,944 | **A*02** | 0.23475 | 2164 | **A*02** | 0.23140 | 1936 | **A*02** | 0.23191 | 1824 |
| **A*24** | 0.15019 | 12,944 | **A*24** | 0.15804 | 2164 | **A*24** | 0.14618 | 1936 | **A*24** | 0.14364 | 1824 |
| **A*03** | 0.11570 | 12,944 | **A*03** | 0.12246 | 2164 | ***A*01*** | *0.12500* | *1936* | **A*01** | 0.11897 | 1824 |
| ***A*01*** | 0.11437 | 12,944 | ***A*01*** | 0.11368 | 2164 | **A*03** | 0.10537 | 1936 | **A*03** | 0.10965 | 1824 |
| **B*35** | 0.21154 | 12,944 | **B*35** | 0.20749 | 2164 | **B*35** | 0.20610 | 1936 | **B*35** | 0.20833 | 1824 |
| **B*51** | 0.12348 | 12,944 | **B*51** | 0.11969 | 2164 | **B*51** | 0.10692 | 1936 | **B*51** | 0.10526 | 1824 |
| **B*44** | 0.07069 | 12,944 | **B*44** | 0.07070 | 2164 | **B*44** | 0.07541 | 1936 | **B*44** | 0.07511 | 1824 |
| **C*07** | 0.21314 | 3852 | **C*07** | 0.22425 | 1262 | **C*07** | 0.19981 | 1056 | **C*07** | 0.20236 | 1018 |
| **C*04** | 0.19315 | 3852 | **C*04** | 0.18304 | 1262 | **C*04** | 0.19223 | 1056 | **C*04** | 0.18959 | 1018 |
| **C*12** | 0.12513 | 3852 | **C*12** | 0.13154 | 1262 | **C*12** | 0.14205 | 1056 | **C*12** | 0.13752 | 1018 |
| **DRB1*11** | 0.20998 | 12,944 | **DRB1*11** | 0.21026 | 2164 | **DRB1*11** | 0.18957 | 1936 | **DRB1*11** | 0.19079 | 1824 |
| **DRB1*04** | 0.14575 | 12,944 | **DRB1*04** | 0.15203 | 2164 | **DRB1*04** | 0.16426 | 1936 | **DRB1*04** | 0.16831 | 1824 |
| **DRB1*13** | 0.11437 | 12,944 | **DRB1*13** | 0.10628 | 2164 | **DRB1*13** | 0.11777 | 1936 | **DRB1*13** | 0.11075 | 1824 |
| **DQB1*03** | 0.40143 | 1400 | **DQB1*03** | 0.41930 | 1202 | **DQB1*03** | 0.42576 | 458 | **DQB1*03** | 0.42444 | 450 |
| **DQB1*05** | 0.21357 | 1400 | **DQB1*05** | 0.21464 | 1202 | **DQB1*05** | 0.21834 | 458 | **DQB1*05** | 0.22222 | 450 |
| **DQB1*06** | 0.19214 | 1400 | **DQB1*06** | 0.18220 | 1202 | **DQB1*06** | 0.17467 | 458 | **DQB1*06** | 0.17333 | 450 |
| **DPB1*04** | 0.55743 | 1184 | **DPB1*04** | 0.54251 | 988 | **DPB1*04** | 0.53623 | 276 | **DPB1*04** | 0.54044 | 272 |
| **DPB1*02** | 0.18159 | 1184 | **DPB1*02** | 0.20243 | 988 | **DPB1*02** | 0.20652 | 276 | **DPB1*02** | 0.20588 | 272 |
| **DPB1*03** | 0.08361 | 1184 | **DPB1*03** | 0.06984 | 988 | **DPB1*03** | 0.08333 | 276 | **DPB1*03** | 0.07721 | 272 |

Abbreviations: AF, allele frequency; 2n, allele count; Tx, transplantation; HSCT, hematopoietic stem cell transplantation.

**Table 2**. Most frequent haplotypes across study groups.

| **Donors** | | **HSCT** | | **Solid Tx candidates** | | **Renal Tx candidates** | |
| --- | --- | --- | --- | --- | --- | --- | --- |
| **Haplotype name** | **HF** | **Haplotype name** | **HF** | **Haplotype name** | **HF** | **Haplotype name** | **HF** |
| **A:B:C** | | | | | | | |
| **24~35~04** | **0.03432** | **24~35~04** | **0.04436** | **24~35~04** | **0.03558** | **11~35~04** | **0.03482** |
| 02~35~04 | 0.03158 | 03~35~04 | 0.03037 | 11~35~04 | 0.03373 | 02~35~04 | 0.03228 |
| 03~35~04 | 0.02599 | 23~49~07 | 0.02489 | 02~35~04 | 0.02863 | 24~35~04 | 0.03107 |
| **A:B.DR** | | | | | | | |
| **24~35~11** | **0.0163** | **24~35~11** | **0.01979** | **03~35~04** | **0.01084** | **01~08~03** | **0.01747** |
| 02~51~11 | 0.0135 | 03~35~11 | 0.01383 | 24~07~12 | 0.00101 | 02~51~04 | 0.01487 |
| 02~51~04 | 0.0116 | 02~51~11 | 0.01231 | 02~55~11 | 0.00101 | 03~35~04 | 0.01332 |
| **DRB1:DQB1:DPB1** | | | | | | | |
| **11~03~04** | **0.14238** | **11~03~04** | **0.13522** | **11~03~04** | **0.12284** | **11~03~04** | **0.12404** |
| 15~06~04 | 0.05765 | 04~03~04 | 0.06252 | 04~03~04 | 0.07992 | 04~03~04 | 0.08201 |
| 13~06~04 | 0.05546 | 15~06~04 | 0.05457 | 03~02~04 | 0.06390 | 03~02~04 | 0.06529 |
| **A:B:C:DRB1** | | | | | | |  |
| **24~35~04~11** | **0.0155** | **24~35~04~11** | **0.01888** | **01~08~07~03** | **0.01678** | **24~35~04~04** | **0.01681** |
| 02~35~04~11 | 0.01278 | 23~49~07~11 | 0.01471 | 24~35~04~11 | 0.01548 | 01~08~07~03 | 0.01656 |
| 23~49~07~11 | 0.01154 | 03~35~04~11 | 0.01373 | 24~35~04~04 | 0.01406 | 02~35~04~11 | 0.01573 |
| **A:B:C:DRB1:DQB1** | | | | | | | |
| **24~35~04~11~03** | **0.0251** | **24~35~04~11~03** | **0.03138** | **02~35~04~11~03** | **0.02374** | **02~35~04~11~03** | **0.02928** |
| 02~35~04~11~03 | 0.0232 | 23~49~07~11~03 | 0.01385 | 24~35~04~04~03 | 0.01991 | 24~35~04~04~03 | 0.01958 |
| 02~35~04~04~03 | 0.01514 | 02~13~06~07~02 | 0.01316 | 01~08~07~03~02 | 0.0177 | 01~08~07~03~02 | 0.01577 |
| **A:B:C:DRB1:DQB1:DPB1** | | | | | | | |
| **02~35~04~11~03~04** | **0.02693** | **24~35~04~11~03~04** | **0.01911** | **24~35~04~04~03~04** | **0.02612** | **24~35~04~04~03~04** | **0.02652** |
| 24~35~04~11~03~04 | 0.01430 | 02~35~04~04~03~02 | 0.01418 | 11~35~04~11~03~04 | 0.01866 | 11~35~04~11~03~04 | 0.01894 |
| 11~52~12~15~06~04 | 0.01331 | 23~49~07~11~03~04 | 0.01280 | 24~13~06~07~02~04 | 0.01493 | 01~08~07~03~02~04 | 0.01515 |

Abbreviations: HF, haplotype frequency; Tx, transplantation; HSCT, hematopoietic stem cell transplantation.

**Table S1.** The allele frequency for HLA-A, -B, -C, -DRB1, -DQB1 and -DPB1 in donors.

**Table S2.** The allele frequency for HLA-A, -B, -C, -DRB1, -DQB1 and -DPB1 in HSCT candidates.

**Table S3.** The allele frequency for HLA-A, -B, -C, -DRB1, -DQB1 and -DPB1 in solid organ transplantation candidates.

**Table S4.** The allele frequency for HLA-A, -B, -C, -DRB1, -DQB1 and -DPB1 in renal transplantation candidates.

**Table S5.** p-values (Comparison of HLA alleles in HSCT, solid organ, and renal transplantation candidates with donors).

**Table S6.** Slatkin’s implementation of Ewens-Watterson homozygosity test of neutrality.

**Table S7.** Hardy-Weinberg Equilibrium analysis of HLA loci in donors. HSCT candidates. and solid organ transplantation candidates.

**Table S8.** Heterozygotes of the most common HLA alleles.

**Table S9.** Most frequently HLA genotypes.

**Table S10.** Pairwise LD estimates.

**Table S11.** Allele ranking for HLA-A loci.

**Table S12.** Allele ranking for HLA-B loci.

**Table S13.** Allele ranking for HLA-C loci.

**Table S14.** Allele ranking for HLA-DRB1 loci.

**Table S15.** Allele ranking for HLA-DQB1 loci.

**Table S16.** Allele ranking for HLA-DPB1 loci.

**Table S1.** The allele frequency for HLA-A, -B, -C, -DRB1, -DQB1 and -DPB1 in donors.

|  | **AF** | **2 n** |  | **AF** | **2 n** |  | **AF** | **2 n** |  | **AF** | **2 n** |  | **AF** | **2n** | **AF** | **2n** |  |
| --- | --- | --- | --- | --- | --- | --- | --- | --- | --- | --- | --- | --- | --- | --- | --- | --- | --- |
| **A*01** | 0.11437 | 12944 | **B*03** | 0.00016 | 12944 | **C*01** | 0.0392 | 3852 | **DRB1*01** | 0.07062 | 12944 | **DQB1*02** | 0.16571 | 1400 | **DPB1*01** | 0.01267 | 1184 |
| **A*02** | 0.24821 | 12944 | **B*04** | 0.00008 | 12944 | **C*02** | 0.03453 | 3852 | **DRB1*02** | 0.00008 | 12944 | **DQB1*03** | 0.40143 | 1400 | **DPB1*02** | 0.18159 | 1184 |
| **A*03** | 0.11570 | 12944 | **B*07** | 0.05154 | 12944 | **C*03** | 0.05633 | 3852 | **DRB1*03** | 0.08175 | 12944 | **DQB1*04** | 0.02714 | 1400 | **DPB1*03** | 0.08361 | 1184 |
| **A*11** | 0.07607 | 12944 | **B*08** | 0.04025 | 12944 | **C*04** | 0.19315 | 3852 | **DRB1*04** | 0.14575 | 12944 | **DQB1*05** | 0.21357 | 1400 | **DPB1*04** | 0.55743 | 1184 |
| **A*22** | 0.00008 | 12944 | **B*13** | 0.03511 | 12944 | **C*05** | 0.02466 | 3852 | **DRB1*07** | 0.09335 | 12944 | **DQB1*06** | 0.19214 | 1400 | **DPB1*05** | 0.01774 | 1184 |
| **A*23** | 0.03262 | 12944 | **B*14** | 0.02227 | 12944 | **C*06** | 0.10462 | 3852 | **DRB1*08** | 0.02359 | 12944 |  |  |  | **DPB1*06** | 0.00253 | 1184 |
| **A*24** | 0.15019 | 12944 | **B*15** | 0.03441 | 12944 | **C*07** | 0.21314 | 3852 | **DRB1*09** | 0.01230 | 12944 |  |  |  | **DPB1*08** | 0.00084 | 1184 |
| **A*25** | 0.01028 | 12944 | **B*18** | 0.05808 | 12944 | **C*08** | 0.03686 | 3852 | **DRB1*10** | 0.02273 | 12944 |  |  |  | **DPB1*09** | 0.01520 | 1184 |
| **A*26** | 0.05793 | 12944 | **B*19** | 0.00008 | 12944 | **C*10** | 0.00026 | 3852 | **DRB1*11** | 0.20998 | 12944 |  |  |  | **DPB1*10** | 0.01605 | 1184 |
| **A*29** | 0.02009 | 12944 | **B*24** | 0.00008 | 12944 | **C*12** | 0.12513 | 3852 | **DRB1*12** | 0.01510 | 12944 |  |  |  | **DPB1*104** | 0.00338 | 1184 |
| **A*30** | 0.03434 | 12944 | **B*27** | 0.03169 | 12944 | **C*13** | 0.00026 | 3852 | **DRB1*13** | 0.11437 | 12944 |  |  |  | **DPB1*105** | 0.00422 | 1184 |
| **A*31** | 0.01565 | 12944 | **B*32** | 0.00016 | 12944 | **C*14** | 0.04076 | 3852 | **DRB1*14** | 0.06330 | 12944 |  |  |  | **DPB1*11** | 0.00338 | 1184 |
| **A*32** | 0.04617 | 12944 | **B*34** | 0.00008 | 12944 | **C*15** | 0.06906 | 3852 | **DRB1*15** | 0.09779 | 12944 |  |  |  | **DPB1*126** | 0.00084 | 1184 |
| **A*33** | 0.02881 | 12944 | **B*35** | 0.21154 | 12944 | **C*16** | 0.03998 | 3852 | **DRB1*16** | 0.04928 | 12944 |  |  |  | **DPB1*129** | 0.00084 | 1184 |
| **A*34** | 0.00016 | 12944 | **B*37** | 0.01409 | 12944 | **C*17** | 0.01765 | 3852 |  |  |  |  |  |  | **DPB1*13** | 0.02111 | 1184 |
| **A*36** | 0.00039 | 12944 | **B*38** | 0.04173 | 12944 | **C*18** | 0.00441 | 3852 |  |  |  |  |  |  | **DPB1*131** | 0.00169 | 1184 |
| **A*38** | 0.00008 | 12944 | **B*39** | 0.01581 | 12944 |  |  |  |  |  |  |  |  |  | **DPB1*14** | 0.01436 | 1184 |
| **A*66** | 0.00498 | 12944 | **B*40** | 0.03722 | 12944 |  |  |  |  |  |  |  |  |  | **DPB1*15** | 0.01182 | 1184 |
| **A*68** | 0.03722 | 12944 | **B*41** | 0.02375 | 12944 |  |  |  |  |  |  |  |  |  | **DPB1*16** | 0.00084 | 1184 |
| **A*69** | 0.00646 | 12944 | **B*42** | 0.00008 | 12944 |  |  |  |  |  |  |  |  |  | **DPB1*17** | 0.02280 | 1184 |
| **A*74** | 0.00023 | 12944 | **B*44** | 0.07069 | 12944 |  |  |  |  |  |  |  |  |  | **DPB1*18** | 0.00169 | 1184 |
|  |  |  | **B*45** | 0.00273 | 12944 |  |  |  |  |  |  |  |  |  | **DPB1*189** | 0.00084 | 1184 |
|  |  |  | **B*46** | 0.00093 | 12944 |  |  |  |  |  |  |  |  |  | **DPB1*19** | 0.00338 | 1184 |
|  |  |  | **B*47** | 0.00109 | 12944 |  |  |  |  |  |  |  |  |  | **DPB1*22** | 0.00084 | 1184 |
|  |  |  | **B*48** | 0.00483 | 12944 |  |  |  |  |  |  |  |  |  | **DPB1*23** | 0.00507 | 1184 |
|  |  |  | **B*49** | 0.03877 | 12944 |  |  |  |  |  |  |  |  |  | **DPB1*26** | 0.00422 | 1184 |
|  |  |  | **B*50** | 0.03379 | 12944 |  |  |  |  |  |  |  |  |  | **DPB1*36** | 0.00084 | 1184 |
|  |  |  | **B*51** | 0.12348 | 12944 |  |  |  |  |  |  |  |  |  | **DPB1*45** | 0.00084 | 1184 |
|  |  |  | **B*52** | 0.02974 | 12944 |  |  |  |  |  |  |  |  |  | **DPB1*47** | 0.00169 | 1184 |
|  |  |  | **B*53** | 0.00397 | 12944 |  |  |  |  |  |  |  |  |  | **DPB1*503** | 0.00084 | 1184 |
|  |  |  | **B*54** | 0.00117 | 12944 |  |  |  |  |  |  |  |  |  | **DPB1*51** | 0.00169 | 1184 |
|  |  |  | **B*55** | 0.03192 | 12944 |  |  |  |  |  |  |  |  |  | **DPB1*61** | 0.00084 | 1184 |
|  |  |  | **B*56** | 0.00343 | 12944 |  |  |  |  |  |  |  |  |  | **DPB1*66** | 0.00169 | 1184 |
|  |  |  | **B*57** | 0.01806 | 12944 |  |  |  |  |  |  |  |  |  | **DPB1*70** | 0.00084 | 1184 |
|  |  |  | **B*58** | 0.01573 | 12944 |  |  |  |  |  |  |  |  |  | **DPB1*84** | 0.00084 | 1184 |
|  |  |  | **B*59** | 0.00031 | 12944 |  |  |  |  |  |  |  |  |  | **DPB1*91** | 0.00084 | 1184 |
|  |  |  | **B*60** | 0.00008 | 12944 |  |  |  |  |  |  |  |  |  |  |  |  |
|  |  |  | **B*66** | 0.00008 | 12944 |  |  |  |  |  |  |  |  |  |  |  |  |
|  |  |  | **B*73** | 0.00078 | 12944 |  |  |  |  |  |  |  |  |  |  |  |  |
|  |  |  | **B*82** | 0.00008 | 12944 |  |  |  |  |  |  |  |  |  |  |  |  |
|  |  |  | **B*83** | 0.00016 | 12944 |  |  |  |  |  |  |  |  |  |  |  |  |

Abbreviations: AF, allele frequency; 2n, allele count

**Table S2.** The allele frequency for HLA-A, -B, -C, -DRB1, -DQB1 and -DPB1 in HSCT candidates.

|  | **AF** | **2n** |  | **AF** | **2n** |  | **AF** | **2n** |  | **AF** | **2n** |  | **AF** | **2n** |  | **AF** | **2n** |
| --- | --- | --- | --- | --- | --- | --- | --- | --- | --- | --- | --- | --- | --- | --- | --- | --- | --- |
| **A*01** | 0.11368 | 2164 | **B*07** | 0.05684 | 2164 | **C*01** | 0.04437 | 1262 | **DRB1*01** | 0.06932 | 2164 | **DQB1*02** | 0.15474 | 1202 | **DPB1*01** | 0.01518 | 988 |
| **A*02** | 0.23475 | 2164 | **B*08** | 0.03512 | 2164 | **C*02** | 0.04041 | 1262 | **DRB1*03** | 0.07994 | 2164 | **DQB1*03** | 0.41930 | 1202 | **DPB1*02** | 0.20243 | 988 |
| **A*03** | 0.12246 | 2164 | **B*13** | 0.03420 | 2164 | **C*03** | 0.05388 | 1262 | **DRB1*04** | 0.15203 | 2164 | **DQB1*04** | 0.02912 | 1202 | **DPB1*03** | 0.06984 | 988 |
| **A*11** | 0.07763 | 2164 | **B*14** | 0.02126 | 2164 | **C*04** | 0.18304 | 1262 | **DRB1*07** | 0.08919 | 2164 | **DQB1*05** | 0.21464 | 1202 | **DPB1*04** | 0.54251 | 988 |
| **A*23** | 0.03743 | 2164 | **B*15** | 0.03789 | 2164 | **C*05** | 0.02536 | 1262 | **DRB1*08** | 0.02865 | 2164 | **DQB1*06** | 0.18220 | 1202 | **DPB1*05** | 0.01518 | 988 |
| **A*24** | 0.15804 | 2164 | **B*18** | 0.05823 | 2164 | **C*06** | 0.09667 | 1262 | **DRB1*09** | 0.01017 | 2164 |  |  |  | **DPB1*06** | 0.00405 | 988 |
| **A*25** | 0.01340 | 2164 | **B*27** | 0.02773 | 2164 | **C*07** | 0.22425 | 1262 | **DRB1*10** | 0.02172 | 2164 |  |  |  | **DPB1*08** | 0.00202 | 988 |
| **A*26** | 0.05961 | 2164 | **B*35** | 0.20749 | 2164 | **C*08** | 0.03566 | 1262 | **DRB1*11** | 0.21026 | 2164 |  |  |  | **DPB1*09** | 0.01923 | 988 |
| **A*29** | 0.02264 | 2164 | **B*37** | 0.01294 | 2164 | **C*12** | 0.13154 | 1262 | **DRB1*12** | 0.01479 | 2164 |  |  |  | **DPB1*10** | 0.01417 | 988 |
| **A*30** | 0.03142 | 2164 | **B*38** | 0.04113 | 2164 | **C*14** | 0.03724 | 1262 | **DRB1*13** | 0.10628 | 2164 |  |  |  | **DPB1*104** | 0.00709 | 988 |
| **A*31** | 0.01340 | 2164 | **B*39** | 0.01617 | 2164 | **C*15** | 0.07132 | 1262 | **DRB1*14** | 0.06562 | 2164 |  |  |  | **DPB1*105** | 0.00607 | 988 |
| **A*32** | 0.03835 | 2164 | **B*40** | 0.03604 | 2164 | **C*16** | 0.03407 | 1262 | **DRB1*15** | 0.10120 | 2164 |  |  |  | **DPB1*11** | 0.00202 | 988 |
| **A*33** | 0.03050 | 2164 | **B*41** | 0.02542 | 2164 | **C*17** | 0.01902 | 1262 | **DRB1*16** | 0.05083 | 2164 |  |  |  | **DPB1*124** | 0.00101 | 988 |
| **A*50** | 0.00046 | 2164 | **B*44** | 0.07070 | 2164 | **C*18** | 0.00317 | 1262 |  |  |  |  |  |  | **DPB1*126** | 0.00101 | 988 |
| **A*66** | 0.00508 | 2164 | **B*45** | 0.00139 | 2164 |  |  |  |  |  |  |  |  |  | **DPB1*129** | 0.00101 | 988 |
| **A*68** | 0.03558 | 2164 | **B*46** | 0.00092 | 2164 |  |  |  |  |  |  |  |  |  | **DPB1*13** | 0.01518 | 988 |
| **A*69** | 0.00555 | 2164 | **B*47** | 0.00092 | 2164 |  |  |  |  |  |  |  |  |  | **DPB1*14** | 0.01721 | 988 |
|  |  |  | **B*48** | 0.00508 | 2164 |  |  |  |  |  |  |  |  |  | **DPB1*15** | 0.00911 | 988 |
|  |  |  | **B*49** | 0.0476 | 2164 |  |  |  |  |  |  |  |  |  | **DPB1*16** | 0.00202 | 988 |
|  |  |  | **B*50** | 0.03004 | 2164 |  |  |  |  |  |  |  |  |  | **DPB1*17** | 0.02227 | 988 |
|  |  |  | **B*51** | 0.11969 | 2164 |  |  |  |  |  |  |  |  |  | **DPB1*18** | 0.00202 | 988 |
|  |  |  | **B*52** | 0.03651 | 2164 |  |  |  |  |  |  |  |  |  | **DPB1*19** | 0.00101 | 988 |
|  |  |  | **B*53** | 0.00277 | 2164 |  |  |  |  |  |  |  |  |  | **DPB1*22** | 0.00101 | 988 |
|  |  |  | **B*54** | 0.00139 | 2164 |  |  |  |  |  |  |  |  |  | **DPB1*23** | 0.00607 | 988 |
|  |  |  | **B*55** | 0.03327 | 2164 |  |  |  |  |  |  |  |  |  | **DPB1*26** | 0.00202 | 988 |
|  |  |  | **B*56** | 0.00555 | 2164 |  |  |  |  |  |  |  |  |  | **DPB1*28** | 0.00101 | 988 |
|  |  |  | **B*57** | 0.01525 | 2164 |  |  |  |  |  |  |  |  |  | **DPB1*296** | 0.00101 | 988 |
|  |  |  | **B*58** | 0.01664 | 2164 |  |  |  |  |  |  |  |  |  | **DPB1*36** | 0.00101 | 988 |
|  |  |  | **B*62** | 0.00046 | 2164 |  |  |  |  |  |  |  |  |  | **DPB1*37** | 0.00101 | 988 |
|  |  |  | **B*73** | 0.00139 | 2164 |  |  |  |  |  |  |  |  |  | **DPB1*415** | 0.00101 | 988 |
|  |  |  |  |  |  |  |  |  |  |  |  |  |  |  | **DPB1*47** | 0.00304 | 988 |
|  |  |  |  |  |  |  |  |  |  |  |  |  |  |  | **DPB1*51** | 0.00405 | 988 |
|  |  |  |  |  |  |  |  |  |  |  |  |  |  |  | **DPB1*535** | 0.00101 | 988 |
|  |  |  |  |  |  |  |  |  |  |  |  |  |  |  | **DPB1*66** | 0.00304 | 988 |
|  |  |  |  |  |  |  |  |  |  |  |  |  |  |  | **DPB1*70** | 0.00101 | 988 |
|  |  |  |  |  |  |  |  |  |  |  |  |  |  |  | **DPB1*79** | 0.00101 | 988 |
|  |  |  |  |  |  |  |  |  |  |  |  |  |  |  | **DPB1*91** | 0.00101 | 988 |

Abbreviations: AF, allele frequency; 2n, allele count

**Table S3.** The allele frequency for HLA-A, -B, -C, -DRB1, -DQB1 and -DPB1 in solid organ transplantation candidates.

|  | **AF** | **2n** |  | **AF** | **2n** |  | **AF** | **2n** |  | **AF** | **2n** |  | **AF** | **2n** |  | **AF** | **2n** |
| --- | --- | --- | --- | --- | --- | --- | --- | --- | --- | --- | --- | --- | --- | --- | --- | --- | --- |
| **A*01** | 0.12500 | 1936 | **B*04** | 0.00052 | 1936 | **C*01** | 0.04167 | 1056 | **DRB1*01** | 0.06612 | 1936 | **DQB1*02** | 0.16157 | 458 | **DPB1*01** | 0.00362 | 276 |
| **A*02** | 0.2314 | 1936 | **B*07** | 0.05062 | 1936 | **C*02** | 0.03314 | 1056 | **DRB1*03** | 0.08884 | 1936 | **DQB1*03** | 0.42576 | 458 | **DPB1*02** | 0.20652 | 276 |
| **A*03** | 0.10537 | 1936 | **B*08** | 0.03822 | 1936 | **C*03** | 0.06723 | 1056 | **DRB1*04** | 0.16426 | 1936 | **DQB1*04** | 0.01965 | 458 | **DPB1*03** | 0.08333 | 276 |
| **A*11** | 0.07438 | 1936 | **B*13** | 0.03771 | 1936 | **C*04** | 0.19223 | 1056 | **DRB1*07** | 0.08729 | 1936 | **DQB1*05** | 0.21834 | 458 | **DPB1*04** | 0.53623 | 276 |
| **A*23** | 0.04029 | 1936 | **B*14** | 0.02893 | 1936 | **C*05** | 0.02273 | 1056 | **DRB1*08** | 0.02841 | 1936 | **DQB1*06** | 0.17467 | 458 | **DPB1*05** | 0.01812 | 276 |
| **A*24** | 0.14618 | 1936 | **B*15** | 0.04390 | 1936 | **C*06** | 0.11837 | 1056 | **DRB1*09** | 0.00878 | 1936 |  |  |  | **DPB1*06** | 0.00362 | 276 |
| **A*25** | 0.00878 | 1936 | **B*18** | 0.05630 | 1936 | **C*07** | 0.19981 | 1056 | **DRB1*10** | 0.02066 | 1936 |  |  |  | **DPB1*09** | 0.00725 | 276 |
| **A*26** | 0.04700 | 1936 | **B*19** | 0.00052 | 1936 | **C*08** | 0.0322 | 1056 | **DRB1*11** | 0.18957 | 1936 |  |  |  | **DPB1*10** | 0.00362 | 276 |
| **A*29** | 0.02014 | 1936 | **B*27** | 0.03151 | 1936 | **C*12** | 0.14205 | 1056 | **DRB1*12** | 0.02324 | 1936 |  |  |  | **DPB1*105** | 0.00362 | 276 |
| **A*30** | 0.03977 | 1936 | **B*35** | 0.20610 | 1936 | **C*14** | 0.04072 | 1056 | **DRB1*13** | 0.11777 | 1936 |  |  |  | **DPB1*11** | 0.00725 | 276 |
| **A*31** | 0.02428 | 1936 | **B*37** | 0.01291 | 1936 | **C*15** | 0.05871 | 1056 | **DRB1*14** | 0.05992 | 1936 |  |  |  | **DPB1*13** | 0.03986 | 276 |
| **A*32** | 0.04442 | 1936 | **B*38** | 0.04287 | 1936 | **C*16** | 0.03598 | 1056 | **DRB1*15** | 0.10072 | 1936 |  |  |  | **DPB1*131** | 0.00362 | 276 |
| **A*33** | 0.03926 | 1936 | **B*39** | 0.01653 | 1936 | **C*17** | 0.01042 | 1056 | **DRB1*16** | 0.04442 | 1936 |  |  |  | **DPB1*14** | 0.01812 | 276 |
| **A*34** | 0.00052 | 1936 | **B*40** | 0.04236 | 1936 | **C*18** | 0.00473 | 1056 |  |  |  |  |  |  | **DPB1*15** | 0.00362 | 276 |
| **A*35** | 0.00052 | 1936 | **B*41** | 0.02169 | 1936 |  |  |  |  |  |  |  |  |  | **DPB1*17** | 0.03623 | 276 |
| **A*36** | 0.00052 | 1936 | **B*42** | 0.00155 | 1936 |  |  |  |  |  |  |  |  |  | **DPB1*23** | 0.01087 | 276 |
| **A*66** | 0.00568 | 1936 | **B*44** | 0.07541 | 1936 |  |  |  |  |  |  |  |  |  | **DPB1*30** | 0.00362 | 276 |
| **A*68** | 0.03771 | 1936 | **B*45** | 0.00103 | 1936 |  |  |  |  |  |  |  |  |  | **DPB1*45** | 0.00362 | 276 |
| **A*69** | 0.00826 | 1936 | **B*46** | 0.00155 | 1936 |  |  |  |  |  |  |  |  |  | **DPB1*47** | 0.00362 | 276 |
| **A*74** | 0.00052 | 1936 | **B*48** | 0.00465 | 1936 |  |  |  |  |  |  |  |  |  | **DPB1*51** | 0.00362 | 276 |
|  |  |  | **B*49** | 0.04442 | 1936 |  |  |  |  |  |  |  |  |  |  |  |  |
|  |  |  | **B*50** | 0.03048 | 1936 |  |  |  |  |  |  |  |  |  |  |  |  |
|  |  |  | **B*51** | 0.10692 | 1936 |  |  |  |  |  |  |  |  |  |  |  |  |
|  |  |  | **B*52** | 0.02583 | 1936 |  |  |  |  |  |  |  |  |  |  |  |  |
|  |  |  | **B*53** | 0.00413 | 1936 |  |  |  |  |  |  |  |  |  |  |  |  |
|  |  |  | **B*54** | 0.00310 | 1936 |  |  |  |  |  |  |  |  |  |  |  |  |
|  |  |  | **B*55** | 0.02944 | 1936 |  |  |  |  |  |  |  |  |  |  |  |  |
|  |  |  | **B*56** | 0.00207 | 1936 |  |  |  |  |  |  |  |  |  |  |  |  |
|  |  |  | **B*57** | 0.01808 | 1936 |  |  |  |  |  |  |  |  |  |  |  |  |
|  |  |  | **B*58** | 0.02014 | 1936 |  |  |  |  |  |  |  |  |  |  |  |  |
|  |  |  | **B*78** | 0.00052 | 1936 |  |  |  |  |  |  |  |  |  |  |  |  |

Abbreviations: AF, allele frequency; 2n, allele count

**Table S4.** The allele frequency for HLA-A, -B, -C, -DRB1, -DQB1 and -DPB1 in renal transplantation candidates.

|  | **AF** | **2n** |  | **AF** | **2n** |  | **AF** | **2n** |  | **AF** | **2n** |  | **AF** | **2n** |  | **AF** | **2n** |
| --- | --- | --- | --- | --- | --- | --- | --- | --- | --- | --- | --- | --- | --- | --- | --- | --- | --- |
| **A*01** | 0.11897 | 1824 | **B*04** | 0.00055 | 1824 | **C*01** | 0.04126 | 1018 | **DRB1*01** | 0.06469 | 1824 | **DQB1*02** | 0.16000 | 450 | **DPB1*01** | 0.00368 | 272 |
| **A*02** | 0.23191 | 1824 | **B*07** | 0.05154 | 1824 | **C*02** | 0.03242 | 1018 | **DRB1*03** | 0.09101 | 1824 | **DQB1*03** | 0.42444 | 450 | **DPB1*02** | 0.20588 | 272 |
| **A*03** | 0.10965 | 1824 | **B*08** | 0.03947 | 1824 | **C*03** | 0.06974 | 1018 | **DRB1*04** | 0.16831 | 1824 | **DQB1*04** | 0.02000 | 450 | **DPB1*03** | 0.07721 | 272 |
| **A*11** | 0.07621 | 1824 | **B*13** | 0.03947 | 1824 | **C*04** | 0.18959 | 1018 | **DRB1*07** | 0.08882 | 1824 | **DQB1*05** | 0.22222 | 450 | **DPB1*04** | 0.54044 | 272 |
| **A*23** | 0.04221 | 1824 | **B*14** | 0.02796 | 1824 | **C*05** | 0.02358 | 1018 | **DRB1*08** | 0.02851 | 1824 | **DQB1*06** | 0.17333 | 450 | **DPB1*05** | 0.01838 | 272 |
| **A*24** | 0.14364 | 1824 | **B*15** | 0.04441 | 1824 | **C*06** | 0.11788 | 1018 | **DRB1*09** | 0.00877 | 1824 |  |  |  | **DPB1*06** | 0.00368 | 272 |
| **A*25** | 0.00877 | 1824 | **B*18** | 0.05592 | 1824 | **C*07** | 0.20236 | 1018 | **DRB1*10** | 0.02138 | 1824 |  |  |  | **DPB1*09** | 0.00735 | 272 |
| **A*26** | 0.04825 | 1824 | **B*19** | 0.00055 | 1824 | **C*08** | 0.03242 | 1018 | **DRB1*11** | 0.19079 | 1824 |  |  |  | **DPB1*10** | 0.00368 | 272 |
| **A*29** | 0.01974 | 1824 | **B*27** | 0.02961 | 1824 | **C*12** | 0.13752 | 1018 | **DRB1*12** | 0.02303 | 1824 |  |  |  | **DPB1*105** | 0.00368 | 272 |
| **A*30** | 0.04167 | 1824 | **B*35** | 0.20833 | 1824 | **C*14** | 0.04126 | 1018 | **DRB1*13** | 0.11075 | 1824 |  |  |  | **DPB1*11** | 0.00735 | 272 |
| **A*31** | 0.02303 | 1824 | **B*37** | 0.01261 | 1824 | **C*15** | 0.05992 | 1018 | **DRB1*14** | 0.05976 | 1824 |  |  |  | **DPB1*13** | 0.04044 | 272 |
| **A*32** | 0.04441 | 1824 | **B*38** | 0.04276 | 1824 | **C*16** | 0.03733 | 1018 | **DRB1*15** | 0.10033 | 1824 |  |  |  | **DPB1*131** | 0.00368 | 272 |
| **A*33** | 0.03838 | 1824 | **B*39** | 0.01645 | 1824 | **C*17** | 0.01081 | 1018 | **DRB1*16** | 0.04386 | 1824 |  |  |  | **DPB1*14** | 0.01838 | 272 |
| **A*34** | 0.00055 | 1824 | **B*40** | 0.04221 | 1824 | **C*18** | 0.00393 | 1018 |  |  |  |  |  |  | **DPB1*15** | 0.00368 | 272 |
| **A*35** | 0.00055 | 1824 | **B*41** | 0.02029 | 1824 |  |  |  |  |  |  |  |  |  | **DPB1*17** | 0.03676 | 272 |
| **A*36** | 0.00055 | 1824 | **B*42** | 0.00164 | 1824 |  |  |  |  |  |  |  |  |  | **DPB1*23** | 0.01103 | 272 |
| **A*66** | 0.00493 | 1824 | **B*44** | 0.07511 | 1824 |  |  |  |  |  |  |  |  |  | **DPB1*30** | 0.00368 | 272 |
| **A*68** | 0.03783 | 1824 | **B*45** | 0.00110 | 1824 |  |  |  |  |  |  |  |  |  | **DPB1*45** | 0.00368 | 272 |
| **A*69** | 0.00822 | 1824 | **B*46** | 0.00164 | 1824 |  |  |  |  |  |  |  |  |  | **DPB1*47** | 0.00368 | 272 |
| **A*74** | 0.00055 | 1824 | **B*48** | 0.00493 | 1824 |  |  |  |  |  |  |  |  |  | **DPB1*51** | 0.00368 | 272 |
|  |  |  | **B*49** | 0.04441 | 1824 |  |  |  |  |  |  |  |  |  |  |  |  |
|  |  |  | **B*50** | 0.03015 | 1824 |  |  |  |  |  |  |  |  |  |  |  |  |
|  |  |  | **B*51** | 0.10526 | 1824 |  |  |  |  |  |  |  |  |  |  |  |  |
|  |  |  | **B*52** | 0.02577 | 1824 |  |  |  |  |  |  |  |  |  |  |  |  |
|  |  |  | **B*53** | 0.00439 | 1824 |  |  |  |  |  |  |  |  |  |  |  |  |
|  |  |  | **B*54** | 0.00329 | 1824 |  |  |  |  |  |  |  |  |  |  |  |  |
|  |  |  | **B*55** | 0.02906 | 1824 |  |  |  |  |  |  |  |  |  |  |  |  |
|  |  |  | **B*56** | 0.00219 | 1824 |  |  |  |  |  |  |  |  |  |  |  |  |
|  |  |  | **B*57** | 0.01754 | 1824 |  |  |  |  |  |  |  |  |  |  |  |  |
|  |  |  | **B*58** | 0.02083 | 1824 |  |  |  |  |  |  |  |  |  |  |  |  |
|  |  |  | **B*78** | 0.00055 | 1824 |  |  |  |  |  |  |  |  |  |  |  |  |

Abbreviations: AF, allele frequency; 2n, allele count

**Table S5.** p-values (Comparison of HLA alleles in HSCT, solid organ, and renal transplantation candidates with donors).

| **HLA- A*** | **Donor vs HSCT** | **Donor vs solid tx** | **Donor-renal tx** | **HLA- B*** | **Donor vs HSCT** | **Donor vs solid tx** | **Donor vs renal tx** | **HLA- C*** | **Donor vs HSCT** | **Donor vs solid tx** | **Donor vs renal tx** | **HLA-DRB1*** | **Donor vs HSCT** | **Donor vs solid tx** | **Donor vs renal tx** | **HLA-DQB1*** | **Donor vs HSCT** | **Donor vs solid tx** | **Donor vs renal tx** | **HLA-DPB1*** | **Donor vs HSCT** | **Donor vs solid tx** | **Donor vs renal tx** |
| --- | --- | --- | --- | --- | --- | --- | --- | --- | --- | --- | --- | --- | --- | --- | --- | --- | --- | --- | --- | --- | --- | --- | --- |
| 01 | 0.942 | 0.182 | 0.556 | 03 | 1.000 | 1.000 | 1.000 | 01 | 0.411 | 0.722 | 0.786 | 01 | 0.856 | 0.504 | 0.377 | 02 | 0.454 | 0.885 | 0.826 | 01 | 0.713 | 0.333 | 0.332 |
| **02** | 0.186 | 0.113 | 0.139 | 04 | 1.000 | 0.245 | 0.233 | 02 | 0.338 | 0.924 | 0.846 | 02 | 1.000 | 1.000 | 1.000 | 03 | 0.358 | 0.381 | 0.408 | 02 | 0.228 | 0.345 | 0.344 |
| 03 | 0.366 | 0.193 | 0.480 | 07 | 0.297 | 0.912 | 1.000 | 03 | 0.778 | 0.184 | 0.116 | 03 | 0.799 | 0.288 | 0.188 | 04 | 0.812 | 0.493 | 0.492 | 03 | 0.259 | 1.000 | 0.807 |
| 11 | 0.793 | 0.818 | 0.962 | 08 | 0.283 | 0.709 | 0.949 | 04 | 0.457 | 0.965 | 0.823 | **04** | 0.450 | ***0.033**** | ***0.012**** | 05 | 0.962 | 0.844 | 0.693 | 04 | 0.489 | 0.545 | 0.636 |
| 22 | 1.000 | 1.000 | 1.000 | 13 | 0.899 | 0.553 | 0.345 | 05 | 0.917 | 0.821 | 0.909 | 07 | 0.575 | 0.424 | 0.575 | 06 | 0.546 | 0.448 | 0.405 | 05 | 0.737 | 1.000 | 1.000 |
| **23** | 0.244 | 0.090 | ***0.038**** | 14 | 0.813 | 0.074 | 0.133 | 06 | 0.454 | 0.217 | 0.232 | 08 | 0.174 | 0.204 | 0.193 |  |  |  |  | 06 | 0.709 | 0.568 | 0.563 |
| 24 | 0.347 | 0.657 | 0.483 | **15** | 0.411 | ***0.041**** | ***0.036**** | 07 | 0.408 | 0.371 | 0.463 | 09 | 0.455 | 0.214 | 0.246 |  |  |  |  | 08 | 0.594 | 1.000 | 1.000 |
| 25 | 0.213 | 0.626 | 0.618 | 16 | 1.000 | 1.000 | 1.000 | 08 | 0.931 | 0.514 | 0.570 | 10 | 0.815 | 0.622 | 0.800 |  |  |  |  | 09 | 0.508 | 0.400 | 0.561 |
| 26 | 0.766 | 0.057 | 0.104 | 18 | 0.960 | 0.794 | 0.748 | 10 | 1.000 | 1.000 | 1.000 | **11** | 0.977 | ***0.041**** | 0.060 |  |  |  |  | 10 | 0.861 | 0.150 | 0.150 |
| 29 | 0.460 | 1.000 | 1.000 | 19 | 1.000 | 0.245 | 0.233 | 12 | 0.559 | 0.147 | 0.291 | **12** | 1.000 | ***0.012**** | ***0.017**** |  |  |  |  | 104 | 0.243 | 1.000 | 1.000 |
| 30 | 0.521 | 0.232 | 0.118 | 24 | 1.000 | 1.000 | 1.000 | 13 | 1.000 | 1.000 | 1.000 | 13 | 0.288 | 0.674 | 0.665 |  |  |  |  | 105 | 0.561 | 1.000 | 1.000 |
| **31** | 0.508 | ***0.008**** | ***0.024**** | 27 | 0.349 | 1.000 | 0.720 | 14 | 0.620 | 1.000 | 0.929 | 14 | 0.669 | 0.615 | 0.606 |  |  |  |  | 11 | 0.695 | 0.318 | 0.312 |
| 32 | 0.116 | 0.771 | 0.811 | 32 | 1.000 | 1.000 | 1.000 | 15 | 0.799 | 0.266 | 0.325 | 15 | 0.612 | 0.682 | 0.736 |  |  |  |  | 124 | 0.455 | 1.000 | 1.000 |
| **33** | 0.678 | ***0.015**** | ***0.028**** | 34 | 1.000 | 1.000 | 1.000 | 16 | 0.399 | 0.592 | 0.786 | 16 | 0.748 | 0.395 | 0.352 |  |  |  |  | 126 | 1.000 | 1.000 | 1.000 |
| 34 | 1.000 | 0.344 | 0.329 | 35 | 0.690 | 0.611 | 0.783 | 17 | 0.716 | 0.127 | 0.162 |  |  |  |  |  |  |  |  | 129 | 1.000 | 1.000 | 1.000 |
| 35 | 1.000 | 0.131 | 0.124 | 37 | 0.766 | 0.755 | 0.670 | 18 | 0.800 | 0.800 | 1.000 |  |  |  |  |  |  |  |  | 13 | 0.339 | 0.083 | 0.081 |
| 36 | 1.000 | 0.569 | 0.549 | 38 | 0.954 | 0.808 | 0.851 |  |  |  |  |  |  |  |  |  |  |  |  | 131 | 0.504 | 0.467 | 0.463 |
| 38 | 1.000 | 1.000 | 1.000 | 39 | 0.853 | 0.771 | 0.841 |  |  |  |  |  |  |  |  |  |  |  |  | 14 | 0.607 | 0.589 | 0.585 |
| 50 | 0.144 | 1.000 | 1.000 | 40 | 0.854 | 0.277 | 0.294 |  |  |  |  |  |  |  |  |  |  |  |  | 15 | 0.675 | 0.329 | 0.329 |
| 66 | 0.870 | 0.610 | 1.000 | 41 | 0.648 | 0.629 | 0.407 |  |  |  |  |  |  |  |  |  |  |  |  | 16 | 0.594 | 1.000 | 1.000 |
| 68 | 0.758 | 0.898 | 0.895 | **42** | 1.000 | ***0.008**** | ***0.007**** |  |  |  |  |  |  |  |  |  |  |  |  | 17 | 1.000 | 0.203 | 0.199 |
| 69 | 0.769 | 0.369 | 0.358 | 44 | 1.000 | 0.449 | 0.496 |  |  |  |  |  |  |  |  |  |  |  |  | 18 | 1.000 | 1.000 | 1.000 |
| 74 | 1.000 | 0.430 | 0.412 | 45 | 0.354 | 0.223 | 0.313 |  |  |  |  |  |  |  |  |  |  |  |  | 189 | 1.000 | 1.000 | 1.000 |
|  |  |  |  | 46 | 1.000 | 0.434 | 0.420 |  |  |  |  |  |  |  |  |  |  |  |  | 19 | 0.385 | 1.000 | 1.000 |
|  |  |  |  | 47 | 1.000 | 0.240 | 0.242 |  |  |  |  |  |  |  |  |  |  |  |  | 22 | 1.000 | 1.000 | 1.000 |
|  |  |  |  | 48 | 0.867 | 1.000 | 0.858 |  |  |  |  |  |  |  |  |  |  |  |  | 23 | 0.779 | 0.384 | 0.382 |
|  |  |  |  | 49 | 0.058 | 0.234 | 0.247 |  |  |  |  |  |  |  |  |  |  |  |  | 26 | 0.466 | 0.590 | 0.591 |
|  |  |  |  | 50 | 0.400 | 0.497 | 0.444 |  |  |  |  |  |  |  |  |  |  |  |  | 28 | 0.455 | 1.000 | 1.000 |
|  |  |  |  | **51** | 0.646 | ***0.040**** | ***0.026**** |  |  |  |  |  |  |  |  |  |  |  |  | 296 | 0.455 | 1.000 | 1.000 |
|  |  |  |  | 52 | 0.092 | 0.385 | 0.373 |  |  |  |  |  |  |  |  |  |  |  |  | 30 | 1.000 | 0.189 | 0.187 |
|  |  |  |  | 53 | 0.569 | 0.847 | 0.696 |  |  |  |  |  |  |  |  |  |  |  |  | 36 | 1.000 | 1.000 | 1.000 |
|  |  |  |  | **54** | 0.737 | ***0.048**** | ***0.038**** |  |  |  |  |  |  |  |  |  |  |  |  | 37 | 0.455 | 1.000 | 1.000 |
|  |  |  |  | 55 | 0.742 | 0.626 | 0.567 |  |  |  |  |  |  |  |  |  |  |  |  | 415 | 0.455 | 1.000 | 1.000 |
|  |  |  |  | 56 | 0.130 | 0.398 | 0.513 |  |  |  |  |  |  |  |  |  |  |  |  | 45 | 1.000 | 0.342 | 0.339 |
|  |  |  |  | 57 | 0.427 | 1.000 | 1.000 |  |  |  |  |  |  |  |  |  |  |  |  | 47 | 0.664 | 0.467 | 0.463 |
|  |  |  |  | 58 | 0.711 | 0.149 | 0.114 |  |  |  |  |  |  |  |  |  |  |  |  | 503 | 1.000 | 1.000 | 1.000 |
|  |  |  |  | 59 | 1.000 | 1.000 | 1.000 |  |  |  |  |  |  |  |  |  |  |  |  | 51 | 0.420 | 0.467 | 0.463 |
|  |  |  |  | 60 | 1.000 | 1.000 | 1.000 |  |  |  |  |  |  |  |  |  |  |  |  | 535 | 0.455 | 1.000 | 1.000 |
|  |  |  |  | 62 | 0.144 | 1.000 | 1.000 |  |  |  |  |  |  |  |  |  |  |  |  | 61 | 1.000 | 1.000 | 1.000 |
|  |  |  |  | 66 | 1.000 | 1.000 | 1.000 |  |  |  |  |  |  |  |  |  |  |  |  | 66 | 0.664 | 1.000 | 1.000 |
|  |  |  |  | 73 | 0.418 | 0.378 | 0.624 |  |  |  |  |  |  |  |  |  |  |  |  | 70 | 1.000 | 1.000 | 1.000 |
|  |  |  |  | 78 | 1.000 | 0.131 | 0.124 |  |  |  |  |  |  |  |  |  |  |  |  | 79 | 0.455 | 1.000 | 1.000 |
|  |  |  |  | 82 | 1.000 | 1.000 | 1.000 |  |  |  |  |  |  |  |  |  |  |  |  | 84 | 1.000 | 1.000 | 1.000 |
|  |  |  |  | 83 | 1.000 | 1.000 | 1.000 |  |  |  |  |  |  |  |  |  |  |  |  | 91 | 1.000 | 1.000 | 1.000 |

*significant (p < 0.05)

Abbreviations: Tx, transplantation; HSCT, hematopoietic stem cell transplant

**Table S6**. Slatkin’s implementation of Ewens-Watterson homozygosity test of neutrality.

| **Donors** | | | | | | |
| --- | --- | --- | --- | --- | --- | --- |
| **Locus** | **k** | **F. obs** | **F. exp** | **Variance in F.** | **Fnd** | **P** |
| A | 21 | 0.1272 | 0.2896 | 0.0144 | –1.3517 | 0.0085* |
| B | 41 | 0.0859 | 0.1600 | 0.0039 | –1.1921 | 0.0277 |
| C | 16 | 0.1256 | 0.3188 | 0.0164 | –1.5071 | 0.0024** |
| DRB1 | 14 | 0.1163 | 0.3924 | 0.0250 | –1.7472 | 0.0000**** |
| DQB1 | 5 | 0.2719 | 0.6224 | 0.0392 | –1.7694 | 0.0068* |
| DPB1 | 36 | 0.3531 | 0.1246 | 0.0019 | **5.2917** | 0.9975** |
| **HSCT candidates** | | | | | | |
| **Locus** | **k** | **F obs** | **F exp** | **Variance in F** | **Fnd** | **P** |
| A | 17 | 0.1246 | 0.2835 | 0.0131 | –1.3894 | 0.0060* |
| B | 30 | 0.0843 | 0.1682 | 0.0041 | –1.3079 | 0.0107* |
| C | 14 | 0.1269 | 0.3117 | 0.0154 | –1.4913 | 0.0013** |
| DRB1 | 13 | 0.1165 | 0.3527 | 0.0193 | –1.6990 | 0.0001*** |
| DQB1 | 5 | 0.2799 | 0.6167 | 0.0388 | –1.7098 | 0.0107* |
| DPB1 | 37 | 0.3425 | 0.1170 | 0.0016 | **5.6852** | 0.9985** |
| **Solid transplant candidates** | | | | | | |
| **Locus** | **k** | **F obs** | **F exp** | **Variance in F** | **Fnd** | **P** |
| A | 20 | 0.1187 | 0.2428 | 0.0094 | –1.2810 | 0.0139* |
| B | 31 | 0.0817 | 0.1601 | 0.0036 | –1.2982 | 0.0106* |
| C | 14 | 0.1265 | 0.3045 | 0.0146 | –1.4755 | 0.0014** |
| DRB1 | 13 | 0.1142 | 0.3484 | 0.0188 | –1.7072 | 0.0001*** |
| DQB1 | 5 | 0.2859 | 0.5760 | 0.0352 | –1.5468 | 0.0224* |
| DPB1 | 20 | 0.3411 | 0.1698 | 0.0036 | **2.8520** | 0.9803* |

* = significant at the %5 level, ** = significant at the %1 level, *** = significant at the %0.1 level, **** = significant at the %0.01 level, ***** = significant at the %0.001 level

Abbreviations: k, distinct alleles; F obs, Observed FX; F exp, Expected F; Fnd, Normalized deviate of F; p, p-value of F; HSCT, hematopoietic stem cell transplantation

**Table S7**. Hardy-Weinberg Equilibrium analysis of HLA loci in donors. HSCT candidates. and solid organ transplantation candidates.

|  | | | | |
| --- | --- | --- | --- | --- |
| **Locus** | **Obs Het** | **Exp Het** | **Chi-square** | **p-HWE** |
| **Donors** | | | | |
| A | 5420 | 5605.2 | 6.12 | *0.0134** |
| B | 5796 | 5870.45 | 0.94 | 0.3312 |
| C | 1589 | 1684.16 | 5.38 | *0.0204** |
| DRB1 | 5553 | 5675.43 | 2.6 | 0.1041 |
| DQB1 | 479 | 509.69 | 1.85 | 0.1741 |
| DPB1 | 387 | 382.97 | 0.04 | 0.8367 |
| **HSCT candidates** | | | | |
| A | 908 | 947.22 | 1.62 | 0.2026 |
| B | 968 | 990.78 | 0.52 | 0.4693 |
| C | 527 | 550.95 | 1.04 | 0.3076 |
| DRB1 | 938 | 955.93 | 0.34 | 0.5620 |
| DQB1 | 421 | 432.80 | 0.32 | 0.5707 |
| DPB1 | 318 | 324.80 | 0.14 | 0.7061 |
| **Solid transplant candidates** | | | | |
| A | 837 | 853.10 | 0.30 | 0.5815 |
| B | 881 | 888.88 | 0.07 | 0.7916 |
| C | 449 | 461.20 | 0.32 | 0.5698 |
| DRB1 | 838 | 857.43 | 0.44 | 0.5071 |
| DQB1 | 157 | 163.52 | 0.26 | 0.6103 |
| DPB1 | 95 | 90.93 | 0.18 | 0.6699 |
| **Renal transplant candidates** | | | | |
| A | 793 | 804.19 | 0.16 | 0.6931 |
| B | 831 | 836.94 | 0.04 | 0.8374 |
| C | 433 | 445 | 0.32 | 0.5695 |
| DRB1 | 787 | 807.31 | 0.51 | 0.4747 |
| DQB1 | 154 | 160.74 | 0.28 | 0.5948 |
| DPB1 | 93 | 89.15 | 0.17 | 0.6838 |

* = significant at the 5% level.

Abbreviations: Obs Het, observed heterozygotes; Exp Het, expected heterozygotes; HSCT, hematopoietic stem cell transplantation

**Table S8.** Heterozygotes of the most common HLA alleles.

| **Donors** | | | | **HSCT candidates** | | | | **Solid transplant candidates** | | | | **Renal transplant candidates** | | | |
| --- | --- | --- | --- | --- | --- | --- | --- | --- | --- | --- | --- | --- | --- | --- | --- |
| **Allel** | **Obs** | **Exp** | **p** | **Allel** | **Obs** | **Exp** | **p** | **Allel** | **Obs** | **Exp** | **p** | **Allel** | **Obs** | **Exp** | **p** |
| **A*02** | 2282 | 2396.71 | *0.0191** | **A*02** | 372 | 388.75 | 0.3957 | **A*02** | 326 | 344.33 | 0.3232 | **A*02** | 307 | 324.90 | 0.3206 |
| **A*24** | 11583 | 1639.29 | 0.1644 | **A*24** | 288 | 287.95 | 0.9977 | **A*24** | 241 | 241.63 | 0.9676 | **A*24** | 228 | 224.37 | 0.8083 |
| **A*03** | 1302 | 1314.08 | 0.7390 | **A*03** | 217 | 232.55 | 0.3079 | **A*01** | 204 | 211.75 | 0.5943 | **A*01** | 185 | 191.18 | 0.6547 |
| **B*35** | 2215 | 2142.25 | 0.1160 | **B*35** | 357 | 355.84 | 0.9509 | **B*35** | 323 | 316.77 | 0.7262 | **B*35** | 310 | 300.83 | 0.5971 |
| **B*51** | 1322 | 1390.16 | 0.0675 | **B*51** | 221 | 228.00 | 0.6429 | **B*51** | 183 | 184.87 | 0.8908 | **B*51** | 170 | 171.79 | 0.8914 |
| **B*44** | 844 | 843.81 | 0.9948 | **B*44** | 139 | 142.18 | 0.7895 | **B*44** | 138 | 134.99 | 0.7956 | **B*44** | 129 | 126.71 | 0.8388 |
| **C*07** | 579 | 646.02 | *0.0084*** | **C*07** | 201 | 219.54 | 0.2109 | **C*07** | 165 | 168.84 | 0.7676 | **C*07** | 160 | 164.31 | 0.7364 |
| **C*04** | 546 | 600.30 | *0.0267** | **C*04** | 179 | 188.72 | 0.4794 | **C*04** | 153 | 163.98 | 0.3914 | **C*04** | 147 | 156.41 | 0.4518 |
| **C*12** | 392 | 421.69 | 0.1483 | **C*12** | 144 | 144.16 | 0.9890 | **C*12** | 126 | 128.69 | 00.812 | **C*12** | 118 | 120.75 | 0.8026 |
| **DRB1*11** | 2121 | 2130.68 | 0.8339 | **DRB1*11** | 347 | 359.33 | 0.5153 | **DRB1*11** | 295 | 297.43 | 0.8880 | **DRB1*11** | 278 | 281.61 | 0.8299 |
| **DRB1*04** | 1532 | 1599.16 | 0.0931 | **DRB1*04** | 279 | 278.98 | 0.9991 | **DRB1*04** | 260 | 265.77 | 0.7235 | **DRB1*04** | 249 | 255.33 | 0.6921 |
| **DRB1*13** | 1239 | 1300.99 | 0.0850 | **DRB1*13** | 198 | 205.55 | 0.5982 | **DRB1*13** | 194 | 201.15 | 0.6142 | **DRB1*13** | 172 | 179.63 | 0.5692 |
| **DQB1*03** | 322 | 336.40 | 0.4325 | **DQB1*03** | 304 | 292.67 | 0.5079 | **DQB1*03** | 105 | 111.98 | 0.5097 | **DQB1*03** | 103 | 109.93 | 0.5086 |
| **DQB1*05** | 219 | 235.14 | 0.2925 | **DQB1*05** | 190 | 202.62 | 0.3752 | **DQB1*05** | 76 | 78.17 | 0.8065 | **DQB1*05** | 76 | 77.78 | 0.8402 |
| **DQB1*06** | 199 | 217.31 | 0.2141 | **DQB1*06** | 159 | 179.10 | 0.1331 | **DQB1*02** | 64 | 62.04 | 0.8039 | **DQB1*02** | 62 | 60.48 | 0.8450 |
| **DPB1*04** | 304 | 292.09 | 0.4861 | **DPB1*04** | 248 | 245.21 | 0.8588 | **DPB1*04** | 76 | 68.64 | 0.3742 | **DPB1*04** | 75 | 67.56 | 0.3650 |
| **DPB1*02** | 177 | 175.96 | 0.9375 | **DPB1*02** | 152 | 159.51 | 0.5519 | **DPB1*02** | 49 | 45.23 | 0.5749 | **DPB1*02** | 48 | 44.47 | 0.5966 |
| **DPB1*03** | 87 | 90.72 | 0.6960 | **DPB1*03** | 63 | 64.18 | 0.8828 | **DPB1*03** | 21 | 21.08 | 0.9855 | **DPB1*03** | 19 | 19.38 | 0.9314 |

* = significant at the 5% level

Abbreviations: Obs, observed; Exp, expected; HSCT, hematopoietic stem cell transplant

**Table S9**. Most frequently HLA genotypes.

| **Donors** | | | | **HSCT candidates** | | | | **Solid transplant candidates** | | | | **Renal transplant candidates** | | | |
| --- | --- | --- | --- | --- | --- | --- | --- | --- | --- | --- | --- | --- | --- | --- | --- |
| **Locus** | **Obs** | **Exp** | **P-value** | **Locus** | **Obs** | **Exp** | **P-value** | **Locus** | **Obs** | **Exp** | **P-value** | **Locus** | **Obs** | **Exp** | **P-value** |
| **A** | | | | | | | | | | | | | | | |
| **02:24** | 459 | 478.80 | 0.3656 | **02:24** | 87 | 80.28 | 0.4536 | **02:02** | 61 | 51.83 | 0.2030 | **02:24** | 31 | 31.74 | 0.8955 |
| **02:02** | 453 | 395.65 | 0.0039** | **02:02** | 68 | 59.63 | 0.2782 | **02:24** | 55 | 65.49 | 0.1950 | **02:02** | 29 | 26.49 | 0.6255 |
| **B** | | | | | | | | | | | | | | | |
| **35:13** | 411 | 95.40 | 0.0000***** | **35:13** | 65 | 15.35 | 0.0000***** | **35:13** | 64 | 15.04 | 0.0000***** | **35:13** | 35 | 8.10 | 0.0000***** |
| **35:51** | 298 | 335.50 | 0.0406* | **35:35** | 46 | 46.58 | 0.9322 | **35:51** | 38 | 42.66 | 0.4754 | **35:51** | 22 | 23.05 | 0.8265 |
| **C** | | | | | | | | | | | | | | | |
| **04:07** | 142 | 158.57 | 0.1881 | **04:07** | 50 | 51.80 | 0.8024 | **04:07** | 34 | 40.56 | 0.3029 | **04:07** | 20 | 21.51 | 0.7450 |
| **07:07** | 121 | 87.49 | 0.0003*** | **07:07** | 41 | 31.73 | 0.0999 | **07:12** | 30 | 29.97 | 0.9959 | **07:12** | 14 | 14.34 | 0.9286 |
| **DRB1** | | | | | | | | | | | | | | | |
| **04:11** | 414 | 393.09 | 0.2915 | **04:11** | 62 | 69.18 | 0.3883 | **04:11** | 58 | 60.28 | 0.7688 | **04:11** | 31 | 32.05 | 0.8526 |
| **11:11** | 288 | 283.16 | 0.7736 | **11:11** | 54 | 47.83 | 0.3726 | **11:13** | 43 | 43.22 | 0.9732 | **11:11** | 24 | 18.71 | 0.2216 |
| **DQB1** | | | | | | | | | | | | | | | |
| **03:03** | 120 | 112.80 | 0.4979 | **03:05** | 110 | 108.18 | 0.8611 | **03:03** | 45 | 41.51 | 0.5883 | **03:05** | 29 | 24.46 | 0.3585 |
| **03:05** | 110 | 120.03 | 0.3601 | **03:03** | 100 | 105.66 | 0.5816 | **03:05** | 45 | 42.58 | 0.7103 | **03:03** | 26 | 24.67 | 0.7894 |
| **DPB1** | | | | | | | | | | | | | | | |
| **04:04** | 178 | 183.95 | 0.6607 | **04:04** | 144 | 145.39 | 0.9080 | **04:04** | 36 | 39.68 | 0.5590 | **04:04** | 24 | 25.25 | 0.8040 |
| **02:04** | 129 | 119.85 | 0.4032 | **02:04** | 110 | 108.50 | 0.8857 | **02:04** | 34 | 30.57 | 0.5344 | **02:04** | 20 | 18.87 | 0.7940 |

* = significant at the 5% level, ** = significant at the 1% level, *** = significant at the 0.1% level

Abbreviations: Obs, observed; Exp, expected; HSCT, hematopoietic stem cell transplant

**Table S10.** Pairwise LD estimates.

| Group | Locus pair | D | D' | Wn | ln(L1) | ln(L0) | S | Permu | p-value |
| --- | --- | --- | --- | --- | --- | --- | --- | --- | --- |
| Donor | A:C | 0.00374 | 0.24323 | 0.20233 | –15229.87 | –15727.89 | 996.04 | 989 | 0.0000* |
| HSCT | A:C | 0.00468 | 0.29527 | 0.24736 | –4906.06 | –5130.10 | 448.09 | 983 | 0.0000* |
| Solid | A:C | 0.00438 | 0.29147 | 0.25296 | –4072.78 | –4267.40 | 389.24 | 973 | 0.0000* |
| Donor | A:B | * | * | * | — | — | NaN | — | — |
| HSCT | A:B | 0.00503 | 0.36983 | 0.29707 | –9308.53 | –9907.12 | 1197.19 | 956 | 0.0000* |
| Solid | A:B | 0.00433 | 0.36451 | 0.30462 | –8432.40 | –8978.07 | 1091.34 | 957 | 0.0000* |
| Donor | A:DRB1 | * | * | * | — | — | NaN | — | — |
| HSCT | A:DRB1 | 0.00361 | 0.21042 | 0.15884 | –8631.42 | –8796.21 | 329.58 | 975 | 0.0000* |
| Solid | A:DRB1 | 0.00301 | 0.21066 | 0.16975 | –7803.15 | –7968.30 | 330.31 | 956 | 0.0000* |
| Donor | A:DQB1 | 0.00435 | 0.13097 | 0.15943 | –4442.24 | –4482.57 | 80.66 | 999 | 0.1151 |
| HSCT | A:DQB1 | 0.00529 | 0.16424 | 0.18676 | –3807.57 | –3850.88 | 86.61 | 995 | 0.0291* |
| Solid | A:DQB1 | 0.00655 | 0.22653 | 0.28004 | –1388.01 | –1425.84 | 75.67 | 988 | 0.0800 |
| Donor | A:DPB1 | 0.00404 | 0.23575 | 0.35888 | –3891.17 | –4086.67 | 391.00 | 997 | 0.0000* |
| HSCT | A:DPB1 | 0.00339 | 0.23032 | 0.28549 | –3310.46 | –3479.13 | 337.34 | 997 | 0.0301* |
| Solid | A:DPB1 | 0.01034 | 0.40161 | 0.34832 | –838.58 | –913.31 | 149.46 | 997 | 0.1625 |
| Donor | C:B | 0.01421 | 0.78288 | 0.60710 | –13333.92 | –17471.28 | 8274.73 | 961 | 0.0000* |
| HSCT | C:B | 0.01433 | 0.80163 | 0.65889 | –4255.41 | –5679.95 | 2849.09 | 983 | 0.0000* |
| Solid | C:B | 0.01342 | 0.76050 | 0.62302 | –3652.63 | –4727.90 | 2150.55 | 992 | 0.0000* |
| Donor | C:DRB1 | 0.00569 | 0.29174 | 0.22613 | –14947.00 | –15524.38 | 1154.77 | 995 | 0.0000* |
| HSCT | C:DRB1 | 0.00526 | 0.29419 | 0.22817 | –4845.26 | –5052.20 | 413.89 | 980 | 0.0000* |
| Solid | C:DRB1 | 0.00496 | 0.28565 | 0.23232 | –4045.98 | –4212.62 | 333.28 | 968 | 0.0000* |
| Donor | C:DQB1 | 0.00904 | 0.23288 | 0.25768 | –3405.22 | –3489.15 | 167.85 | 994 | 0.0000* |
| HSCT | C:DQB1 | 0.01001 | 0.25134 | 0.24261 | –3042.47 | –3107.33 | 129.72 | 996 | 0.0000* |
| Solid | C:DQB1 | 0.00814 | 0.23605 | 0.25881 | –1361.50 | –1394.76 | 66.52 | 996 | 0.0994 |
| Donor | C:DPB1 | 0.00532 | 0.24158 | 0.25488 | –2867.15 | –3006.46 | 278.61 | 991 | 0.0081* |
| HSCT | C:DPB1 | 0.00712 | 0.28903 | 0.27774 | –2530.83 | –2677.25 | 292.84 | 997 | 0.0050* |
| Solid | C:DPB1 | 0.01030 | 0.35006 | 0.42918 | –805.31 | –879.76 | 148.90 | 998 | 0.0561 |
| Donor | B:DQB1 | 0.00721 | 0.31616 | 0.37908 | –4892.93 | –5111.45 | 437.04 | 986 | 0.0000* |
| HSCT | B:DQB1 | 0.00865 | 0.34346 | 0.38722 | –4192.86 | –4387.99 | 390.26 | 984 | 0.0000* |
| Solid | B:DQB1 | 0.00854 | 0.38030 | 0.40993 | –1555.48 | –1639.06 | 167.16 | 996 | 0.0000* |
| Donor | B:DPB1 | 0.00460 | 0.30253 | 0.23271 | –4356.84 | –4631.07 | 548.46 | 996 | 0.0000* |
| HSCT | B:DPB1 | 0.00441 | 0.31758 | 0.23272 | –3697.67 | –3932.99 | 470.63 | 999 | 0.0110* |
| Solid | B:DPB1 | 0.00544 | 0.40087 | 0.40205 | –933.03 | –1035.67 | 205.28 | 999 | 0.1682 |
| Donor | DRB1:DQB1 | 0.03830 | 0.87768 | 0.82206 | –3251.74 | –4442.16 | 2380.84 | 998 | 0.0000* |
| HSCT | DRB1:DQB1 | 0.03844 | 0.87764 | 0.82099 | –2779.80 | –3787.54 | 2015.48 | 995 | 0.0000* |
| Solid | DRB1:DQB1 | 0.03853 | 0.90313 | 0.82442 | –1034.45 | –1446.73 | 824.56 | 994 | 0.0000* |
| Donor | DRB1:DPB1 | 0.00792 | 0.27770 | 0.25392 | –3868.94 | –4067.33 | 396.79 | 994 | 0.0000* |
| HSCT | DRB1:DPB1 | 0.00690 | 0.26833 | 0.28389 | –3269.44 | –3446.26 | 353.62 | 996 | 0.0000* |
| Solid | DRB1:DPB1 | 0.00718 | 0.33180 | 0.29816 | –864.65 | –928.32 | 127.36 | 999 | 0.6216 |
| Donor | DQB1:DPB1 | 0.00615 | 0.16621 | 0.26964 | –2958.45 | –3045.38 | 173.84 | 995 | 0.0000* |
| HSCT | DQB1:DPB1 | 0.00862 | 0.18800 | 0.26302 | –2538.35 | –2617.08 | 157.45 | 995 | 0.0040* |
| Solid | DQB1:DPB1 | 0.00811 | 0.21954 | 0.34341 | –644.22 | –676.76 | 65.09 | 998 | 0.1593 |

†no data after filtering in Pypop, *significant (p < 0.05); Loglikelihood under linkage equilibrium [ln(L_0)], Loglikelihood obtained via the EM algorithm [ln(L_1)], D’. Hedrick’s statistic (Hedrick. 1987); Wn, Cramer’s V statistic (Cramér. 1946) for global LD

Abbreviations: HSCT, hematopoietic stem cell transplant; Tx, transplantation

**Table S11.** Allele ranking for HLA–A loci.

|  | **Donor** | | **HSCT** | | **Solid transplant candidates** | |
| --- | --- | --- | --- | --- | --- | --- |
|  |  | **AF** |  | **AF** |  | **AF** |
| 1 | A*02 | 0.24821 | A*02 | 0.23475 | A*02 | 0.23140 |
| 2 | A*24 | 0.15019 | A*24 | 0.15804 | A*24 | 0.14618 |
| 3 | A*03 | 0.11570 | A*03 | 0.12246 | **A*01** | **0.12500** |
| **4** | **A*01** | **0.11437** | **A*01** | **0.11368** | A*03 | 0.10537 |
| 5 | A*11 | 0.07607 | A*11 | 0.07763 | A*11 | 0.07438 |
| 6 | A*26 | 0.05793 | A*26 | 0.05961 | A*26 | 0.04700 |
| 7 | A*32 | 0.04617 | A*32 | 0.03835 | A*32 | 0.04442 |
| ***8*** | ***A*68*** | ***0.03722*** | **A*23** | **0.03743** | **A*23** | **0.04029** |
| 9 | A*30 | 0.03434 | ***A*68*** | ***0.03558*** | A*30 | 0.03977 |
| **10** | **A*23** | **0.03262** | A*30 | 0.03142 | **A*33** | **0.03926** |
| **11** | **A*33** | **0.02881** | **A*33** | **0.03050** | ***A*68*** | ***0.03771*** |
| 12 | A*29 | 0.02009 | A*29 | 0.02264 | **A*31** | **0.02428** |
| **13** | **A*31** | **0.01565** | **A*31** | **0.01340** | A*29 | 0.02014 |
| 14 | A*25 | 0.01028 | A*25 | 0.01340 | A*25 | 0.00878 |
| 15 | A*69 | 0.00646 | A*69 | 0.00555 | A*69 | 0.00826 |
| 16 | A*66 | 0.00498 | A*66 | 0.00508 | A*66 | 0.00568 |
| 17 | A*36 | 0.00039 | **A*50** | **0.00046** | A*34 | 0.00052 |
| 18 | A*74 | 0.00023 |  |  | A*35 | 0.00052 |
| 19 | A*34 | 0.00016 |  |  | A*36 | 0.00052 |
| 20 | A*22 | 0.00008 |  |  | A*74 | 0.00052 |
| 21 | A*38 | 0.00008 |  |  |  |  |

Abbreviations: HSCT, hematopoietic stem cell transplant

**Table S12**. Allele ranking for HLA–B loci.

|  | **Donor** | | **HSCT** | | **Solid transplant candidates** | |
| --- | --- | --- | --- | --- | --- | --- |
|  |  | **AF** |  | **AF** |  | **AF** |
| 1 | B*35 | 0.21154 | B*35 | 0.20749 | B*35 | 0.2061 |
| 2 | B*51 | 0.12348 | B*51 | 0.11969 | B*51 | 0.1069 |
| 3 | B*44 | 0.07069 | B*44 | 0.07070 | B*44 | 0.0754 |
| 4 | B*18 | 0.05808 | B*18 | 0.05823 | B*18 | 0.0563 |
| 5 | B*07 | 0.05154 | B*07 | 0.05684 | B*07 | 0.0506 |
| ***6*** | ***B*38*** | ***0.04173*** | **B*49** | **0.04760** | **B*49** | **0.0444** |
| ***7*** | ***B*08*** | ***0.04025*** | ***B*38*** | ***0.04110*** | **B*15** | **0.0439** |
| **8** | **B*49** | **0.03877** | **B*15** | **0.03789** | ***B*38*** | ***0.0429*** |
| 9 | B*40 | 0.03722 | **B*52** | **0.03651** | B*40 | 0.0424 |
| 10 | B*13 | 0.03511 | B*40 | 0.03604 | ***B*08*** | ***0.0382*** |
| **11** | **B*15** | **0.03441** | ***B*08*** | ***0.03510*** | B*13 | 0.0377 |
| 12 | ***B*50*** | ***0.03379*** | B*13 | 0.03420 | B*27 | 0.0315 |
| 13 | B*55 | 0.03192 | B*55 | 0.03327 | ***B*50*** | ***0.0305*** |
| 14 | B*27 | 0.03169 | ***B*50*** | ***0.03000*** | B*55 | 0.0294 |
| **15** | **B*52** | **0.02974** | B*27 | 0.02773 | B*14 | 0.0289 |
| 16 | B*41 | 0.02375 | B*41 | 0.02542 | **B*52** | **0.0258** |
| 17 | B*14 | 0.02227 | B*14 | 0.02126 | B*41 | 0.0217 |
| 18 | B*57 | 0.01806 | B*58 | 0.01664 | B*58 | 0.0201 |
| 19 | B*39 | 0.01581 | B*39 | 0.01617 | B*57 | 0.0181 |
| 20 | B*58 | 0.01573 | B*57 | 0.01525 | B*39 | 0.0165 |
| 21 | B*37 | 0.01409 | B*37 | 0.01294 | B*37 | 0.0129 |
| 22 | B*48 | 0.00483 | B*56 | 0.00555 | B*48 | 0.0047 |
| 23 | B*53 | 0.00397 | B*48 | 0.00508 | B*53 | 0.0041 |
| 24 | B*56 | 0.00343 | B*53 | 0.00277 | B*54 | 0.0031 |
| 25 | B*45 | 0.00273 | B*45 | 0.00139 | B*56 | 0.0021 |
| 26 | B*54 | 0.00117 | B*54 | 0.00139 | B*42 | 0.0016 |
| 27 | B*47 | 0.00109 | B*73 | 0.00139 | B*46 | 0.0016 |
| 28 | B*46 | 0.00093 | B*46 | 0.00092 | B*45 | 0.0010 |
| 29 | B*73 | 0.00078 | B*47 | 0.00092 | B*04 | 0.0005 |
| 30 | B*59 | 0.00031 | B*62 | 0.00046 | B*19 | 0.0005 |
| 31 | B*83 | 0.00016 |  |  | B*78 | 0.0005 |
| 32 | B*32 | 0.00016 |  |  |  |  |
| 33 | B*03 | 0.00016 |  |  |  |  |
| 34 | B*82 | 0.00008 |  |  |  |  |
| 35 | B*66 | 0.00008 |  |  |  |  |
| 36 | B*60 | 0.00008 |  |  |  |  |
| 37 | B*42 | 0.00008 |  |  |  |  |
| 38 | B*34 | 0.00008 |  |  |  |  |
| 39 | B*24 | 0.00008 |  |  |  |  |
| 40 | B*19 | 0.00008 |  |  |  |  |
| 41 | B*04 | 0.00008 |  |  |  |  |

Abbreviations: HSCT, hematopoietic stem cell transplant

**Table S13.** Allele ranking for HLA–C loci.

|  | **Donor** | | **HSCT** | | **Solid transplant candidates** | |
| --- | --- | --- | --- | --- | --- | --- |
|  |  | **AF** |  | **AF** |  | **AF** |
| 1 | C*07 | 0.21314 | C*07 | 0.22425 | C*07 | 0.19981 |
| 2 | C*04 | 0.19315 | C*04 | 0.18304 | C*04 | 0.19223 |
| 3 | C*12 | 0.12513 | C*12 | 0.13154 | C*12 | 0.14205 |
| 4 | C*06 | 0.10462 | C*06 | 0.09667 | C*06 | 0.11837 |
| 5 | C*15 | 0.06906 | C*15 | 0.07132 | C*03 | 0.06723 |
| 6 | C*03 | 0.05633 | C*03 | 0.05388 | C*15 | 0.05871 |
| ***7*** | ***C*14*** | ***0.04076*** | **C*01** | **0.04437** | **C*01** | **0.04167** |
| ***8*** | ***C*16*** | ***0.03998*** | **C*02** | **0.04041** | ***C*14*** | ***0.04070*** |
| **9** | **C*01** | **0.03920** | ***C*14*** | ***0.03724*** | ***C*16*** | ***0.03600*** |
| 10 | C*08 | 0.03686 | C*08 | 0.03566 | **C*02** | **0.03314** |
| **11** | **C*02** | **0.03453** | ***C*16*** | ***0.03407*** | C*08 | 0.03220 |
| 12 | C*05 | 0.02466 | C*05 | 0.02536 | C*05 | 0.02273 |
| 13 | C*17 | 0.01765 | C*17 | 0.01902 | C*17 | 0.01042 |
| 14 | C*18 | 0.00441 | C*18 | 0.00317 | C*18 | 0.00473 |
| 15 | C*10 | 0.00026 |  |  |  |  |
| 16 | C*13 | 0.00026 |  |  |  |  |

Abbreviations: HSCT, hematopoietic stem cell transplant

**Table S14.** Allele ranking for HLA–DRB1 loci.

|  | **Donor** | | **HSCT** | | **Solid transplant candidates** | |
| --- | --- | --- | --- | --- | --- | --- |
|  |  | **AF** |  | **AF** |  | **AF** |
| 1 | DRB1*11 | 0.20998 | DRB1*11 | 0.2103 | DRB1*11 | 0.18957 |
| 2 | DRB1*04 | 0.14575 | DRB1*04 | 0.1520 | DRB1*04 | 0.16426 |
| 3 | DRB1*13 | 0.11437 | DRB1*13 | 0.1063 | DRB1*13 | 0.11777 |
| 4 | DRB1*15 | 0.09779 | DRB1*15 | 0.1012 | DRB1*15 | 0.10072 |
| 5 | DRB1*07 | 0.09335 | DRB1*07 | 0.0892 | **DRB1*03** | **0.08884** |
| **6** | **DRB1*03** | **0.08175** | **DRB1*03** | **0.0799** | DRB1*07 | 0.08729 |
| 7 | DRB1*01 | 0.07062 | DRB1*01 | 0.0693 | DRB1*01 | 0.06612 |
| 8 | DRB1*14 | 0.06330 | DRB1*14 | 0.0656 | DRB1*14 | 0.05992 |
| 9 | DRB1*16 | 0.04928 | DRB1*16 | 0.0508 | DRB1*16 | 0.04442 |
| 10 | DRB1*08 | 0.02359 | DRB1*08 | 0.0287 | DRB1*08 | 0.02841 |
| 11 | DRB1*10 | 0.02273 | DRB1*10 | 0.0217 | **DRB1*12** | **0.02324** |
| **12** | **DRB1*12** | **0.01510** | **DRB1*12** | **0.0148** | DRB1*10 | 0.02066 |
| 13 | DRB1*09 | 0.01230 | DRB1*09 | 0.0102 | DRB1*09 | 0.00878 |
| 14 | DRB1*02 | 0.00008 |  |  |  |  |

Abbreviations: HSCT, hematopoietic stem cell transplant

**Table S15.** Allele ranking for HLA–DQB1 loci.

|  | **Donor** | | **HSCT** | | **Solid transplant candidates** | |
| --- | --- | --- | --- | --- | --- | --- |
|  |  | **AF** |  | **AF** |  | **AF** |
| 1 | DQB1*03 | 0.4014 | DQB1*03 | 0.41930 | DQB1*03 | 0.42576 |
| 2 | DQB1*05 | 0.2136 | DQB1*05 | 0.21464 | DQB1*05 | 0.21834 |
| 3 | DQB1*06 | 0.1921 | DQB1*06 | 0.18220 | DQB1*06 | 0.17467 |
| 4 | DQB1*02 | 0.1657 | DQB1*02 | 0.15474 | DQB1*02 | 0.16157 |
| 5 | DQB1*04 | 0.0271 | DQB1*04 | 0.02912 | DQB1*04 | 0.01965 |

Abbreviations: HSCT, hematopoietic stem cell transplant

**Table S16.** Allele ranking for HLA–DPB1 loci.

|  | **Donor** | | **HSCT** | | **Solid transplant candidates** | |
| --- | --- | --- | --- | --- | --- | --- |
|  |  | **AF** |  | **AF** |  | **AF** |
| 1 | DPB1*04 | 0.55743 | DPB1*04 | 0.54251 | DPB1*04 | 0.53623 |
| 2 | DPB1*02 | 0.18159 | DPB1*02 | 0.20243 | DPB1*02 | 0.20652 |
| 3 | DPB1*03 | 0.08361 | DPB1*03 | 0.06984 | DPB1*03 | 0.08333 |
| ***4*** | ***DPB1*17*** | ***0.02208*** | ***DPB1*17*** | ***0.02230*** | **DPB1*13** | **0.03986** |
| **5** | **DPB1*13** | **0.02111** | **DPB1*09** | **0.01923** | ***DPB1*17*** | ***0.03620*** |
| 6 | DPB1*05 | 0.01774 | **DPB1*14** | **0.01721** | DPB1*05 | 0.01812 |
| ***7*** | ***DPB1*10*** | ***0.01605*** | **DPB1*01** | **0.01518** | **DPB1*14** | **0.01812** |
| **8** | **DPB1*09** | **0.01520** | DPB1*05 | 0.01518 | **DPB1*23** | **0.01087** |
| **9** | **DPB1*14** | **0.01436** | **DPB1*13** | **0.01518** | **DPB1*09** | **0.00725** |
| **10** | **DPB1*01** | **0.01267** | ***DPB1*10*** | ***0.01420*** | **DPB1*11** | **0.00725** |
| 11 | DPB1*15 | 0.01182 | DPB1*15 | 0.00911 | **DPB1*01** | **0.00362** |
| **12** | **DPB1*23** | **0.00507** | DPB1*104 | 0.00709 | DPB1*06 | 0.00362 |
| 13 | DPB1*26 | 0.00422 | DPB1*105 | 0.00607 | ***DPB1*10*** | ***0.00360*** |
| 14 | DPB1*105 | 0.00422 | **DPB1*23** | **0.00607** | DPB1*15 | 0.00362 |
| 15 | DPB1*19 | 0.00338 | DPB1*06 | 0.00405 | DPB1*105 | 0.00362 |
| **16** | **DPB1*11** | **0.00338** | DPB1*51 | 0.00405 | DPB1*131 | 0.00362 |
| 17 | DPB1*104 | 0.00338 | DPB1*47 | 0.00304 | DPB1*30 | 0.00362 |
| 18 | DPB1*06 | 0.00253 | DPB1*66 | 0.00304 | DPB1*45 | 0.00362 |
| 19 | DPB1*66 | 0.00169 | DPB1*08 | 0.00202 | DPB1*47 | 0.00362 |
| 20 | DPB1*51 | 0.00169 | **DPB1*11** | **0.00202** | DPB1*51 | 0.00362 |
| 21 | DPB1*47 | 0.00169 | DPB1*16 | 0.00202 |  |  |
| 22 | DPB1*18 | 0.00169 | DPB1*18 | 0.00202 |  |  |
| 23 | DPB1*131 | 0.00169 | DPB1*26 | 0.00202 |  |  |
| 24 | DPB1*91 | 0.00084 | DPB1*124 | 0.00101 |  |  |
| 25 | DPB1*84 | 0.00084 | DPB1*126 | 0.00101 |  |  |
| 26 | DPB1*70 | 0.00084 | DPB1*129 | 0.00101 |  |  |
| 27 | DPB1*61 | 0.00084 | DPB1*19 | 0.00101 |  |  |
| 28 | DPB1*503 | 0.00084 | DPB1*22 | 0.00101 |  |  |
| 29 | DPB1*45 | 0.00084 | DPB1*28 | 0.00101 |  |  |
| 30 | DPB1*36 | 0.00084 | DPB1*296 | 0.00101 |  |  |
| 31 | DPB1*22 | 0.00084 | DPB1*36 | 0.00101 |  |  |
| 32 | DPB1*189 | 0.00084 | DPB1*37 | 0.00101 |  |  |
| 33 | DPB1*16 | 0.00084 | DPB1*415 | 0.00101 |  |  |
| 34 | DPB1*129 | 0.00084 | DPB1*535 | 0.00101 |  |  |
| 35 | DPB1*126 | 0.00084 | DPB1*70 | 0.00101 |  |  |
| 36 | DPB1*08 | 0.00084 | DPB1*79 | 0.00101 |  |  |
| 37 |  |  | DPB1*91 | 0.00101 |  |  |

Abbreviations: HSCT, hematopoietic stem cell transplant

Supplement File 2. xls

**Sheet 1.** Haplotype analysis statics.

**Sheet 2.** Donors haplotype.

**Sheet 3.** HSCT candidates haplotype.

**Sheet 4.** Solid Tx candidates haplotype.

**Sheet 5.** Renal Tx candidates haplotype.

**Sheet 1.** Haplotype analysis statics.

| **Donor** | **A:B:C** | **A:B:DRB1** | **A:B:C:DRB1** | **A:B:C:DRB1:DQB1** | **DRB1:DQB1:DPB1** | **A:B:C:DRB1:DQB1:DPB1** |
| --- | --- | --- | --- | --- | --- | --- |
| Number of individuals: | 1926 | 6422 | 1926 | 553 | 587 | 442 |
| Unique phenotypes: | 1499 | * | 1645 | 507 | 378 | 411 |
| Unique genotypes: | 4921 | * | 10085 | 5198 | 1064 | 7108 |
| Number of haplotypes: | 2322 | 3669 | 9338 | 7743 | 506 | 12,160 |
| **HSCT** | **A:B:C** | **A:B:DRB1** | **A:B:C:DRB1** | **A:B:C:DRB1:DQB1** | **DRB1:DQB1:DPB1** | **A:B:C:DRB1:DQB1:DPB1** |
| Number of individuals: | 631 | 1082 | 631 | 494 | 494 | 388 |
| Unique phenotypes: | 598 | 1056 | 623 | 489 | 347 | 387 |
| Unique genotypes: | 2003 | 3511 | 3906 | 5298 | 981 | 6987 |
| Number of haplotypes: | 1436 | 2101 | 4848 | 7681 | 500 | 12,130 |
| **Solid Tx** | **A:B:C** | **A:B:DRB1** | **A:B:C:DRB1** | **A:B:C:DRB1:DQB1** | **DRB1:DQB1:DPB1** | **A:B:C:DRB1:DQB1:DPB1** |
| Number of individuals: | 528 | 968 | 528 | 226 | 137 | 134 |
| Unique phenotypes: | 503 | 929 | 517 | 224 | 120 | 134 |
| Unique genotypes: | 1693 | 3122 | 3264 | 2464 | 332 | 2519 |
| Number of haplotypes: | 1329 | 2114 | 4379 | 4161 | 225 | 4704 |
| *Tx: Transplantation, HSCT:Hematopoietic stem cell transplant* | | |  |  |  |  |

**Sheet 2.** Donors haplotype.

| **Name** | **Frequency** | **Name** | **Frequency** | **Name** | **Frequency** | **Name** | **Frequency** | **Name** | **Frequency** | **Name** | **Frequency** |
| --- | --- | --- | --- | --- | --- | --- | --- | --- | --- | --- | --- |
| A24~B35~C04 | 0.03432 | A24~B35~C04~DR11 | 0.0155 | A24~B35~C04~DR11~DQ03 | 0.0251 | A24~B35~C04~DR11~DQ03~DP04 | 0.02125 | DR11~DQ03~DP04 | 0.14238 | A24~B35~DR11 | 0.0173 |
| A02~B35~C04 | 0.03158 | A02~B35~C04~DR11 | 0.01278 | A02~B35~C04~DR11~DQ03 | 0.0232 | A02~B35~C04~DR11~DQ03~DP04 | 0.02021 | DR15~DQ06~DP04 | 0.05765 | A02~B51~DR11 | 0.0127 |
| A03~B35~C04 | 0.02599 | A23~B49~C07~DR11 | 0.01154 | A02~B35~C04~DR04~DQ03 | 0.01514 | A11~B52~C12~DR15~DQ06~DP04 | 0.01331 | DR13~DQ06~DP04 | 0.05546 | A02~B51~DR04 | 0.0113 |
| A23~B49~C07 | 0.02138 | A01~B08~C07~DR03 | 0.0104 | A23~B49~C07~DR11~DQ03 | 0.01266 | A23~B49~C07~DR11~DQ03~DP04 | 0.01188 | DR03~DQ02~DP04 | 0.05144 | A01~B08~DR03 | 0.0111 |
| A01~B35~C04 | 0.02097 | A02~B50~C06~DR07 | 0.00894 | A02~B50~C06~DR07~DQ02 | 0.01266 | A02~B35~C06~DR07~DQ02~DP02 | 0.01131 | DR04~DQ03~DP04 | 0.04962 | A01~B35~DR11 | 0.0100 |
| A11~B35~C04 | 0.02036 | A01~B35~C04~DR11 | 0.00856 | A03~B07~C07~DR15~DQ06 | 0.01197 | A02~B07~C07~DR15~DQ06~DP04 | 0.01018 | DR01~DQ05~DP04 | 0.04431 | A02~B18~DR11 | 0.0096 |
| A02~B51~C15 | 0.01599 | A30~B13~C06~DR07 | 0.00816 | A11~B52~C12~DR15~DQ06 | 0.01157 | A01~B35~C04~DR13~DQ06~DP04 | 0.01001 | DR14~DQ05~DP04 | 0.03576 | A02~B50~DR07 | 0.0094 |
| A02~B51~C14 | 0.01478 | A02~B07~C07~DR15 | 0.00813 | A01~B08~C07~DR03~DQ02 | 0.00979 | A02~B51~C15~DR11~DQ03~DP04 | 0.00908 | DR04~DQ03~DP02 | 0.03508 | A33~B14~DR01 | 0.0092 |
| A02~B18~C07 | 0.01382 | A11~B52~C12~DR15 | 0.00808 | A01~B35~C04~DR13~DQ06 | 0.00964 | A02~B35~C04~DR04~DQ03~DP02 | 0.00905 | DR13~DQ06~DP02 | 0.02754 | A03~B35~DR04 | 0.0087 |
| A26~B38~C12 | 0.01328 | A33~B14~C08~DR01 | 0.00741 | A02~B51~C15~DR11~DQ03 | 0.00943 | A26~B08~C07~DR03~DQ02~DP04 | 0.00905 | DR07~DQ02~DP04 | 0.02703 | A24~B18~DR11 | 0.0078 |
| A02~B50~C06 | 0.01313 | A02~B18~C07~DR11 | 0.00724 | A03~B35~C04~DR01~DQ05 | 0.00904 | A03~B35~C04~DR07~DQ02~DP04 | 0.00905 | DR16~DQ05~DP04 | 0.02578 | A02~B35~DR11 | 0.0077 |
| A03~B07~C07 | 0.01303 | A02~B35~C04~DR14 | 0.00688 | A02~B07~C07~DR15~DQ06 | 0.00904 | A03~B07~C07~DR15~DQ06~DP04 | 0.00792 | DR15~DQ06~DP02 | 0.02139 | A03~B35~DR11 | 0.0074 |
| A02~B07~C07 | 0.01283 | A02~B51~C14~DR11 | 0.00684 | A03~B08~C07~DR03~DQ02 | 0.00904 | A01~B08~C07~DR03~DQ02~DP04 | 0.00776 | DR11~DQ03~DP02 | 0.02102 | A11~B52~DR15 | 0.0068 |
| A32~B35~C04 | 0.0126 | A03~B07~C07~DR15 | 0.00682 | A02~B35~C06~DR07~DQ02 | 0.00814 | A24~B35~C04~DR11~DQ03~DP02 | 0.0067 | DR11~DQ03~DP03 | 0.0188 | A11~B35~DR01 | 0.0068 |
| A33~B14~C08 | 0.01181 | A03~B35~C04~DR01 | 0.00671 | A30~B13~C06~DR07~DQ02 | 0.00814 | A02~B51~C15~DR14~DQ05~DP02 | 0.00566 | DR07~DQ02~DP17 | 0.01675 | A24~B35~DR04 | 0.0068 |
| A02~B44~C05 | 0.01152 | A11~B35~C04~DR01 | 0.00651 | A24~B52~C12~DR15~DQ06 | 0.00813 | A01~B35~C06~DR11~DQ03~DP04 | 0.00566 | DR04~DQ03~DP03 | 0.0125 | A02~B07~DR15 | 0.0067 |
| A01~B08~C07 | 0.01118 | A03~B35~C04~DR15 | 0.00598 | A03~B35~C04~DR07~DQ02 | 0.00723 | A02~B35~C04~DR13~DQ06~DP02 | 0.00566 | DR03~DQ02~DP03 | 0.01183 | A26~B38~DR04 | 0.0064 |
| A30~B13~C06 | 0.0105 | A29~B14~C08~DR01 | 0.00596 | A33~B14~C08~DR01~DQ05 | 0.00723 | A25~B18~C12~DR15~DQ06~DP04 | 0.00566 | DR07~DQ02~DP02 | 0.01113 | A23~B49~DR11 | 0.0064 |
| A24~B07~C07 | 0.01007 | A26~B38~C12~DR04 | 0.00581 | A26~B08~C07~DR03~DQ02 | 0.00716 | A02~B44~C05~DR04~DQ03~DP04 | 0.00566 | DR03~DQ02~DP02 | 0.01022 | A02~B13~DR07 | 0.0063 |
| A01~B57~C06 | 0.01005 | A24~B13~C06~DR07 | 0.00581 | A02~B18~C07~DR11~DQ03 | 0.00687 | A33~B14~C08~DR01~DQ05~DP04 | 0.00566 | DR14~DQ05~DP02 | 0.00943 | A30~B13~DR07 | 0.0063 |
| A11~B52~C12 | 0.009 | A01~B51~C15~DR11 | 0.0058 | A01~B35~C04~DR11~DQ03 | 0.0067 | A02~B38~C12~DR11~DQ03~DP04 | 0.00566 | DR08~DQ03~DP04 | 0.00917 | A02~B51~DR13 | 0.0061 |
| A02~B38~C12 | 0.0087 | A24~B35~C04~DR04 | 0.0057 | A24~B49~C07~DR11~DQ03 | 0.00646 | A02~B18~C07~DR11~DQ03~DP04 | 0.00486 | DR07~DQ02~DP03 | 0.00879 | A32~B35~DR11 | 0.0060 |
| A01~B51~C15 | 0.00787 | A32~B35~C04~DR11 | 0.00568 | A02~B44~C05~DR04~DQ03 | 0.00633 | A11~B55~C01~DR13~DQ06~DP04 | 0.00452 | DR01~DQ05~DP02 | 0.00856 | A03~B44~DR04 | 0.0056 |
| A02~B41~C17 | 0.00744 | A01~B35~C04~DR13 | 0.00545 | A24~B38~C12~DR13~DQ06 | 0.00633 | A11~B35~C04~DR11~DQ03~DP04 | 0.00452 | DR13~DQ03~DP04 | 0.00837 | A02~B38~DR13 | 0.0052 |
| A02~B13~C06 | 0.00707 | A02~B51~C15~DR04 | 0.00543 | A02~B35~C04~DR13~DQ06 | 0.00614 | A02~B51~C07~DR13~DQ06~DP04 | 0.00452 | DR16~DQ05~DP02 | 0.00811 | A02~B35~DR14 | 0.0050 |
| A03~B08~C07 | 0.00703 | A03~B08~C07~DR03 | 0.00519 | A02~B51~C07~DR13~DQ06 | 0.00542 | A24~B51~C14~DR04~DQ03~DP04 | 0.00452 | DR11~DQ03~DP13 | 0.00764 | A24~B08~DR03 | 0.0048 |
| A02~B51~C16 | 0.00695 | A02~B08~C07~DR03 | 0.00507 | A24~B13~C06~DR07~DQ02 | 0.00542 | A29~B07~C15~DR13~DQ03~DP02 | 0.00452 | DR10~DQ05~DP04 | 0.00752 | A24~B35~DR14 | 0.0047 |
| A02~B39~C12 | 0.00685 | A03~B52~C12~DR15 | 0.00501 | A02~B41~C17~DR11~DQ03 | 0.00542 | A26~B51~C15~DR11~DQ03~DP04 | 0.00452 | DR08~DQ04~DP04 | 0.00693 | A03~B35~DR01 | 0.0046 |
| A02~B15~C03 | 0.00648 | A02~B35~C04~DR04 | 0.00496 | A02~B51~C15~DR14~DQ05 | 0.00541 | A24~B35~C04~DR04~DQ03~DP04 | 0.00452 | DR11~DQ03~DP14 | 0.00644 | A02~B51~DR14 | 0.0046 |
| A24~B44~C05 | 0.00635 | A02~B13~C06~DR07 | 0.0047 | A03~B35~C04~DR13~DQ06 | 0.00529 | A03~B13~C06~DR01~DQ05~DP04 | 0.00452 | DR15~DQ06~DP03 | 0.00626 | A01~B35~DR13 | 0.0045 |
| A01~B37~C06 | 0.00629 | A02~B38~C12~DR13 | 0.00468 | A11~B35~C04~DR01~DQ05 | 0.00528 | A24~B18~C12~DR11~DQ03~DP04 | 0.00452 | DR12~DQ03~DP02 | 0.00613 | A03~B07~DR15 | 0.0043 |
| A01~B49~C07 | 0.00624 | A02~B44~C05~DR04 | 0.0045 | A24~B51~C15~DR15~DQ06 | 0.00519 | A24~B52~C12~DR15~DQ06~DP02 | 0.00452 | DR08~DQ04~DP03 | 0.0058 | A24~B35~DR13 | 0.0042 |
| A03~B18~C12 | 0.00607 | A24~B35~C04~DR13 | 0.00446 | A24~B18~C12~DR11~DQ03 | 0.00497 | A32~B13~C06~DR07~DQ02~DP17 | 0.00452 | DR07~DQ03~DP02 | 0.00577 | A11~B35~DR14 | 0.0042 |
| A03~B50~C06 | 0.00597 | A03~B35~C04~DR04 | 0.00437 | A32~B35~C04~DR11~DQ03 | 0.00461 | A01~B52~C12~DR15~DQ06~DP02 | 0.00452 | DR07~DQ03~DP04 | 0.00554 | A02~B35~DR04 | 0.0041 |
| A02~B27~C02 | 0.00597 | A24~B18~C12~DR11 | 0.00426 | A26~B38~C12~DR11~DQ03 | 0.00452 | A02~B41~C17~DR03~DQ02~DP03 | 0.00452 | DR11~DQ03~DP10 | 0.00543 | A02~B44~DR16 | 0.0040 |
| A02~B40~C03 | 0.00596 | A30~B35~C04~DR04 | 0.00407 | A24~B51~C14~DR04~DQ03 | 0.00452 | A02~B35~C12~DR11~DQ03~DP02 | 0.0045 | DR15~DQ05~DP04 | 0.0052 | A02~B08~DR03 | 0.0040 |
| A29~B14~C08 | 0.0059 | A03~B35~C04~DR11 | 0.00405 | A25~B18~C12~DR15~DQ06 | 0.00452 | A24~B49~C07~DR11~DQ03~DP03 | 0.00437 | DR09~DQ03~DP04 | 0.00511 | A24~B44~DR11 | 0.0039 |
| A24~B51~C14 | 0.00589 | A02~B35~C04~DR13 | 0.00394 | A01~B57~C06~DR07~DQ03 | 0.00452 | A02~B18~C07~DR01~DQ05~DP02 | 0.00419 | DR15~DQ06~DP01 | 0.00464 | A02~B44~DR11 | 0.0039 |
| A24~B18~C07 | 0.00565 | A24~B08~C07~DR03 | 0.00359 | A03~B15~C07~DR11~DQ03 | 0.00452 | A23~B49~C07~DR04~DQ03~DP04 | 0.00396 | DR15~DQ05~DP02 | 0.00445 | A03~B08~DR03 | 0.0039 |
| A24~B35~C12 | 0.00565 | A01~B57~C06~DR07 | 0.00347 | A24~B18~C07~DR01~DQ05 | 0.00398 | A02~B38~C12~DR13~DQ06~DP04 | 0.00339 | DR12~DQ03~DP04 | 0.0043 | A01~B51~DR11 | 0.0039 |
| A02~B08~C07 | 0.00546 | A32~B35~C04~DR04 | 0.00336 | A02~B35~C04~DR14~DQ05 | 0.00384 | A01~B57~C06~DR11~DQ03~DP04 | 0.00339 | DR16~DQ05~DP10 | 0.00423 | A25~B18~DR15 | 0.0039 |
| A24~B13~C06 | 0.00546 | A02~B35~C04~DR01 | 0.00333 | A24~B35~C04~DR04~DQ03 | 0.00378 | A02~B49~C07~DR01~DQ05~DP04 | 0.00339 | DR01~DQ05~DP09 | 0.00423 | A02~B44~DR04 | 0.0038 |
| A24~B55~C01 | 0.00545 | A02~B51~C15~DR11 | 0.00331 | A32~B52~C12~DR15~DQ06 | 0.00377 | A02~B07~C07~DR15~DQ05~DP04 | 0.00339 | DR15~DQ05~DP13 | 0.00417 | A24~B51~DR11 | 0.0036 |
| A24~B51~C15 | 0.00537 | A02~B51~C16~DR11 | 0.00316 | A11~B55~C01~DR13~DQ06 | 0.00362 | A01~B50~C06~DR07~DQ02~DP04 | 0.00339 | DR01~DQ05~DP03 | 0.00391 | A02~B41~DR03 | 0.0036 |
| A29~B07~C15 | 0.00534 | A24~B07~C07~DR15 | 0.00314 | A01~B40~C15~DR14~DQ05 | 0.00362 | A03~B08~C07~DR03~DQ02~DP03 | 0.00339 | DR04~DQ03~DP05 | 0.00375 | A02~B27~DR16 | 0.0036 |
| A24~B08~C07 | 0.00522 | A03~B35~C04~DR13 | 0.00313 | A01~B35~C06~DR11~DQ03 | 0.00362 | A03~B44~C16~DR04~DQ03~DP04 | 0.00339 | DR04~DQ04~DP04 | 0.00356 | A02~B51~DR16 | 0.0035 |
| A30~B35~C04 | 0.0051 | A11~B35~C04~DR11 | 0.00311 | A23~B49~C07~DR04~DQ03 | 0.00362 | A11~B13~C02~DR04~DQ03~DP04 | 0.00339 | DR10~DQ05~DP02 | 0.00341 | A02~B51~DR01 | 0.0035 |
| A03~B52~C12 | 0.00501 | A32~B52~C12~DR15 | 0.00311 | A01~B50~C06~DR07~DQ02 | 0.00362 | A02~B35~C04~DR14~DQ05~DP04 | 0.00339 | DR07~DQ03~DP13 | 0.00335 | A11~B35~DR04 | 0.0035 |
| A01~B51~C14 | 0.00497 | A02~B41~C17~DR13 | 0.00311 | A29~B07~C15~DR13~DQ03 | 0.00362 | A24~B35~C04~DR11~DQ03~DP03 | 0.00339 | DR14~DQ05~DP03 | 0.00306 | A02~B44~DR15 | 0.0035 |
| A02~B51~C07 | 0.00477 | A03~B44~C16~DR04 | 0.00307 | A03~B44~C16~DR04~DQ03 | 0.00362 | A02~B35~C04~DR01~DQ05~DP04 | 0.00339 | DR07~DQ02~DP05 | 0.00302 | A03~B18~DR11 | 0.0034 |
| A03~B51~C15 | 0.00474 | A24~B07~C07~DR11 | 0.00305 | A30~B13~C06~DR13~DQ06 | 0.00362 | A02~B50~C06~DR07~DQ02~DP03 | 0.00339 | DR13~DQ03~DP03 | 0.00302 | A02~B35~DR01 | 0.0033 |
| A24~B52~C12 | 0.00468 | A69~B18~C07~DR15 | 0.00304 | A33~B51~C14~DR11~DQ03 | 0.00362 | A03~B35~C04~DR13~DQ06~DP04 | 0.00339 | DR14~DQ05~DP05 | 0.00299 | A01~B35~DR04 | 0.0033 |
| A24~B40~C03 | 0.00441 | A24~B49~C07~DR11 | 0.00301 | A02~B49~C07~DR13~DQ06 | 0.00362 | A24~B40~C03~DR15~DQ06~DP04 | 0.00339 | DR13~DQ03~DP02 | 0.00293 | A01~B57~DR07 | 0.0033 |
| A32~B52~C12 | 0.00415 | A03~B50~C06~DR03 | 0.00289 | A01~B49~C07~DR14~DQ05 | 0.00362 | A03~B18~C07~DR16~DQ05~DP04 | 0.00339 | DR14~DQ05~DP26 | 0.00284 | A03~B55~DR14 | 0.0033 |
| A03~B15~C07 | 0.00411 | A24~B15~C07~DR13 | 0.00288 | A11~B49~C07~DR15~DQ06 | 0.00349 | A24~B50~C06~DR04~DQ03~DP02 | 0.00339 | DR11~DQ03~DP15 | 0.00262 | A24~B51~DR04 | 0.0032 |
| A26~B08~C07 | 0.00407 | A24~B44~C16~DR11 | 0.00284 | A02~B51~C16~DR11~DQ03 | 0.00317 | A02~B50~C06~DR07~DQ02~DP04 | 0.00339 | DR07~DQ03~DP09 | 0.00253 | A24~B35~DR01 | 0.0032 |
| A24~B44~C16 | 0.00402 | A24~B51~C15~DR11 | 0.00283 | A01~B51~C15~DR11~DQ03 | 0.00276 | A30~B13~C06~DR13~DQ06~DP02 | 0.00339 | DR10~DQ05~DP15 | 0.0025 | A02~B35~DR13 | 0.0031 |
| A01~B35~C06 | 0.004 | A25~B18~C12~DR15 | 0.0028 | A11~B44~C04~DR07~DQ02 | 0.00273 | A02~B27~C14~DR11~DQ03~DP13 | 0.00339 | DR01~DQ05~DP01 | 0.00239 | A02~B39~DR16 | 0.0031 |
| A01~B40~C15 | 0.00392 | A02~B39~C12~DR08 | 0.00278 | A02~B51~C14~DR04~DQ03 | 0.00272 | A24~B35~C04~DR08~DQ03~DP04 | 0.00339 | DR15~DQ06~DP15 | 0.00237 | A11~B35~DR11 | 0.0031 |
| A03~B35~C12 | 0.00386 | A03~B07~C07~DR11 | 0.00275 | A03~B13~C06~DR01~DQ05 | 0.00271 | A01~B15~C03~DR13~DQ06~DP04 | 0.0033 | DR04~DQ04~DP03 | 0.00232 | A24~B51~DR15 | 0.0031 |
| A02~B44~C07 | 0.00384 | A26~B55~C03~DR14 | 0.00274 | A01~B35~C04~DR13~DQ03 | 0.00271 | A32~B52~C12~DR15~DQ06~DP04 | 0.00253 | DR03~DQ02~DP01 | 0.00231 | A03~B51~DR04 | 0.0030 |
| A26~B55~C03 | 0.00383 | A02~B40~C03~DR13 | 0.00273 | A02~B35~C12~DR14~DQ05 | 0.00271 | A02~B35~C04~DR11~DQ03~DP03 | 0.00242 | DR11~DQ03~DP09 | 0.00226 | A24~B51~DR13 | 0.0030 |
| A26~B35~C04 | 0.00382 | A02~B44~C05~DR11 | 0.00272 | A01~B18~C12~DR14~DQ05 | 0.00271 | A24~B55~C01~DR13~DQ06~DP04 | 0.00226 | DR08~DQ03~DP03 | 0.00218 | A26~B38~DR11 | 0.0029 |
| A11~B49~C07 | 0.00371 | A03~B18~C12~DR11 | 0.00271 | A02~B49~C07~DR01~DQ05 | 0.00271 | A02~B08~C07~DR03~DQ02~DP04 | 0.00226 | DR01~DQ05~DP05 | 0.00214 | A23~B44~DR07 | 0.0029 |
| A11~B13~C06 | 0.00368 | A24~B52~C12~DR15 | 0.00264 | A11~B35~C04~DR11~DQ03 | 0.00271 | A02~B55~C01~DR11~DQ03~DP04 | 0.00226 | DR16~DQ05~DP09 | 0.0021 | A03~B35~DR13 | 0.0028 |
| A02~B58~C07 | 0.00357 | A26~B38~C12~DR11 | 0.00262 | A24~B44~C05~DR04~DQ03 | 0.00271 | A01~B18~C12~DR14~DQ05~DP04 | 0.00226 | DR13~DQ06~DP15 | 0.00189 | A26~B38~DR14 | 0.0028 |
| A03~B41~C17 | 0.00353 | A01~B40~C15~DR14 | 0.0026 | A11~B55~C03~DR16~DQ05 | 0.00271 | A01~B40~C15~DR14~DQ05~DP04 | 0.00226 | DR11~DQ06~DP04 | 0.00189 | A01~B13~DR07 | 0.0028 |
| A26~B55~C01 | 0.00348 | A11~B49~C07~DR11 | 0.00251 | A02~B44~C02~DR16~DQ05 | 0.00271 | A01~B35~C04~DR16~DQ05~DP04 | 0.00226 | DR11~DQ05~DP04 | 0.00186 | A02~B40~DR11 | 0.0027 |
| A02~B18~C12 | 0.00348 | A26~B38~C12~DR14 | 0.00248 | A30~B13~C06~DR11~DQ03 | 0.00271 | A26~B38~C12~DR03~DQ02~DP04 | 0.00226 | DR04~DQ03~DP14 | 0.00178 | A03~B35~DR07 | 0.0027 |
| A25~B18~C12 | 0.00336 | A32~B13~C06~DR07 | 0.00242 | A32~B13~C06~DR07~DQ02 | 0.00271 | A03~B55~C04~DR14~DQ05~DP09 | 0.00226 | DR01~DQ05~DP17 | 0.00175 | A03~B51~DR11 | 0.0027 |
| A68~B51~C15 | 0.00334 | A24~B35~C04~DR14 | 0.00241 | A11~B08~C07~DR03~DQ02 | 0.00271 | A02~B51~C16~DR11~DQ03~DP04 | 0.00226 | DR04~DQ03~DP13 | 0.00174 | A24~B07~DR15 | 0.0027 |
| A68~B35~C04 | 0.00332 | A24~B07~C07~DR04 | 0.00239 | A26~B27~C01~DR01~DQ05 | 0.00271 | A24~B44~C16~DR04~DQ03~DP04 | 0.00226 | DR13~DQ05~DP02 | 0.00172 | A24~B49~DR11 | 0.0027 |
| A69~B18~C07 | 0.0033 | A33~B14~C08~DR11 | 0.00235 | A11~B13~C02~DR04~DQ03 | 0.00271 | A23~B49~C07~DR14~DQ05~DP02 | 0.00226 | DR07~DQ03~DP03 | 0.00171 | A03~B35~DR14 | 0.0026 |
| A32~B18~C12 | 0.00323 | A02~B41~C17~DR03 | 0.00234 | A02~B38~C12~DR11~DQ03 | 0.00271 | A30~B13~C06~DR07~DQ05~DP04 | 0.00226 | DR03~DQ03~DP04 | 0.00171 | A26~B38~DR13 | 0.0026 |
| A29~B35~C04 | 0.00322 | A26~B35~C04~DR08 | 0.00234 | A01~B15~C03~DR13~DQ06 | 0.00271 | A02~B44~C02~DR16~DQ05~DP04 | 0.00226 | DR15~DQ06~DP19 | 0.0017 | A03~B52~DR15 | 0.0025 |
| A02~B57~C06 | 0.00322 | A68~B35~C04~DR04 | 0.00231 | A23~B49~C07~DR15~DQ05 | 0.00271 | A03~B51~C16~DR01~DQ05~DP04 | 0.00226 | DR15~DQ05~DP03 | 0.0017 | A26~B08~DR03 | 0.0025 |
| A24~B18~C12 | 0.00317 | A02~B07~C07~DR04 | 0.00231 | A68~B51~C15~DR07~DQ02 | 0.00271 | A02~B18~C07~DR16~DQ05~DP13 | 0.00226 | DR10~DQ05~DP14 | 0.0017 | A03~B35~DR15 | 0.0024 |
| A02~B44~C16 | 0.00314 | A11~B50~C06~DR07 | 0.00228 | A68~B44~C16~DR04~DQ03 | 0.00271 | A01~B40~C15~DR14~DQ05~DP02 | 0.00226 | DR04~DQ04~DP05 | 0.0017 | A26~B35~DR11 | 0.0024 |
| A11~B50~C06 | 0.00313 | A01~B44~C05~DR11 | 0.00228 | A24~B44~C02~DR16~DQ05 | 0.00271 | A24~B13~C06~DR07~DQ02~DP17 | 0.00226 | DR15~DQ06~DP23 | 0.0017 | A24~B35~DR15 | 0.0024 |
| A01~B15~C07 | 0.00305 | A03~B35~C12~DR04 | 0.00228 | A24~B40~C03~DR15~DQ06 | 0.00271 | A24~B35~C06~DR11~DQ03~DP04 | 0.00226 | DR03~DQ02~DP66 | 0.0017 | A11~B35~DR15 | 0.0024 |
| A30~B49~C07 | 0.00305 | A32~B18~C12~DR11 | 0.00227 | A33~B58~C03~DR03~DQ02 | 0.00271 | A02~B58~C07~DR08~DQ04~DP04 | 0.00226 | DR16~DQ05~DP23 | 0.0017 | A01~B37~DR10 | 0.0024 |
| A24~B49~C07 | 0.00288 | A11~B08~C07~DR03 | 0.00227 | A24~B13~C06~DR03~DQ02 | 0.00271 | A03~B35~C04~DR04~DQ03~DP04 | 0.00226 | DR01~DQ05~DP10 | 0.0017 | A32~B35~DR04 | 0.0024 |
| A29~B58~C07 | 0.00288 | A24~B51~C14~DR15 | 0.00227 | A02~B27~C14~DR03~DQ02 | 0.00271 | A01~B18~C12~DR16~DQ05~DP04 | 0.00226 | DR04~DQ03~DP47 | 0.0017 | A01~B15~DR13 | 0.0024 |
| A33~B58~C03 | 0.00285 | A24~B18~C07~DR04 | 0.00225 | A01~B41~C17~DR07~DQ03 | 0.00271 | A03~B08~C07~DR03~DQ02~DP04 | 0.00226 | DR09~DQ03~DP10 | 0.0017 | A02~B41~DR13 | 0.0023 |
| A01~B52~C12 | 0.00285 | A11~B35~C04~DR08 | 0.00222 | A24~B35~C04~DR08~DQ03 | 0.00271 | A24~B27~C01~DR03~DQ02~DP04 | 0.00226 | DR07~DQ02~DP105 | 0.0017 | A11~B08~DR03 | 0.0023 |
| A23~B44~C04 | 0.00285 | A30~B51~C16~DR13 | 0.00215 | A02~B38~C12~DR13~DQ06 | 0.00271 | A24~B48~C08~DR04~DQ03~DP02 | 0.00226 | DR11~DQ03~DP104 | 0.0017 | A24~B55~DR13 | 0.0023 |
| A02~B15~C01 | 0.00284 | A24~B35~C04~DR01 | 0.00215 | A24~B14~C08~DR01~DQ05 | 0.00271 | A30~B13~C06~DR07~DQ02~DP05 | 0.00226 | DR14~DQ05~DP17 | 0.00161 | A24~B55~DR14 | 0.0023 |
| A32~B38~C12 | 0.00275 | A23~B49~C07~DR15 | 0.00208 | A02~B35~C03~DR03~DQ02 | 0.00271 | A03~B51~C12~DR04~DQ03~DP02 | 0.00226 | DR14~DQ03~DP04 | 0.00159 | A02~B40~DR15 | 0.0023 |
| A01~B07~C07 | 0.00275 | A01~B37~C06~DR16 | 0.00208 | A11~B08~C03~DR03~DQ02 | 0.00271 | A11~B35~C04~DR08~DQ04~DP04 | 0.00226 | DR07~DQ02~DP14 | 0.00159 | A68~B51~DR11 | 0.0022 |
| A24~B15~C03 | 0.00273 | A02~B58~C07~DR11 | 0.00208 | A02~B46~C01~DR08~DQ06 | 0.00271 | A30~B35~C04~DR04~DQ03~DP47 | 0.00226 | DR10~DQ05~DP03 | 0.00159 | A02~B27~DR04 | 0.0022 |
| A02~B35~C12 | 0.00268 | A68~B53~C04~DR13 | 0.00208 | A68~B35~C04~DR14~DQ05 | 0.00271 | A30~B53~C04~DR15~DQ06~DP04 | 0.00226 | DR07~DQ02~DP15 | 0.00149 | A29~B14~DR01 | 0.0022 |
| A11~B40~C03 | 0.00263 | A30~B27~C02~DR13 | 0.00206 | A03~B38~C12~DR13~DQ06 | 0.00271 | A26~B15~C07~DR13~DQ03~DP02 | 0.00226 | DR08~DQ04~DP02 | 0.00147 | A24~B44~DR04 | 0.0021 |
| A24~B55~C03 | 0.0026 | A01~B35~C04~DR15 | 0.00206 | A02~B51~C15~DR04~DQ03 | 0.00271 | A02~B44~C04~DR07~DQ02~DP02 | 0.00226 | DR04~DQ04~DP26 | 0.00142 | A02~B35~DR15 | 0.0021 |
| A31~B15~C01 | 0.00255 | A24~B51~C15~DR04 | 0.00205 | A23~B44~C04~DR07~DQ02 | 0.0027 | A11~B38~C12~DR04~DQ03~DP02 | 0.00226 | DR10~DQ05~DP05 | 0.0014 | A02~B41~DR04 | 0.0021 |
| A32~B51~C15 | 0.00253 | A23~B44~C04~DR07 | 0.00205 | A03~B51~C16~DR16~DQ05 | 0.00269 | A32~B35~C04~DR15~DQ06~DP04 | 0.00226 | DR07~DQ02~DP11 | 0.00135 | A68~B44~DR04 | 0.0021 |
| A03~B44~C16 | 0.00248 | A02~B51~C14~DR04 | 0.00204 | A32~B51~C16~DR11~DQ03 | 0.00262 | A24~B52~C12~DR15~DQ06~DP04 | 0.00226 | DR14~DQ03~DP02 | 0.00129 | A01~B18~DR11 | 0.0021 |
| A24~B48~C08 | 0.00242 | A01~B57~C06~DR04 | 0.00204 | A24~B35~C04~DR14~DQ05 | 0.00248 | A24~B07~C07~DR11~DQ03~DP04 | 0.00226 | DR11~DQ03~DP17 | 0.00129 | A32~B44~DR11 | 0.0021 |
| A02~B39~C07 | 0.00241 | A02~B35~C12~DR14 | 0.00203 | A24~B27~C02~DR11~DQ03 | 0.00239 | A33~B35~C04~DR11~DQ03~DP04 | 0.00226 | DR10~DQ05~DP10 | 0.00129 | A11~B51~DR04 | 0.0021 |
| A24~B07~C15 | 0.00235 | A32~B08~C07~DR03 | 0.00202 | A24~B35~C04~DR01~DQ05 | 0.00234 | A02~B49~C07~DR10~DQ05~DP04 | 0.00226 | DR04~DQ03~DP11 | 0.00121 | A02~B15~DR04 | 0.0021 |
| A11~B08~C07 | 0.00234 | A02~B41~C17~DR04 | 0.00202 | A68~B35~C04~DR04~DQ03 | 0.00225 | A23~B49~C07~DR15~DQ05~DP03 | 0.00226 | DR15~DQ03~DP04 | 0.00117 | A29~B35~DR11 | 0.0021 |
| A11~B51~C04 | 0.00231 | A30~B13~C06~DR13 | 0.00201 | A01~B52~C12~DR15~DQ06 | 0.00202 | A33~B52~C12~DR15~DQ06~DP02 | 0.00226 | DR04~DQ04~DP02 | 0.00111 | A24~B27~DR11 | 0.0020 |
| A68~B53~C04 | 0.00231 | A03~B51~C15~DR04 | 0.00201 | A03~B18~C07~DR16~DQ05 | 0.00194 | A02~B57~C06~DR13~DQ06~DP04 | 0.00226 | DR07~DQ02~DP09 | 0.00111 | A02~B44~DR07 | 0.0020 |
| A11~B51~C15 | 0.0023 | A24~B07~C15~DR13 | 0.002 | A01~B35~C04~DR04~DQ03 | 0.00194 | A26~B35~C12~DR04~DQ03~DP04 | 0.00226 | DR11~DQ03~DP06 | 0.00104 | A24~B52~DR15 | 0.0020 |
| A02~B15~C07 | 0.0023 | A01~B15~C07~DR13 | 0.00199 | A03~B35~C04~DR11~DQ03 | 0.00191 | A33~B44~C05~DR07~DQ03~DP02 | 0.00226 | DR10~DQ05~DP09 | 0.00104 | A30~B35~DR04 | 0.0020 |
| A32~B13~C06 | 0.00228 | A02~B27~C02~DR16 | 0.00197 | A02~B40~C03~DR13~DQ06 | 0.0019 | A30~B08~C07~DR11~DQ03~DP04 | 0.00226 | DR14~DQ05~DP10 | 0.00097 | A02~B49~DR13 | 0.0020 |
| A01~B40~C03 | 0.00226 | A02~B51~C14~DR01 | 0.00196 | A02~B08~C07~DR03~DQ02 | 0.00186 | A01~B58~C04~DR04~DQ03~DP04 | 0.00226 | DR15~DQ06~DP05 | 0.00097 | A01~B52~DR15 | 0.0020 |
| A24~B51~C16 | 0.00222 | A26~B55~C01~DR03 | 0.00195 | A24~B07~C07~DR11~DQ03 | 0.00181 | A03~B18~C12~DR11~DQ03~DP13 | 0.00226 | DR14~DQ02~DP04 | 0.00093 | A32~B52~DR15 | 0.0020 |
| A30~B27~C02 | 0.00221 | A24~B51~C15~DR15 | 0.00195 | A11~B35~C04~DR04~DQ03 | 0.00181 | A03~B55~C01~DR11~DQ03~DP04 | 0.00226 | DR07~DQ02~DP13 | 0.0009 | A01~B57~DR11 | 0.0019 |
| A02~B35~C03 | 0.0022 | A02~B18~C07~DR01 | 0.00193 | A03~B51~C07~DR16~DQ05 | 0.00181 | A33~B58~C03~DR03~DQ03~DP04 | 0.00226 | DR04~DQ02~DP04 | 0.00088 | A02~B15~DR13 | 0.0019 |
| A30~B51~C16 | 0.00211 | A29~B35~C04~DR11 | 0.00192 | A02~B44~C06~DR11~DQ03 | 0.00181 | A26~B15~C07~DR08~DQ03~DP03 | 0.00226 | DR04~DQ06~DP03 | 0.00087 | A29~B07~DR10 | 0.0019 |
| A32~B08~C07 | 0.00207 | A02~B44~C07~DR16 | 0.00191 | A02~B39~C12~DR16~DQ05 | 0.00181 | A24~B07~C07~DR10~DQ05~DP04 | 0.00226 | DR13~DQ03~DP09 | 0.00086 | A32~B51~DR04 | 0.0019 |
| A03~B40~C03 | 0.00207 | A02~B15~C03~DR13 | 0.0019 | A01~B35~C04~DR16~DQ05 | 0.00181 | A68~B52~C12~DR15~DQ06~DP04 | 0.00226 | DR07~DQ06~DP13 | 0.00086 | A02~B27~DR11 | 0.0019 |
| A01~B44~C05 | 0.00206 | A02~B51~C15~DR14 | 0.00186 | A26~B38~C12~DR03~DQ02 | 0.00181 | A02~B15~C03~DR07~DQ02~DP17 | 0.00226 | DR16~DQ03~DP04 | 0.00086 | A11~B44~DR07 | 0.0019 |
| A32~B27~C02 | 0.00203 | A02~B44~C16~DR04 | 0.00184 | A02~B41~C07~DR03~DQ02 | 0.00181 | A01~B57~C06~DR07~DQ03~DP04 | 0.00226 | DR11~DQ02~DP02 | 0.00085 | A02~B51~DR09 | 0.0019 |
| A02~B49~C07 | 0.00203 | A03~B13~C06~DR01 | 0.00182 | A68~B51~C14~DR14~DQ05 | 0.00181 | A11~B15~C03~DR07~DQ03~DP04 | 0.00226 | DR08~DQ05~DP04 | 0.00085 | A24~B51~DR14 | 0.0019 |
| A03~B13~C06 | 0.00196 | A03~B15~C07~DR11 | 0.00182 | A02~B07~C07~DR15~DQ05 | 0.00181 | A11~B49~C07~DR15~DQ06~DP04 | 0.00226 | DR15~DQ06~DP91 | 0.00085 | A32~B38~DR11 | 0.0019 |
| A24~B38~C12 | 0.00195 | A01~B40~C03~DR04 | 0.00182 | A01~B57~C06~DR13~DQ03 | 0.00181 | A24~B40~C03~DR13~DQ06~DP04 | 0.00226 | DR08~DQ03~DP14 | 0.00085 | A30~B49~DR13 | 0.0019 |
| A23~B51~C16 | 0.00195 | A24~B35~C12~DR15 | 0.00181 | A01~B35~C06~DR07~DQ02 | 0.00181 | A32~B38~C12~DR14~DQ05~DP04 | 0.00226 | DR10~DQ05~DP01 | 0.00085 | A03~B50~DR07 | 0.0019 |
| A02~B40~C15 | 0.00193 | A24~B27~C02~DR11 | 0.0018 | A02~B51~C16~DR13~DQ06 | 0.00181 | A23~B35~C04~DR13~DQ06~DP15 | 0.00226 | DR13~DQ06~DP18 | 0.00085 | A02~B40~DR13 | 0.0018 |
| A01~B41~C17 | 0.00192 | A02~B50~C06~DR11 | 0.00177 | A03~B51~C01~DR01~DQ05 | 0.00181 | A24~B48~C08~DR08~DQ04~DP03 | 0.00226 | DR15~DQ06~DP36 | 0.00085 | A26~B51~DR11 | 0.0018 |
| A32~B49~C07 | 0.0019 | A03~B35~C04~DR07 | 0.00176 | A02~B44~C05~DR11~DQ03 | 0.00181 | A01~B51~C15~DR11~DQ03~DP14 | 0.00226 | DR04~DQ06~DP18 | 0.00085 | A11~B35~DR13 | 0.0018 |
| A11~B38~C12 | 0.00189 | A03~B41~C17~DR04 | 0.00175 | A01~B07~C07~DR15~DQ05 | 0.00181 | A31~B52~C12~DR14~DQ05~DP26 | 0.00226 | DR11~DQ03~DP61 | 0.00085 | A23~B50~DR03 | 0.0018 |
| A30~B08~C07 | 0.00189 | A68~B50~C06~DR07 | 0.00174 | A02~B07~C07~DR11~DQ03 | 0.00181 | A24~B35~C12~DR13~DQ06~DP04 | 0.00226 | DR11~DQ04~DP08 | 0.00085 | A03~B07~DR04 | 0.0018 |
| A26~B51~C14 | 0.00189 | A11~B44~C04~DR07 | 0.00174 | A26~B35~C04~DR11~DQ03 | 0.00181 | A02~B51~C07~DR13~DQ06~DP02 | 0.00226 | DR04~DQ03~DP01 | 0.00085 | A33~B58~DR03 | 0.0018 |
| A02~B51~C04 | 0.00189 | A02~B18~C12~DR11 | 0.00174 | A68~B51~C15~DR11~DQ03 | 0.00181 | A02~B40~C03~DR09~DQ03~DP04 | 0.00226 | DR16~DQ05~DP22 | 0.00085 | A24~B40~DR11 | 0.0018 |
| A24~B37~C06 | 0.00184 | A24~B55~C01~DR14 | 0.00172 | A26~B51~C14~DR15~DQ06 | 0.00181 | A03~B07~C07~DR15~DQ05~DP13 | 0.00226 | DR13~DQ06~DP11 | 0.00085 | A02~B40~DR04 | 0.0018 |
| A30~B38~C12 | 0.00181 | A02~B18~C07~DR04 | 0.00172 | A29~B58~C07~DR08~DQ04 | 0.00181 | A29~B07~C07~DR15~DQ03~DP04 | 0.00226 | DR14~DQ05~DP01 | 0.00085 | A03~B50~DR03 | 0.0018 |
| A26~B07~C07 | 0.00181 | A31~B15~C01~DR04 | 0.00171 | A24~B18~C07~DR04~DQ03 | 0.00181 | A01~B51~C06~DR07~DQ02~DP03 | 0.00226 | DR15~DQ04~DP02 | 0.00085 | A24~B40~DR04 | 0.0018 |
| A03~B14~C08 | 0.00176 | A01~B49~C07~DR13 | 0.0017 | A32~B49~C07~DR16~DQ05 | 0.00181 | A24~B51~C15~DR15~DQ06~DP04 | 0.00226 | DR08~DQ03~DP70 | 0.00085 | A11~B49~DR11 | 0.0018 |
| A32~B51~C14 | 0.00171 | A02~B51~C04~DR04 | 0.00169 | A31~B44~C05~DR04~DQ03 | 0.00181 | A02~B51~C15~DR14~DQ05~DP04 | 0.00224 | DR04~DQ05~DP105 | 0.00085 | A66~B41~DR13 | 0.0018 |
| A02~B14~C08 | 0.00169 | A23~B49~C07~DR13 | 0.00169 | A32~B18~C12~DR11~DQ03 | 0.00181 | A11~B08~C03~DR03~DQ02~DP03 | 0.00211 | DR16~DQ05~DP51 | 0.00085 | A01~B50~DR07 | 0.0018 |
| A26~B15~C07 | 0.00168 | A24~B55~C01~DR13 | 0.00168 | A24~B50~C06~DR03~DQ02 | 0.00181 | A02~B41~C17~DR11~DQ03~DP04 | 0.0017 | DR13~DQ03~DP105 | 0.00085 | A01~B40~DR14 | 0.0018 |
| A24~B35~C07 | 0.00168 | A11~B35~C04~DR10 | 0.00168 | A24~B48~C08~DR04~DQ03 | 0.00181 | A24~B35~C04~DR01~DQ05~DP02 | 0.00147 | DR07~DQ02~DP51 | 0.00085 | A26~B15~DR13 | 0.0018 |
| A01~B14~C08 | 0.00168 | A02~B51~C15~DR09 | 0.00167 | A26~B39~C12~DR04~DQ04 | 0.00181 | A11~B51~C14~DR03~DQ02~DP04 | 0.0014 | DR03~DQ06~DP10 | 0.00085 | A26~B51~DR04 | 0.0017 |
| A11~B55~C01 | 0.00167 | A01~B57~C06~DR13 | 0.00167 | A03~B44~C04~DR07~DQ03 | 0.00181 | A01~B35~C04~DR13~DQ03~DP04 | 0.0013 | DR13~DQ03~DP14 | 0.00085 | A24~B13~DR07 | 0.0017 |
| A01~B55~C01 | 0.00166 | A02~B44~C07~DR11 | 0.00165 | A11~B35~C04~DR08~DQ04 | 0.00181 | A03~B07~C07~DR15~DQ06~DP01 | 0.0013 | DR13~DQ06~DP16 | 0.00085 | A02~B51~DR15 | 0.0017 |
| A33~B48~C08 | 0.00166 | A24~B35~C04~DR15 | 0.0016 | A32~B38~C12~DR11~DQ03 | 0.00181 | A01~B49~C07~DR11~DQ03~DP04 | 0.00129 | DR09~DQ03~DP13 | 0.00085 | A11~B51~DR15 | 0.0017 |
| A24~B44~C02 | 0.00165 | A02~B39~C12~DR16 | 0.00156 | A30~B53~C04~DR15~DQ06 | 0.00181 | A24~B08~C07~DR03~DQ02~DP03 | 0.00129 | DR07~DQ03~DP129 | 0.00085 | A03~B07~DR11 | 0.0017 |
| A68~B40~C03 | 0.00165 | A02~B51~C07~DR13 | 0.00156 | A26~B15~C07~DR13~DQ03 | 0.00181 | A11~B08~C03~DR03~DQ02~DP04 | 0.00128 | DR16~DQ05~DP503 | 0.00085 | A68~B35~DR13 | 0.0017 |
| A66~B41~C17 | 0.00163 | A11~B55~C03~DR16 | 0.00156 | A01~B51~C14~DR13~DQ06 | 0.00181 | A02~B51~C15~DR16~DQ05~DP04 | 0.00113 | DR16~DQ03~DP05 | 0.00085 | A68~B35~DR11 | 0.0017 |
| A23~B50~C06 | 0.00163 | A29~B58~C07~DR08 | 0.00156 | A02~B44~C04~DR07~DQ02 | 0.00181 | A02~B39~C12~DR16~DQ05~DP04 | 0.00113 | DR12~DQ03~DP23 | 0.00085 | A02~B35~DR16 | 0.0017 |
| A33~B35~C07 | 0.00162 | A24~B38~C12~DR13 | 0.00156 | A29~B49~C07~DR11~DQ03 | 0.00181 | A24~B44~C04~DR07~DQ03~DP03 | 0.00113 | DR10~DQ05~DP105 | 0.00085 | A24~B07~DR11 | 0.0017 |
| A29~B45~C06 | 0.00156 | A68~B07~C07~DR15 | 0.00156 | A11~B38~C12~DR04~DQ03 | 0.00181 | A01~B45~C16~DR04~DQ03~DP03 | 0.00113 | DR03~DQ02~DP23 | 0.00085 | A32~B27~DR11 | 0.0017 |
| A01~B35~C12 | 0.00155 | A02~B35~C03~DR03 | 0.00156 | A32~B35~C04~DR15~DQ06 | 0.00181 | A02~B35~C06~DR07~DQ02~DP04 | 0.00113 | DR07~DQ02~DP126 | 0.00085 | A02~B15~DR08 | 0.0017 |
| A29~B57~C18 | 0.00152 | A29~B57~C18~DR01 | 0.00156 | A02~B35~C04~DR03~DQ02 | 0.00181 | A24~B13~C14~DR04~DQ04~DP14 | 0.00113 | DR07~DQ02~DP45 | 0.00085 | A32~B08~DR03 | 0.0017 |
| A26~B35~C12 | 0.00151 | A29~B45~C06~DR11 | 0.00156 | A11~B51~C15~DR04~DQ03 | 0.00181 | A32~B55~C01~DR15~DQ06~DP04 | 0.00113 | DR08~DQ04~DP131 | 0.00085 | A24~B35~DR12 | 0.0017 |
| A03~B51~C01 | 0.00149 | A68~B35~C02~DR14 | 0.00156 | A02~B41~C06~DR03~DQ02 | 0.00181 | A26~B07~C12~DR15~DQ05~DP13 | 0.00113 | DR07~DQ02~DP104 | 0.00085 | A26~B44~DR04 | 0.0016 |
| A01~B18~C07 | 0.00149 | A29~B58~C07~DR04 | 0.00156 | A30~B40~C03~DR04~DQ03 | 0.00181 | A01~B57~C06~DR15~DQ06~DP36 | 0.00113 | DR07~DQ03~DP131 | 0.00085 | A32~B18~DR11 | 0.0016 |
| A03~B44~C12 | 0.00149 | A02~B35~C06~DR11 | 0.00155 | A03~B07~C07~DR03~DQ02 | 0.00181 | A03~B37~C04~DR16~DQ05~DP04 | 0.00113 | DR03~DQ02~DP104 | 0.00085 | A02~B44~DR01 | 0.0016 |
| A68~B39~C12 | 0.00146 | A02~B57~C06~DR07 | 0.00155 | A02~B57~C06~DR13~DQ06 | 0.00181 | A03~B35~C04~DR11~DQ05~DP04 | 0.00113 | DR04~DQ03~DP06 | 0.00085 | A02~B35~DR03 | 0.0016 |
| A32~B35~C03 | 0.00146 | A24~B07~C07~DR03 | 0.00154 | A26~B35~C12~DR04~DQ03 | 0.00181 | A24~B14~C14~DR04~DQ06~DP18 | 0.00113 | DR12~DQ03~DP189 | 0.00085 | A02~B57~DR07 | 0.0016 |
| A32~B40~C02 | 0.00146 | A26~B08~C07~DR03 | 0.00154 | A33~B44~C05~DR07~DQ03 | 0.00181 | A66~B55~C17~DR13~DQ06~DP02 | 0.00113 | DR07~DQ03~DP17 | 0.0008 | A01~B51~DR13 | 0.0016 |
| A03~B27~C02 | 0.00145 | A02~B18~C07~DR15 | 0.00154 | A02~B35~C12~DR04~DQ03 | 0.00181 | A69~B35~C06~DR11~DQ03~DP61 | 0.00113 | DR15~DQ06~DP17 | 0.0008 | A01~B57~DR13 | 0.0015 |
| A26~B49~C07 | 0.00144 | A02~B15~C07~DR16 | 0.00154 | A02~B27~C02~DR10~DQ05 | 0.00181 | A74~B50~C04~DR07~DQ02~DP05 | 0.00113 | DR11~DQ04~DP09 | 0.00078 | A01~B35~DR01 | 0.0015 |
| A11~B35~C12 | 0.00143 | A02~B35~C04~DR10 | 0.00153 | A01~B58~C04~DR04~DQ03 | 0.00181 | A23~B44~C05~DR01~DQ05~DP04 | 0.00113 | DR11~DQ06~DP01 | 0.0007 | A02~B07~DR01 | 0.0015 |
| A24~B44~C07 | 0.00142 | A24~B51~C16~DR04 | 0.00153 | A03~B55~C01~DR11~DQ03 | 0.00181 | A26~B38~C12~DR11~DQ03~DP04 | 0.00113 | DR13~DQ06~DP13 | 0.00067 | A32~B51~DR11 | 0.0015 |
| A32~B39~C12 | 0.00142 | A32~B49~C07~DR13 | 0.00153 | A26~B15~C07~DR08~DQ03 | 0.00181 | A02~B41~C07~DR14~DQ02~DP04 | 0.00113 | DR16~DQ05~DP06 | 0.00066 | A02~B35~DR08 | 0.0015 |
| A30~B35~C06 | 0.00141 | A23~B50~C06~DR03 | 0.00152 | A24~B07~C07~DR10~DQ05 | 0.00181 | A68~B51~C14~DR03~DQ05~DP04 | 0.00113 | DR04~DQ04~DP13 | 0.00066 | A01~B37~DR11 | 0.0015 |
| A24~B44~C04 | 0.00139 | A23~B49~C07~DR01 | 0.00152 | A26~B51~C12~DR13~DQ06 | 0.00181 | A03~B51~C07~DR15~DQ05~DP04 | 0.00113 | DR12~DQ03~DP05 | 0.00064 | A24~B44~DR16 | 0.0015 |
| A68~B44~C14 | 0.00139 | A02~B38~C12~DR14 | 0.00149 | A11~B35~C04~DR16~DQ05 | 0.00181 | A68~B44~C04~DR03~DQ02~DP02 | 0.00113 | DR04~DQ04~DP14 | 0.00064 | A02~B39~DR09 | 0.0015 |
| A24~B14~C08 | 0.00137 | A02~B51~C14~DR12 | 0.00149 | A11~B15~C03~DR11~DQ03 | 0.00181 | A03~B35~C04~DR01~DQ05~DP09 | 0.00113 | DR15~DQ05~DP14 | 0.00063 | A03~B49~DR11 | 0.0015 |
| A68~B35~C02 | 0.00136 | A01~B37~C06~DR10 | 0.00149 | A68~B40~C03~DR13~DQ06 | 0.00181 | A24~B35~C03~DR01~DQ05~DP03 | 0.00113 | DR11~DQ05~DP02 | 0.0005 | A11~B51~DR14 | 0.0015 |
| A03~B44~C02 | 0.00132 | A23~B49~C07~DR04 | 0.00147 | A29~B07~C15~DR10~DQ05 | 0.00181 | A01~B44~C16~DR15~DQ06~DP10 | 0.00113 | DR15~DQ06~DP13 | 0.00046 | A03~B51~DR07 | 0.0014 |
| A01~B50~C06 | 0.00132 | A02~B40~C03~DR11 | 0.00147 | A03~B50~C06~DR04~DQ04 | 0.00181 | A02~B50~C06~DR11~DQ03~DP04 | 0.00113 | DR13~DQ06~DP09 | 0.00043 | A11~B51~DR11 | 0.0014 |
| A68~B35~C16 | 0.00131 | A24~B44~C02~DR16 | 0.00146 | A32~B38~C12~DR14~DQ05 | 0.00181 | A02~B35~C04~DR04~DQ03~DP04 | 0.00113 | DR15~DQ05~DP05 | 0.00042 | A01~B41~DR07 | 0.0014 |
| A03~B55~C03 | 0.00131 | A11~B13~C06~DR07 | 0.00146 | A31~B27~C02~DR15~DQ06 | 0.00181 | A32~B35~C04~DR11~DQ03~DP03 | 0.00113 | DR04~DQ05~DP04 | 0.00021 | A01~B35~DR03 | 0.0014 |
| A30~B44~C16 | 0.00131 | A02~B40~C03~DR07 | 0.00145 | A24~B48~C08~DR08~DQ04 | 0.00181 | A32~B51~C16~DR04~DQ03~DP03 | 0.00113 | DR04~DQ03~DP15 | 0.0002 | A11~B13~DR07 | 0.0014 |
| A02~B15~C12 | 0.00129 | A68~B51~C15~DR07 | 0.00145 | A33~B58~C03~DR13~DQ06 | 0.00181 | A03~B35~C04~DR11~DQ03~DP02 | 0.00113 | DR13~DQ03~DP01 | 0.00018 | A02~B44~DR13 | 0.0014 |
| A24~B27~C02 | 0.00126 | A11~B40~C03~DR11 | 0.00144 | A02~B14~C08~DR04~DQ03 | 0.00181 | A03~B07~C07~DR04~DQ04~DP08 | 0.00113 | DR15~DQ03~DP02 | 0.00016 | A23~B49~DR13 | 0.0014 |
| A33~B51~C16 | 0.00126 | A03~B15~C07~DR13 | 0.00142 | A30~B14~C08~DR04~DQ03 | 0.00181 | A24~B07~C07~DR04~DQ03~DP02 | 0.00113 |  |  | A03~B55~DR13 | 0.0014 |
| A23~B35~C04 | 0.00125 | A03~B44~C12~DR04 | 0.00142 | A03~B51~C15~DR13~DQ03 | 0.00181 | A24~B35~C04~DR04~DQ03~DP02 | 0.00113 |  |  | A02~B55~DR04 | 0.0014 |
| A31~B48~C08 | 0.00125 | A11~B35~C04~DR15 | 0.0014 | A01~B41~C07~DR03~DQ02 | 0.00181 | A11~B50~C06~DR11~DQ03~DP02 | 0.00113 |  |  | A33~B35~DR13 | 0.0014 |
| A11~B15~C07 | 0.00123 | A01~B51~C14~DR15 | 0.00137 | A31~B52~C12~DR14~DQ05 | 0.00181 | A01~B51~C05~DR11~DQ03~DP01 | 0.00113 |  |  | A03~B07~DR13 | 0.0014 |
| A03~B38~C12 | 0.00122 | A02~B51~C16~DR13 | 0.00136 | A01~B27~C07~DR04~DQ03 | 0.00181 | A24~B57~C06~DR03~DQ02~DP09 | 0.00113 |  |  | A23~B49~DR15 | 0.0014 |
| A11~B55~C03 | 0.00121 | A11~B07~C07~DR15 | 0.00136 | A02~B15~C15~DR08~DQ04 | 0.00181 | A24~B18~C07~DR11~DQ03~DP04 | 0.00113 |  |  | A01~B35~DR14 | 0.0013 |
| A03~B35~C02 | 0.00121 | A01~B08~C07~DR13 | 0.00133 | A24~B35~C12~DR13~DQ06 | 0.00181 | A33~B35~C08~DR01~DQ02~DP17 | 0.00113 |  |  | A26~B35~DR04 | 0.0013 |
| A02~B55~C03 | 0.00119 | A24~B55~C03~DR14 | 0.00133 | A02~B40~C03~DR09~DQ03 | 0.00181 | A03~B44~C05~DR11~DQ03~DP02 | 0.00113 |  |  | A02~B52~DR15 | 0.0013 |
| A01~B35~C07 | 0.00117 | A32~B38~C12~DR11 | 0.00132 | A33~B07~C07~DR01~DQ05 | 0.00181 | A03~B44~C04~DR11~DQ03~DP04 | 0.00113 |  |  | A02~B15~DR11 | 0.0013 |
| A02~B46~C01 | 0.00116 | A01~B40~C15~DR15 | 0.0013 | A32~B35~C04~DR16~DQ05 | 0.00181 | A01~B44~C07~DR15~DQ06~DP04 | 0.00113 |  |  | A24~B18~DR15 | 0.0013 |
| A23~B38~C12 | 0.00113 | A66~B41~C17~DR13 | 0.0013 | A01~B51~C06~DR07~DQ02 | 0.00181 | A03~B40~C03~DR16~DQ05~DP04 | 0.00113 |  |  | A32~B13~DR07 | 0.0013 |
| A31~B35~C04 | 0.00113 | A02~B39~C07~DR04 | 0.0013 | A02~B15~C03~DR15~DQ06 | 0.00181 | A11~B55~C05~DR16~DQ05~DP10 | 0.00113 |  |  | A32~B35~DR13 | 0.0013 |
| A11~B35~C02 | 0.0011 | A02~B15~C01~DR13 | 0.0013 | A68~B15~C04~DR04~DQ03 | 0.00181 | A01~B35~C06~DR07~DQ02~DP02 | 0.00113 |  |  | A02~B18~DR16 | 0.0013 |
| A31~B51~C15 | 0.0011 | A02~B39~C07~DR09 | 0.0013 | A29~B50~C06~DR11~DQ03 | 0.00181 | A01~B13~C07~DR04~DQ03~DP17 | 0.00113 |  |  | A02~B18~DR15 | 0.0013 |
| A02~B35~C06 | 0.00109 | A30~B44~C16~DR07 | 0.0013 | A24~B44~C07~DR11~DQ03 | 0.00181 | A30~B49~C16~DR11~DQ03~DP09 | 0.00113 |  |  | A02~B44~DR03 | 0.0013 |
| A01~B18~C12 | 0.00108 | A24~B40~C03~DR03 | 0.0013 | A24~B40~C03~DR08~DQ04 | 0.00181 | A03~B44~C05~DR01~DQ05~DP01 | 0.00113 |  |  | A01~B35~DR15 | 0.0013 |
| A01~B38~C07 | 0.00108 | A29~B07~C15~DR10 | 0.0013 | A68~B35~C04~DR13~DQ06 | 0.00181 | A26~B51~C01~DR10~DQ05~DP02 | 0.00113 |  |  | A30~B13~DR13 | 0.0013 |
| A24~B15~C07 | 0.00106 | A33~B48~C08~DR16 | 0.0013 | A30~B51~C16~DR11~DQ03 | 0.00181 | A24~B50~C06~DR11~DQ03~DP04 | 0.00113 |  |  | A01~B15~DR04 | 0.0013 |
| A02~B44~C04 | 0.00106 | A01~B41~C17~DR07 | 0.0013 | A01~B57~C06~DR03~DQ02 | 0.00181 | A30~B13~C06~DR15~DQ05~DP04 | 0.00113 |  |  | A03~B51~DR13 | 0.0013 |
| A24~B50~C06 | 0.00105 | A32~B27~C02~DR07 | 0.0013 | A24~B35~C04~DR13~DQ03 | 0.00181 | A30~B13~C06~DR11~DQ03~DP04 | 0.00113 |  |  | A02~B18~DR04 | 0.0012 |
| A68~B44~C16 | 0.00104 | A01~B13~C16~DR07 | 0.0013 | A33~B35~C08~DR01~DQ05 | 0.00181 | A02~B44~C02~DR11~DQ03~DP22 | 0.00113 |  |  | A03~B18~DR01 | 0.0012 |
| A03~B57~C06 | 0.00104 | A32~B40~C02~DR16 | 0.0013 | A03~B13~C06~DR07~DQ02 | 0.00181 | A02~B44~C05~DR12~DQ03~DP04 | 0.00113 |  |  | A02~B50~DR15 | 0.0012 |
| A31~B44~C04 | 0.00104 | A01~B35~C06~DR11 | 0.00129 | A31~B15~C01~DR11~DQ03 | 0.00181 | A68~B44~C05~DR11~DQ03~DP02 | 0.00113 |  |  | A02~B51~DR07 | 0.0012 |
| A02~B45~C16 | 0.00104 | A26~B35~C04~DR11 | 0.00128 | A23~B52~C12~DR15~DQ06 | 0.00176 | A25~B40~C02~DR01~DQ05~DP02 | 0.00113 |  |  | A24~B18~DR04 | 0.0012 |
| A68~B35~C07 | 0.00104 | A02~B27~C02~DR09 | 0.00128 | A24~B55~C01~DR13~DQ06 | 0.00174 | A01~B07~C07~DR15~DQ05~DP04 | 0.00113 |  |  | A24~B55~DR11 | 0.0012 |
| A23~B35~C06 | 0.00104 | A29~B07~C15~DR15 | 0.00128 | A02~B40~C03~DR07~DQ02 | 0.00172 | A24~B44~C16~DR03~DQ02~DP01 | 0.00113 |  |  | A24~B49~DR04 | 0.0012 |
| A25~B52~C02 | 0.00104 | A24~B44~C05~DR16 | 0.00128 | A02~B18~C07~DR16~DQ05 | 0.00167 | A02~B13~C06~DR11~DQ02~DP10 | 0.00113 |  |  | A29~B07~DR04 | 0.0012 |
| A31~B35~C14 | 0.00104 | A02~B18~C07~DR10 | 0.00128 | A02~B52~C12~DR15~DQ06 | 0.00167 | A32~B35~C06~DR07~DQ03~DP03 | 0.00113 |  |  | A32~B51~DR15 | 0.0012 |
| A25~B27~C04 | 0.00104 | A01~B49~C07~DR14 | 0.00127 | A32~B51~C14~DR03~DQ02 | 0.00165 | A02~B15~C12~DR04~DQ06~DP04 | 0.00113 |  |  | A02~B51~DR10 | 0.0012 |
| A11~B57~C04 | 0.00104 | A32~B35~C04~DR13 | 0.00125 | A03~B07~C07~DR11~DQ03 | 0.00157 | A11~B08~C07~DR03~DQ02~DP01 | 0.00113 |  |  | A11~B35~DR10 | 0.0012 |
| A26~B27~C01 | 0.00104 | A26~B08~C07~DR10 | 0.00125 | A02~B51~C15~DR09~DQ03 | 0.00136 | A01~B44~C15~DR11~DQ03~DP04 | 0.00113 |  |  | A01~B07~DR11 | 0.0012 |
| A02~B55~C01 | 0.00103 | A02~B14~C08~DR01 | 0.00125 | A02~B44~C07~DR16~DQ05 | 0.00127 | A26~B27~C01~DR01~DQ05~DP03 | 0.00113 |  |  | A03~B07~DR10 | 0.0012 |
| A30~B53~C04 | 0.00103 | A01~B37~C06~DR07 | 0.00125 | A23~B35~C04~DR13~DQ06 | 0.00112 | A03~B35~C04~DR13~DQ06~DP11 | 0.00113 |  |  | A24~B35~DR16 | 0.0012 |
| A69~B35~C12 | 0.00103 | A02~B44~C04~DR07 | 0.00124 | A24~B08~C07~DR03~DQ02 | 0.00109 | A11~B35~C04~DR01~DQ05~DP04 | 0.00113 |  |  | A32~B35~DR15 | 0.0012 |
| A01~B51~C16 | 0.00102 | A02~B35~C04~DR15 | 0.00122 | A11~B49~C07~DR14~DQ05 | 0.00107 | A11~B44~C04~DR07~DQ02~DP09 | 0.00113 |  |  | A02~B49~DR01 | 0.0012 |
| A11~B40~C07 | 0.00102 | A03~B50~C06~DR15 | 0.00122 | A68~B18~C12~DR11~DQ03 | 0.00106 | A68~B07~C07~DR16~DQ05~DP04 | 0.00113 |  |  | A33~B58~DR13 | 0.0012 |
| A01~B15~C03 | 0.00102 | A24~B51~C14~DR14 | 0.00121 | A11~B51~C14~DR03~DQ02 | 0.00106 | A02~B35~C04~DR16~DQ05~DP02 | 0.00113 |  |  | A01~B49~DR13 | 0.0012 |
| A01~B38~C12 | 0.00101 | A24~B44~C05~DR04 | 0.00121 | A02~B15~C03~DR04~DQ03 | 0.00104 | A02~B07~C07~DR04~DQ03~DP04 | 0.00113 |  |  | A01~B27~DR04 | 0.0012 |
| A11~B07~C07 | 0.00101 | A02~B15~C03~DR04 | 0.00121 | A11~B35~C04~DR15~DQ06 | 0.00103 | A02~B07~C07~DR04~DQ05~DP04 | 0.00113 |  |  | A23~B44~DR01 | 0.0012 |
| A01~B57~C18 | 0.001 | A32~B51~C14~DR15 | 0.0012 | A01~B18~C07~DR13~DQ06 | 0.00102 | A02~B35~C04~DR16~DQ05~DP04 | 0.00113 |  |  | A23~B49~DR04 | 0.0012 |
| A02~B27~C01 | 0.001 | A32~B51~C15~DR04 | 0.00119 | A02~B51~C16~DR04~DQ03 | 0.001 | A68~B35~C04~DR04~DQ03~DP02 | 0.00113 |  |  | A30~B51~DR11 | 0.0012 |
| A32~B51~C16 | 0.00099 | A68~B44~C14~DR04 | 0.00119 | A01~B35~C04~DR07~DQ02 | 0.001 | A25~B18~C12~DR11~DQ03~DP06 | 0.00113 |  |  | A02~B35~DR07 | 0.0011 |
| A11~B27~C02 | 0.00099 | A24~B18~C07~DR13 | 0.00118 | A02~B50~C06~DR01~DQ05 | 0.00099 | A02~B35~C04~DR13~DQ03~DP04 | 0.00113 |  |  | A02~B40~DR07 | 0.0011 |
| A29~B50~C06 | 0.00098 | A02~B38~C07~DR13 | 0.00117 | A26~B55~C01~DR13~DQ06 | 0.00098 | A68~B55~C03~DR15~DQ05~DP13 | 0.00113 |  |  | A24~B15~DR11 | 0.0011 |
| A01~B48~C08 | 0.00098 | A68~B51~C15~DR11 | 0.00116 | A24~B51~C16~DR16~DQ05 | 0.00093 | A03~B07~C07~DR14~DQ05~DP01 | 0.00113 |  |  | A02~B07~DR11 | 0.0011 |
| A68~B51~C14 | 0.00098 | A24~B51~C15~DR13 | 0.00114 | A02~B51~C15~DR15~DQ06 | 0.00092 | A26~B51~C14~DR15~DQ06~DP03 | 0.00113 |  |  | A24~B27~DR04 | 0.0011 |
| A32~B07~C07 | 0.00097 | A01~B49~C07~DR01 | 0.00114 | A03~B07~C07~DR14~DQ05 | 0.00092 | A29~B35~C07~DR01~DQ05~DP02 | 0.00113 |  |  | A02~B13~DR13 | 0.0011 |
| A11~B44~C04 | 0.00097 | A26~B38~C12~DR15 | 0.00113 | A03~B35~C04~DR15~DQ06 | 0.00091 | A02~B18~C07~DR11~DQ03~DP02 | 0.00113 |  |  | A11~B35~DR07 | 0.0011 |
| A26~B44~C07 | 0.00095 | A24~B37~C06~DR10 | 0.00113 | A30~B08~C07~DR11~DQ03 | 0.0009 | A26~B18~C07~DR15~DQ05~DP04 | 0.00113 |  |  | A31~B35~DR13 | 0.0011 |
| A02~B48~C08 | 0.00095 | A24~B51~C14~DR08 | 0.00112 | A30~B40~C03~DR11~DQ03 | 0.0009 | A68~B49~C07~DR14~DQ05~DP05 | 0.00113 |  |  | A03~B38~DR13 | 0.0011 |
| A24~B27~C01 | 0.00095 | A03~B55~C03~DR14 | 0.00112 | A02~B60~C03~DR11~DQ03 | 0.0009 | A32~B51~C15~DR14~DQ05~DP05 | 0.00113 |  |  | A24~B07~DR13 | 0.0011 |
| A31~B51~C14 | 0.00095 | A23~B35~C04~DR07 | 0.00112 | A02~B07~C07~DR13~DQ06 | 0.0009 | A11~B35~C04~DR04~DQ03~DP04 | 0.00113 |  |  | A02~B07~DR08 | 0.0011 |
| A30~B40~C03 | 0.00093 | A11~B35~C04~DR04 | 0.00112 | A02~B51~C12~DR14~DQ05 | 0.0009 | A11~B35~C04~DR04~DQ03~DP02 | 0.00113 |  |  | A11~B51~DR13 | 0.0011 |
| A11~B51~C14 | 0.00092 | A30~B08~C07~DR03 | 0.0011 | A24~B44~C04~DR07~DQ03 | 0.0009 | A30~B51~C14~DR08~DQ03~DP04 | 0.00113 |  |  | A26~B38~DR15 | 0.0011 |
| A01~B08~C06 | 0.0009 | A32~B51~C16~DR11 | 0.0011 | A01~B45~C16~DR04~DQ03 | 0.0009 | A32~B18~C07~DR12~DQ03~DP04 | 0.00113 |  |  | A01~B07~DR15 | 0.0011 |
| A01~B35~C15 | 0.0009 | A03~B08~C07~DR01 | 0.0011 | A24~B13~C14~DR04~DQ04 | 0.0009 | A26~B51~C16~DR13~DQ06~DP04 | 0.00113 |  |  | A24~B07~DR01 | 0.0011 |
| A02~B35~C01 | 0.00087 | A02~B38~C12~DR15 | 0.00109 | A02~B55~C01~DR11~DQ03 | 0.0009 | A26~B44~C05~DR13~DQ06~DP04 | 0.00113 |  |  | A32~B35~DR16 | 0.0011 |
| A32~B15~C01 | 0.00087 | A11~B35~C04~DR13 | 0.00109 | A01~B55~C01~DR14~DQ05 | 0.0009 | A02~B15~C07~DR04~DQ03~DP04 | 0.00113 |  |  | A68~B07~DR11 | 0.0011 |
| A26~B51~C16 | 0.00085 | A02~B41~C07~DR03 | 0.00108 | A32~B55~C01~DR15~DQ06 | 0.0009 | A23~B27~C15~DR11~DQ03~DP02 | 0.00113 |  |  | A11~B40~DR11 | 0.0011 |
| A02~B44~C02 | 0.00085 | A01~B51~C07~DR11 | 0.00108 | A26~B07~C12~DR15~DQ05 | 0.0009 | A32~B49~C07~DR04~DQ03~DP02 | 0.00113 |  |  | A02~B14~DR01 | 0.0011 |
| A68~B27~C02 | 0.00083 | A23~B49~C07~DR03 | 0.00108 | A23~B49~C07~DR15~DQ06 | 0.0009 | A01~B51~C14~DR08~DQ06~DP03 | 0.00113 |  |  | A24~B50~DR15 | 0.0011 |
| A11~B15~C03 | 0.00082 | A02~B39~C12~DR09 | 0.00108 | A01~B57~C06~DR15~DQ06 | 0.0009 | A01~B51~C14~DR15~DQ04~DP03 | 0.00113 |  |  | A31~B35~DR11 | 0.0011 |
| A03~B55~C01 | 0.00081 | A11~B38~C12~DR04 | 0.00108 | A01~B49~C07~DR15~DQ06 | 0.0009 | A31~B44~C05~DR11~DQ03~DP02 | 0.00113 |  |  | A68~B51~DR13 | 0.0011 |
| A03~B18~C07 | 0.0008 | A03~B13~C06~DR07 | 0.00107 | A03~B37~C06~DR16~DQ05 | 0.0009 | A32~B18~C12~DR04~DQ03~DP03 | 0.00113 |  |  | A03~B44~DR16 | 0.0011 |
| A02~B51~C03 | 0.00079 | A24~B35~C04~DR08 | 0.00107 | A69~B35~C04~DR11~DQ03 | 0.0009 | A24~B50~C06~DR11~DQ06~DP04 | 0.00113 |  |  | A33~B51~DR11 | 0.0011 |
| A29~B44~C16 | 0.00079 | A11~B35~C02~DR04 | 0.00106 | A74~B50~C06~DR07~DQ02 | 0.0009 | A30~B58~C07~DR15~DQ06~DP04 | 0.00113 |  |  | A03~B15~DR13 | 0.0011 |
| A26~B07~C12 | 0.00079 | A02~B51~C15~DR16 | 0.00106 | A23~B44~C05~DR01~DQ05 | 0.0009 | A31~B38~C12~DR16~DQ05~DP02 | 0.00113 |  |  | A69~B18~DR15 | 0.0011 |
| A24~B07~C04 | 0.00078 | A01~B51~C15~DR15 | 0.00104 | A01~B50~C06~DR03~DQ02 | 0.0009 | A02~B51~C02~DR01~DQ05~DP17 | 0.00113 |  |  | A03~B18~DR04 | 0.0010 |
| A02~B44~C06 | 0.00078 | A02~B13~C06~DR14 | 0.00104 | A68~B44~C04~DR03~DQ02 | 0.0009 | A03~B51~C01~DR04~DQ03~DP04 | 0.00113 |  |  | A01~B35~DR07 | 0.0010 |
| A24~B50~C15 | 0.00078 | A02~B58~C07~DR08 | 0.00104 | A24~B55~C03~DR14~DQ05 | 0.0009 | A33~B58~C03~DR04~DQ03~DP02 | 0.00113 |  |  | A68~B35~DR07 | 0.0010 |
| A02~B15~C06 | 0.00078 | A24~B35~C07~DR03 | 0.00104 | A02~B50~C06~DR11~DQ06 | 0.0009 | A26~B39~C17~DR04~DQ04~DP15 | 0.00113 |  |  | A02~B08~DR01 | 0.0010 |
| A24~B55~C14 | 0.00078 | A03~B40~C03~DR01 | 0.00104 | A01~B50~C16~DR15~DQ06 | 0.0009 | A66~B41~C12~DR10~DQ05~DP04 | 0.00113 |  |  | A02~B51~DR03 | 0.0010 |
| A24~B18~C02 | 0.00078 | A29~B07~C15~DR04 | 0.00104 | A24~B35~C07~DR11~DQ03 | 0.0009 | A33~B51~C07~DR01~DQ05~DP10 | 0.00113 |  |  | A68~B18~DR11 | 0.0010 |
| A24~B35~C18 | 0.00078 | A02~B40~C15~DR15 | 0.00104 | A03~B07~C07~DR04~DQ04 | 0.0009 | A30~B27~C01~DR08~DQ04~DP04 | 0.00113 |  |  | A01~B51~DR15 | 0.0010 |
| A02~B54~C01 | 0.00078 | A24~B27~C01~DR11 | 0.00104 | A24~B07~C07~DR04~DQ03 | 0.0009 | A03~B44~C04~DR07~DQ03~DP09 | 0.00113 |  |  | A68~B35~DR14 | 0.0010 |
| A01~B41~C06 | 0.00078 | A11~B08~C04~DR03 | 0.00104 | A11~B50~C06~DR11~DQ03 | 0.0009 | A69~B07~C07~DR11~DQ02~DP02 | 0.00113 |  |  | A31~B35~DR16 | 0.0010 |
| A68~B37~C06 | 0.00078 | A01~B14~C08~DR04 | 0.00104 | A01~B51~C16~DR11~DQ03 | 0.0009 | A24~B51~C15~DR11~DQ03~DP03 | 0.00113 |  |  | A32~B35~DR14 | 0.0010 |
| A11~B39~C07 | 0.00078 | A23~B51~C16~DR13 | 0.00104 | A23~B49~C07~DR14~DQ03 | 0.0009 | A26~B35~C04~DR13~DQ03~DP04 | 0.00113 |  |  | A24~B15~DR04 | 0.0010 |
| A02~B27~C14 | 0.00078 | A11~B51~C04~DR15 | 0.00104 | A02~B35~C07~DR14~DQ05 | 0.0009 | A68~B15~C12~DR08~DQ06~DP04 | 0.00113 |  |  | A26~B07~DR15 | 0.0010 |
| A33~B73~C15 | 0.00078 | A31~B35~C14~DR11 | 0.00104 | A03~B44~C05~DR11~DQ03 | 0.0009 | A02~B44~C14~DR13~DQ06~DP04 | 0.00113 |  |  | A24~B13~DR03 | 0.0010 |
| A68~B35~C03 | 0.00077 | A33~B51~C14~DR11 | 0.00104 | A01~B44~C07~DR15~DQ06 | 0.0009 | A01~B40~C03~DR04~DQ04~DP03 | 0.00113 |  |  | A11~B50~DR07 | 0.0010 |
| A01~B41~C07 | 0.00077 | A11~B51~C15~DR07 | 0.00104 | A03~B40~C05~DR16~DQ05 | 0.0009 | A23~B51~C03~DR04~DQ04~DP03 | 0.00113 |  |  | A31~B50~DR07 | 0.0010 |
| A69~B07~C07 | 0.00077 | A11~B57~C04~DR13 | 0.00104 | A01~B13~C07~DR04~DQ03 | 0.0009 | A02~B15~C03~DR04~DQ03~DP04 | 0.00113 |  |  | A01~B57~DR16 | 0.0010 |
| A33~B35~C04 | 0.00077 | A03~B41~C17~DR03 | 0.00104 | A26~B51~C16~DR10~DQ05 | 0.0009 | A03~B52~C12~DR14~DQ04~DP02 | 0.00113 |  |  | A30~B18~DR03 | 0.0010 |
| A23~B08~C04 | 0.00076 | A01~B57~C18~DR16 | 0.00104 | A24~B50~C06~DR11~DQ03 | 0.0009 | A26~B38~C12~DR15~DQ02~DP04 | 0.00113 |  |  | A02~B39~DR08 | 0.0010 |
| A32~B27~C15 | 0.00076 | A01~B52~C12~DR15 | 0.00103 | A02~B35~C12~DR15~DQ05 | 0.0009 | A11~B38~C12~DR10~DQ05~DP04 | 0.00113 |  |  | A24~B52~DR08 | 0.0010 |
| A03~B44~C04 | 0.00076 | A02~B15~C03~DR15 | 0.00101 | A68~B44~C05~DR12~DQ03 | 0.0009 | A23~B49~C12~DR15~DQ06~DP04 | 0.00113 |  |  | A02~B57~DR13 | 0.0009 |
| A03~B27~C12 | 0.00075 | A02~B51~C14~DR13 | 0.00101 | A25~B40~C02~DR01~DQ05 | 0.0009 | A24~B35~C07~DR07~DQ02~DP03 | 0.00113 |  |  | A24~B15~DR13 | 0.0009 |
| A68~B13~C16 | 0.00075 | A26~B48~C08~DR12 | 0.001 | A01~B51~C07~DR04~DQ03 | 0.0009 | A02~B51~C15~DR11~DQ03~DP10 | 0.00113 |  |  | A03~B38~DR11 | 0.0009 |
| A33~B18~C12 | 0.00074 | A32~B35~C04~DR15 | 0.001 | A02~B07~C07~DR03~DQ02 | 0.0009 | A02~B51~C16~DR09~DQ03~DP10 | 0.00113 |  |  | A02~B50~DR03 | 0.0009 |
| A23~B51~C15 | 0.00072 | A02~B44~C16~DR03 | 0.001 | A02~B44~C16~DR03~DQ02 | 0.0009 | A02~B40~C15~DR08~DQ04~DP03 | 0.00113 |  |  | A02~B07~DR03 | 0.0009 |
| A03~B51~C16 | 0.00072 | A02~B51~C16~DR04 | 0.00099 | A24~B44~C16~DR03~DQ02 | 0.0009 | A11~B35~C12~DR01~DQ03~DP04 | 0.00113 |  |  | A01~B08~DR11 | 0.0009 |
| A29~B44~C07 | 0.00071 | A01~B57~C06~DR03 | 0.00098 | A32~B13~C06~DR11~DQ03 | 0.0009 | A32~B38~C12~DR11~DQ05~DP04 | 0.00113 |  |  | A03~B51~DR03 | 0.0009 |
| A11~B08~C03 | 0.00071 | A02~B35~C04~DR16 | 0.00098 | A02~B15~C12~DR04~DQ06 | 0.0009 | A02~B35~C12~DR14~DQ02~DP04 | 0.00113 |  |  | A02~B18~DR14 | 0.0009 |
| A31~B40~C03 | 0.00071 | A11~B35~C12~DR15 | 0.00097 | A01~B44~C15~DR11~DQ03 | 0.0009 | A32~B52~C12~DR03~DQ05~DP04 | 0.00113 |  |  | A23~B35~DR11 | 0.0009 |
| A26~B44~C05 | 0.00071 | A23~B35~C04~DR13 | 0.00096 | A68~B07~C07~DR16~DQ05 | 0.0009 | A01~B35~C04~DR01~DQ05~DP04 | 0.00113 |  |  | A01~B38~DR11 | 0.0009 |
| A02~B51~C02 | 0.00069 | A33~B14~C08~DR13 | 0.00095 | A68~B35~C04~DR16~DQ05 | 0.0009 | A03~B49~C07~DR15~DQ06~DP02 | 0.00113 |  |  | A24~B51~DR08 | 0.0009 |
| A26~B39~C12 | 0.00069 | A02~B44~C05~DR01 | 0.00093 | A02~B07~C07~DR04~DQ05 | 0.0009 | A32~B15~C01~DR14~DQ05~DP05 | 0.00113 |  |  | A24~B07~DR04 | 0.0009 |
| A29~B49~C07 | 0.00069 | A02~B50~C06~DR16 | 0.00093 | A02~B35~C04~DR16~DQ05 | 0.0009 | A24~B44~C04~DR04~DQ03~DP02 | 0.00113 |  |  | A23~B44~DR11 | 0.0009 |
| A29~B51~C15 | 0.00069 | A03~B35~C04~DR08 | 0.00092 | A25~B18~C12~DR11~DQ03 | 0.0009 | A01~B44~C07~DR16~DQ05~DP23 | 0.00113 |  |  | A01~B57~DR04 | 0.0009 |
| A24~B57~C06 | 0.00069 | A24~B44~C05~DR11 | 0.00091 | A26~B55~C03~DR11~DQ03 | 0.0009 | A02~B44~C07~DR09~DQ03~DP04 | 0.00113 |  |  | A02~B35~DR12 | 0.0009 |
| A29~B35~C07 | 0.00068 | A01~B07~C07~DR03 | 0.00091 | A26~B55~C03~DR15~DQ05 | 0.0009 | A02~B51~C14~DR14~DQ03~DP02 | 0.00113 |  |  | A32~B40~DR16 | 0.0009 |
| A02~B35~C14 | 0.00067 | A24~B44~C05~DR13 | 0.00091 | A26~B49~C07~DR15~DQ05 | 0.0009 | A01~B57~C06~DR13~DQ03~DP03 | 0.00113 |  |  | A03~B58~DR03 | 0.0009 |
| A24~B39~C12 | 0.00066 | A02~B35~C04~DR03 | 0.0009 | A02~B35~C07~DR01~DQ05 | 0.0009 | A30~B41~C17~DR13~DQ03~DP70 | 0.00113 |  |  | A33~B14~DR13 | 0.0009 |
| A03~B44~C07 | 0.00065 | A02~B49~C07~DR13 | 0.0009 | A02~B18~C07~DR03~DQ02 | 0.0009 | A66~B38~C12~DR08~DQ03~DP02 | 0.00113 |  |  | A01~B49~DR11 | 0.0009 |
| A11~B51~C06 | 0.00064 | A33~B58~C03~DR03 | 0.00089 | A32~B37~C01~DR10~DQ05 | 0.0009 | A03~B37~C06~DR04~DQ05~DP105 | 0.00113 |  |  | A29~B45~DR11 | 0.0009 |
| A24~B51~C01 | 0.00064 | A01~B35~C04~DR14 | 0.00089 | A68~B18~C07~DR14~DQ05 | 0.0009 | A01~B51~C07~DR09~DQ05~DP10 | 0.00113 |  |  | A68~B53~DR13 | 0.0009 |
| A68~B15~C04 | 0.00063 | A01~B18~C12~DR11 | 0.00089 | A32~B51~C15~DR14~DQ05 | 0.0009 | A03~B07~C14~DR16~DQ03~DP02 | 0.00113 |  |  | A24~B41~DR07 | 0.0009 |
| A11~B18~C12 | 0.00063 | A02~B57~C06~DR13 | 0.00089 | A03~B44~C04~DR04~DQ03 | 0.0009 | A02~B50~C01~DR01~DQ05~DP03 | 0.00113 |  |  | A68~B40~DR04 | 0.0009 |
| A11~B56~C01 | 0.00062 | A30~B40~C03~DR11 | 0.00089 | A26~B51~C16~DR13~DQ06 | 0.0009 | A24~B55~C06~DR16~DQ05~DP51 | 0.00113 |  |  | A33~B14~DR11 | 0.0009 |
| A03~B08~C06 | 0.00061 | A26~B07~C07~DR15 | 0.00087 | A02~B27~C15~DR11~DQ03 | 0.0009 | A01~B15~C07~DR07~DQ03~DP105 | 0.00113 |  |  | A29~B58~DR08 | 0.0009 |
| A33~B07~C07 | 0.00061 | A02~B15~C03~DR07 | 0.00087 | A23~B15~C07~DR04~DQ03 | 0.0009 | A23~B41~C17~DR04~DQ03~DP14 | 0.00113 |  |  | A32~B35~DR03 | 0.0009 |
| A03~B49~C07 | 0.00061 | A02~B44~C05~DR03 | 0.00086 | A01~B18~C12~DR04~DQ03 | 0.0009 | A01~B51~C06~DR13~DQ06~DP04 | 0.00113 |  |  | A02~B51~DR12 | 0.0009 |
| A03~B35~C06 | 0.0006 | A03~B35~C12~DR14 | 0.00086 | A01~B51~C14~DR08~DQ04 | 0.0009 | A24~B37~C14~DR14~DQ05~DP02 | 0.00113 |  |  | A68~B40~DR14 | 0.0009 |
| A02~B57~C18 | 0.0006 | A02~B35~C06~DR07 | 0.00084 | A01~B51~C14~DR15~DQ06 | 0.0009 | A24~B56~C01~DR04~DQ03~DP04 | 0.00113 |  |  | A30~B49~DR11 | 0.0009 |
| A31~B15~C03 | 0.00059 | A24~B35~C12~DR04 | 0.00084 | A24~B27~C01~DR03~DQ02 | 0.0009 | A32~B44~C05~DR12~DQ03~DP04 | 0.00113 |  |  | A30~B08~DR03 | 0.0009 |
| A02~B40~C02 | 0.00059 | A32~B39~C12~DR11 | 0.00083 | A24~B27~C01~DR11~DQ06 | 0.0009 | A25~B51~C15~DR01~DQ05~DP02 | 0.00113 |  |  | A24~B40~DR14 | 0.0009 |
| A29~B55~C01 | 0.00059 | A03~B51~C15~DR11 | 0.00083 | A30~B38~C12~DR15~DQ06 | 0.0009 | A11~B49~C07~DR14~DQ05~DP04 | 0.00113 |  |  | A26~B38~DR01 | 0.0009 |
| A24~B40~C02 | 0.00058 | A01~B50~C06~DR07 | 0.00082 | A31~B58~C07~DR16~DQ05 | 0.0009 | A24~B50~C01~DR08~DQ03~DP04 | 0.00113 |  |  | A02~B18~DR03 | 0.0009 |
| A30~B39~C12 | 0.00057 | A02~B51~C15~DR15 | 0.00082 | A02~B51~C02~DR04~DQ03 | 0.0009 | A26~B56~C06~DR07~DQ03~DP04 | 0.00113 |  |  | A24~B50~DR03 | 0.0009 |
| A30~B41~C17 | 0.00057 | A32~B52~C12~DR04 | 0.00081 | A33~B58~C03~DR04~DQ03 | 0.0009 | A03~B08~C15~DR16~DQ05~DP04 | 0.00113 |  |  | A01~B41~DR13 | 0.0009 |
| A03~B35~C01 | 0.00056 | A26~B55~C01~DR11 | 0.00081 | A66~B41~C17~DR10~DQ05 | 0.0009 | A02~B39~C12~DR08~DQ03~DP04 | 0.00113 |  |  | A24~B55~DR08 | 0.0009 |
| A02~B18~C02 | 0.00056 | A24~B44~C16~DR04 | 0.0008 | A33~B51~C07~DR01~DQ05 | 0.0009 | A33~B14~C06~DR14~DQ05~DP04 | 0.00113 |  |  | A01~B57~DR01 | 0.0009 |
| A01~B58~C07 | 0.00055 | A01~B35~C15~DR11 | 0.00079 | A30~B27~C01~DR08~DQ04 | 0.0009 | A26~B35~C14~DR04~DQ06~DP03 | 0.00113 |  |  | A03~B15~DR11 | 0.0008 |
| A01~B13~C16 | 0.00055 | A33~B58~C03~DR13 | 0.00079 | A69~B07~C07~DR11~DQ02 | 0.0009 | A30~B13~C06~DR13~DQ03~DP04 | 0.00113 |  |  | A24~B18~DR13 | 0.0008 |
| A33~B51~C04 | 0.00054 | A01~B57~C06~DR15 | 0.00078 | A26~B15~C12~DR08~DQ03 | 0.0009 | A03~B13~C06~DR07~DQ02~DP04 | 0.00113 |  |  | A30~B13~DR11 | 0.0008 |
| A02~B52~C12 | 0.00054 | A23~B49~C07~DR14 | 0.00078 | A03~B44~C12~DR13~DQ06 | 0.0009 | A33~B35~C08~DR07~DQ02~DP51 | 0.00113 |  |  | A23~B41~DR07 | 0.0008 |
| A68~B40~C15 | 0.00054 | A01~B38~C12~DR13 | 0.00078 | A23~B41~C17~DR04~DQ03 | 0.0009 | A32~B18~C12~DR11~DQ05~DP04 | 0.00113 |  |  | A02~B35~DR10 | 0.0008 |
| A02~B51~C12 | 0.00053 | A25~B52~C02~DR15 | 0.00078 | A01~B40~C03~DR04~DQ04 | 0.0009 | A24~B44~C04~DR15~DQ06~DP04 | 0.00113 |  |  | A11~B55~DR07 | 0.0008 |
| A31~B44~C05 | 0.00053 | A26~B07~C12~DR15 | 0.00078 | A23~B51~C03~DR04~DQ04 | 0.0009 | A30~B14~C05~DR01~DQ05~DP03 | 0.00113 |  |  | A02~B39~DR04 | 0.0008 |
| A02~B51~C01 | 0.00053 | A01~B35~C07~DR03 | 0.00078 | A30~B35~C04~DR04~DQ03 | 0.0009 | A02~B35~C04~DR01~DQ05~DP03 | 0.00113 |  |  | A68~B13~DR07 | 0.0008 |
| A11~B52~C07 | 0.00053 | A33~B73~C15~DR04 | 0.00078 | A03~B52~C12~DR15~DQ04 | 0.0009 | A29~B14~C18~DR01~DQ05~DP04 | 0.00113 |  |  | A24~B37~DR10 | 0.0008 |
| A24~B35~C05 | 0.00053 | A32~B18~C12~DR04 | 0.00078 | A26~B38~C12~DR14~DQ02 | 0.0009 | A29~B57~C08~DR01~DQ05~DP01 | 0.00113 |  |  | A01~B44~DR11 | 0.0008 |
| A26~B13~C06 | 0.00052 | A03~B51~C01~DR01 | 0.00078 | A11~B35~C04~DR10~DQ05 | 0.0009 | A68~B37~C06~DR13~DQ05~DP02 | 0.00113 |  |  | A01~B57~DR03 | 0.0008 |
| A03~B35~C16 | 0.00052 | A30~B35~C12~DR11 | 0.00078 | A24~B35~C12~DR07~DQ02 | 0.0009 | A23~B35~C04~DR13~DQ06~DP04 | 0.00113 |  |  | A03~B07~DR01 | 0.0008 |
| A24~B35~C01 | 0.00052 | A24~B40~C02~DR11 | 0.00078 | A02~B40~C15~DR08~DQ04 | 0.0009 | A11~B55~C07~DR03~DQ03~DP04 | 0.00113 |  |  | A02~B50~DR11 | 0.0008 |
| A23~B35~C07 | 0.00052 | A02~B15~C12~DR04 | 0.00078 | A11~B35~C12~DR01~DQ05 | 0.0009 | A29~B49~C01~DR11~DQ03~DP04 | 0.00113 |  |  | A25~B18~DR04 | 0.0008 |
| A03~B35~C07 | 0.00052 | A26~B27~C01~DR01 | 0.00078 | A32~B52~C12~DR03~DQ02 | 0.0009 | A32~B35~C04~DR11~DQ03~DP02 | 0.00113 |  |  | A23~B49~DR01 | 0.0008 |
| A01~B18~C06 | 0.00052 | A26~B55~C03~DR11 | 0.00078 | A01~B35~C04~DR01~DQ05 | 0.0009 | A24~B37~C12~DR12~DQ03~DP04 | 0.00113 |  |  | A26~B40~DR04 | 0.0008 |
| A68~B51~C07 | 0.00052 | A26~B51~C16~DR13 | 0.00078 | A32~B15~C01~DR14~DQ05 | 0.0009 | A03~B35~C02~DR01~DQ05~DP04 | 0.00113 |  |  | A03~B49~DR04 | 0.0008 |
| A31~B35~C08 | 0.00052 | A03~B44~C07~DR07 | 0.00078 | A24~B44~C04~DR04~DQ03 | 0.0009 | A24~B35~C02~DR03~DQ06~DP10 | 0.00113 |  |  | A02~B27~DR08 | 0.0008 |
| A68~B18~C16 | 0.00052 | A24~B18~C02~DR11 | 0.00078 | A02~B51~C14~DR14~DQ03 | 0.0009 | A01~B35~C06~DR01~DQ05~DP10 | 0.00113 |  |  | A24~B07~DR07 | 0.0008 |
| A74~B50~C06 | 0.00052 | A23~B51~C15~DR14 | 0.00078 | A30~B38~C12~DR13~DQ03 | 0.0009 | A29~B58~C15~DR11~DQ03~DP04 | 0.00113 |  |  | A24~B51~DR07 | 0.0008 |
| A33~B35~C08 | 0.00052 | A32~B51~C15~DR11 | 0.00078 | A66~B41~C17~DR08~DQ03 | 0.0009 | A23~B38~C12~DR11~DQ03~DP14 | 0.00113 |  |  | A26~B35~DR03 | 0.0008 |
| A66~B07~C07 | 0.00052 | A03~B27~C12~DR15 | 0.00078 | A01~B37~C06~DR04~DQ05 | 0.0009 | A02~B13~C06~DR07~DQ02~DP15 | 0.00113 |  |  | A01~B49~DR14 | 0.0008 |
| A03~B15~C15 | 0.00052 | A03~B35~C02~DR04 | 0.00078 | A03~B37~C06~DR04~DQ05 | 0.0009 | A24~B27~C01~DR04~DQ03~DP03 | 0.00113 |  |  | A31~B27~DR04 | 0.0008 |
| A11~B40~C14 | 0.00052 | A24~B14~C08~DR04 | 0.00078 | A01~B07~C14~DR09~DQ03 | 0.0009 | A03~B07~C07~DR11~DQ03~DP13 | 0.00113 |  |  | A26~B08~DR10 | 0.0008 |
| A01~B51~C06 | 0.00052 | A01~B40~C03~DR09 | 0.00078 | A24~B55~C01~DR16~DQ05 | 0.0009 | A01~B51~C01~DR11~DQ06~DP01 | 0.00113 |  |  | A29~B58~DR04 | 0.0008 |
| A31~B14~C08 | 0.00052 | A29~B50~C06~DR11 | 0.00078 | A01~B15~C07~DR07~DQ03 | 0.0009 | A29~B38~C12~DR11~DQ03~DP17 | 0.00113 |  |  | A11~B40~DR04 | 0.0008 |
| A26~B44~C14 | 0.00052 | A24~B40~C03~DR08 | 0.00078 | A03~B41~C17~DR04~DQ03 | 0.0009 | A03~B18~C12~DR13~DQ06~DP02 | 0.00113 |  |  | A02~B15~DR03 | 0.0008 |
| A66~B18~C12 | 0.00052 | A01~B41~C06~DR03 | 0.00078 | A03~B35~C04~DR07~DQ03 | 0.0009 | A11~B38~C12~DR13~DQ06~DP02 | 0.00113 |  |  | A01~B37~DR15 | 0.0008 |
| A24~B15~C15 | 0.00052 | A68~B37~C06~DR13 | 0.00078 | A24~B37~C06~DR14~DQ05 | 0.0009 | A02~B35~C04~DR03~DQ02~DP04 | 0.00113 |  |  | A30~B35~DR03 | 0.0008 |
| A32~B51~C01 | 0.00052 | A02~B51~C01~DR14 | 0.00078 | A32~B56~C01~DR12~DQ03 | 0.0009 | A33~B07~C07~DR03~DQ02~DP02 | 0.00113 |  |  | A02~B13~DR03 | 0.0008 |
| A26~B41~C07 | 0.00052 | A24~B13~C06~DR08 | 0.00078 | A25~B51~C15~DR11~DQ05 | 0.0009 | A11~B51~C15~DR04~DQ03~DP04 | 0.00113 |  |  | A30~B51~DR13 | 0.0008 |
| A26~B53~C04 | 0.00052 | A33~B38~C12~DR15 | 0.00078 | A24~B50~C06~DR08~DQ03 | 0.0009 | A11~B51~C15~DR04~DQ03~DP05 | 0.00113 |  |  | A26~B55~DR11 | 0.0008 |
| A11~B15~C02 | 0.00052 | A68~B35~C07~DR11 | 0.00078 | A26~B56~C01~DR07~DQ03 | 0.0009 | A01~B15~C06~DR03~DQ03~DP14 | 0.00113 |  |  | A26~B27~DR01 | 0.0008 |
| A30~B18~C05 | 0.00052 | A23~B55~C01~DR03 | 0.00078 | A03~B08~C15~DR15~DQ05 | 0.0009 | A02~B41~C03~DR13~DQ02~DP02 | 0.00113 |  |  | A26~B51~DR13 | 0.0008 |
| A30~B15~C03 | 0.00052 | A24~B35~C12~DR12 | 0.00078 | A02~B07~C07~DR08~DQ03 | 0.0009 | A02~B41~C06~DR03~DQ02~DP15 | 0.00113 |  |  | A30~B27~DR13 | 0.0008 |
| A68~B56~C01 | 0.00052 | A32~B51~C16~DR16 | 0.00078 | A02~B39~C12~DR08~DQ03 | 0.0009 | A02~B19~C07~DR03~DQ03~DP04 | 0.00113 |  |  | A01~B37~DR16 | 0.0008 |
| A69~B13~C07 | 0.00052 | A24~B07~C07~DR07 | 0.00078 | A33~B14~C06~DR11~DQ03 | 0.0009 | A31~B40~C03~DR04~DQ03~DP02 | 0.00113 |  |  | A23~B44~DR04 | 0.0008 |
| A36~B52~C12 | 0.00052 | A03~B18~C12~DR13 | 0.00078 | A26~B35~C14~DR04~DQ03 | 0.0009 | A31~B51~C14~DR11~DQ02~DP09 | 0.00113 |  |  | A11~B55~DR15 | 0.0008 |
| A01~B58~C04 | 0.00052 | A11~B38~C12~DR13 | 0.00078 | A24~B27~C01~DR11~DQ03 | 0.0009 | A32~B51~C01~DR03~DQ03~DP02 | 0.00113 |  |  | A03~B15~DR04 | 0.0008 |
| A33~B27~C02 | 0.00052 | A25~B27~C04~DR04 | 0.00078 | A32~B18~C12~DR11~DQ05 | 0.0009 | A02~B49~C07~DR07~DQ02~DP04 | 0.00113 |  |  | A24~B56~DR04 | 0.0008 |
| A30~B14~C08 | 0.00052 | A31~B48~C08~DR03 | 0.00078 | A24~B44~C04~DR15~DQ06 | 0.0009 | A03~B49~C07~DR11~DQ03~DP03 | 0.00113 |  |  | A01~B40~DR15 | 0.0008 |
| A33~B49~C07 | 0.00052 | A68~B40~C03~DR09 | 0.00078 | A30~B14~C05~DR01~DQ05 | 0.0009 | A03~B49~C07~DR07~DQ02~DP03 | 0.00113 |  |  | A02~B15~DR16 | 0.0008 |
| A23~B52~C12 | 0.00052 | A02~B51~C03~DR14 | 0.00078 | A29~B14~C08~DR01~DQ05 | 0.0009 | A31~B15~C01~DR04~DQ03~DP02 | 0.00113 |  |  | A25~B51~DR13 | 0.0008 |
| A02~B35~C15 | 0.00051 | A02~B35~C01~DR13 | 0.00078 | A29~B57~C18~DR01~DQ05 | 0.0009 | A02~B40~C07~DR13~DQ06~DP16 | 0.00113 |  |  | A26~B44~DR11 | 0.0007 |
| A31~B51~C16 | 0.0005 | A02~B46~C01~DR08 | 0.00078 | A68~B37~C06~DR13~DQ05 | 0.0009 | A30~B07~C02~DR15~DQ06~DP03 | 0.00113 |  |  | A24~B35~DR07 | 0.0007 |
| A30~B40~C02 | 0.0005 | A69~B13~C06~DR04 | 0.00078 | A11~B55~C01~DR03~DQ03 | 0.0009 | A29~B51~C07~DR07~DQ03~DP02 | 0.00113 |  |  | A03~B51~DR15 | 0.0007 |
| A02~B44~C15 | 0.00049 | A02~B27~C14~DR11 | 0.00078 | A24~B37~C12~DR12~DQ03 | 0.0009 | A69~B35~C12~DR14~DQ05~DP02 | 0.00113 |  |  | A24~B18~DR14 | 0.0007 |
| A26~B15~C01 | 0.00049 | A11~B08~C03~DR03 | 0.00078 | A01~B37~C06~DR07~DQ02 | 0.0009 | A32~B41~C17~DR11~DQ03~DP14 | 0.00113 |  |  | A23~B35~DR07 | 0.0007 |
| A68~B15~C03 | 0.00049 | A03~B51~C16~DR16 | 0.00078 | A24~B35~C02~DR03~DQ06 | 0.0009 | A30~B51~C15~DR11~DQ03~DP10 | 0.00113 |  |  | A02~B37~DR10 | 0.0007 |
| A26~B44~C04 | 0.00049 | A02~B51~C14~DR10 | 0.00078 | A01~B35~C06~DR01~DQ05 | 0.0009 | A03~B50~C04~DR15~DQ06~DP03 | 0.00113 |  |  | A03~B44~DR01 | 0.0007 |
| A30~B51~C15 | 0.00049 | A03~B08~C07~DR13 | 0.00078 | A29~B58~C15~DR04~DQ03 | 0.0009 | A23~B49~C07~DR14~DQ03~DP04 | 0.00113 |  |  | A68~B35~DR08 | 0.0007 |
| A11~B44~C16 | 0.00049 | A03~B07~C07~DR07 | 0.00078 | A23~B38~C12~DR11~DQ03 | 0.0009 | A01~B51~C16~DR13~DQ03~DP04 | 0.00113 |  |  | A03~B35~DR16 | 0.0007 |
| A24~B56~C01 | 0.00048 | A68~B35~C16~DR14 | 0.00077 | A01~B51~C01~DR11~DQ06 | 0.0009 | A30~B18~C07~DR11~DQ06~DP04 | 0.00113 |  |  | A11~B44~DR04 | 0.0007 |
| A02~B07~C15 | 0.00048 | A24~B40~C03~DR04 | 0.00077 | A29~B38~C12~DR11~DQ03 | 0.0009 | A32~B14~C08~DR13~DQ06~DP02 | 0.00113 |  |  | A01~B37~DR14 | 0.0007 |
| A68~B07~C07 | 0.00047 | A26~B35~C04~DR03 | 0.00076 | A11~B18~C12~DR13~DQ06 | 0.0009 | A30~B35~C04~DR08~DQ03~DP03 | 0.00113 |  |  | A02~B38~DR11 | 0.0007 |
| A11~B51~C01 | 0.00047 | A01~B55~C01~DR13 | 0.00076 | A33~B07~C07~DR03~DQ02 | 0.0009 | A68~B38~C12~DR07~DQ02~DP105 | 0.00113 |  |  | A02~B07~DR13 | 0.0007 |
| A31~B49~C07 | 0.00047 | A02~B44~C05~DR13 | 0.00075 | A01~B15~C03~DR13~DQ03 | 0.0009 | A68~B38~C12~DR07~DQ02~DP04 | 0.00113 |  |  | A24~B27~DR16 | 0.0007 |
| A68~B50~C06 | 0.00045 | A69~B07~C07~DR11 | 0.00074 | A02~B19~C07~DR03~DQ03 | 0.0009 | A03~B07~C07~DR03~DQ02~DP03 | 0.00113 |  |  | A01~B37~DR13 | 0.0007 |
| A26~B56~C01 | 0.00045 | A01~B15~C03~DR04 | 0.00074 | A31~B35~C04~DR04~DQ03 | 0.0009 | A11~B40~C03~DR04~DQ03~DP03 | 0.00113 |  |  | A31~B40~DR04 | 0.0007 |
| A25~B51~C15 | 0.00045 | A03~B35~C06~DR07 | 0.00073 | A26~B13~C06~DR07~DQ02 | 0.0009 | A26~B51~C14~DR11~DQ03~DP03 | 0.00113 |  |  | A11~B55~DR04 | 0.0007 |
| A02~B44~C01 | 0.00045 | A24~B51~C14~DR04 | 0.00073 | A31~B51~C01~DR11~DQ03 | 0.0009 | A29~B49~C07~DR11~DQ03~DP15 | 0.00113 |  |  | A03~B13~DR07 | 0.0007 |
| A69~B45~C04 | 0.00043 | A02~B07~C07~DR01 | 0.00073 | A03~B49~C07~DR11~DQ03 | 0.0009 | A01~B18~C07~DR13~DQ06~DP02 | 0.00113 |  |  | A03~B07~DR07 | 0.0007 |
| A25~B44~C05 | 0.00042 | A29~B07~C15~DR13 | 0.00072 | A03~B49~C07~DR07~DQ02 | 0.0009 | A30~B35~C06~DR16~DQ02~DP17 | 0.00113 |  |  | A32~B49~DR13 | 0.0007 |
| A33~B13~C06 | 0.00041 | A33~B35~C07~DR13 | 0.00072 | A02~B35~C01~DR04~DQ03 | 0.0009 | A11~B44~C04~DR14~DQ02~DP02 | 0.00113 |  |  | A68~B39~DR16 | 0.0007 |
| A02~B49~C12 | 0.00041 | A24~B18~C12~DR07 | 0.00072 | A02~B49~C07~DR10~DQ05 | 0.0009 | A31~B39~C07~DR07~DQ03~DP04 | 0.00113 |  |  | A26~B50~DR07 | 0.0007 |
| A11~B35~C15 | 0.0004 | A24~B15~C03~DR13 | 0.00072 | A30~B40~C02~DR13~DQ06 | 0.0009 | A01~B49~C06~DR13~DQ06~DP04 | 0.00113 |  |  | A26~B35~DR01 | 0.0007 |
| A02~B51~C06 | 0.0004 | A26~B38~C12~DR03 | 0.00071 | A02~B51~C07~DR07~DQ03 | 0.0009 | A01~B57~C07~DR14~DQ05~DP04 | 0.00113 |  |  | A11~B51~DR03 | 0.0007 |
| A30~B35~C12 | 0.0004 | A24~B50~C06~DR03 | 0.00071 | A69~B35~C12~DR14~DQ05 | 0.0009 | A68~B51~C16~DR11~DQ04~DP04 | 0.00113 |  |  | A02~B55~DR14 | 0.0007 |
| A31~B52~C12 | 0.00039 | A23~B35~C06~DR11 | 0.00069 | A30~B51~C15~DR11~DQ03 | 0.0009 | A24~B48~C08~DR12~DQ06~DP02 | 0.00113 |  |  | A24~B55~DR15 | 0.0007 |
| A01~B27~C01 | 0.00039 | A03~B51~C15~DR15 | 0.00069 | A33~B14~C08~DR13~DQ06 | 0.0009 | A01~B15~C07~DR04~DQ04~DP02 | 0.00113 |  |  | A01~B15~DR09 | 0.0007 |
| A01~B55~C03 | 0.00039 | A02~B35~C04~DR07 | 0.00069 | A30~B35~C04~DR08~DQ03 | 0.0009 | A01~B15~C07~DR13~DQ06~DP04 | 0.00113 |  |  | A02~B15~DR14 | 0.0007 |
| A68~B13~C06 | 0.00038 | A01~B18~C07~DR04 | 0.00068 | A11~B40~C03~DR04~DQ03 | 0.0009 | A02~B35~C12~DR04~DQ03~DP11 | 0.00113 |  |  | A02~B27~DR03 | 0.0007 |
| A29~B18~C07 | 0.00037 | A02~B15~C03~DR11 | 0.00067 | A30~B35~C06~DR16~DQ05 | 0.0009 | A24~B55~C01~DR03~DQ02~DP03 | 0.00113 |  |  | A11~B35~DR08 | 0.0007 |
| A26~B15~C12 | 0.00037 | A02~B50~C06~DR03 | 0.00067 | A31~B39~C07~DR14~DQ03 | 0.0009 | A26~B35~C12~DR03~DQ02~DP04 | 0.00113 |  |  | A26~B38~DR07 | 0.0007 |
| A26~B15~C04 | 0.00037 | A03~B41~C17~DR13 | 0.00066 | A31~B15~C07~DR15~DQ05 | 0.0009 | A29~B35~C04~DR15~DQ06~DP14 | 0.00113 |  |  | A11~B55~DR16 | 0.0007 |
| A32~B50~C06 | 0.00036 | A24~B44~C07~DR16 | 0.00065 | A01~B57~C06~DR13~DQ06 | 0.0009 | A32~B18~C02~DR11~DQ03~DP04 | 0.00113 |  |  | A01~B41~DR03 | 0.0007 |
| A25~B51~C16 | 0.00036 | A11~B49~C07~DR15 | 0.00065 | A24~B51~C16~DR11~DQ04 | 0.0009 | A24~B44~C02~DR16~DQ05~DP04 | 0.00113 |  |  | A02~B39~DR12 | 0.0007 |
| A11~B18~C07 | 0.00036 | A01~B51~C15~DR13 | 0.00065 | A24~B48~C08~DR12~DQ06 | 0.0009 | A24~B35~C12~DR09~DQ03~DP03 | 0.00113 |  |  | A24~B14~DR01 | 0.0007 |
| A11~B44~C03 | 0.00036 | A01~B18~C07~DR11 | 0.00064 | A01~B15~C07~DR04~DQ04 | 0.0009 | A68~B58~C07~DR08~DQ04~DP03 | 0.00113 |  |  | A11~B38~DR07 | 0.0007 |
| A24~B40~C15 | 0.00036 | A02~B08~C07~DR01 | 0.00064 | A01~B15~C07~DR13~DQ06 | 0.0009 | A02~B40~C08~DR14~DQ06~DP10 | 0.00113 |  |  | A01~B15~DR11 | 0.0006 |
| A69~B35~C04 | 0.00036 | A26~B35~C04~DR04 | 0.00064 | A03~B35~C12~DR04~DQ03 | 0.0009 | A11~B15~C03~DR13~DQ05~DP02 | 0.00113 |  |  | A03~B44~DR11 | 0.0006 |
| A32~B07~C15 | 0.00036 | A03~B38~C12~DR11 | 0.00064 | A24~B55~C01~DR03~DQ02 | 0.0009 | A30~B18~C08~DR11~DQ03~DP02 | 0.00113 |  |  | A24~B18~DR07 | 0.0006 |
| A02~B38~C07 | 0.00035 | A26~B51~C15~DR14 | 0.00064 | A26~B35~C12~DR03~DQ02 | 0.0009 | A01~B51~C08~DR11~DQ03~DP02 | 0.00113 |  |  | A29~B38~DR04 | 0.0006 |
| A32~B51~C12 | 0.00035 | A31~B44~C05~DR04 | 0.00063 | A29~B35~C04~DR15~DQ06 | 0.0009 | A33~B14~C14~DR03~DQ02~DP02 | 0.00113 |  |  | A68~B35~DR04 | 0.0006 |
| A30~B37~C06 | 0.00035 | A11~B15~C07~DR13 | 0.00063 | A32~B18~C02~DR11~DQ03 | 0.0009 | A23~B49~C07~DR13~DQ03~DP04 | 0.00113 |  |  | A24~B44~DR13 | 0.0006 |
| A68~B55~C01 | 0.00035 | A32~B15~C01~DR14 | 0.00063 | A01~B57~C14~DR03~DQ05 | 0.0009 | A02~B18~C07~DR03~DQ02~DP03 | 0.00113 |  |  | A01~B37~DR07 | 0.0006 |
| A11~B37~C06 | 0.00033 | A02~B50~C06~DR04 | 0.00063 | A03~B18~C12~DR09~DQ03 | 0.0009 | A24~B40~C03~DR03~DQ02~DP04 | 0.00113 |  |  | A03~B41~DR04 | 0.0006 |
| A02~B48~C03 | 0.00033 | A01~B49~C07~DR07 | 0.00063 | A03~B35~C12~DR09~DQ03 | 0.0009 | A02~B40~C07~DR03~DQ02~DP04 | 0.00113 |  |  | A01~B27~DR07 | 0.0006 |
| A30~B07~C14 | 0.00033 | A03~B14~C08~DR15 | 0.00062 | A68~B58~C07~DR08~DQ04 | 0.0009 | A29~B45~C06~DR11~DQ03~DP03 | 0.00113 |  |  | A01~B27~DR11 | 0.0006 |
| A24~B51~C07 | 0.00033 | A01~B38~C12~DR04 | 0.00062 | A11~B15~C08~DR14~DQ05 | 0.0009 | A03~B27~C12~DR15~DQ06~DP15 | 0.00113 |  |  | A11~B55~DR11 | 0.0006 |
| A26~B27~C02 | 0.00033 | A03~B50~C06~DR04 | 0.00062 | A30~B18~C08~DR11~DQ03 | 0.0009 | A24~B40~C04~DR04~DQ03~DP04 | 0.00113 |  |  | A01~B35~DR16 | 0.0006 |
| A68~B18~C12 | 0.00032 | A01~B37~C06~DR04 | 0.00062 | A01~B14~C08~DR03~DQ02 | 0.0009 | A26~B15~C02~DR13~DQ06~DP04 | 0.00113 |  |  | A11~B07~DR15 | 0.0006 |
| A24~B08~C03 | 0.00032 | A03~B57~C06~DR01 | 0.00061 | A01~B51~C14~DR11~DQ03 | 0.0009 | A03~B52~C12~DR04~DQ03~DP02 | 0.00113 |  |  | A68~B35~DR16 | 0.0006 |
| A68~B57~C06 | 0.00032 | A11~B49~C07~DR13 | 0.00061 | A23~B49~C07~DR13~DQ03 | 0.0009 | A11~B44~C03~DR11~DQ02~DP02 | 0.00113 |  |  | A11~B35~DR16 | 0.0006 |
| A32~B40~C03 | 0.00032 | A03~B27~C02~DR11 | 0.00061 | A24~B40~C03~DR03~DQ02 | 0.0009 | A24~B50~C06~DR03~DQ02~DP02 | 0.00113 |  |  | A26~B35~DR14 | 0.0006 |
| A24~B55~C07 | 0.00032 | A11~B51~C06~DR11 | 0.00061 | A29~B45~C06~DR11~DQ03 | 0.0009 | A03~B38~C12~DR13~DQ06~DP09 | 0.00113 |  |  | A24~B38~DR13 | 0.0006 |
| A68~B52~C12 | 0.00032 | A02~B51~C14~DR14 | 0.0006 | A03~B27~C12~DR15~DQ06 | 0.0009 | A24~B13~C16~DR03~DQ02~DP04 | 0.00113 |  |  | A24~B40~DR07 | 0.0006 |
| A26~B15~C03 | 0.00031 | A11~B15~C07~DR15 | 0.00059 | A03~B52~C12~DR04~DQ03 | 0.0009 | A24~B13~C07~DR03~DQ02~DP04 | 0.00113 |  |  | A02~B27~DR15 | 0.0006 |
| A33~B38~C12 | 0.00031 | A11~B51~C14~DR03 | 0.00059 | A11~B44~C03~DR11~DQ03 | 0.0009 | A66~B52~C07~DR07~DQ02~DP02 | 0.00113 |  |  | A23~B49~DR14 | 0.0006 |
| A68~B44~C05 | 0.00031 | A29~B07~C15~DR11 | 0.00059 | A24~B50~C06~DR04~DQ03 | 0.0009 | A02~B38~C04~DR11~DQ06~DP04 | 0.00113 |  |  | A03~B44~DR07 | 0.0006 |
| A03~B40~C04 | 0.00031 | A02~B27~C02~DR04 | 0.00059 | A24~B35~C16~DR11~DQ03 | 0.0009 | A24~B35~C07~DR13~DQ06~DP04 | 0.00113 |  |  | A03~B55~DR11 | 0.0006 |
| A02~B53~C04 | 0.00031 | A68~B39~C12~DR16 | 0.00059 | A66~B52~C07~DR07~DQ02 | 0.0009 | A11~B07~C07~DR10~DQ05~DP14 | 0.00113 |  |  | A01~B57~DR15 | 0.0006 |
| A23~B08~C06 | 0.00031 | A03~B08~C06~DR03 | 0.00059 | A03~B07~C07~DR07~DQ02 | 0.0009 | A31~B35~C04~DR10~DQ05~DP15 | 0.00113 |  |  | A01~B38~DR14 | 0.0006 |
| A24~B15~C12 | 0.00031 | A02~B50~C06~DR13 | 0.00058 | A03~B07~C07~DR07~DQ03 | 0.0009 | A32~B52~C12~DR11~DQ03~DP04 | 0.00113 |  |  | A68~B55~DR13 | 0.0006 |
| A23~B44~C05 | 0.00031 | A02~B38~C12~DR16 | 0.00058 | A24~B38~C07~DR11~DQ06 | 0.0009 | A68~B27~C02~DR11~DQ03~DP04 | 0.00113 |  |  | A24~B50~DR11 | 0.0006 |
| A02~B55~C04 | 0.00031 | A68~B52~C12~DR15 | 0.00058 | A11~B07~C07~DR10~DQ05 | 0.0009 | A26~B51~C14~DR13~DQ05~DP02 | 0.00113 |  |  | A24~B08~DR15 | 0.0006 |
| A11~B27~C12 | 0.0003 | A03~B18~C12~DR07 | 0.00058 | A31~B35~C04~DR10~DQ05 | 0.0009 | A68~B51~C12~DR14~DQ06~DP04 | 0.00113 |  |  | A01~B08~DR13 | 0.0006 |
| A01~B53~C04 | 0.0003 | A01~B35~C04~DR04 | 0.00058 | A32~B52~C12~DR11~DQ03 | 0.0009 | A11~B41~C07~DR11~DQ03~DP02 | 0.00113 |  |  | A11~B35~DR12 | 0.0006 |
| A02~B41~C07 | 0.0003 | A02~B35~C12~DR04 | 0.00058 | A68~B27~C02~DR11~DQ03 | 0.0009 | A30~B51~C16~DR16~DQ05~DP503 | 0.00113 |  |  | A32~B15~DR11 | 0.0006 |
| A25~B35~C04 | 0.0003 | A24~B35~C12~DR13 | 0.00057 | A02~B50~C06~DR04~DQ03 | 0.0009 | A02~B51~C03~DR11~DQ03~DP05 | 0.00113 |  |  | A03~B41~DR03 | 0.0006 |
| A02~B40~C08 | 0.0003 | A02~B27~C02~DR08 | 0.00057 | A11~B40~C07~DR11~DQ03 | 0.0009 | A24~B15~C16~DR16~DQ03~DP14 | 0.00113 |  |  | A02~B14~DR04 | 0.0006 |
| A33~B51~C15 | 0.0003 | A03~B18~C07~DR16 | 0.00057 | A30~B51~C16~DR16~DQ05 | 0.0009 | A01~B44~C05~DR12~DQ03~DP04 | 0.00113 |  |  | A33~B44~DR13 | 0.0006 |
| A23~B41~C17 | 0.0003 | A02~B51~C16~DR09 | 0.00057 | A24~B15~C03~DR16~DQ03 | 0.0009 | A02~B51~C03~DR12~DQ03~DP23 | 0.00113 |  |  | A30~B44~DR07 | 0.0006 |
| A02~B27~C12 | 0.00029 | A24~B15~C03~DR11 | 0.00056 | A02~B35~C05~DR11~DQ03 | 0.0009 | A24~B40~C15~DR12~DQ03~DP04 | 0.00113 |  |  | A01~B49~DR07 | 0.0006 |
| A11~B08~C04 | 0.00029 | A01~B08~C06~DR03 | 0.00056 | A01~B44~C05~DR12~DQ03 | 0.0009 | A11~B27~C02~DR10~DQ05~DP03 | 0.00113 |  |  | A29~B44~DR11 | 0.0006 |
| A02~B40~C07 | 0.00029 | A01~B51~C14~DR13 | 0.00056 | A02~B15~C03~DR07~DQ02 | 0.0009 | A32~B40~C02~DR16~DQ05~DP03 | 0.00113 |  |  | A02~B44~DR12 | 0.0006 |
| A26~B35~C14 | 0.00029 | A01~B35~C04~DR16 | 0.00056 | A02~B40~C02~DR07~DQ02 | 0.0009 | A24~B44~C04~DR01~DQ05~DP04 | 0.00113 |  |  | A03~B07~DR16 | 0.0006 |
| A32~B51~C04 | 0.00029 | A01~B40~C03~DR14 | 0.00056 | A24~B44~C02~DR01~DQ05 | 0.0009 | A03~B18~C04~DR10~DQ05~DP105 | 0.00113 |  |  | A11~B49~DR15 | 0.0006 |
| A02~B13~C01 | 0.00029 | A02~B40~C03~DR15 | 0.00056 | A03~B18~C12~DR10~DQ05 | 0.0009 | A01~B35~C03~DR07~DQ02~DP02 | 0.00113 |  |  | A23~B49~DR03 | 0.0006 |
| A32~B27~C07 | 0.00029 | A03~B50~C06~DR07 | 0.00055 | A68~B35~C03~DR03~DQ02 | 0.0009 | A68~B35~C04~DR03~DQ02~DP04 | 0.00113 |  |  | A03~B35~DR10 | 0.0006 |
| A26~B14~C08 | 0.00029 | A03~B44~C05~DR11 | 0.00055 | A24~B51~C15~DR04~DQ03 | 0.0009 | A24~B40~C03~DR11~DQ03~DP04 | 0.00113 |  |  | A03~B13~DR01 | 0.0006 |
| A30~B13~C12 | 0.00028 | A11~B51~C15~DR04 | 0.00055 | A24~B40~C03~DR13~DQ06 | 0.0009 | A11~B49~C07~DR15~DQ06~DP13 | 0.00113 |  |  | A24~B44~DR01 | 0.0006 |
| A30~B07~C07 | 0.00028 | A11~B35~C04~DR14 | 0.00055 | A32~B15~C12~DR04~DQ03 | 0.0009 | A68~B51~C15~DR04~DQ03~DP04 | 0.00113 |  |  | A01~B49~DR04 | 0.0006 |
| A24~B35~C02 | 0.00028 | A24~B55~C01~DR11 | 0.00055 | A02~B48~C08~DR04~DQ03 | 0.0009 | A32~B15~C12~DR04~DQ03~DP02 | 0.00113 |  |  | A29~B35~DR15 | 0.0006 |
| A30~B18~C07 | 0.00028 | A68~B40~C15~DR14 | 0.00055 | A02~B27~C02~DR15~DQ06 | 0.0009 | A29~B07~C15~DR10~DQ05~DP04 | 0.00113 |  |  | A26~B51~DR15 | 0.0006 |
| A30~B41~C16 | 0.00028 | A24~B39~C12~DR16 | 0.00055 | A24~B44~C02~DR09~DQ03 | 0.0009 | A03~B49~C07~DR11~DQ04~DP09 | 0.00113 |  |  | A02~B55~DR16 | 0.0006 |
| A02~B35~C17 | 0.00028 | A11~B55~C01~DR13 | 0.00054 | A26~B40~C03~DR11~DQ03 | 0.0009 | A03~B49~C07~DR11~DQ04~DP04 | 0.00113 |  |  | A24~B52~DR01 | 0.0006 |
| A01~B51~C04 | 0.00028 | A01~B51~C14~DR11 | 0.00054 | A32~B07~C15~DR13~DQ03 | 0.0009 | A02~B48~C08~DR04~DQ03~DP04 | 0.00113 |  |  | A03~B51~DR16 | 0.0006 |
| A23~B51~C14 | 0.00028 | A02~B15~C07~DR11 | 0.00054 | A23~B37~C06~DR10~DQ05 | 0.0009 | A31~B27~C02~DR15~DQ06~DP03 | 0.00113 |  |  | A02~B27~DR01 | 0.0006 |
| A24~B58~C07 | 0.00028 | A24~B44~C05~DR10 | 0.00054 | A33~B49~C07~DR15~DQ06 | 0.0009 | A03~B07~C07~DR07~DQ03~DP09 | 0.00113 |  |  | A26~B55~DR04 | 0.0006 |
| A02~B37~C06 | 0.00028 | A02~B51~C16~DR01 | 0.00054 | A01~B44~C07~DR03~DQ02 | 0.0009 | A26~B44~C02~DR09~DQ03~DP04 | 0.00113 |  |  | A30~B38~DR13 | 0.0006 |
| A03~B40~C02 | 0.00027 | A03~B07~C07~DR14 | 0.00054 | A30~B15~C07~DR03~DQ02 | 0.0009 | A32~B07~C15~DR13~DQ03~DP03 | 0.00113 |  |  | A26~B38~DR03 | 0.0006 |
| A24~B55~C02 | 0.00027 | A02~B07~C07~DR09 | 0.00053 | A03~B18~C07~DR11~DQ02 | 0.0009 | A23~B37~C06~DR13~DQ06~DP09 | 0.00113 |  |  | A24~B40~DR03 | 0.0006 |
| A02~B18~C16 | 0.00027 | A30~B39~C12~DR13 | 0.00053 | A30~B27~C02~DR16~DQ05 | 0.0009 | A32~B49~C07~DR15~DQ06~DP01 | 0.00113 |  |  | A23~B44~DR13 | 0.0006 |
| A11~B35~C14 | 0.00027 | A24~B57~C06~DR13 | 0.00053 | A11~B39~C12~DR03~DQ02 | 0.0009 | A01~B35~C04~DR14~DQ05~DP02 | 0.00113 |  |  | A32~B44~DR12 | 0.0006 |
| A24~B35~C16 | 0.00027 | A03~B35~C12~DR11 | 0.00053 | A03~B18~C12~DR11~DQ04 | 0.0009 | A01~B44~C07~DR03~DQ02~DP04 | 0.00113 |  |  | A01~B40~DR04 | 0.0006 |
| A01~B18~C01 | 0.00027 | A32~B18~C07~DR11 | 0.00052 | A25~B18~C07~DR11~DQ03 | 0.0009 | A30~B15~C07~DR03~DQ02~DP04 | 0.00113 |  |  | A66~B18~DR16 | 0.0006 |
| A01~B07~C04 | 0.00027 | A02~B38~C12~DR11 | 0.00052 | A11~B18~C07~DR10~DQ05 | 0.0009 | A33~B14~C08~DR01~DQ05~DP09 | 0.00113 |  |  | A11~B55~DR13 | 0.0006 |
| A32~B35~C07 | 0.00027 | A01~B35~C06~DR15 | 0.00052 | A11~B27~C02~DR03~DQ02 | 0.0009 | A03~B18~C07~DR07~DQ02~DP04 | 0.00113 |  |  | A26~B55~DR13 | 0.0006 |
| A33~B55~C01 | 0.00027 | A11~B52~C12~DR13 | 0.00052 | A11~B55~C01~DR03~DQ02 | 0.0009 | A30~B58~C02~DR13~DQ05~DP04 | 0.00113 |  |  | A69~B35~DR11 | 0.0006 |
| A25~B07~C07 | 0.00027 | A03~B14~C08~DR01 | 0.00052 | A29~B51~C15~DR07~DQ06 | 0.0009 | A33~B27~C03~DR16~DQ06~DP02 | 0.00113 |  |  | A26~B55~DR14 | 0.0006 |
| A26~B51~C07 | 0.00027 | A24~B38~C12~DR11 | 0.00052 | A24~B55~C01~DR08~DQ04 | 0.0009 | A02~B35~C01~DR04~DQ03~DP03 | 0.00113 |  |  | A25~B18~DR13 | 0.0006 |
| A29~B51~C14 | 0.00026 | A02~B15~C01~DR09 | 0.00052 | A31~B49~C07~DR16~DQ05 | 0.0009 | A32~B39~C12~DR03~DQ02~DP02 | 0.00113 |  |  | A68~B40~DR13 | 0.0006 |
| A02~B50~C16 | 0.00026 | A68~B27~C02~DR15 | 0.00052 | A03~B57~C06~DR13~DQ06 | 0.0009 | A02~B51~C08~DR04~DQ03~DP14 | 0.00113 |  |  | A68~B53~DR01 | 0.0006 |
| A23~B15~C03 | 0.00026 | A02~B07~C07~DR13 | 0.00052 | A24~B40~C12~DR16~DQ05 | 0.0009 | A30~B14~C14~DR04~DQ03~DP13 | 0.00113 |  |  | A24~B15~DR12 | 0.0006 |
| A25~B51~C06 | 0.00026 | A01~B45~C16~DR04 | 0.00052 | A02~B15~C12~DR08~DQ04 | 0.0009 | A30~B14~C08~DR04~DQ04~DP04 | 0.00113 |  |  | A03~B27~DR11 | 0.0005 |
| A33~B50~C06 | 0.00026 | A02~B51~C07~DR07 | 0.00052 | A32~B35~C15~DR15~DQ06 | 0.0009 | A01~B07~C07~DR07~DQ03~DP02 | 0.00113 |  |  | A02~B38~DR04 | 0.0005 |
| A02~B35~C16 | 0.00026 | A33~B14~C08~DR07 | 0.00052 | A23~B38~C12~DR04~DQ06 | 0.0009 | A24~B52~C07~DR11~DQ03~DP04 | 0.00113 |  |  | A02~B40~DR16 | 0.0005 |
| A23~B07~C15 | 0.00026 | A24~B55~C14~DR04 | 0.00052 | A02~B07~C03~DR11~DQ03 | 0.0009 | A01~B51~C14~DR15~DQ06~DP17 | 0.00113 |  |  | A02~B52~DR13 | 0.0005 |
| A29~B35~C16 | 0.00026 | A68~B18~C16~DR16 | 0.00052 | A02~B07~C12~DR15~DQ05 | 0.0009 | A32~B38~C12~DR11~DQ03~DP15 | 0.00113 |  |  | A30~B35~DR11 | 0.0005 |
| A02~B73~C15 | 0.00026 | A03~B55~C01~DR13 | 0.00052 | A31~B35~C16~DR07~DQ02 | 0.0009 | A01~B18~C12~DR11~DQ03~DP04 | 0.00113 |  |  | A02~B41~DR11 | 0.0005 |
| A68~B44~C04 | 0.00026 | A68~B51~C14~DR14 | 0.00052 | A02~B35~C12~DR13~DQ06 | 0.0009 | A02~B27~C02~DR13~DQ06~DP02 | 0.00113 |  |  | A68~B44~DR11 | 0.0005 |
| A23~B57~C06 | 0.00026 | A33~B35~C08~DR01 | 0.00052 | A02~B41~C17~DR13~DQ03 | 0.0009 | A01~B27~C07~DR04~DQ03~DP02 | 0.00113 |  |  | A23~B35~DR04 | 0.0005 |
| A32~B35~C15 | 0.00026 | A33~B13~C06~DR07 | 0.00052 | A01~B57~C06~DR01~DQ05 | 0.0009 | A11~B27~C02~DR03~DQ02~DP02 | 0.00113 |  |  | A32~B37~DR10 | 0.0005 |
| A03~B07~C05 | 0.00026 | A02~B27~C02~DR01 | 0.00052 | A02~B51~C14~DR16~DQ05 | 0.0009 | A11~B55~C01~DR03~DQ02~DP02 | 0.00113 |  |  | A02~B58~DR11 | 0.0005 |
| A03~B18~C15 | 0.00026 | A02~B44~C02~DR16 | 0.00052 | A68~B35~C04~DR15~DQ06 | 0.0009 | A02~B51~C15~DR13~DQ06~DP13 | 0.00113 |  |  | A03~B14~DR01 | 0.0005 |
| A02~B57~C07 | 0.00026 | A24~B51~C07~DR04 | 0.00052 | A30~B07~C07~DR04~DQ03 | 0.0009 | A29~B51~C16~DR07~DQ06~DP04 | 0.00113 |  |  | A02~B39~DR11 | 0.0005 |
| A11~B55~C02 | 0.00026 | A32~B49~C07~DR10 | 0.00052 | A66~B49~C17~DR15~DQ06 | 0.0009 | A24~B55~C01~DR08~DQ04~DP04 | 0.00113 |  |  | A02~B18~DR10 | 0.0005 |
| A25~B52~C12 | 0.00026 | A01~B27~C01~DR01 | 0.00052 | A26~B35~C04~DR10~DQ05 | 0.0009 | A11~B44~C12~DR11~DQ03~DP105 | 0.00113 |  |  | A26~B38~DR16 | 0.0005 |
| A03~B55~C12 | 0.00026 | A02~B18~C02~DR04 | 0.00052 | A69~B18~C07~DR11~DQ03 | 0.0009 | A23~B52~C04~DR07~DQ02~DP14 | 0.00113 |  |  | A02~B46~DR08 | 0.0005 |
| A01~B58~C03 | 0.00026 | A32~B49~C07~DR04 | 0.00052 | A24~B41~C17~DR15~DQ06 | 0.0009 | A31~B49~C07~DR16~DQ05~DP02 | 0.00113 |  |  | A11~B27~DR15 | 0.0005 |
| A03~B50~C04 | 0.00026 | A30~B38~C12~DR16 | 0.00052 | A24~B35~C04~DR04~DQ06 | 0.0009 | A03~B57~C06~DR13~DQ06~DP13 | 0.00113 |  |  | A11~B27~DR11 | 0.0005 |
| A01~B27~C07 | 0.00026 | A24~B48~C08~DR04 | 0.00052 | A24~B55~C03~DR16~DQ05 | 0.0009 | A03~B40~C04~DR01~DQ05~DP04 | 0.00113 |  |  | A11~B52~DR03 | 0.0005 |
| A30~B08~C02 | 0.00026 | A31~B40~C03~DR04 | 0.00052 | A03~B45~C16~DR11~DQ03 | 0.0009 | A24~B35~C12~DR16~DQ05~DP04 | 0.00113 |  |  | A69~B51~DR11 | 0.0005 |
| A30~B13~C04 | 0.00026 | A30~B53~C04~DR15 | 0.00052 | A69~B18~C07~DR15~DQ06 | 0.0009 | A03~B15~C15~DR08~DQ04~DP03 | 0.00113 |  |  | A24~B57~DR07 | 0.0005 |
| A68~B18~C07 | 0.00026 | A11~B18~C07~DR14 | 0.00052 | A68~B44~C14~DR08~DQ04 | 0.0009 | A03~B15~C12~DR08~DQ04~DP03 | 0.00113 |  |  | A02~B44~DR10 | 0.0005 |
| A01~B35~C14 | 0.00026 | A23~B51~C14~DR04 | 0.00052 | A02~B15~C01~DR12~DQ03 | 0.0009 | A30~B35~C15~DR15~DQ06~DP04 | 0.00113 |  |  | A01~B38~DR13 | 0.0005 |
| A24~B51~C05 | 0.00026 | A24~B44~C07~DR11 | 0.00052 | A24~B39~C07~DR13~DQ03 | 0.0009 | A23~B38~C12~DR04~DQ06~DP04 | 0.00113 |  |  | A02~B52~DR14 | 0.0005 |
| A01~B57~C14 | 0.00026 | A02~B40~C02~DR04 | 0.00052 | A26~B38~C12~DR13~DQ06 | 0.0009 | A01~B44~C04~DR04~DQ03~DP02 | 0.00113 |  |  | A02~B50~DR04 | 0.0005 |
| A11~B50~C04 | 0.00026 | A02~B35~C18~DR15 | 0.00052 | A68~B40~C03~DR07~DQ03 | 0.0009 | A02~B07~C15~DR08~DQ03~DP04 | 0.00113 |  |  | A31~B51~DR11 | 0.0005 |
| A01~B55~C04 | 0.00026 | A11~B40~C07~DR12 | 0.00052 | A68~B57~C07~DR13~DQ06 | 0.0009 | A02~B15~C03~DR11~DQ04~DP02 | 0.00113 |  |  | A33~B44~DR11 | 0.0005 |
| A02~B60~C03 | 0.00026 | A32~B35~C12~DR01 | 0.00052 | A02~B07~C14~DR11~DQ03 | 0.0009 | A68~B35~C04~DR11~DQ03~DP04 | 0.00113 |  |  | A02~B40~DR12 | 0.0005 |
| A01~B55~C06 | 0.00026 | A24~B35~C01~DR12 | 0.00052 | A03~B35~C16~DR16~DQ05 | 0.0009 | A24~B49~C12~DR11~DQ03~DP04 | 0.00113 |  |  | A02~B18~DR12 | 0.0005 |
| A66~B14~C17 | 0.00026 | A23~B38~C12~DR11 | 0.00052 | A68~B13~C02~DR04~DQ02 | 0.0009 | A02~B40~C03~DR13~DQ06~DP04 | 0.00113 |  |  | A02~B54~DR04 | 0.0005 |
| A68~B51~C06 | 0.00026 | A24~B52~C12~DR14 | 0.00052 | A03~B35~C15~DR11~DQ03 | 0.0009 | A02~B40~C03~DR07~DQ02~DP02 | 0.00113 |  |  | A11~B27~DR04 | 0.0005 |
| A03~B24~C04 | 0.00026 | A69~B45~C04~DR11 | 0.00052 | A26~B15~C01~DR13~DQ06 | 0.0009 | A02~B35~C12~DR13~DQ06~DP04 | 0.00113 |  |  | A32~B27~DR04 | 0.0005 |
| A24~B03~C07 | 0.00026 | A30~B38~C12~DR08 | 0.00052 | A26~B38~C12~DR15~DQ05 | 0.0009 | A03~B35~C04~DR01~DQ05~DP01 | 0.00113 |  |  | A68~B07~DR15 | 0.0005 |
| A23~B50~C07 | 0.00026 | A30~B38~C12~DR13 | 0.00052 | A01~B07~C07~DR10~DQ05 | 0.0009 | A26~B51~C14~DR16~DQ05~DP04 | 0.00113 |  |  | A03~B35~DR08 | 0.0005 |
| A03~B55~C05 | 0.00026 | A11~B40~C14~DR04 | 0.00052 | A24~B14~C08~DR03~DQ02 | 0.0009 | A68~B50~C04~DR15~DQ06~DP04 | 0.00113 |  |  | A03~B40~DR07 | 0.0005 |
| A68~B50~C07 | 0.00026 | A03~B54~C01~DR04 | 0.00052 | A03~B51~C07~DR10~DQ05 | 0.0009 | A11~B07~C07~DR08~DQ03~DP04 | 0.00113 |  |  | A03~B07~DR03 | 0.0005 |
| A32~B37~C01 | 0.00026 | A03~B18~C06~DR10 | 0.00052 | A02~B35~C06~DR14~DQ05 | 0.0009 | A66~B49~C17~DR15~DQ06~DP03 | 0.00113 |  |  | A03~B50~DR04 | 0.0005 |
| A68~B49~C07 | 0.00026 | A03~B08~C07~DR11 | 0.00052 | A29~B07~C07~DR15~DQ03 | 0.0009 | A26~B35~C04~DR10~DQ05~DP03 | 0.00113 |  |  | A01~B51~DR07 | 0.0005 |
| A23~B27~C15 | 0.00026 | A11~B35~C07~DR13 | 0.00052 | A02~B58~C07~DR16~DQ05 | 0.0009 | A02~B44~C16~DR03~DQ02~DP11 | 0.00113 |  |  | A01~B51~DR04 | 0.0005 |
| A31~B58~C07 | 0.00026 | A33~B44~C07~DR07 | 0.00052 | A02~B14~C08~DR15~DQ05 | 0.0009 | A24~B41~C17~DR15~DQ06~DP04 | 0.00113 |  |  | A03~B07~DR14 | 0.0005 |
| A11~B54~C07 | 0.00026 | A29~B35~C04~DR01 | 0.00052 | A26~B15~C12~DR14~DQ05 | 0.0009 | A02~B51~C03~DR13~DQ05~DP02 | 0.00113 |  |  | A29~B18~DR11 | 0.0005 |
| A01~B35~C08 | 0.00026 | A02~B55~C03~DR07 | 0.00052 | A24~B15~C06~DR04~DQ04 | 0.0009 | A24~B55~C16~DR16~DQ06~DP02 | 0.00113 |  |  | A02~B55~DR07 | 0.0005 |
| A26~B44~C12 | 0.00026 | A02~B27~C01~DR03 | 0.00052 | A32~B51~C16~DR03~DQ02 | 0.0009 | A03~B45~C16~DR11~DQ03~DP02 | 0.00113 |  |  | A02~B14~DR07 | 0.0005 |
| A25~B51~C14 | 0.00026 | A11~B51~C04~DR16 | 0.00052 | A24~B38~C12~DR10~DQ03 | 0.0009 | A23~B49~C07~DR03~DQ02~DP23 | 0.00113 |  |  | A03~B18~DR07 | 0.0005 |
| A33~B15~C08 | 0.00026 | A11~B51~C01~DR14 | 0.00052 | A25~B35~C12~DR13~DQ06 | 0.0009 | A02~B44~C14~DR15~DQ04~DP02 | 0.00113 |  |  | A24~B27~DR13 | 0.0005 |
| A69~B40~C03 | 0.00026 | A29~B18~C07~DR07 | 0.00052 | A32~B51~C16~DR07~DQ02 | 0.0009 | A68~B27~C02~DR08~DQ06~DP04 | 0.00113 |  |  | A03~B35~DR03 | 0.0005 |
| A68~B40~C01 | 0.00026 | A03~B55~C12~DR14 | 0.00052 | A32~B07~C12~DR14~DQ05 | 0.0009 | A02~B14~C08~DR01~DQ05~DP02 | 0.00113 |  |  | A01~B41~DR10 | 0.0005 |
| A11~B13~C08 | 0.00026 | A24~B15~C03~DR16 | 0.00052 | A26~B58~C07~DR03~DQ02 | 0.0009 | A24~B15~C01~DR12~DQ03~DP01 | 0.00113 |  |  | A26~B15~DR11 | 0.0005 |
| A68~B44~C12 | 0.00026 | A31~B14~C08~DR01 | 0.00052 | A30~B38~C06~DR15~DQ06 | 0.0009 | A02~B39~C07~DR13~DQ03~DP04 | 0.00113 |  |  | A11~B51~DR09 | 0.0005 |
| A33~B44~C14 | 0.00026 | A26~B44~C14~DR04 | 0.00052 | A11~B52~C12~DR08~DQ04 | 0.0009 | A02~B40~C03~DR07~DQ03~DP13 | 0.00113 |  |  | A02~B40~DR14 | 0.0005 |
| A31~B40~C07 | 0.00026 | A24~B08~C07~DR08 | 0.00052 | A02~B27~C02~DR07~DQ02 | 0.0009 | A68~B57~C07~DR13~DQ06~DP04 | 0.00113 |  |  | A24~B44~DR03 | 0.0005 |
| A66~B40~C17 | 0.00026 | A69~B35~C12~DR14 | 0.00052 | A33~B49~C07~DR03~DQ02 | 0.0009 | A30~B40~C03~DR13~DQ06~DP04 | 0.00113 |  |  | A32~B55~DR14 | 0.0005 |
| A68~B40~C08 | 0.00026 | A30~B38~C12~DR14 | 0.00052 | A30~B13~C04~DR15~DQ06 | 0.0009 | A30~B40~C03~DR11~DQ03~DP04 | 0.00113 |  |  | A68~B44~DR15 | 0.0005 |
| A03~B48~C03 | 0.00026 | A68~B35~C16~DR04 | 0.00052 | A26~B13~C17~DR13~DQ03 | 0.0009 | A01~B37~C06~DR07~DQ02~DP126 | 0.00113 |  |  | A24~B35~DR08 | 0.0005 |
| A33~B13~C07 | 0.00026 | A66~B18~C12~DR16 | 0.00052 | A03~B40~C03~DR16~DQ06 | 0.0009 | A02~B18~C15~DR09~DQ03~DP04 | 0.00113 |  |  | A01~B52~DR07 | 0.0005 |
| A02~B39~C01 | 0.00026 | A33~B51~C16~DR12 | 0.00052 | A02~B15~C03~DR01~DQ05 | 0.0009 | A01~B58~C06~DR13~DQ03~DP02 | 0.00113 |  |  | A01~B52~DR01 | 0.0005 |
| A66~B55~C01 | 0.00026 | A02~B49~C07~DR10 | 0.00052 | A03~B51~C16~DR03~DQ02 | 0.0009 | A33~B57~C03~DR13~DQ06~DP02 | 0.00113 |  |  | A11~B44~DR14 | 0.0005 |
| A32~B56~C01 | 0.00026 | A24~B15~C15~DR14 | 0.00052 | A26~B44~C14~DR04~DQ03 | 0.0009 | A01~B51~C14~DR13~DQ06~DP45 | 0.00113 |  |  | A11~B15~DR11 | 0.0005 |
| A33~B14~C05 | 0.00026 | A32~B35~C03~DR16 | 0.00052 | A11~B15~C07~DR13~DQ06 | 0.0009 | A02~B50~C07~DR07~DQ05~DP02 | 0.00113 |  |  | A01~B27~DR09 | 0.0005 |
| A23~B07~C14 | 0.00026 | A24~B44~C02~DR03 | 0.00052 | A02~B27~C02~DR09~DQ03 | 0.0009 | A02~B58~C06~DR16~DQ02~DP02 | 0.00113 |  |  | A31~B51~DR15 | 0.0005 |
| A29~B08~C04 | 0.00026 | A02~B49~C07~DR07 | 0.00052 | A02~B58~C06~DR03~DQ02 | 0.0009 | A02~B14~C08~DR15~DQ05~DP04 | 0.00113 |  |  | A69~B15~DR03 | 0.0005 |
| A68~B35~C15 | 0.00026 | A32~B27~C15~DR04 | 0.00052 | A01~B57~C18~DR16~DQ05 | 0.0009 | A02~B15~C12~DR14~DQ05~DP17 | 0.00113 |  |  | A69~B13~DR04 | 0.0005 |
| A11~B08~C02 | 0.00026 | A30~B35~C06~DR16 | 0.00052 | A26~B51~C07~DR13~DQ06 | 0.0009 | A26~B15~C03~DR15~DQ06~DP02 | 0.00113 |  |  | A02~B49~DR07 | 0.0005 |
| A33~B40~C02 | 0.00026 | A32~B55~C01~DR11 | 0.00052 | A11~B15~C07~DR11~DQ03 | 0.0009 | A24~B15~C06~DR04~DQ04~DP05 | 0.00113 |  |  | A31~B51~DR14 | 0.0005 |
| A11~B59~C07 | 0.00026 | A30~B49~C07~DR10 | 0.00052 | A26~B08~C07~DR07~DQ03 | 0.0009 | A03~B49~C07~DR16~DQ05~DP09 | 0.00113 |  |  | A02~B38~DR15 | 0.0005 |
| A33~B14~C06 | 0.00026 | A30~B40~C02~DR13 | 0.00052 | A01~B57~C06~DR04~DQ03 | 0.0009 | A03~B14~C16~DR13~DQ06~DP04 | 0.00113 |  |  | A30~B53~DR03 | 0.0005 |
| A26~B40~C07 | 0.00026 | A01~B41~C07~DR13 | 0.00052 | A29~B07~C02~DR15~DQ06 | 0.0009 | A23~B51~C08~DR08~DQ04~DP131 | 0.00113 |  |  | A32~B15~DR13 | 0.0005 |
| A01~B51~C02 | 0.00026 | A26~B53~C04~DR12 | 0.00052 | A31~B35~C04~DR03~DQ02 | 0.0009 | A01~B55~C01~DR14~DQ05~DP02 | 0.00113 |  |  | A31~B35~DR03 | 0.0005 |
| A02~B41~C16 | 0.00026 | A01~B44~C02~DR08 | 0.00052 | A02~B48~C08~DR12~DQ03 | 0.0009 | A02~B50~C06~DR07~DQ03~DP13 | 0.00113 |  |  | A11~B50~DR03 | 0.0005 |
| A30~B14~C05 | 0.00026 | A25~B44~C05~DR13 | 0.00052 | A30~B41~C17~DR04~DQ02 | 0.0009 | A02~B50~C06~DR11~DQ06~DP104 | 0.00113 |  |  | A26~B39~DR04 | 0.0005 |
| A24~B45~C06 | 0.00026 | A26~B44~C07~DR12 | 0.00052 | A68~B53~C04~DR13~DQ06 | 0.0009 | A23~B44~C04~DR15~DQ06~DP05 | 0.00113 |  |  | A02~B57~DR04 | 0.0005 |
| A33~B55~C17 | 0.00026 | A25~B51~C16~DR07 | 0.00052 | A26~B40~C03~DR04~DQ03 | 0.0009 | A30~B13~C04~DR03~DQ02~DP17 | 0.00113 |  |  | A03~B40~DR01 | 0.0005 |
| A22~B55~C01 | 0.00026 | A02~B15~C01~DR08 | 0.00052 | A33~B18~C12~DR14~DQ05 | 0.0009 | A23~B44~C04~DR07~DQ02~DP03 | 0.00113 |  |  | A26~B55~DR03 | 0.0005 |
| A24~B41~C02 | 0.00026 | A11~B15~C02~DR16 | 0.00052 | A32~B15~C03~DR15~DQ06 | 0.0009 | A01~B37~C06~DR04~DQ03~DP02 | 0.00113 |  |  | A29~B07~DR15 | 0.0005 |
| A29~B41~C01 | 0.00026 | A30~B18~C05~DR03 | 0.00052 | A11~B07~C07~DR15~DQ03 | 0.0009 | A24~B38~C12~DR10~DQ05~DP04 | 0.00113 |  |  | A02~B15~DR07 | 0.0005 |
| A11~B44~C08 | 0.00026 | A30~B15~C03~DR14 | 0.00052 | A03~B35~C12~DR07~DQ02 | 0.0009 | A25~B35~C12~DR13~DQ06~DP03 | 0.00113 |  |  | A03~B51~DR09 | 0.0005 |
| A30~B15~C12 | 0.00026 | A11~B27~C02~DR14 | 0.00052 | A33~B50~C06~DR03~DQ02 | 0.0009 | A02~B39~C16~DR16~DQ02~DP17 | 0.00113 |  |  | A33~B18~DR15 | 0.0005 |
| A68~B07~C08 | 0.00026 | A33~B58~C03~DR16 | 0.00052 | A03~B27~C02~DR03~DQ02 | 0.0009 | A32~B51~C12~DR07~DQ05~DP10 | 0.00113 |  |  | A26~B18~DR13 | 0.0005 |
| A32~B55~C07 | 0.00026 | A33~B44~C05~DR07 | 0.00052 | A33~B58~C03~DR01~DQ05 | 0.0009 | A24~B07~C07~DR14~DQ05~DP04 | 0.00113 |  |  | A32~B52~DR04 | 0.0005 |
| A23~B51~C04 | 0.00026 | A31~B39~C07~DR14 | 0.00052 | A11~B44~C16~DR01~DQ05 | 0.0009 | A32~B18~C12~DR01~DQ05~DP04 | 0.00113 |  |  | A68~B50~DR07 | 0.0005 |
| A26~B07~C08 | 0.00026 | A68~B56~C01~DR03 | 0.00052 | A03~B55~C04~DR16~DQ05 | 0.0009 | A01~B35~C04~DR01~DQ05~DP104 | 0.00113 |  |  | A31~B15~DR04 | 0.0005 |
| A30~B08~C04 | 0.00026 | A36~B52~C12~DR15 | 0.00052 | A32~B15~C03~DR16~DQ05 | 0.0009 | A26~B58~C07~DR03~DQ02~DP03 | 0.00113 |  |  | A30~B40~DR11 | 0.0005 |
| A69~B35~C01 | 0.00026 | A01~B51~C06~DR07 | 0.00052 | A02~B51~C04~DR14~DQ05 | 0.0009 | A11~B08~C07~DR03~DQ02~DP15 | 0.00113 |  |  | A69~B57~DR07 | 0.0005 |
| A23~B57~C18 | 0.00026 | A01~B58~C04~DR07 | 0.00052 | A02~B57~C06~DR04~DQ03 | 0.0009 | A30~B38~C06~DR15~DQ06~DP04 | 0.00113 |  |  | A01~B58~DR01 | 0.0005 |
| A03~B37~C03 | 0.00026 | A24~B48~C08~DR08 | 0.00052 | A11~B40~C07~DR12~DQ03 | 0.0009 | A01~B08~C07~DR03~DQ02~DP01 | 0.00113 |  |  | A30~B41~DR03 | 0.0005 |
| A33~B39~C12 | 0.00026 | A30~B14~C08~DR04 | 0.00052 | A24~B15~C03~DR12~DQ03 | 0.0009 | A24~B56~C01~DR11~DQ03~DP10 | 0.00113 |  |  | A24~B44~DR14 | 0.0004 |
| A11~B03~C12 | 0.00026 | A31~B52~C12~DR14 | 0.00052 | A11~B38~C12~DR10~DQ05 | 0.0009 | A02~B27~C02~DR07~DQ03~DP05 | 0.00113 |  |  | A24~B18~DR01 | 0.0004 |
| A02~B19~C07 | 0.00026 | A33~B07~C07~DR01 | 0.00052 | A03~B49~C07~DR01~DQ05 | 0.0009 | A02~B44~C05~DR04~DQ02~DP11 | 0.00113 |  |  | A02~B18~DR01 | 0.0004 |
| A29~B27~C07 | 0.00026 | A68~B15~C04~DR04 | 0.00052 | A24~B56~C01~DR11~DQ03 | 0.0009 | A02~B15~C03~DR01~DQ05~DP04 | 0.00113 |  |  | A29~B13~DR07 | 0.0004 |
| A25~B51~C12 | 0.00026 | A32~B51~C14~DR12 | 0.00052 | A30~B40~C03~DR13~DQ06 | 0.0009 | A02~B51~C16~DR03~DQ03~DP04 | 0.00113 |  |  | A02~B13~DR04 | 0.0004 |
| A68~B42~C17 | 0.00026 | A31~B15~C01~DR09 | 0.00052 | A02~B44~C05~DR01~DQ05 | 0.0009 | A03~B51~C16~DR04~DQ02~DP02 | 0.00113 |  |  | A02~B52~DR11 | 0.0004 |
| A30~B44~C17 | 0.00026 | A01~B14~C08~DR13 | 0.00052 | A30~B37~C06~DR11~DQ03 | 0.0009 | A02~B27~C02~DR09~DQ03~DP04 | 0.00113 |  |  | A31~B44~DR07 | 0.0004 |
| A68~B51~C12 | 0.00026 | A30~B49~C07~DR15 | 0.00052 | A11~B39~C12~DR09~DQ03 | 0.0009 | A11~B41~C17~DR07~DQ03~DP131 | 0.00113 |  |  | A68~B51~DR07 | 0.0004 |
| A31~B07~C15 | 0.00026 | A68~B55~C01~DR13 | 0.00052 | A32~B51~C14~DR15~DQ05 | 0.0009 | A01~B57~C18~DR16~DQ05~DP13 | 0.00113 |  |  | A11~B15~DR15 | 0.0004 |
| A01~B07~C18 | 0.00026 | A24~B27~C02~DR01 | 0.00052 | A11~B52~C12~DR11~DQ03 | 0.0009 | A24~B37~C06~DR03~DQ02~DP04 | 0.00113 |  |  | A11~B52~DR11 | 0.0004 |
| A66~B13~C16 | 0.00026 | A24~B18~C12~DR16 | 0.00051 | A03~B50~C06~DR01~DQ05 | 0.00082 | A26~B35~C07~DR16~DQ05~DP04 | 0.00113 |  |  | A66~B13~DR07 | 0.0004 |
| A31~B35~C02 | 0.00026 | A02~B51~C07~DR04 | 0.00051 | A24~B18~C07~DR13~DQ06 | 0.00079 | A32~B51~C04~DR13~DQ06~DP04 | 0.00113 |  |  | A30~B13~DR04 | 0.0004 |
| A30~B50~C07 | 0.00026 | A01~B07~C07~DR07 | 0.00051 | A01~B15~C03~DR04~DQ03 | 0.00077 | A03~B51~C16~DR16~DQ05~DP03 | 0.00113 |  |  | A02~B07~DR04 | 0.0004 |
| A26~B40~C13 | 0.00026 | A11~B35~C12~DR14 | 0.00051 | A23~B49~C07~DR14~DQ05 | 0.00074 | A26~B08~C07~DR07~DQ03~DP04 | 0.00113 |  |  | A02~B41~DR15 | 0.0004 |
| A68~B39~C02 | 0.00026 | A26~B51~C14~DR15 | 0.00051 | A01~B44~C07~DR16~DQ05 | 0.00054 | A01~B57~C06~DR04~DQ03~DP02 | 0.00113 |  |  | A03~B27~DR04 | 0.0004 |
| A11~B55~C16 | 0.00026 | A01~B15~C07~DR04 | 0.00051 | A26~B15~C04~DR13~DQ03 | 0.00045 | A01~B27~C02~DR04~DQ06~DP04 | 0.00113 |  |  | A26~B44~DR13 | 0.0004 |
| A03~B35~C10 | 0.00026 | A02~B51~C12~DR04 | 0.0005 | A26~B15~C04~DR13~DQ06 | 0.00045 | A31~B35~C04~DR03~DQ02~DP04 | 0.00113 |  |  | A01~B37~DR04 | 0.0004 |
| A36~B35~C04 | 0.00026 | A11~B15~C03~DR11 | 0.0005 | A24~B40~C02~DR04~DQ03 | 0.00045 | A26~B38~C12~DR13~DQ06~DP02 | 0.00113 |  |  | A03~B58~DR04 | 0.0004 |
| A03~B18~C01 | 0.00026 | A26~B49~C07~DR01 | 0.0005 | A24~B40~C02~DR04~DQ06 | 0.00045 | A02~B48~C08~DR12~DQ03~DP02 | 0.00113 |  |  | A02~B07~DR10 | 0.0004 |
| A36~B52~C08 | 0.00026 | A32~B50~C06~DR15 | 0.0005 | A02~B51~C16~DR09~DQ03 | 0.00045 | A26~B55~C03~DR11~DQ02~DP04 | 0.00113 |  |  | A32~B27~DR07 | 0.0004 |
| A02~B55~C16 | 0.00026 | A11~B37~C06~DR13 | 0.00049 | A02~B27~C02~DR13~DQ06 | 0.00032 | A11~B18~C12~DR15~DQ06~DP02 | 0.00113 |  |  | A29~B07~DR11 | 0.0004 |
| A23~B15~C14 | 0.00026 | A03~B57~C06~DR03 | 0.00049 | A68~B18~C12~DR04~DQ03 | 0.0003 | A33~B35~C04~DR14~DQ05~DP04 | 0.00113 |  |  | A01~B39~DR11 | 0.0004 |
| A30~B18~C08 | 0.00026 | A01~B15~C03~DR13 | 0.00049 | A24~B51~C15~DR11~DQ03 | 0.00024 | A01~B35~C12~DR07~DQ02~DP09 | 0.00113 |  |  | A01~B50~DR01 | 0.0004 |
| A66~B52~C07 | 0.00026 | A31~B51~C15~DR15 | 0.00049 | A02~B40~C03~DR12~DQ03 | 0.00023 | A01~B27~C02~DR11~DQ03~DP104 | 0.00113 |  |  | A01~B51~DR16 | 0.0004 |
| A26~B44~C02 | 0.00026 | A24~B35~C03~DR04 | 0.00048 | A24~B51~C15~DR12~DQ03 | 0.00023 | A03~B35~C04~DR03~DQ02~DP104 | 0.00113 |  |  | A24~B49~DR13 | 0.0004 |
| A66~B49~C17 | 0.00026 | A02~B18~C07~DR16 | 0.00048 | A02~B51~C03~DR12~DQ03 | 0.00023 | A32~B58~C03~DR01~DQ05~DP04 | 0.00113 |  |  | A11~B18~DR11 | 0.0004 |
| A03~B45~C16 | 0.00026 | A29~B55~C01~DR03 | 0.00048 | A24~B40~C15~DR12~DQ03 | 0.00023 | A02~B51~C16~DR14~DQ05~DP10 | 0.00113 |  |  | A11~B18~DR01 | 0.0004 |
| A68~B57~C07 | 0.00026 | A24~B55~C03~DR13 | 0.00048 | A02~B40~C15~DR12~DQ03 | 0.00023 | A03~B51~C12~DR16~DQ05~DP04 | 0.00113 |  |  | A69~B35~DR04 | 0.0004 |
| A01~B58~C06 | 0.00026 | A01~B41~C07~DR03 | 0.00047 | A24~B51~C03~DR12~DQ03 | 0.00023 | A24~B35~C04~DR04~DQ06~DP03 | 0.00113 |  |  | A11~B18~DR04 | 0.0004 |
| A30~B38~C06 | 0.00026 | A30~B49~C07~DR11 | 0.00047 | A02~B51~C15~DR12~DQ03 | 0.00023 | A11~B44~C16~DR01~DQ05~DP04 | 0.00113 |  |  | A11~B44~DR11 | 0.0004 |
| A29~B27~C02 | 0.00026 | A24~B52~C12~DR01 | 0.00047 | A24~B40~C03~DR12~DQ03 | 0.00023 | A24~B51~C16~DR16~DQ05~DP04 | 0.00113 |  |  | A29~B07~DR13 | 0.0004 |
| A03~B73~C01 | 0.00026 | A26~B55~C01~DR13 | 0.00047 | A02~B35~C04~DR01~DQ05 | 0.00014 | A32~B15~C03~DR16~DQ05~DP04 | 0.00113 |  |  | A01~B35~DR10 | 0.0004 |
| A24~B73~C15 | 0.00026 | A68~B40~C03~DR13 | 0.00047 | A24~B14~C14~DR04~DQ06 | 0.00011 | A26~B35~C04~DR11~DQ03~DP23 | 0.00113 |  |  | A11~B38~DR04 | 0.0004 |
| A25~B40~C02 | 0.00025 | A26~B38~C12~DR16 | 0.00045 | A66~B55~C17~DR13~DQ06 | 0.00011 | A68~B40~C03~DR13~DQ06~DP02 | 0.00113 |  |  | A33~B58~DR01 | 0.0004 |
| A33~B51~C07 | 0.00025 | A11~B35~C04~DR07 | 0.00045 | A24~B55~C14~DR04~DQ06 | 0.00011 | A02~B51~C04~DR14~DQ05~DP04 | 0.00113 |  |  | A32~B18~DR16 | 0.0004 |
| A25~B41~C07 | 0.00025 | A26~B39~C12~DR16 | 0.00045 | A66~B14~C17~DR13~DQ06 | 0.00011 | A02~B50~C06~DR04~DQ03~DP06 | 0.00113 |  |  | A24~B38~DR03 | 0.0004 |
| A01~B51~C07 | 0.00025 | A32~B51~C14~DR03 | 0.00045 | A24~B14~C17~DR04~DQ06 | 0.00011 | A02~B57~C06~DR04~DQ03~DP04 | 0.00113 |  |  | A24~B07~DR10 | 0.0004 |
| A33~B51~C02 | 0.00025 | A29~B44~C07~DR15 | 0.00045 | A66~B55~C14~DR13~DQ06 | 0.00011 | A11~B40~C07~DR12~DQ03~DP189 | 0.00113 |  |  | A24~B55~DR04 | 0.0004 |
| A68~B58~C07 | 0.00025 | A30~B41~C17~DR04 | 0.00044 | A24~B55~C17~DR04~DQ06 | 0.00011 | A24~B15~C03~DR12~DQ03~DP02 | 0.00113 |  |  | A02~B07~DR16 | 0.0004 |
| A03~B51~C12 | 0.00024 | A33~B14~C08~DR04 | 0.00044 | A66~B14~C14~DR13~DQ06 | 0.00011 | A24~B35~C05~DR13~DQ06~DP04 | 0.00113 |  |  | A23~B07~DR07 | 0.0004 |
| A68~B41~C07 | 0.00024 | A32~B18~C12~DR01 | 0.00044 | A24~B14~C14~DR13~DQ06 | 0.00011 | A03~B07~C07~DR15~DQ03~DP01 | 0.00096 |  |  | A23~B07~DR15 | 0.0004 |
| A26~B35~C17 | 0.00024 | A02~B55~C01~DR14 | 0.00044 | A66~B55~C17~DR04~DQ06 | 0.00011 | A32~B51~C14~DR03~DQ02~DP04 | 0.00087 |  |  | A02~B40~DR09 | 0.0004 |
| A30~B41~C01 | 0.00024 | A02~B51~C06~DR13 | 0.00043 | A24~B55~C14~DR13~DQ06 | 0.00011 | A02~B41~C17~DR04~DQ03~DP04 | 0.00057 |  |  | A33~B55~DR13 | 0.0004 |
| A29~B38~C12 | 0.00024 | A02~B37~C06~DR10 | 0.00043 | A66~B14~C17~DR04~DQ06 | 0.00011 | A01~B15~C03~DR13~DQ06~DP02 | 0.00009 |  |  | A03~B40~DR04 | 0.0004 |
| A29~B40~C02 | 0.00023 | A30~B51~C14~DR16 | 0.00043 | A24~B14~C17~DR13~DQ06 | 0.00011 | A02~B35~C12~DR14~DQ05~DP02 | 0.00003 |  |  | A02~B58~DR04 | 0.0004 |
| A01~B44~C02 | 0.00023 | A03~B51~C14~DR07 | 0.00043 | A66~B55~C14~DR04~DQ06 | 0.00011 |  |  |  |  | A68~B50~DR03 | 0.0004 |
| A32~B55~C04 | 0.00022 | A02~B08~C07~DR07 | 0.00042 | A24~B55~C17~DR13~DQ06 | 0.00011 |  |  |  |  | A03~B18~DR10 | 0.0004 |
| A11~B40~C08 | 0.00022 | A02~B51~C14~DR16 | 0.00042 | A66~B14~C14~DR04~DQ06 | 0.00011 |  |  |  |  | A33~B14~DR03 | 0.0004 |
| A11~B39~C12 | 0.00022 | A02~B14~C08~DR16 | 0.00042 | A30~B18~C07~DR08~DQ03 | 0.00011 |  |  |  |  | A29~B35~DR01 | 0.0004 |
| A31~B27~C02 | 0.00021 | A02~B44~C01~DR13 | 0.00042 | A32~B51~C14~DR12~DQ03 | 0.00011 |  |  |  |  | A01~B44~DR15 | 0.0004 |
| A01~B53~C06 | 0.00021 | A02~B44~C05~DR07 | 0.00041 | A30~B51~C07~DR08~DQ03 | 0.00011 |  |  |  |  | A01~B44~DR04 | 0.0004 |
| A03~B40~C12 | 0.00021 | A24~B40~C15~DR13 | 0.00041 | A32~B18~C14~DR12~DQ03 | 0.00011 |  |  |  |  | A24~B50~DR07 | 0.0004 |
| A29~B08~C07 | 0.00019 | A24~B15~C01~DR14 | 0.00041 | A30~B18~C14~DR08~DQ03 | 0.00011 |  |  |  |  | A03~B51~DR01 | 0.0004 |
| A33~B41~C17 | 0.00019 | A23~B49~C06~DR15 | 0.00041 | A32~B51~C07~DR12~DQ03 | 0.00011 |  |  |  |  | A01~B49~DR01 | 0.0004 |
| A66~B48~C03 | 0.00019 | A01~B35~C12~DR11 | 0.0004 | A30~B51~C14~DR08~DQ03 | 0.00011 |  |  |  |  | A11~B51~DR07 | 0.0004 |
| A11~B14~C08 | 0.00017 | A03~B18~C12~DR10 | 0.0004 | A32~B18~C07~DR12~DQ03 | 0.00011 |  |  |  |  | A23~B50~DR15 | 0.0004 |
| A30~B44~C03 | 0.00017 | A01~B49~C07~DR16 | 0.0004 | A30~B18~C07~DR12~DQ03 | 0.00011 |  |  |  |  | A11~B54~DR13 | 0.0004 |
| A01~B44~C15 | 0.00016 | A23~B52~C12~DR15 | 0.0004 | A32~B51~C14~DR08~DQ03 | 0.00011 |  |  |  |  | A24~B07~DR03 | 0.0004 |
| A33~B44~C07 | 0.00016 | A02~B44~C16~DR15 | 0.0004 | A30~B51~C07~DR12~DQ03 | 0.00011 |  |  |  |  | A29~B58~DR14 | 0.0004 |
| A26~B15~C06 | 0.00016 | A33~B58~C03~DR11 | 0.00039 | A32~B18~C14~DR08~DQ03 | 0.00011 |  |  |  |  | A24~B48~DR12 | 0.0004 |
| A29~B44~C04 | 0.00015 | A03~B07~C07~DR03 | 0.00039 | A30~B18~C14~DR12~DQ03 | 0.00011 |  |  |  |  | A30~B13~DR01 | 0.0004 |
| A68~B13~C07 | 0.00015 | A26~B40~C02~DR04 | 0.00039 | A32~B51~C07~DR08~DQ03 | 0.00011 |  |  |  |  | A30~B13~DR16 | 0.0004 |
| A24~B51~C02 | 0.00014 | A24~B48~C08~DR12 | 0.00039 | A30~B51~C14~DR12~DQ03 | 0.00011 |  |  |  |  | A24~B58~DR13 | 0.0004 |
| A68~B46~C01 | 0.00014 | A68~B57~C06~DR04 | 0.00039 | A32~B18~C07~DR08~DQ03 | 0.00011 |  |  |  |  | A11~B35~DR03 | 0.0004 |
| A03~B49~C12 | 0.00012 | A02~B40~C03~DR04 | 0.00038 | A23~B35~C04~DR01~DQ02 | 0.00006 |  |  |  |  | A24~B39~DR11 | 0.0004 |
| A11~B35~C01 | 0.00011 | A03~B07~C07~DR04 | 0.00038 | A29~B50~C06~DR03~DQ05 | 0.00006 |  |  |  |  | A02~B58~DR08 | 0.0004 |
| A33~B44~C05 | 0.00011 | A30~B49~C07~DR13 | 0.00038 | A23~B50~C04~DR01~DQ02 | 0.00006 |  |  |  |  | A03~B37~DR13 | 0.0004 |
| A23~B40~C03 | 0.00009 | A02~B51~C15~DR13 | 0.00038 | A29~B35~C06~DR03~DQ05 | 0.00006 |  |  |  |  | A68~B15~DR10 | 0.0004 |
| A69~B45~C16 | 0.00009 | A23~B49~C07~DR07 | 0.00038 | A23~B35~C06~DR01~DQ02 | 0.00006 |  |  |  |  | A11~B50~DR11 | 0.0004 |
| A68~B38~C12 | 0.00009 | A02~B51~C07~DR15 | 0.00038 | A29~B50~C04~DR03~DQ05 | 0.00006 |  |  |  |  | A30~B51~DR16 | 0.0004 |
| A01~B44~C16 | 0.00008 | A24~B44~C04~DR04 | 0.00038 | A23~B50~C06~DR01~DQ02 | 0.00006 |  |  |  |  | A26~B27~DR07 | 0.0004 |
| A24~B44~C01 | 0.00007 | A02~B18~C12~DR14 | 0.00037 | A29~B35~C04~DR03~DQ05 | 0.00006 |  |  |  |  | A23~B40~DR11 | 0.0004 |
| A26~B18~C12 | 0.00006 | A11~B07~C07~DR08 | 0.00037 | A23~B35~C04~DR03~DQ02 | 0.00006 |  |  |  |  | A68~B40~DR09 | 0.0004 |
| A32~B15~C03 | 0.00006 | A24~B51~C14~DR01 | 0.00037 | A29~B50~C06~DR01~DQ05 | 0.00006 |  |  |  |  | A03~B35~DR09 | 0.0004 |
| A33~B52~C12 | 0.00005 | A31~B15~C01~DR14 | 0.00037 | A23~B50~C04~DR03~DQ02 | 0.00006 |  |  |  |  | A31~B51~DR04 | 0.0004 |
| A25~B55~C03 | 0.00004 | A24~B40~C15~DR04 | 0.00037 | A29~B35~C06~DR01~DQ05 | 0.00006 |  |  |  |  | A26~B39~DR16 | 0.0004 |
|  |  | A01~B57~C06~DR01 | 0.00036 | A23~B35~C06~DR03~DQ02 | 0.00006 |  |  |  |  | A23~B49~DR07 | 0.0004 |
|  |  | A02~B51~C15~DR12 | 0.00036 | A29~B50~C04~DR01~DQ05 | 0.00006 |  |  |  |  | A30~B41~DR12 | 0.0004 |
|  |  | A11~B35~C04~DR03 | 0.00036 | A23~B50~C06~DR03~DQ02 | 0.00006 |  |  |  |  | A03~B52~DR14 | 0.0004 |
|  |  | A24~B51~C01~DR11 | 0.00036 | A29~B35~C04~DR01~DQ05 | 0.00006 |  |  |  |  | A02~B08~DR13 | 0.0004 |
|  |  | A11~B27~C02~DR03 | 0.00036 | A23~B35~C04~DR01~DQ05 | 0.00006 |  |  |  |  | A26~B35~DR08 | 0.0004 |
|  |  | A03~B44~C02~DR16 | 0.00035 | A29~B50~C06~DR03~DQ02 | 0.00006 |  |  |  |  | A26~B51~DR08 | 0.0004 |
|  |  | A01~B55~C03~DR13 | 0.00035 | A23~B50~C04~DR01~DQ05 | 0.00006 |  |  |  |  | A24~B27~DR12 | 0.0004 |
|  |  | A24~B51~C01~DR04 | 0.00035 | A29~B35~C06~DR03~DQ02 | 0.00006 |  |  |  |  | A24~B48~DR04 | 0.0004 |
|  |  | A03~B55~C01~DR08 | 0.00035 | A23~B35~C06~DR01~DQ05 | 0.00006 |  |  |  |  | A26~B51~DR14 | 0.0004 |
|  |  | A26~B15~C07~DR13 | 0.00035 | A29~B50~C04~DR03~DQ02 | 0.00006 |  |  |  |  | A26~B51~DR16 | 0.0004 |
|  |  | A24~B50~C06~DR07 | 0.00035 | A23~B50~C06~DR01~DQ05 | 0.00006 |  |  |  |  | A33~B58~DR11 | 0.0004 |
|  |  | A02~B49~C07~DR11 | 0.00035 | A29~B35~C04~DR03~DQ02 | 0.00006 |  |  |  |  | A03~B49~DR01 | 0.0004 |
|  |  | A26~B55~C01~DR14 | 0.00034 | A23~B35~C04~DR03~DQ05 | 0.00006 |  |  |  |  | A33~B13~DR07 | 0.0004 |
|  |  | A31~B35~C04~DR10 | 0.00034 | A29~B50~C06~DR01~DQ02 | 0.00006 |  |  |  |  | A31~B44~DR16 | 0.0004 |
|  |  | A32~B51~C12~DR01 | 0.00034 | A23~B50~C04~DR03~DQ05 | 0.00006 |  |  |  |  | A03~B40~DR14 | 0.0004 |
|  |  | A11~B27~C02~DR07 | 0.00034 | A29~B35~C06~DR01~DQ02 | 0.00006 |  |  |  |  | A24~B52~DR10 | 0.0004 |
|  |  | A11~B44~C16~DR04 | 0.00034 | A23~B35~C06~DR03~DQ05 | 0.00006 |  |  |  |  | A03~B18~DR16 | 0.0004 |
|  |  | A01~B07~C07~DR15 | 0.00034 | A29~B50~C04~DR01~DQ02 | 0.00006 |  |  |  |  | A02~B40~DR08 | 0.0004 |
|  |  | A03~B38~C12~DR14 | 0.00034 | A23~B50~C06~DR03~DQ05 | 0.00006 |  |  |  |  | A30~B49~DR15 | 0.0004 |
|  |  | A30~B13~C06~DR11 | 0.00034 | A29~B35~C04~DR01~DQ02 | 0.00006 |  |  |  |  | A25~B52~DR15 | 0.0004 |
|  |  | A02~B39~C12~DR10 | 0.00034 | A03~B14~C08~DR08~DQ04 | 0.00006 |  |  |  |  | A33~B48~DR16 | 0.0004 |
|  |  | A01~B18~C07~DR15 | 0.00033 | A23~B51~C16~DR13~DQ06 | 0.00006 |  |  |  |  | A29~B57~DR01 | 0.0004 |
|  |  | A02~B52~C12~DR15 | 0.00033 | A03~B51~C08~DR08~DQ04 | 0.00006 |  |  |  |  | A33~B35~DR12 | 0.0004 |
|  |  | A01~B35~C04~DR01 | 0.00033 | A23~B14~C16~DR13~DQ06 | 0.00006 |  |  |  |  | A69~B13~DR16 | 0.0004 |
|  |  | A24~B07~C14~DR13 | 0.00033 | A03~B14~C16~DR08~DQ04 | 0.00006 |  |  |  |  | A03~B44~DR14 | 0.0003 |
|  |  | A24~B50~C15~DR11 | 0.00033 | A23~B51~C08~DR13~DQ06 | 0.00006 |  |  |  |  | A26~B27~DR11 | 0.0003 |
|  |  | A29~B49~C07~DR11 | 0.00033 | A03~B51~C16~DR08~DQ04 | 0.00006 |  |  |  |  | A68~B27~DR11 | 0.0003 |
|  |  | A29~B35~C04~DR03 | 0.00033 | A23~B14~C08~DR13~DQ06 | 0.00006 |  |  |  |  | A68~B27~DR13 | 0.0003 |
|  |  | A29~B44~C07~DR11 | 0.00033 | A03~B14~C08~DR13~DQ04 | 0.00006 |  |  |  |  | A68~B18~DR14 | 0.0003 |
|  |  | A03~B07~C07~DR10 | 0.00033 | A23~B51~C16~DR08~DQ06 | 0.00006 |  |  |  |  | A01~B58~DR07 | 0.0003 |
|  |  | A01~B49~C07~DR04 | 0.00033 | A03~B51~C08~DR13~DQ04 | 0.00006 |  |  |  |  | A23~B50~DR04 | 0.0003 |
|  |  | A02~B57~C06~DR04 | 0.00033 | A23~B14~C16~DR08~DQ06 | 0.00006 |  |  |  |  | A68~B18~DR10 | 0.0003 |
|  |  | A01~B35~C04~DR03 | 0.00032 | A03~B14~C16~DR13~DQ04 | 0.00006 |  |  |  |  | A68~B18~DR16 | 0.0003 |
|  |  | A01~B55~C01~DR14 | 0.00032 | A23~B51~C08~DR08~DQ06 | 0.00006 |  |  |  |  | A33~B44~DR07 | 0.0003 |
|  |  | A68~B07~C07~DR04 | 0.00032 | A03~B51~C16~DR13~DQ04 | 0.00006 |  |  |  |  | A33~B35~DR16 | 0.0003 |
|  |  | A11~B51~C15~DR15 | 0.00032 | A23~B14~C08~DR08~DQ06 | 0.00006 |  |  |  |  | A26~B35~DR16 | 0.0003 |
|  |  | A11~B39~C12~DR04 | 0.00032 | A03~B14~C08~DR08~DQ06 | 0.00006 |  |  |  |  | A24~B38~DR14 | 0.0003 |
|  |  | A11~B49~C07~DR14 | 0.00032 | A23~B51~C16~DR13~DQ04 | 0.00006 |  |  |  |  | A24~B40~DR13 | 0.0003 |
|  |  | A32~B27~C02~DR11 | 0.00032 | A03~B51~C08~DR08~DQ06 | 0.00006 |  |  |  |  | A30~B18~DR11 | 0.0003 |
|  |  | A32~B35~C04~DR07 | 0.00032 | A23~B14~C16~DR13~DQ04 | 0.00006 |  |  |  |  | A30~B35~DR01 | 0.0003 |
|  |  | A03~B40~C03~DR04 | 0.00032 | A03~B14~C16~DR08~DQ06 | 0.00006 |  |  |  |  | A03~B38~DR04 | 0.0003 |
|  |  | A33~B35~C07~DR11 | 0.00032 | A23~B51~C08~DR13~DQ04 | 0.00006 |  |  |  |  | A02~B38~DR14 | 0.0003 |
|  |  | A24~B51~C15~DR14 | 0.00032 | A03~B51~C16~DR08~DQ06 | 0.00006 |  |  |  |  | A68~B52~DR15 | 0.0003 |
|  |  | A01~B15~C07~DR07 | 0.00031 | A23~B14~C08~DR13~DQ04 | 0.00006 |  |  |  |  | A26~B52~DR15 | 0.0003 |
|  |  | A25~B18~C12~DR14 | 0.00031 | A03~B14~C08~DR13~DQ06 | 0.00006 |  |  |  |  | A02~B58~DR03 | 0.0003 |
|  |  | A25~B51~C15~DR11 | 0.00031 | A23~B51~C16~DR08~DQ04 | 0.00006 |  |  |  |  | A33~B58~DR16 | 0.0003 |
|  |  | A68~B51~C15~DR13 | 0.00031 | A03~B51~C08~DR13~DQ06 | 0.00006 |  |  |  |  | A02~B08~DR09 | 0.0003 |
|  |  | A02~B44~C05~DR16 | 0.00031 | A23~B14~C16~DR08~DQ04 | 0.00006 |  |  |  |  | A68~B44~DR13 | 0.0003 |
|  |  | A26~B44~C05~DR11 | 0.00031 | A03~B14~C16~DR13~DQ06 | 0.00006 |  |  |  |  | A32~B57~DR16 | 0.0003 |
|  |  | A01~B52~C12~DR01 | 0.00031 | A23~B51~C08~DR08~DQ04 | 0.00006 |  |  |  |  | A24~B27~DR07 | 0.0003 |
|  |  | A02~B51~C12~DR14 | 0.00031 | A03~B51~C16~DR13~DQ06 | 0.00006 |  |  |  |  | A23~B55~DR07 | 0.0003 |
|  |  | A11~B52~C07~DR11 | 0.00031 | A23~B14~C08~DR08~DQ04 | 0.00006 |  |  |  |  | A01~B38~DR03 | 0.0003 |
|  |  | A69~B35~C04~DR11 | 0.0003 | A23~B52~C12~DR11~DQ03 | 0.00001 |  |  |  |  | A01~B08~DR14 | 0.0003 |
|  |  | A24~B51~C04~DR11 | 0.0003 |  |  |  |  |  |  | A23~B38~DR11 | 0.0003 |
|  |  | A11~B55~C01~DR03 | 0.0003 |  |  |  |  |  |  | A26~B40~DR11 | 0.0003 |
|  |  | A02~B18~C12~DR08 | 0.0003 |  |  |  |  |  |  | A01~B38~DR04 | 0.0003 |
|  |  | A32~B35~C04~DR03 | 0.0003 |  |  |  |  |  |  | A03~B37~DR01 | 0.0003 |
|  |  | A26~B39~C12~DR04 | 0.0003 |  |  |  |  |  |  | A02~B35~DR09 | 0.0003 |
|  |  | A68~B51~C14~DR11 | 0.0003 |  |  |  |  |  |  | A31~B15~DR11 | 0.0003 |
|  |  | A68~B44~C16~DR04 | 0.0003 |  |  |  |  |  |  | A01~B55~DR13 | 0.0003 |
|  |  | A24~B35~C03~DR15 | 0.0003 |  |  |  |  |  |  | A33~B35~DR11 | 0.0003 |
|  |  | A02~B35~C12~DR11 | 0.00029 |  |  |  |  |  |  | A03~B27~DR12 | 0.0003 |
|  |  | A24~B40~C03~DR13 | 0.00029 |  |  |  |  |  |  | A25~B27~DR04 | 0.0003 |
|  |  | A31~B51~C14~DR15 | 0.00029 |  |  |  |  |  |  | A32~B40~DR04 | 0.0003 |
|  |  | A24~B40~C03~DR11 | 0.00029 |  |  |  |  |  |  | A32~B38~DR13 | 0.0003 |
|  |  | A30~B51~C14~DR04 | 0.00029 |  |  |  |  |  |  | A68~B35~DR01 | 0.0003 |
|  |  | A33~B51~C15~DR11 | 0.00029 |  |  |  |  |  |  | A02~B07~DR09 | 0.0003 |
|  |  | A23~B44~C04~DR15 | 0.00029 |  |  |  |  |  |  | A03~B55~DR04 | 0.0003 |
|  |  | A01~B51~C01~DR11 | 0.00029 |  |  |  |  |  |  | A68~B49~DR04 | 0.0003 |
|  |  | A02~B55~C04~DR11 | 0.00029 |  |  |  |  |  |  | A02~B15~DR01 | 0.0003 |
|  |  | A11~B52~C12~DR11 | 0.00029 |  |  |  |  |  |  | A24~B38~DR11 | 0.0003 |
|  |  | A24~B37~C06~DR04 | 0.00029 |  |  |  |  |  |  | A24~B15~DR01 | 0.0003 |
|  |  | A23~B35~C07~DR11 | 0.00028 |  |  |  |  |  |  | A68~B40~DR11 | 0.0003 |
|  |  | A30~B08~C06~DR03 | 0.00028 |  |  |  |  |  |  | A32~B55~DR03 | 0.0003 |
|  |  | A01~B51~C16~DR11 | 0.00028 |  |  |  |  |  |  | A01~B08~DR07 | 0.0003 |
|  |  | A02~B55~C03~DR04 | 0.00028 |  |  |  |  |  |  | A11~B15~DR16 | 0.0003 |
|  |  | A24~B07~C07~DR14 | 0.00028 |  |  |  |  |  |  | A33~B08~DR03 | 0.0003 |
|  |  | A11~B52~C12~DR14 | 0.00028 |  |  |  |  |  |  | A01~B51~DR01 | 0.0003 |
|  |  | A24~B52~C12~DR04 | 0.00028 |  |  |  |  |  |  | A01~B44~DR13 | 0.0003 |
|  |  | A03~B49~C07~DR01 | 0.00028 |  |  |  |  |  |  | A24~B39~DR16 | 0.0003 |
|  |  | A11~B51~C15~DR14 | 0.00028 |  |  |  |  |  |  | A02~B50~DR16 | 0.0003 |
|  |  | A02~B15~C03~DR01 | 0.00028 |  |  |  |  |  |  | A01~B44~DR12 | 0.0003 |
|  |  | A26~B49~C07~DR13 | 0.00028 |  |  |  |  |  |  | A01~B07~DR03 | 0.0003 |
|  |  | A01~B37~C06~DR01 | 0.00028 |  |  |  |  |  |  | A03~B39~DR16 | 0.0003 |
|  |  | A11~B57~C06~DR07 | 0.00028 |  |  |  |  |  |  | A02~B27~DR09 | 0.0003 |
|  |  | A29~B07~C07~DR11 | 0.00027 |  |  |  |  |  |  | A24~B15~DR08 | 0.0003 |
|  |  | A02~B51~C02~DR04 | 0.00027 |  |  |  |  |  |  | A03~B14~DR15 | 0.0003 |
|  |  | A02~B51~C07~DR11 | 0.00027 |  |  |  |  |  |  | A03~B18~DR13 | 0.0003 |
|  |  | A01~B41~C17~DR15 | 0.00027 |  |  |  |  |  |  | A01~B58~DR11 | 0.0003 |
|  |  | A11~B51~C14~DR11 | 0.00027 |  |  |  |  |  |  | A01~B55~DR04 | 0.0003 |
|  |  | A30~B35~C04~DR11 | 0.00027 |  |  |  |  |  |  | A23~B27~DR11 | 0.0003 |
|  |  | A26~B51~C15~DR15 | 0.00027 |  |  |  |  |  |  | A23~B39~DR11 | 0.0003 |
|  |  | A11~B51~C16~DR14 | 0.00027 |  |  |  |  |  |  | A24~B51~DR03 | 0.0003 |
|  |  | A69~B18~C07~DR11 | 0.00027 |  |  |  |  |  |  | A02~B58~DR14 | 0.0003 |
|  |  | A01~B35~C07~DR11 | 0.00027 |  |  |  |  |  |  | A02~B48~DR14 | 0.0003 |
|  |  | A01~B35~C12~DR07 | 0.00027 |  |  |  |  |  |  | A11~B14~DR01 | 0.0003 |
|  |  | A01~B57~C06~DR16 | 0.00027 |  |  |  |  |  |  | A24~B48~DR11 | 0.0003 |
|  |  | A24~B56~C01~DR15 | 0.00027 |  |  |  |  |  |  | A32~B07~DR01 | 0.0003 |
|  |  | A01~B35~C07~DR13 | 0.00027 |  |  |  |  |  |  | A30~B35~DR16 | 0.0003 |
|  |  | A02~B07~C04~DR07 | 0.00027 |  |  |  |  |  |  | A68~B51~DR04 | 0.0003 |
|  |  | A68~B35~C04~DR13 | 0.00027 |  |  |  |  |  |  | A02~B14~DR03 | 0.0003 |
|  |  | A01~B35~C16~DR14 | 0.00027 |  |  |  |  |  |  | A32~B50~DR07 | 0.0003 |
|  |  | A33~B14~C08~DR15 | 0.00027 |  |  |  |  |  |  | A24~B35~DR03 | 0.0003 |
|  |  | A26~B08~C07~DR11 | 0.00027 |  |  |  |  |  |  | A33~B14~DR07 | 0.0003 |
|  |  | A03~B52~C12~DR04 | 0.00026 |  |  |  |  |  |  | A33~B51~DR01 | 0.0003 |
|  |  | A24~B55~C01~DR01 | 0.00026 |  |  |  |  |  |  | A33~B51~DR04 | 0.0003 |
|  |  | A26~B38~C12~DR13 | 0.00026 |  |  |  |  |  |  | A24~B39~DR04 | 0.0003 |
|  |  | A01~B55~C01~DR04 | 0.00026 |  |  |  |  |  |  | A01~B15~DR07 | 0.0003 |
|  |  | A26~B55~C03~DR15 | 0.00026 |  |  |  |  |  |  | A68~B35~DR03 | 0.0003 |
|  |  | A01~B08~C07~DR04 | 0.00026 |  |  |  |  |  |  | A01~B51~DR08 | 0.0003 |
|  |  | A03~B07~C07~DR13 | 0.00026 |  |  |  |  |  |  | A30~B55~DR14 | 0.0003 |
|  |  | A23~B44~C04~DR11 | 0.00026 |  |  |  |  |  |  | A26~B50~DR11 | 0.0003 |
|  |  | A02~B38~C12~DR04 | 0.00026 |  |  |  |  |  |  | A01~B50~DR13 | 0.0003 |
|  |  | A26~B44~C05~DR15 | 0.00026 |  |  |  |  |  |  | A01~B07~DR16 | 0.0003 |
|  |  | A24~B13~C06~DR11 | 0.00026 |  |  |  |  |  |  | A03~B40~DR16 | 0.0003 |
|  |  | A68~B35~C01~DR13 | 0.00026 |  |  |  |  |  |  | A26~B13~DR07 | 0.0003 |
|  |  | A23~B49~C07~DR16 | 0.00026 |  |  |  |  |  |  | A24~B15~DR14 | 0.0003 |
|  |  | A11~B50~C04~DR01 | 0.00026 |  |  |  |  |  |  | A24~B58~DR03 | 0.0003 |
|  |  | A01~B35~C06~DR07 | 0.00026 |  |  |  |  |  |  | A30~B13~DR12 | 0.0003 |
|  |  | A24~B55~C01~DR15 | 0.00026 |  |  |  |  |  |  | A24~B40~DR08 | 0.0003 |
|  |  | A29~B08~C07~DR03 | 0.00026 |  |  |  |  |  |  | A24~B58~DR08 | 0.0003 |
|  |  | A24~B18~C12~DR04 | 0.00026 |  |  |  |  |  |  | A02~B38~DR16 | 0.0003 |
|  |  | A03~B44~C16~DR11 | 0.00026 |  |  |  |  |  |  | A31~B44~DR04 | 0.0003 |
|  |  | A29~B44~C05~DR15 | 0.00026 |  |  |  |  |  |  | A30~B14~DR04 | 0.0003 |
|  |  | A01~B52~C12~DR04 | 0.00026 |  |  |  |  |  |  | A11~B13~DR14 | 0.0003 |
|  |  | A32~B07~C15~DR13 | 0.00026 |  |  |  |  |  |  | A26~B44~DR15 | 0.0003 |
|  |  | A68~B51~C07~DR15 | 0.00026 |  |  |  |  |  |  | A26~B39~DR11 | 0.0003 |
|  |  | A68~B51~C04~DR15 | 0.00026 |  |  |  |  |  |  | A30~B50~DR07 | 0.0003 |
|  |  | A02~B44~C03~DR11 | 0.00026 |  |  |  |  |  |  | A30~B51~DR04 | 0.0003 |
|  |  | A02~B60~C06~DR11 | 0.00026 |  |  |  |  |  |  | A68~B56~DR13 | 0.0003 |
|  |  | A02~B44~C06~DR09 | 0.00026 |  |  |  |  |  |  | A25~B44~DR07 | 0.0003 |
|  |  | A01~B07~C04~DR13 | 0.00026 |  |  |  |  |  |  | A02~B45~DR04 | 0.0003 |
|  |  | A02~B35~C14~DR04 | 0.00026 |  |  |  |  |  |  | A26~B49~DR15 | 0.0003 |
|  |  | A01~B55~C06~DR15 | 0.00026 |  |  |  |  |  |  | A26~B49~DR14 | 0.0003 |
|  |  | A01~B44~C16~DR15 | 0.00026 |  |  |  |  |  |  | A03~B50~DR15 | 0.0003 |
|  |  | A03~B51~C01~DR07 | 0.00026 |  |  |  |  |  |  | A68~B41~DR13 | 0.0003 |
|  |  | A32~B18~C12~DR15 | 0.00026 |  |  |  |  |  |  | A11~B58~DR03 | 0.0003 |
|  |  | A01~B35~C07~DR14 | 0.00026 |  |  |  |  |  |  | A23~B51~DR13 | 0.0003 |
|  |  | A02~B73~C15~DR04 | 0.00026 |  |  |  |  |  |  | A23~B52~DR15 | 0.0003 |
|  |  | A33~B51~C04~DR04 | 0.00026 |  |  |  |  |  |  | A32~B40~DR03 | 0.0003 |
|  |  | A33~B51~C04~DR15 | 0.00026 |  |  |  |  |  |  | A26~B07~DR07 | 0.0003 |
|  |  | A31~B48~C08~DR12 | 0.00026 |  |  |  |  |  |  | A68~B47~DR13 | 0.0003 |
|  |  | A01~B37~C06~DR11 | 0.00026 |  |  |  |  |  |  | A02~B44~DR08 | 0.0003 |
|  |  | A02~B15~C06~DR16 | 0.00026 |  |  |  |  |  |  | A68~B58~DR08 | 0.0003 |
|  |  | A66~B14~C17~DR13 | 0.00026 |  |  |  |  |  |  | A23~B41~DR13 | 0.0003 |
|  |  | A03~B35~C16~DR04 | 0.00026 |  |  |  |  |  |  | A03~B13~DR11 | 0.0003 |
|  |  | A74~B50~C06~DR07 | 0.00026 |  |  |  |  |  |  | A11~B40~DR08 | 0.0003 |
|  |  | A74~B51~C14~DR07 | 0.00026 |  |  |  |  |  |  | A32~B44~DR07 | 0.0003 |
|  |  | A24~B35~C07~DR04 | 0.00026 |  |  |  |  |  |  | A01~B41~DR15 | 0.0003 |
|  |  | A01~B39~C12~DR04 | 0.00026 |  |  |  |  |  |  | A24~B15~DR16 | 0.0003 |
|  |  | A01~B35~C14~DR11 | 0.00026 |  |  |  |  |  |  | A11~B39~DR16 | 0.0003 |
|  |  | A01~B38~C12~DR03 | 0.00026 |  |  |  |  |  |  | A26~B41~DR13 | 0.0003 |
|  |  | A02~B51~C07~DR03 | 0.00026 |  |  |  |  |  |  | A03~B41~DR11 | 0.0003 |
|  |  | A01~B44~C04~DR03 | 0.00026 |  |  |  |  |  |  | A26~B41~DR03 | 0.0003 |
|  |  | A03~B35~C07~DR04 | 0.00026 |  |  |  |  |  |  | A24~B52~DR04 | 0.0003 |
|  |  | A23~B50~C07~DR11 | 0.00026 |  |  |  |  |  |  | A32~B38~DR15 | 0.0003 |
|  |  | A11~B50~C06~DR11 | 0.00026 |  |  |  |  |  |  | A30~B51~DR01 | 0.0003 |
|  |  | A01~B35~C06~DR03 | 0.00026 |  |  |  |  |  |  | A30~B40~DR09 | 0.0003 |
|  |  | A24~B35~C05~DR11 | 0.00026 |  |  |  |  |  |  | A11~B55~DR14 | 0.0003 |
|  |  | A03~B40~C05~DR16 | 0.00026 |  |  |  |  |  |  | A69~B51~DR16 | 0.0003 |
|  |  | A01~B35~C06~DR04 | 0.00026 |  |  |  |  |  |  | A03~B08~DR15 | 0.0003 |
|  |  | A01~B35~C07~DR04 | 0.00026 |  |  |  |  |  |  | A26~B49~DR01 | 0.0003 |
|  |  | A03~B44~C07~DR15 | 0.00026 |  |  |  |  |  |  | A32~B51~DR08 | 0.0003 |
|  |  | A26~B51~C16~DR10 | 0.00026 |  |  |  |  |  |  | A68~B37~DR13 | 0.0003 |
|  |  | A30~B13~C12~DR15 | 0.00026 |  |  |  |  |  |  | A32~B07~DR13 | 0.0003 |
|  |  | A68~B44~C05~DR12 | 0.00026 |  |  |  |  |  |  | A66~B41~DR10 | 0.0003 |
|  |  | A24~B35~C02~DR01 | 0.00026 |  |  |  |  |  |  | A01~B52~DR04 | 0.0003 |
|  |  | A25~B40~C02~DR01 | 0.00026 |  |  |  |  |  |  | A11~B38~DR10 | 0.0003 |
|  |  | A02~B35~C14~DR11 | 0.00026 |  |  |  |  |  |  | A24~B37~DR16 | 0.0003 |
|  |  | A01~B51~C14~DR03 | 0.00026 |  |  |  |  |  |  | A26~B56~DR08 | 0.0003 |
|  |  | A01~B51~C14~DR08 | 0.00026 |  |  |  |  |  |  | A11~B52~DR08 | 0.0003 |
|  |  | A01~B44~C15~DR11 | 0.00026 |  |  |  |  |  |  | A30~B15~DR16 | 0.0003 |
|  |  | A25~B44~C02~DR16 | 0.00026 |  |  |  |  |  |  | A02~B49~DR10 | 0.0003 |
|  |  | A01~B38~C07~DR14 | 0.00026 |  |  |  |  |  |  | A11~B57~DR13 | 0.0003 |
|  |  | A02~B50~C16~DR07 | 0.00026 |  |  |  |  |  |  | A30~B40~DR13 | 0.0003 |
|  |  | A32~B07~C04~DR15 | 0.00026 |  |  |  |  |  |  | A01~B45~DR03 | 0.0003 |
|  |  | A68~B50~C07~DR07 | 0.00026 |  |  |  |  |  |  | A11~B27~DR10 | 0.0003 |
|  |  | A26~B49~C07~DR15 | 0.00026 |  |  |  |  |  |  | A68~B38~DR15 | 0.0003 |
|  |  | A02~B35~C07~DR01 | 0.00026 |  |  |  |  |  |  | A30~B15~DR08 | 0.0003 |
|  |  | A01~B18~C01~DR12 | 0.00026 |  |  |  |  |  |  | A01~B15~DR14 | 0.0002 |
|  |  | A02~B35~C14~DR13 | 0.00026 |  |  |  |  |  |  | A29~B35~DR07 | 0.0002 |
|  |  | A32~B37~C01~DR10 | 0.00026 |  |  |  |  |  |  | A01~B18~DR04 | 0.0002 |
|  |  | A26~B18~C07~DR14 | 0.00026 |  |  |  |  |  |  | A02~B52~DR01 | 0.0002 |
|  |  | A68~B49~C07~DR15 | 0.00026 |  |  |  |  |  |  | A68~B51~DR16 | 0.0002 |
|  |  | A30~B18~C07~DR08 | 0.00026 |  |  |  |  |  |  | A02~B15~DR15 | 0.0002 |
|  |  | A32~B39~C12~DR13 | 0.00026 |  |  |  |  |  |  | A11~B52~DR13 | 0.0002 |
|  |  | A31~B49~C07~DR01 | 0.00026 |  |  |  |  |  |  | A11~B18~DR13 | 0.0002 |
|  |  | A23~B27~C15~DR04 | 0.00026 |  |  |  |  |  |  | A68~B27~DR04 | 0.0002 |
|  |  | A31~B58~C07~DR15 | 0.00026 |  |  |  |  |  |  | A26~B18~DR11 | 0.0002 |
|  |  | A66~B41~C17~DR10 | 0.00026 |  |  |  |  |  |  | A03~B37~DR04 | 0.0002 |
|  |  | A33~B51~C07~DR01 | 0.00026 |  |  |  |  |  |  | A02~B13~DR15 | 0.0002 |
|  |  | A30~B27~C01~DR08 | 0.00026 |  |  |  |  |  |  | A02~B07~DR07 | 0.0002 |
|  |  | A03~B49~C07~DR04 | 0.00026 |  |  |  |  |  |  | A01~B27~DR13 | 0.0002 |
|  |  | A29~B35~C16~DR01 | 0.00026 |  |  |  |  |  |  | A31~B35~DR12 | 0.0002 |
|  |  | A68~B35~C12~DR13 | 0.00026 |  |  |  |  |  |  | A01~B35~DR12 | 0.0002 |
|  |  | A68~B15~C12~DR13 | 0.00026 |  |  |  |  |  |  | A29~B58~DR16 | 0.0002 |
|  |  | A01~B51~C16~DR16 | 0.00026 |  |  |  |  |  |  | A02~B58~DR16 | 0.0002 |
|  |  | A02~B54~C07~DR04 | 0.00026 |  |  |  |  |  |  | A02~B49~DR04 | 0.0002 |
|  |  | A31~B40~C03~DR14 | 0.00026 |  |  |  |  |  |  | A02~B49~DR03 | 0.0002 |
|  |  | A01~B48~C08~DR04 | 0.00026 |  |  |  |  |  |  | A23~B35~DR03 | 0.0002 |
|  |  | A01~B48~C08~DR03 | 0.00026 |  |  |  |  |  |  | A11~B58~DR13 | 0.0002 |
|  |  | A24~B18~C16~DR12 | 0.00026 |  |  |  |  |  |  | A68~B35~DR15 | 0.0002 |
|  |  | A30~B08~C07~DR13 | 0.00026 |  |  |  |  |  |  | A32~B07~DR11 | 0.0002 |
|  |  | A30~B51~C15~DR13 | 0.00026 |  |  |  |  |  |  | A32~B51~DR10 | 0.0002 |
|  |  | A32~B40~C03~DR11 | 0.00026 |  |  |  |  |  |  | A02~B08~DR11 | 0.0002 |
|  |  | A02~B44~C06~DR04 | 0.00026 |  |  |  |  |  |  | A01~B56~DR01 | 0.0002 |
|  |  | A01~B14~C08~DR16 | 0.00026 |  |  |  |  |  |  | A32~B51~DR16 | 0.0002 |
|  |  | A30~B49~C07~DR16 | 0.00026 |  |  |  |  |  |  | A02~B51~DR08 | 0.0002 |
|  |  | A23~B35~C07~DR16 | 0.00026 |  |  |  |  |  |  | A33~B35~DR15 | 0.0002 |
|  |  | A26~B44~C12~DR04 | 0.00026 |  |  |  |  |  |  | A03~B51~DR08 | 0.0002 |
|  |  | A25~B50~C06~DR13 | 0.00026 |  |  |  |  |  |  | A29~B38~DR13 | 0.0002 |
|  |  | A25~B51~C06~DR12 | 0.00026 |  |  |  |  |  |  | A01~B37~DR03 | 0.0002 |
|  |  | A30~B53~C04~DR11 | 0.00026 |  |  |  |  |  |  | A11~B41~DR13 | 0.0002 |
|  |  | A26~B15~C03~DR13 | 0.00026 |  |  |  |  |  |  | A02~B27~DR13 | 0.0002 |
|  |  | A11~B27~C12~DR14 | 0.00026 |  |  |  |  |  |  | A68~B08~DR03 | 0.0002 |
|  |  | A23~B41~C17~DR10 | 0.00026 |  |  |  |  |  |  | A32~B44~DR04 | 0.0002 |
|  |  | A02~B35~C17~DR13 | 0.00026 |  |  |  |  |  |  | A02~B58~DR01 | 0.0002 |
|  |  | A03~B52~C12~DR03 | 0.00026 |  |  |  |  |  |  | A02~B52~DR04 | 0.0002 |
|  |  | A68~B15~C03~DR04 | 0.00026 |  |  |  |  |  |  | A02~B27~DR10 | 0.0002 |
|  |  | A33~B15~C08~DR04 | 0.00026 |  |  |  |  |  |  | A68~B51~DR15 | 0.0002 |
|  |  | A02~B35~C12~DR13 | 0.00026 |  |  |  |  |  |  | A30~B13~DR15 | 0.0002 |
|  |  | A02~B38~C12~DR03 | 0.00026 |  |  |  |  |  |  | A24~B35~DR10 | 0.0002 |
|  |  | A23~B51~C16~DR01 | 0.00026 |  |  |  |  |  |  | A24~B52~DR14 | 0.0002 |
|  |  | A01~B15~C16~DR04 | 0.00026 |  |  |  |  |  |  | A02~B50~DR14 | 0.0002 |
|  |  | A01~B15~C03~DR15 | 0.00026 |  |  |  |  |  |  | A32~B52~DR11 | 0.0002 |
|  |  | A02~B44~C15~DR04 | 0.00026 |  |  |  |  |  |  | A25~B51~DR01 | 0.0002 |
|  |  | A02~B40~C15~DR08 | 0.00026 |  |  |  |  |  |  | A25~B35~DR15 | 0.0002 |
|  |  | A02~B35~C15~DR04 | 0.00026 |  |  |  |  |  |  | A31~B51~DR13 | 0.0002 |
|  |  | A02~B40~C15~DR16 | 0.00026 |  |  |  |  |  |  | A33~B44~DR15 | 0.0002 |
|  |  | A02~B45~C16~DR08 | 0.00026 |  |  |  |  |  |  | A03~B37~DR10 | 0.0002 |
|  |  | A02~B57~C18~DR15 | 0.00026 |  |  |  |  |  |  | A32~B18~DR15 | 0.0002 |
|  |  | A11~B35~C18~DR12 | 0.00026 |  |  |  |  |  |  | A30~B37~DR10 | 0.0002 |
|  |  | A11~B35~C12~DR01 | 0.00026 |  |  |  |  |  |  | A31~B35~DR04 | 0.0002 |
|  |  | A02~B35~C16~DR14 | 0.00026 |  |  |  |  |  |  | A33~B18~DR11 | 0.0002 |
|  |  | A02~B35~C12~DR03 | 0.00026 |  |  |  |  |  |  | A33~B49~DR04 | 0.0002 |
|  |  | A32~B52~C12~DR03 | 0.00026 |  |  |  |  |  |  | A03~B49~DR15 | 0.0002 |
|  |  | A32~B35~C12~DR03 | 0.00026 |  |  |  |  |  |  | A32~B35~DR10 | 0.0002 |
|  |  | A69~B40~C03~DR14 | 0.00026 |  |  |  |  |  |  | A68~B40~DR01 | 0.0002 |
|  |  | A32~B27~C02~DR14 | 0.00026 |  |  |  |  |  |  | A68~B15~DR04 | 0.0002 |
|  |  | A26~B39~C12~DR13 | 0.00026 |  |  |  |  |  |  | A02~B55~DR11 | 0.0002 |
|  |  | A24~B55~C07~DR14 | 0.00026 |  |  |  |  |  |  | A24~B51~DR09 | 0.0002 |
|  |  | A02~B44~C15~DR16 | 0.00026 |  |  |  |  |  |  | A32~B49~DR04 | 0.0002 |
|  |  | A66~B07~C07~DR03 | 0.00026 |  |  |  |  |  |  | A32~B39~DR07 | 0.0002 |
|  |  | A11~B13~C08~DR07 | 0.00026 |  |  |  |  |  |  | A32~B51~DR14 | 0.0002 |
|  |  | A68~B38~C12~DR08 | 0.00026 |  |  |  |  |  |  | A01~B14~DR11 | 0.0002 |
|  |  | A33~B44~C14~DR13 | 0.00026 |  |  |  |  |  |  | A02~B41~DR01 | 0.0002 |
|  |  | A31~B40~C07~DR04 | 0.00026 |  |  |  |  |  |  | A25~B18~DR11 | 0.0002 |
|  |  | A24~B58~C07~DR14 | 0.00026 |  |  |  |  |  |  | A24~B18~DR16 | 0.0002 |
|  |  | A23~B38~C12~DR13 | 0.00026 |  |  |  |  |  |  | A69~B15~DR01 | 0.0002 |
|  |  | A68~B39~C12~DR13 | 0.00026 |  |  |  |  |  |  | A26~B58~DR03 | 0.0002 |
|  |  | A68~B39~C12~DR07 | 0.00026 |  |  |  |  |  |  | A26~B47~DR15 | 0.0002 |
|  |  | A29~B44~C16~DR09 | 0.00026 |  |  |  |  |  |  | A24~B15~DR15 | 0.0002 |
|  |  | A26~B51~C15~DR09 | 0.00026 |  |  |  |  |  |  | A31~B14~DR13 | 0.0002 |
|  |  | A03~B27~C02~DR04 | 0.00026 |  |  |  |  |  |  | A23~B18~DR01 | 0.0002 |
|  |  | A26~B35~C12~DR04 | 0.00026 |  |  |  |  |  |  | A03~B52~DR04 | 0.0002 |
|  |  | A66~B40~C17~DR13 | 0.00026 |  |  |  |  |  |  | A26~B58~DR13 | 0.0002 |
|  |  | A68~B40~C08~DR09 | 0.00026 |  |  |  |  |  |  | A01~B08~DR08 | 0.0002 |
|  |  | A26~B51~C06~DR11 | 0.00026 |  |  |  |  |  |  | A03~B57~DR07 | 0.0002 |
|  |  | A03~B15~C03~DR13 | 0.00026 |  |  |  |  |  |  | A02~B55~DR10 | 0.0002 |
|  |  | A23~B15~C15~DR13 | 0.00026 |  |  |  |  |  |  | A03~B52~DR11 | 0.0002 |
|  |  | A02~B57~C07~DR13 | 0.00026 |  |  |  |  |  |  | A24~B08~DR07 | 0.0002 |
|  |  | A03~B50~C04~DR11 | 0.00026 |  |  |  |  |  |  | A68~B53~DR11 | 0.0002 |
|  |  | A02~B48~C03~DR13 | 0.00026 |  |  |  |  |  |  | A68~B18~DR13 | 0.0002 |
|  |  | A66~B48~C03~DR04 | 0.00026 |  |  |  |  |  |  | A24~B53~DR13 | 0.0002 |
|  |  | A03~B48~C03~DR04 | 0.00026 |  |  |  |  |  |  | A03~B39~DR04 | 0.0002 |
|  |  | A30~B53~C04~DR03 | 0.00026 |  |  |  |  |  |  | A23~B50~DR07 | 0.0002 |
|  |  | A33~B13~C07~DR03 | 0.00026 |  |  |  |  |  |  | A24~B52~DR03 | 0.0002 |
|  |  | A02~B39~C01~DR16 | 0.00026 |  |  |  |  |  |  | A68~B58~DR07 | 0.0002 |
|  |  | A66~B55~C01~DR13 | 0.00026 |  |  |  |  |  |  | A02~B48~DR04 | 0.0002 |
|  |  | A24~B54~C01~DR04 | 0.00026 |  |  |  |  |  |  | A29~B44~DR04 | 0.0002 |
|  |  | A02~B35~C01~DR04 | 0.00026 |  |  |  |  |  |  | A23~B14~DR01 | 0.0002 |
|  |  | A23~B35~C06~DR07 | 0.00026 |  |  |  |  |  |  | A02~B48~DR12 | 0.0002 |
|  |  | A01~B35~C06~DR01 | 0.00026 |  |  |  |  |  |  | A26~B13~DR12 | 0.0002 |
|  |  | A24~B07~C04~DR13 | 0.00026 |  |  |  |  |  |  | A33~B14~DR10 | 0.0002 |
|  |  | A03~B57~C06~DR10 | 0.00026 |  |  |  |  |  |  | A23~B14~DR10 | 0.0002 |
|  |  | A01~B07~C14~DR09 | 0.00026 |  |  |  |  |  |  | A02~B39~DR13 | 0.0002 |
|  |  | A03~B51~C07~DR16 | 0.00026 |  |  |  |  |  |  | A30~B41~DR04 | 0.0002 |
|  |  | A02~B55~C01~DR01 | 0.00026 |  |  |  |  |  |  | A30~B41~DR11 | 0.0002 |
|  |  | A01~B37~C06~DR13 | 0.00026 |  |  |  |  |  |  | A01~B41~DR04 | 0.0002 |
|  |  | A24~B55~C03~DR16 | 0.00026 |  |  |  |  |  |  | A11~B27~DR01 | 0.0002 |
|  |  | A24~B40~C03~DR07 | 0.00026 |  |  |  |  |  |  | A11~B44~DR15 | 0.0002 |
|  |  | A11~B56~C04~DR04 | 0.00026 |  |  |  |  |  |  | A23~B49~DR16 | 0.0002 |
|  |  | A32~B56~C01~DR12 | 0.00026 |  |  |  |  |  |  | A26~B48~DR04 | 0.0002 |
|  |  | A01~B27~C02~DR09 | 0.00026 |  |  |  |  |  |  | A32~B55~DR13 | 0.0002 |
|  |  | A26~B18~C12~DR14 | 0.00026 |  |  |  |  |  |  | A32~B53~DR13 | 0.0002 |
|  |  | A69~B35~C12~DR04 | 0.00026 |  |  |  |  |  |  | A31~B35~DR07 | 0.0002 |
|  |  | A03~B51~C04~DR01 | 0.00026 |  |  |  |  |  |  | A68~B15~DR15 | 0.0002 |
|  |  | A03~B51~C15~DR01 | 0.00026 |  |  |  |  |  |  | A30~B49~DR10 | 0.0002 |
|  |  | A33~B14~C05~DR13 | 0.00026 |  |  |  |  |  |  | A01~B35~DR08 | 0.0002 |
|  |  | A26~B55~C03~DR04 | 0.00026 |  |  |  |  |  |  | A24~B55~DR01 | 0.0002 |
|  |  | A26~B35~C03~DR08 | 0.00026 |  |  |  |  |  |  | A01~B14~DR13 | 0.0002 |
|  |  | A26~B51~C14~DR03 | 0.00026 |  |  |  |  |  |  | A24~B41~DR13 | 0.0002 |
|  |  | A26~B56~C01~DR08 | 0.00026 |  |  |  |  |  |  | A01~B40~DR09 | 0.0002 |
|  |  | A26~B56~C01~DR03 | 0.00026 |  |  |  |  |  |  | A68~B57~DR04 | 0.0002 |
|  |  | A24~B55~C14~DR15 | 0.00026 |  |  |  |  |  |  | A30~B07~DR01 | 0.0002 |
|  |  | A01~B58~C07~DR01 | 0.00026 |  |  |  |  |  |  | A30~B07~DR11 | 0.0002 |
|  |  | A24~B14~C08~DR01 | 0.00026 |  |  |  |  |  |  | A11~B40~DR12 | 0.0002 |
|  |  | A32~B51~C16~DR07 | 0.00026 |  |  |  |  |  |  | A24~B40~DR12 | 0.0002 |
|  |  | A03~B40~C04~DR14 | 0.00026 |  |  |  |  |  |  | A31~B51~DR12 | 0.0002 |
|  |  | A11~B40~C07~DR11 | 0.00026 |  |  |  |  |  |  | A31~B35~DR10 | 0.0002 |
|  |  | A23~B07~C14~DR15 | 0.00026 |  |  |  |  |  |  | A01~B18~DR15 | 0.0002 |
|  |  | A23~B57~C06~DR03 | 0.00026 |  |  |  |  |  |  | A02~B45~DR08 | 0.0002 |
|  |  | A01~B40~C03~DR10 | 0.00026 |  |  |  |  |  |  | A02~B45~DR13 | 0.0002 |
|  |  | A11~B35~C01~DR15 | 0.00026 |  |  |  |  |  |  | A31~B57~DR01 | 0.0002 |
|  |  | A02~B13~C01~DR15 | 0.00026 |  |  |  |  |  |  | A23~B51~DR04 | 0.0002 |
|  |  | A01~B35~C12~DR01 | 0.00026 |  |  |  |  |  |  | A01~B08~DR16 | 0.0002 |
|  |  | A03~B08~C07~DR16 | 0.00026 |  |  |  |  |  |  | A01~B55~DR16 | 0.0002 |
|  |  | A33~B35~C07~DR04 | 0.00026 |  |  |  |  |  |  | A01~B55~DR03 | 0.0002 |
|  |  | A29~B08~C04~DR04 | 0.00026 |  |  |  |  |  |  | A25~B35~DR14 | 0.0002 |
|  |  | A25~B35~C04~DR14 | 0.00026 |  |  |  |  |  |  | A23~B50~DR08 | 0.0002 |
|  |  | A11~B08~C02~DR01 | 0.00026 |  |  |  |  |  |  | A33~B51~DR15 | 0.0002 |
|  |  | A24~B52~C12~DR09 | 0.00026 |  |  |  |  |  |  | A30~B58~DR11 | 0.0002 |
|  |  | A02~B48~C08~DR01 | 0.00026 |  |  |  |  |  |  | A25~B13~DR07 | 0.0002 |
|  |  | A33~B40~C02~DR16 | 0.00026 |  |  |  |  |  |  | A68~B39~DR03 | 0.0002 |
|  |  | A01~B53~C04~DR14 | 0.00026 |  |  |  |  |  |  | A68~B51~DR14 | 0.0002 |
|  |  | A26~B35~C12~DR03 | 0.00026 |  |  |  |  |  |  | A68~B37~DR11 | 0.0002 |
|  |  | A11~B59~C07~DR11 | 0.00026 |  |  |  |  |  |  | A26~B18~DR04 | 0.0002 |
|  |  | A24~B44~C04~DR15 | 0.00026 |  |  |  |  |  |  | A01~B45~DR04 | 0.0002 |
|  |  | A33~B14~C06~DR11 | 0.00026 |  |  |  |  |  |  | A30~B18~DR12 | 0.0002 |
|  |  | A26~B35~C14~DR04 | 0.00026 |  |  |  |  |  |  | A03~B38~DR08 | 0.0002 |
|  |  | A02~B40~C07~DR11 | 0.00026 |  |  |  |  |  |  | A29~B35~DR03 | 0.0002 |
|  |  | A29~B18~C12~DR11 | 0.00026 |  |  |  |  |  |  | A02~B15~DR09 | 0.0002 |
|  |  | A23~B44~C05~DR11 | 0.00026 |  |  |  |  |  |  | A31~B48~DR14 | 0.0002 |
|  |  | A02~B53~C04~DR13 | 0.00026 |  |  |  |  |  |  | A11~B53~DR14 | 0.0002 |
|  |  | A24~B44~C04~DR16 | 0.00026 |  |  |  |  |  |  | A26~B35~DR10 | 0.0002 |
|  |  | A11~B35~C01~DR14 | 0.00026 |  |  |  |  |  |  | A24~B39~DR08 | 0.0002 |
|  |  | A26~B40~C07~DR11 | 0.00026 |  |  |  |  |  |  | A01~B44~DR03 | 0.0002 |
|  |  | A26~B08~C07~DR07 | 0.00026 |  |  |  |  |  |  | A32~B38~DR14 | 0.0002 |
|  |  | A31~B15~C03~DR15 | 0.00026 |  |  |  |  |  |  | A26~B52~DR03 | 0.0002 |
|  |  | A30~B14~C05~DR01 | 0.00026 |  |  |  |  |  |  | A24~B40~DR16 | 0.0002 |
|  |  | A02~B35~C05~DR04 | 0.00026 |  |  |  |  |  |  | A23~B52~DR04 | 0.0002 |
|  |  | A31~B51~C16~DR11 | 0.00026 |  |  |  |  |  |  | A23~B44~DR15 | 0.0002 |
|  |  | A31~B51~C16~DR15 | 0.00026 |  |  |  |  |  |  | A32~B41~DR03 | 0.0002 |
|  |  | A01~B44~C07~DR03 | 0.00026 |  |  |  |  |  |  | A02~B40~DR03 | 0.0002 |
|  |  | A24~B45~C06~DR11 | 0.00026 |  |  |  |  |  |  | A32~B41~DR13 | 0.0002 |
|  |  | A03~B18~C15~DR11 | 0.00026 |  |  |  |  |  |  | A01~B15~DR03 | 0.0002 |
|  |  | A29~B51~C15~DR07 | 0.00026 |  |  |  |  |  |  | A11~B15~DR07 | 0.0002 |
|  |  | A03~B41~C17~DR10 | 0.00026 |  |  |  |  |  |  | A02~B54~DR14 | 0.0002 |
|  |  | A02~B45~C16~DR13 | 0.00026 |  |  |  |  |  |  | A30~B49~DR04 | 0.0002 |
|  |  | A11~B35~C04~DR12 | 0.00026 |  |  |  |  |  |  | A24~B38~DR08 | 0.0002 |
|  |  | A32~B55~C04~DR04 | 0.00026 |  |  |  |  |  |  | A34~B44~DR07 | 0.0002 |
|  |  | A11~B55~C01~DR15 | 0.00026 |  |  |  |  |  |  | A11~B40~DR14 | 0.0002 |
|  |  | A01~B53~C06~DR15 | 0.00026 |  |  |  |  |  |  | A03~B27~DR03 | 0.0002 |
|  |  | A33~B55~C17~DR11 | 0.00026 |  |  |  |  |  |  | A33~B18~DR07 | 0.0002 |
|  |  | A22~B55~C01~DR14 | 0.00026 |  |  |  |  |  |  | A01~B55~DR14 | 0.0002 |
|  |  | A24~B55~C02~DR11 | 0.00026 |  |  |  |  |  |  | A33~B73~DR04 | 0.0002 |
|  |  | A24~B41~C02~DR11 | 0.00026 |  |  |  |  |  |  | A02~B73~DR04 | 0.0002 |
|  |  | A02~B55~C01~DR04 | 0.00026 |  |  |  |  |  |  | A31~B48~DR12 | 0.0002 |
|  |  | A29~B55~C01~DR04 | 0.00026 |  |  |  |  |  |  | A74~B50~DR07 | 0.0002 |
|  |  | A29~B41~C01~DR04 | 0.00026 |  |  |  |  |  |  | A24~B48~DR08 | 0.0002 |
|  |  | A01~B41~C17~DR10 | 0.00026 |  |  |  |  |  |  | A68~B44~DR01 | 0.0002 |
|  |  | A11~B44~C08~DR10 | 0.00026 |  |  |  |  |  |  | A33~B55~DR11 | 0.0002 |
|  |  | A30~B15~C12~DR03 | 0.00026 |  |  |  |  |  |  | A03~B55~DR01 | 0.0002 |
|  |  | A69~B35~C12~DR03 | 0.00026 |  |  |  |  |  |  | A25~B35~DR08 | 0.0002 |
|  |  | A29~B51~C14~DR11 | 0.00026 |  |  |  |  |  |  | A24~B47~DR13 | 0.0002 |
|  |  | A02~B14~C08~DR15 | 0.00026 |  |  |  |  |  |  | A30~B35~DR14 | 0.0002 |
|  |  | A68~B07~C08~DR11 | 0.00026 |  |  |  |  |  |  | A32~B07~DR14 | 0.0002 |
|  |  | A30~B41~C16~DR03 | 0.00026 |  |  |  |  |  |  | A03~B39~DR13 | 0.0002 |
|  |  | A68~B13~C06~DR07 | 0.00026 |  |  |  |  |  |  | A31~B55~DR15 | 0.0002 |
|  |  | A23~B44~C04~DR01 | 0.00026 |  |  |  |  |  |  | A24~B53~DR12 | 0.0002 |
|  |  | A23~B18~C12~DR11 | 0.00026 |  |  |  |  |  |  | A30~B53~DR07 | 0.0002 |
|  |  | A26~B27~C02~DR01 | 0.00026 |  |  |  |  |  |  | A30~B51~DR15 | 0.0002 |
|  |  | A32~B55~C07~DR13 | 0.00026 |  |  |  |  |  |  | A25~B40~DR11 | 0.0002 |
|  |  | A23~B35~C06~DR03 | 0.00026 |  |  |  |  |  |  | A33~B07~DR01 | 0.0002 |
|  |  | A26~B07~C08~DR15 | 0.00026 |  |  |  |  |  |  | A03~B40~DR03 | 0.0002 |
|  |  | A30~B08~C04~DR03 | 0.00026 |  |  |  |  |  |  | A32~B50~DR04 | 0.0002 |
|  |  | A11~B35~C14~DR10 | 0.00026 |  |  |  |  |  |  | A03~B38~DR14 | 0.0002 |
|  |  | A24~B35~C02~DR03 | 0.00026 |  |  |  |  |  |  | A25~B44~DR16 | 0.0002 |
|  |  | A26~B35~C02~DR04 | 0.00026 |  |  |  |  |  |  | A24~B13~DR08 | 0.0002 |
|  |  | A33~B55~C01~DR11 | 0.00026 |  |  |  |  |  |  | A30~B52~DR07 | 0.0002 |
|  |  | A68~B44~C12~DR11 | 0.00026 |  |  |  |  |  |  | A03~B37~DR12 | 0.0002 |
|  |  | A24~B51~C02~DR15 | 0.00026 |  |  |  |  |  |  | A29~B57~DR13 | 0.0002 |
|  |  | A02~B51~C02~DR08 | 0.00026 |  |  |  |  |  |  | A30~B38~DR15 | 0.0002 |
|  |  | A26~B14~C08~DR03 | 0.00026 |  |  |  |  |  |  | A24~B58~DR14 | 0.0002 |
|  |  | A29~B38~C12~DR11 | 0.00026 |  |  |  |  |  |  | A69~B07~DR11 | 0.0002 |
|  |  | A33~B07~C07~DR03 | 0.00026 |  |  |  |  |  |  | A01~B37~DR01 | 0.0002 |
|  |  | A23~B57~C18~DR01 | 0.00026 |  |  |  |  |  |  | A26~B56~DR01 | 0.0002 |
|  |  | A31~B51~C15~DR13 | 0.00026 |  |  |  |  |  |  | A01~B14~DR04 | 0.0002 |
|  |  | A33~B58~C03~DR01 | 0.00026 |  |  |  |  |  |  | A26~B52~DR14 | 0.0002 |
|  |  | A02~B15~C06~DR03 | 0.00026 |  |  |  |  |  |  | A11~B57~DR12 | 0.0002 |
|  |  | A26~B07~C07~DR07 | 0.00026 |  |  |  |  |  |  | A32~B52~DR03 | 0.0002 |
|  |  | A11~B44~C03~DR11 | 0.00026 |  |  |  |  |  |  | A69~B45~DR11 | 0.0002 |
|  |  | A30~B44~C03~DR15 | 0.00026 |  |  |  |  |  |  | A24~B50~DR01 | 0.0002 |
|  |  | A03~B37~C03~DR09 | 0.00026 |  |  |  |  |  |  | A31~B51~DR03 | 0.0002 |
|  |  | A01~B35~C08~DR03 | 0.00026 |  |  |  |  |  |  | A68~B55~DR09 | 0.0002 |
|  |  | A02~B51~C16~DR03 | 0.00026 |  |  |  |  |  |  | A23~B57~DR03 | 0.0002 |
|  |  | A01~B52~C12~DR16 | 0.00026 |  |  |  |  |  |  | A11~B56~DR15 | 0.0002 |
|  |  | A33~B39~C12~DR04 | 0.00026 |  |  |  |  |  |  | A03~B08~DR01 | 0.0002 |
|  |  | A11~B03~C12~DR03 | 0.00026 |  |  |  |  |  |  | A11~B08~DR01 | 0.0002 |
|  |  | A01~B52~C12~DR03 | 0.00026 |  |  |  |  |  |  | A26~B13~DR04 | 0.0002 |
|  |  | A01~B08~C03~DR07 | 0.00026 |  |  |  |  |  |  | A29~B50~DR11 | 0.0002 |
|  |  | A02~B19~C07~DR03 | 0.00026 |  |  |  |  |  |  | A29~B18~DR07 | 0.0002 |
|  |  | A68~B53~C04~DR15 | 0.00026 |  |  |  |  |  |  | A31~B15~DR09 | 0.0002 |
|  |  | A25~B27~C04~DR11 | 0.00026 |  |  |  |  |  |  | A11~B56~DR01 | 0.0002 |
|  |  | A03~B18~C04~DR11 | 0.00026 |  |  |  |  |  |  | A24~B45~DR11 | 0.0002 |
|  |  | A29~B27~C07~DR11 | 0.00026 |  |  |  |  |  |  | A30~B15~DR14 | 0.0002 |
|  |  | A30~B51~C15~DR11 | 0.00026 |  |  |  |  |  |  | A29~B55~DR03 | 0.0002 |
|  |  | A30~B40~C03~DR04 | 0.00026 |  |  |  |  |  |  | A23~B55~DR03 | 0.0002 |
|  |  | A32~B51~C01~DR16 | 0.00026 |  |  |  |  |  |  | A03~B37~DR09 | 0.0002 |
|  |  | A31~B51~C01~DR11 | 0.00026 |  |  |  |  |  |  | A31~B48~DR03 | 0.0002 |
|  |  | A03~B49~C12~DR11 | 0.00026 |  |  |  |  |  |  | A26~B53~DR12 | 0.0002 |
|  |  | A11~B35~C15~DR16 | 0.00026 |  |  |  |  |  |  | A32~B52~DR13 | 0.0002 |
|  |  | A32~B07~C07~DR12 | 0.00026 |  |  |  |  |  |  | A66~B49~DR15 | 0.0002 |
|  |  | A25~B51~C12~DR13 | 0.00026 |  |  |  |  |  |  | A01~B58~DR03 | 0.0002 |
|  |  | A24~B49~C12~DR11 | 0.00026 |  |  |  |  |  |  | A30~B37~DR04 | 0.0002 |
|  |  | A11~B35~C04~DR16 | 0.00026 |  |  |  |  |  |  | A68~B39~DR08 | 0.0002 |
|  |  | A11~B13~C06~DR13 | 0.00026 |  |  |  |  |  |  | A01~B73~DR04 | 0.0002 |
|  |  | A24~B15~C04~DR13 | 0.00026 |  |  |  |  |  |  | A31~B39~DR14 | 0.0002 |
|  |  | A11~B56~C01~DR01 | 0.00026 |  |  |  |  |  |  | A26~B48~DR07 | 0.0002 |
|  |  | A11~B35~C03~DR04 | 0.00026 |  |  |  |  |  |  | A02~B46~DR09 | 0.0002 |
|  |  | A11~B35~C03~DR14 | 0.00026 |  |  |  |  |  |  | A36~B52~DR15 | 0.0002 |
|  |  | A11~B35~C03~DR13 | 0.00026 |  |  |  |  |  |  | A23~B08~DR13 | 0.0002 |
|  |  | A23~B07~C15~DR07 | 0.00026 |  |  |  |  |  |  | A68~B15~DR12 | 0.0002 |
|  |  | A68~B42~C17~DR03 | 0.00026 |  |  |  |  |  |  | A23~B41~DR03 | 0.0002 |
|  |  | A03~B15~C12~DR10 | 0.00026 |  |  |  |  |  |  | A24~B56~DR13 | 0.0002 |
|  |  | A29~B35~C07~DR15 | 0.00026 |  |  |  |  |  |  | A24~B56~DR11 | 0.0002 |
|  |  | A25~B07~C07~DR03 | 0.00026 |  |  |  |  |  |  | A32~B32~DR11 | 0.0002 |
|  |  | A32~B35~C07~DR14 | 0.00026 |  |  |  |  |  |  | A26~B07~DR12 | 0.0002 |
|  |  | A01~B35~C15~DR13 | 0.00026 |  |  |  |  |  |  | A03~B39~DR12 | 0.0002 |
|  |  | A03~B15~C03~DR08 | 0.00026 |  |  |  |  |  |  | A68~B18~DR08 | 0.0002 |
|  |  | A30~B13~C06~DR12 | 0.00026 |  |  |  |  |  |  | A30~B27~DR12 | 0.0002 |
|  |  | A24~B44~C02~DR01 | 0.00026 |  |  |  |  |  |  | A31~B37~DR08 | 0.0002 |
|  |  | A01~B51~C02~DR10 | 0.00026 |  |  |  |  |  |  | A01~B14~DR10 | 0.0002 |
|  |  | A02~B44~C17~DR03 | 0.00026 |  |  |  |  |  |  | A66~B13~DR14 | 0.0002 |
|  |  | A31~B35~C15~DR11 | 0.00026 |  |  |  |  |  |  | A33~B13~DR15 | 0.0002 |
|  |  | A68~B35~C03~DR01 | 0.00026 |  |  |  |  |  |  | A32~B83~DR13 | 0.0002 |
|  |  | A02~B27~C12~DR15 | 0.00026 |  |  |  |  |  |  | A30~B50~DR15 | 0.0002 |
|  |  | A01~B07~C18~DR13 | 0.00026 |  |  |  |  |  |  | A25~B41~DR10 | 0.0002 |
|  |  | A03~B27~C15~DR03 | 0.00026 |  |  |  |  |  |  | A11~B58~DR10 | 0.0002 |
|  |  | A66~B35~C07~DR11 | 0.00026 |  |  |  |  |  |  | A66~B35~DR09 | 0.0002 |
|  |  | A66~B49~C07~DR11 | 0.00026 |  |  |  |  |  |  | A31~B52~DR14 | 0.0002 |
|  |  | A01~B39~C07~DR03 | 0.00026 |  |  |  |  |  |  | A26~B40~DR09 | 0.0002 |
|  |  | A02~B58~C07~DR03 | 0.00026 |  |  |  |  |  |  | A66~B47~DR08 | 0.0002 |
|  |  | A26~B27~C01~DR07 | 0.00026 |  |  |  |  |  |  | A68~B38~DR09 | 0.0002 |
|  |  | A02~B15~C12~DR08 | 0.00026 |  |  |  |  |  |  | A33~B39~DR08 | 0.0002 |
|  |  | A31~B18~C02~DR13 | 0.00026 |  |  |  |  |  |  | A66~B49~DR01 | 0.0002 |
|  |  | A11~B55~C03~DR14 | 0.00026 |  |  |  |  |  |  | A26~B51~DR07 | 0.0001 |
|  |  | A30~B27~C07~DR11 | 0.00026 |  |  |  |  |  |  | A29~B18~DR04 | 0.0001 |
|  |  | A30~B08~C02~DR13 | 0.00026 |  |  |  |  |  |  | A29~B13~DR04 | 0.0001 |
|  |  | A33~B44~C05~DR03 | 0.00026 |  |  |  |  |  |  | A31~B52~DR16 | 0.0001 |
|  |  | A03~B07~C05~DR16 | 0.00026 |  |  |  |  |  |  | A11~B18~DR15 | 0.0001 |
|  |  | A01~B08~C07~DR08 | 0.00026 |  |  |  |  |  |  | A66~B41~DR07 | 0.0001 |
|  |  | A33~B35~C12~DR13 | 0.00026 |  |  |  |  |  |  | A02~B41~DR07 | 0.0001 |
|  |  | A23~B44~C04~DR03 | 0.00026 |  |  |  |  |  |  | A66~B41~DR04 | 0.0001 |
|  |  | A30~B15~C01~DR14 | 0.00026 |  |  |  |  |  |  | A26~B44~DR14 | 0.0001 |
|  |  | A02~B50~C15~DR01 | 0.00026 |  |  |  |  |  |  | A01~B58~DR04 | 0.0001 |
|  |  | A68~B35~C03~DR07 | 0.00026 |  |  |  |  |  |  | A23~B41~DR04 | 0.0001 |
|  |  | A68~B13~C03~DR07 | 0.00026 |  |  |  |  |  |  | A32~B35~DR07 | 0.0001 |
|  |  | A03~B50~C07~DR14 | 0.00026 |  |  |  |  |  |  | A02~B41~DR16 | 0.0001 |
|  |  | A01~B35~C12~DR13 | 0.00026 |  |  |  |  |  |  | A68~B38~DR03 | 0.0001 |
|  |  | A26~B51~C12~DR13 | 0.00026 |  |  |  |  |  |  | A68~B38~DR11 | 0.0001 |
|  |  | A01~B44~C04~DR04 | 0.00026 |  |  |  |  |  |  | A24~B53~DR07 | 0.0001 |
|  |  | A30~B37~C06~DR04 | 0.00026 |  |  |  |  |  |  | A11~B07~DR11 | 0.0001 |
|  |  | A30~B07~C07~DR16 | 0.00026 |  |  |  |  |  |  | A24~B13~DR15 | 0.0001 |
|  |  | A30~B49~C07~DR07 | 0.00026 |  |  |  |  |  |  | A11~B58~DR15 | 0.0001 |
|  |  | A29~B44~C16~DR14 | 0.00026 |  |  |  |  |  |  | A26~B38~DR10 | 0.0001 |
|  |  | A03~B49~C07~DR12 | 0.00026 |  |  |  |  |  |  | A02~B39~DR01 | 0.0001 |
|  |  | A26~B40~C13~DR12 | 0.00026 |  |  |  |  |  |  | A02~B56~DR01 | 0.0001 |
|  |  | A01~B44~C05~DR09 | 0.00026 |  |  |  |  |  |  | A01~B44~DR07 | 0.0001 |
|  |  | A31~B35~C04~DR09 | 0.00026 |  |  |  |  |  |  | A33~B38~DR16 | 0.0001 |
|  |  | A30~B41~C01~DR01 | 0.00026 |  |  |  |  |  |  | A33~B52~DR15 | 0.0001 |
|  |  | A30~B18~C12~DR01 | 0.00026 |  |  |  |  |  |  | A69~B49~DR13 | 0.0001 |
|  |  | A01~B57~C18~DR11 | 0.00026 |  |  |  |  |  |  | A24~B13~DR13 | 0.0001 |
|  |  | A24~B15~C12~DR12 | 0.00026 |  |  |  |  |  |  | A68~B08~DR15 | 0.0001 |
|  |  | A02~B39~C12~DR12 | 0.00026 |  |  |  |  |  |  | A11~B52~DR04 | 0.0001 |
|  |  | A68~B39~C02~DR08 | 0.00026 |  |  |  |  |  |  | A02~B39~DR03 | 0.0001 |
|  |  | A01~B73~C15~DR04 | 0.00026 |  |  |  |  |  |  | A33~B44~DR03 | 0.0001 |
|  |  | A03~B57~C06~DR09 | 0.00026 |  |  |  |  |  |  | A29~B08~DR09 | 0.0001 |
|  |  | A03~B73~C01~DR04 | 0.00026 |  |  |  |  |  |  | A29~B52~DR04 | 0.0001 |
|  |  | A11~B55~C16~DR10 | 0.00026 |  |  |  |  |  |  | A02~B57~DR16 | 0.0001 |
|  |  | A02~B58~C07~DR16 | 0.00026 |  |  |  |  |  |  | A32~B18~DR14 | 0.0001 |
|  |  | A03~B35~C10~DR14 | 0.00026 |  |  |  |  |  |  | A03~B27~DR10 | 0.0001 |
|  |  | A11~B40~C07~DR08 | 0.00026 |  |  |  |  |  |  | A03~B07~DR08 | 0.0001 |
|  |  | A68~B44~C05~DR10 | 0.00026 |  |  |  |  |  |  | A24~B38~DR04 | 0.0001 |
|  |  | A24~B55~C03~DR11 | 0.00026 |  |  |  |  |  |  | A68~B55~DR04 | 0.0001 |
|  |  | A26~B15~C06~DR16 | 0.00026 |  |  |  |  |  |  | A68~B44~DR14 | 0.0001 |
|  |  | A26~B13~C06~DR16 | 0.00026 |  |  |  |  |  |  | A23~B44~DR14 | 0.0001 |
|  |  | A01~B58~C03~DR13 | 0.00026 |  |  |  |  |  |  | A68~B38~DR12 | 0.0001 |
|  |  | A02~B07~C15~DR01 | 0.00026 |  |  |  |  |  |  | A26~B57~DR04 | 0.0001 |
|  |  | A32~B15~C01~DR12 | 0.00026 |  |  |  |  |  |  | A01~B57~DR14 | 0.0001 |
|  |  | A24~B39~C07~DR14 | 0.00026 |  |  |  |  |  |  | A24~B37~DR01 | 0.0001 |
|  |  | A68~B40~C04~DR08 | 0.00026 |  |  |  |  |  |  | A24~B37~DR13 | 0.0001 |
|  |  | A32~B08~C07~DR04 | 0.00026 |  |  |  |  |  |  | A11~B51~DR01 | 0.0001 |
|  |  | A03~B51~C04~DR07 | 0.00026 |  |  |  |  |  |  | A31~B15~DR13 | 0.0001 |
|  |  | A36~B35~C04~DR15 | 0.00026 |  |  |  |  |  |  | A24~B44~DR15 | 0.0001 |
|  |  | A24~B14~C08~DR03 | 0.00026 |  |  |  |  |  |  | A30~B07~DR10 | 0.0001 |
|  |  | A31~B15~C03~DR07 | 0.00026 |  |  |  |  |  |  | A32~B37~DR11 | 0.0001 |
|  |  | A24~B46~C01~DR09 | 0.00026 |  |  |  |  |  |  | A23~B49~DR09 | 0.0001 |
|  |  | A02~B51~C14~DR07 | 0.00026 |  |  |  |  |  |  | A68~B07~DR01 | 0.0001 |
|  |  | A68~B35~C15~DR04 | 0.00026 |  |  |  |  |  |  | A02~B55~DR15 | 0.0001 |
|  |  | A33~B50~C06~DR03 | 0.00026 |  |  |  |  |  |  | A33~B40~DR13 | 0.0001 |
|  |  | A29~B07~C15~DR07 | 0.00026 |  |  |  |  |  |  | A02~B58~DR13 | 0.0001 |
|  |  | A03~B18~C01~DR04 | 0.00026 |  |  |  |  |  |  | A29~B27~DR11 | 0.0001 |
|  |  | A31~B51~C15~DR03 | 0.00026 |  |  |  |  |  |  | A29~B27~DR01 | 0.0001 |
|  |  | A01~B48~C08~DR09 | 0.00026 |  |  |  |  |  |  | A68~B18~DR15 | 0.0001 |
|  |  | A03~B13~C07~DR11 | 0.00026 |  |  |  |  |  |  | A32~B39~DR10 | 0.0001 |
|  |  | A01~B52~C12~DR12 | 0.00026 |  |  |  |  |  |  | A32~B39~DR04 | 0.0001 |
|  |  | A36~B52~C08~DR13 | 0.00026 |  |  |  |  |  |  | A11~B41~DR03 | 0.0001 |
|  |  | A11~B48~C08~DR14 | 0.00026 |  |  |  |  |  |  | A32~B15~DR10 | 0.0001 |
|  |  | A02~B48~C08~DR14 | 0.00026 |  |  |  |  |  |  | A03~B15~DR16 | 0.0001 |
|  |  | A68~B15~C03~DR12 | 0.00026 |  |  |  |  |  |  | A11~B15~DR14 | 0.0001 |
|  |  | A31~B35~C08~DR03 | 0.00026 |  |  |  |  |  |  | A03~B14~DR13 | 0.0001 |
|  |  | A31~B18~C08~DR14 | 0.00026 |  |  |  |  |  |  | A03~B08~DR13 | 0.0001 |
|  |  | A29~B44~C04~DR04 | 0.00026 |  |  |  |  |  |  | A29~B37~DR01 | 0.0001 |
|  |  | A02~B55~C16~DR04 | 0.00026 |  |  |  |  |  |  | A33~B15~DR15 | 0.0001 |
|  |  | A30~B07~C14~DR10 | 0.00026 |  |  |  |  |  |  | A30~B18~DR10 | 0.0001 |
|  |  | A01~B48~C08~DR14 | 0.00026 |  |  |  |  |  |  | A30~B35~DR10 | 0.0001 |
|  |  | A23~B15~C14~DR03 | 0.00026 |  |  |  |  |  |  | A26~B49~DR04 | 0.0001 |
|  |  | A01~B57~C02~DR10 | 0.00026 |  |  |  |  |  |  | A02~B39~DR07 | 0.0001 |
|  |  | A02~B27~C14~DR03 | 0.00026 |  |  |  |  |  |  | A24~B55~DR16 | 0.0001 |
|  |  | A03~B18~C12~DR09 | 0.00026 |  |  |  |  |  |  | A01~B18~DR14 | 0.0001 |
|  |  | A03~B35~C12~DR09 | 0.00026 |  |  |  |  |  |  | A03~B18~DR14 | 0.0001 |
|  |  | A68~B58~C07~DR08 | 0.00026 |  |  |  |  |  |  | A11~B37~DR13 | 0.0001 |
|  |  | A11~B15~C08~DR14 | 0.00026 |  |  |  |  |  |  | A33~B14~DR12 | 0.0001 |
|  |  | A26~B18~C08~DR03 | 0.00026 |  |  |  |  |  |  | A03~B39~DR11 | 0.0001 |
|  |  | A24~B50~C06~DR15 | 0.00026 |  |  |  |  |  |  | A68~B07~DR16 | 0.0001 |
|  |  | A25~B52~C07~DR15 | 0.00026 |  |  |  |  |  |  | A31~B49~DR01 | 0.0001 |
|  |  | A66~B52~C07~DR07 | 0.00026 |  |  |  |  |  |  | A26~B15~DR15 | 0.0001 |
|  |  | A30~B51~C16~DR16 | 0.00026 |  |  |  |  |  |  | A31~B52~DR15 | 0.0001 |
|  |  | A01~B44~C05~DR12 | 0.00026 |  |  |  |  |  |  | A23~B51~DR16 | 0.0001 |
|  |  | A24~B40~C03~DR12 | 0.00026 |  |  |  |  |  |  | A23~B08~DR14 | 0.0001 |
|  |  | A31~B48~C08~DR15 | 0.00026 |  |  |  |  |  |  | A29~B14~DR04 | 0.0001 |
|  |  | A26~B44~C03~DR09 | 0.00026 |  |  |  |  |  |  | A29~B55~DR04 | 0.0001 |
|  |  | A33~B49~C07~DR15 | 0.00026 |  |  |  |  |  |  | A01~B13~DR04 | 0.0001 |
|  |  | A30~B15~C07~DR03 | 0.00026 |  |  |  |  |  |  | A31~B27~DR14 | 0.0001 |
|  |  | A01~B27~C07~DR04 | 0.00026 |  |  |  |  |  |  | A31~B35~DR14 | 0.0001 |
|  |  | A31~B49~C07~DR16 | 0.00026 |  |  |  |  |  |  | A11~B18~DR07 | 0.0001 |
|  |  | A24~B40~C12~DR16 | 0.00026 |  |  |  |  |  |  | A23~B50~DR16 | 0.0001 |
|  |  | A02~B15~C15~DR08 | 0.00026 |  |  |  |  |  |  | A23~B50~DR11 | 0.0001 |
|  |  | A32~B35~C15~DR15 | 0.00026 |  |  |  |  |  |  | A01~B58~DR13 | 0.0001 |
|  |  | A02~B07~C15~DR08 | 0.00026 |  |  |  |  |  |  | A33~B52~DR08 | 0.0001 |
|  |  | A31~B35~C12~DR07 | 0.00026 |  |  |  |  |  |  | A26~B08~DR07 | 0.0001 |
|  |  | A66~B49~C17~DR15 | 0.00026 |  |  |  |  |  |  | A03~B45~DR11 | 0.0001 |
|  |  | A26~B35~C04~DR10 | 0.00026 |  |  |  |  |  |  | A03~B45~DR15 | 0.0001 |
|  |  | A03~B45~C16~DR11 | 0.00026 |  |  |  |  |  |  | A29~B52~DR11 | 0.0001 |
|  |  | A24~B15~C01~DR12 | 0.00026 |  |  |  |  |  |  | A11~B27~DR03 | 0.0001 |
|  |  | A68~B57~C07~DR13 | 0.00026 |  |  |  |  |  |  | A01~B40~DR07 | 0.0001 |
|  |  | A68~B13~C16~DR16 | 0.00026 |  |  |  |  |  |  | A01~B40~DR10 | 0.0001 |
|  |  | A03~B51~C07~DR10 | 0.00026 |  |  |  |  |  |  | A03~B41~DR10 | 0.0001 |
|  |  | A68~B46~C01~DR08 | 0.00026 |  |  |  |  |  |  | A23~B51~DR01 | 0.0001 |
|  |  | A29~B51~C15~DR14 | 0.00026 |  |  |  |  |  |  | A23~B27~DR10 | 0.0001 |
|  |  | A26~B15~C12~DR14 | 0.00026 |  |  |  |  |  |  | A11~B39~DR15 | 0.0001 |
|  |  | A24~B15~C06~DR04 | 0.00026 |  |  |  |  |  |  | A29~B07~DR07 | 0.0001 |
|  |  | A03~B14~C08~DR08 | 0.00026 |  |  |  |  |  |  | A29~B07~DR03 | 0.0001 |
|  |  | A11~B39~C12~DR09 | 0.00026 |  |  |  |  |  |  | A24~B57~DR13 | 0.0001 |
|  |  | A30~B38~C06~DR11 | 0.00026 |  |  |  |  |  |  | A26~B45~DR11 | 0.0001 |
|  |  | A33~B49~C07~DR03 | 0.00026 |  |  |  |  |  |  | A26~B39~DR15 | 0.0001 |
|  |  | A30~B13~C04~DR15 | 0.00026 |  |  |  |  |  |  | A11~B39~DR03 | 0.0001 |
|  |  | A26~B35~C17~DR13 | 0.00026 |  |  |  |  |  |  | A02~B60~DR11 | 0.0001 |
|  |  | A03~B51~C16~DR03 | 0.00026 |  |  |  |  |  |  | A02~B44~DR09 | 0.0001 |
|  |  | A01~B58~C06~DR03 | 0.00026 |  |  |  |  |  |  | A26~B08~DR04 | 0.0001 |
|  |  | A30~B37~C06~DR11 | 0.00026 |  |  |  |  |  |  | A26~B51~DR10 | 0.0001 |
|  |  | A26~B51~C07~DR16 | 0.00026 |  |  |  |  |  |  | A11~B52~DR07 | 0.0001 |
|  |  | A29~B27~C02~DR04 | 0.00026 |  |  |  |  |  |  | A03~B41~DR13 | 0.0001 |
|  |  | A31~B35~C04~DR03 | 0.00026 |  |  |  |  |  |  | A33~B35~DR01 | 0.0001 |
|  |  | A02~B48~C08~DR12 | 0.00026 |  |  |  |  |  |  | A03~B49~DR03 | 0.0001 |
|  |  | A26~B41~C07~DR03 | 0.00026 |  |  |  |  |  |  | A26~B50~DR10 | 0.0001 |
|  |  | A33~B18~C12~DR14 | 0.00026 |  |  |  |  |  |  | A01~B50~DR10 | 0.0001 |
|  |  | A31~B27~C02~DR16 | 0.00026 |  |  |  |  |  |  | A03~B40~DR10 | 0.0001 |
|  |  | A01~B18~C04~DR16 | 0.00026 |  |  |  |  |  |  | A33~B14~DR15 | 0.0001 |
|  |  | A24~B15~C03~DR12 | 0.00026 |  |  |  |  |  |  | A01~B48~DR04 | 0.0001 |
|  |  | A68~B51~C16~DR04 | 0.00026 |  |  |  |  |  |  | A26~B48~DR01 | 0.0001 |
|  |  | A29~B44~C16~DR15 | 0.00026 |  |  |  |  |  |  | A02~B39~DR14 | 0.0001 |
|  |  | A11~B44~C16~DR01 | 0.00026 |  |  |  |  |  |  | A36~B18~DR08 | 0.0001 |
|  |  | A01~B49~C07~DR12 | 0.00026 |  |  |  |  |  |  | A30~B53~DR13 | 0.0001 |
|  |  | A68~B44~C14~DR15 | 0.00026 |  |  |  |  |  |  | A30~B55~DR16 | 0.0001 |
|  |  | A01~B07~C07~DR10 | 0.00026 |  |  |  |  |  |  | A31~B13~DR13 | 0.0001 |
|  |  | A25~B52~C02~DR10 | 0.00026 |  |  |  |  |  |  | A68~B49~DR13 | 0.0001 |
|  |  | A11~B18~C07~DR10 | 0.00026 |  |  |  |  |  |  | A30~B15~DR13 | 0.0001 |
|  |  | A11~B27~C02~DR15 | 0.00026 |  |  |  |  |  |  | A01~B52~DR13 | 0.0001 |
|  |  | A32~B15~C03~DR11 | 0.00026 |  |  |  |  |  |  | A33~B58~DR15 | 0.0001 |
|  |  | A26~B35~C12~DR14 | 0.00026 |  |  |  |  |  |  | A33~B58~DR14 | 0.0001 |
|  |  | A02~B40~C08~DR01 | 0.00026 |  |  |  |  |  |  | A02~B08~DR07 | 0.0001 |
|  |  | A24~B51~C05~DR01 | 0.00026 |  |  |  |  |  |  | A66~B15~DR04 | 0.0001 |
|  |  | A24~B35~C16~DR11 | 0.00026 |  |  |  |  |  |  | A03~B55~DR16 | 0.0001 |
|  |  | A24~B13~C06~DR03 | 0.00026 |  |  |  |  |  |  | A03~B57~DR13 | 0.0001 |
|  |  | A24~B56~C01~DR11 | 0.00025 |  |  |  |  |  |  | A23~B41~DR08 | 0.0001 |
|  |  | A02~B27~C02~DR07 | 0.00025 |  |  |  |  |  |  | A01~B41~DR08 | 0.0001 |
|  |  | A11~B52~C12~DR08 | 0.00025 |  |  |  |  |  |  | A24~B49~DR01 | 0.0001 |
|  |  | A33~B41~C17~DR15 | 0.00025 |  |  |  |  |  |  | A68~B18~DR12 | 0.0001 |
|  |  | A33~B51~C02~DR11 | 0.00025 |  |  |  |  |  |  | A31~B14~DR01 | 0.0001 |
|  |  | A29~B07~C07~DR07 | 0.00024 |  |  |  |  |  |  | A01~B14~DR14 | 0.0001 |
|  |  | A23~B51~C04~DR13 | 0.00024 |  |  |  |  |  |  | A66~B41~DR01 | 0.0001 |
|  |  | A11~B40~C03~DR14 | 0.00024 |  |  |  |  |  |  | A66~B35~DR01 | 0.0001 |
|  |  | A11~B51~C04~DR13 | 0.00024 |  |  |  |  |  |  | A02~B57~DR09 | 0.0001 |
|  |  | A01~B55~C03~DR04 | 0.00024 |  |  |  |  |  |  | A68~B27~DR09 | 0.0001 |
|  |  | A26~B44~C04~DR03 | 0.00024 |  |  |  |  |  |  | A31~B18~DR15 | 0.0001 |
|  |  | A03~B27~C02~DR03 | 0.00024 |  |  |  |  |  |  | A31~B59~DR15 | 0.0001 |
|  |  | A11~B55~C02~DR04 | 0.00024 |  |  |  |  |  |  | A68~B37~DR15 | 0.0001 |
|  |  | A68~B40~C01~DR14 | 0.00023 |  |  |  |  |  |  | A68~B37~DR14 | 0.0001 |
|  |  | A11~B52~C07~DR12 | 0.00023 |  |  |  |  |  |  | A24~B14~DR16 | 0.0001 |
|  |  | A01~B15~C03~DR11 | 0.00022 |  |  |  |  |  |  | A01~B14~DR16 | 0.0001 |
|  |  | A32~B51~C15~DR03 | 0.00022 |  |  |  |  |  |  | A33~B07~DR16 | 0.0001 |
|  |  | A31~B44~C04~DR07 | 0.00022 |  |  |  |  |  |  | A31~B07~DR01 | 0.0001 |
|  |  | A03~B40~C02~DR11 | 0.00022 |  |  |  |  |  |  | A31~B35~DR01 | 0.0001 |
|  |  | A25~B55~C03~DR13 | 0.00021 |  |  |  |  |  |  | A11~B39~DR04 | 0.0001 |
|  |  | A11~B39~C12~DR03 | 0.00021 |  |  |  |  |  |  | A24~B35~DR09 | 0.0001 |
|  |  | A02~B44~C07~DR07 | 0.00021 |  |  |  |  |  |  | A03~B50~DR01 | 0.0001 |
|  |  | A26~B15~C07~DR07 | 0.0002 |  |  |  |  |  |  | A26~B44~DR01 | 0.0001 |
|  |  | A68~B27~C02~DR11 | 0.0002 |  |  |  |  |  |  | A33~B50~DR14 | 0.0001 |
|  |  | A02~B44~C04~DR03 | 0.0002 |  |  |  |  |  |  | A02~B14~DR11 | 0.0001 |
|  |  | A68~B07~C07~DR16 | 0.0002 |  |  |  |  |  |  | A03~B38~DR09 | 0.0001 |
|  |  | A32~B50~C15~DR04 | 0.00019 |  |  |  |  |  |  | A31~B57~DR07 | 0.0001 |
|  |  | A31~B15~C07~DR15 | 0.00018 |  |  |  |  |  |  | A24~B48~DR13 | 0.0001 |
|  |  | A01~B49~C12~DR04 | 0.00018 |  |  |  |  |  |  | A26~B48~DR16 | 0.0001 |
|  |  | A31~B15~C03~DR10 | 0.00018 |  |  |  |  |  |  | A26~B27~DR15 | 0.0001 |
|  |  | A32~B40~C02~DR11 | 0.00017 |  |  |  |  |  |  | A30~B08~DR13 | 0.0001 |
|  |  | A24~B55~C01~DR08 | 0.00017 |  |  |  |  |  |  | A24~B08~DR08 | 0.0001 |
|  |  | A03~B48~C08~DR12 | 0.00017 |  |  |  |  |  |  | A03~B40~DR08 | 0.0001 |
|  |  | A26~B57~C06~DR13 | 0.00016 |  |  |  |  |  |  | A03~B58~DR08 | 0.0001 |
|  |  | A68~B51~C15~DR16 | 0.00016 |  |  |  |  |  |  | A11~B58~DR01 | 0.0001 |
|  |  | A26~B44~C04~DR14 | 0.00014 |  |  |  |  |  |  | A30~B14~DR11 | 0.0001 |
|  |  | A03~B35~C12~DR01 | 0.00011 |  |  |  |  |  |  | A30~B44~DR11 | 0.0001 |
|  |  | A24~B44~C01~DR13 | 0.0001 |  |  |  |  |  |  | A25~B35~DR13 | 0.0001 |
|  |  | A01~B40~C03~DR15 | 0.00009 |  |  |  |  |  |  | A03~B13~DR15 | 0.0001 |
|  |  | A11~B39~C12~DR10 | 0.00008 |  |  |  |  |  |  | A32~B27~DR14 | 0.0001 |
|  |  | A11~B41~C17~DR10 | 0.00008 |  |  |  |  |  |  | A32~B15~DR15 | 0.0001 |
|  |  | A03~B51~C01~DR04 | 0.00007 |  |  |  |  |  |  | A01~B56~DR13 | 0.0001 |
|  |  | A69~B18~C07~DR09 | 0.00006 |  |  |  |  |  |  | A25~B55~DR07 | 0.0001 |
|  |  | A02~B27~C02~DR13 | 0.00006 |  |  |  |  |  |  | A11~B18~DR14 | 0.0001 |
|  |  | A29~B44~C05~DR03 | 0.00006 |  |  |  |  |  |  | A23~B45~DR04 | 0.0001 |
|  |  | A23~B35~C06~DR13 | 0.00006 |  |  |  |  |  |  | A68~B39~DR04 | 0.0001 |
|  |  | A11~B14~C08~DR01 | 0.00006 |  |  |  |  |  |  | A30~B39~DR12 | 0.0001 |
|  |  | A26~B44~C05~DR10 | 0.00005 |  |  |  |  |  |  | A24~B59~DR01 | 0.0001 |
|  |  | A24~B44~C16~DR10 | 0.00004 |  |  |  |  |  |  | A03~B15~DR08 | 0.0001 |
|  |  | A03~B03~C04~DR04 | 0.00003 |  |  |  |  |  |  | A33~B38~DR13 | 0.0001 |
|  |  | A24~B24~C07~DR16 | 0.00003 |  |  |  |  |  |  | A11~B40~DR07 | 0.0001 |
|  |  | A03~B24~C04~DR04 | 0.00003 |  |  |  |  |  |  | A11~B15~DR01 | 0.0001 |
|  |  | A24~B03~C07~DR16 | 0.00003 |  |  |  |  |  |  | A33~B27~DR08 | 0.0001 |
|  |  | A03~B03~C07~DR04 | 0.00003 |  |  |  |  |  |  | A29~B44~DR13 | 0.0001 |
|  |  | A24~B24~C04~DR16 | 0.00003 |  |  |  |  |  |  | A01~B59~DR14 | 0.0001 |
|  |  | A03~B24~C07~DR04 | 0.00003 |  |  |  |  |  |  | A02~B53~DR13 | 0.0001 |
|  |  | A24~B03~C04~DR16 | 0.00003 |  |  |  |  |  |  | A03~B08~DR12 | 0.0001 |
|  |  | A03~B03~C04~DR16 | 0.00003 |  |  |  |  |  |  | A26~B18~DR03 | 0.0001 |
|  |  | A24~B24~C07~DR04 | 0.00003 |  |  |  |  |  |  | A26~B39~DR08 | 0.0001 |
|  |  | A03~B24~C04~DR16 | 0.00003 |  |  |  |  |  |  | A29~B18~DR15 | 0.0001 |
|  |  | A24~B03~C07~DR04 | 0.00003 |  |  |  |  |  |  | A25~B27~DR07 | 0.0001 |
|  |  | A03~B03~C07~DR16 | 0.00003 |  |  |  |  |  |  | A26~B40~DR16 | 0.0001 |
|  |  | A24~B24~C04~DR04 | 0.00003 |  |  |  |  |  |  | A03~B56~DR15 | 0.0001 |
|  |  | A03~B24~C07~DR16 | 0.00003 |  |  |  |  |  |  | A11~B57~DR04 | 0.0001 |
|  |  | A24~B03~C04~DR04 | 0.00003 |  |  |  |  |  |  | A02~B14~DR15 | 0.0001 |
|  |  | A68~B35~C04~DR15 | 0.00002 |  |  |  |  |  |  | A11~B44~DR16 | 0.0001 |
|  |  |  |  |  |  |  |  |  |  | A68~B14~DR12 | 0.0001 |
|  |  |  |  |  |  |  |  |  |  | A03~B14~DR11 | 0.0001 |
|  |  |  |  |  |  |  |  |  |  | A26~B40~DR08 | 0.0001 |
|  |  |  |  |  |  |  |  |  |  | A68~B44~DR03 | 0.0001 |
|  |  |  |  |  |  |  |  |  |  | A68~B07~DR03 | 0.0001 |
|  |  |  |  |  |  |  |  |  |  | A24~B55~DR03 | 0.0001 |
|  |  |  |  |  |  |  |  |  |  | A02~B38~DR03 | 0.0001 |
|  |  |  |  |  |  |  |  |  |  | A29~B44~DR14 | 0.0001 |
|  |  |  |  |  |  |  |  |  |  | A29~B38~DR14 | 0.0001 |
|  |  |  |  |  |  |  |  |  |  | A33~B38~DR07 | 0.0001 |
|  |  |  |  |  |  |  |  |  |  | A33~B37~DR16 | 0.0001 |
|  |  |  |  |  |  |  |  |  |  | A26~B41~DR11 | 0.0001 |
|  |  |  |  |  |  |  |  |  |  | A25~B08~DR03 | 0.0001 |
|  |  |  |  |  |  |  |  |  |  | A33~B55~DR07 | 0.0001 |
|  |  |  |  |  |  |  |  |  |  | A66~B14~DR04 | 0.0001 |
|  |  |  |  |  |  |  |  |  |  | A66~B55~DR13 | 0.0001 |
|  |  |  |  |  |  |  |  |  |  | A23~B15~DR13 | 0.0001 |
|  |  |  |  |  |  |  |  |  |  | A33~B41~DR16 | 0.0001 |
|  |  |  |  |  |  |  |  |  |  | A31~B48~DR04 | 0.0001 |
|  |  |  |  |  |  |  |  |  |  | A33~B14~DR08 | 0.0001 |
|  |  |  |  |  |  |  |  |  |  | A29~B58~DR15 | 0.0001 |
|  |  |  |  |  |  |  |  |  |  | A30~B39~DR13 | 0.0001 |
|  |  |  |  |  |  |  |  |  |  | A30~B27~DR08 | 0.0001 |
|  |  |  |  |  |  |  |  |  |  | A23~B35~DR14 | 0.0001 |
|  |  |  |  |  |  |  |  |  |  | A33~B13~DR01 | 0.0001 |
|  |  |  |  |  |  |  |  |  |  | A11~B52~DR12 | 0.0001 |
|  |  |  |  |  |  |  |  |  |  | A33~B49~DR03 | 0.0001 |
|  |  |  |  |  |  |  |  |  |  | A33~B49~DR15 | 0.0001 |
|  |  |  |  |  |  |  |  |  |  | A32~B51~DR12 | 0.0001 |
|  |  |  |  |  |  |  |  |  |  | A29~B39~DR14 | 0.0001 |
|  |  |  |  |  |  |  |  |  |  | A31~B55~DR16 | 0.0001 |
|  |  |  |  |  |  |  |  |  |  | A23~B38~DR04 | 0.0001 |
|  |  |  |  |  |  |  |  |  |  | A25~B58~DR03 | 0.0001 |
|  |  |  |  |  |  |  |  |  |  | A11~B13~DR12 | 0.0001 |
|  |  |  |  |  |  |  |  |  |  | A02~B55~DR01 | 0.0001 |
|  |  |  |  |  |  |  |  |  |  | A68~B13~DR03 | 0.0001 |
|  |  |  |  |  |  |  |  |  |  | A24~B50~DR09 | 0.0001 |
|  |  |  |  |  |  |  |  |  |  | A30~B53~DR11 | 0.0001 |
|  |  |  |  |  |  |  |  |  |  | A26~B27~DR16 | 0.0001 |
|  |  |  |  |  |  |  |  |  |  | A25~B40~DR01 | 0.0001 |
|  |  |  |  |  |  |  |  |  |  | A30~B40~DR03 | 0.0001 |
|  |  |  |  |  |  |  |  |  |  | A26~B27~DR12 | 0.0001 |
|  |  |  |  |  |  |  |  |  |  | A69~B08~DR16 | 0.0001 |
|  |  |  |  |  |  |  |  |  |  | A32~B50~DR15 | 0.0001 |
|  |  |  |  |  |  |  |  |  |  | A32~B08~DR04 | 0.0001 |
|  |  |  |  |  |  |  |  |  |  | A68~B55~DR07 | 0.0001 |
|  |  |  |  |  |  |  |  |  |  | A29~B13~DR11 | 0.0001 |
|  |  |  |  |  |  |  |  |  |  | A26~B13~DR16 | 0.0001 |
|  |  |  |  |  |  |  |  |  |  | A68~B55~DR15 | 0.0001 |
|  |  |  |  |  |  |  |  |  |  | A30~B07~DR16 | 0.0001 |
|  |  |  |  |  |  |  |  |  |  | A26~B45~DR13 | 0.0001 |
|  |  |  |  |  |  |  |  |  |  | A68~B50~DR10 | 0.0001 |
|  |  |  |  |  |  |  |  |  |  | A32~B56~DR13 | 0.0001 |
|  |  |  |  |  |  |  |  |  |  | A29~B08~DR01 | 0.0001 |
|  |  |  |  |  |  |  |  |  |  | A68~B38~DR04 | 0.0001 |
|  |  |  |  |  |  |  |  |  |  | A69~B08~DR07 | 0.0001 |
|  |  |  |  |  |  |  |  |  |  | A01~B07~DR12 | 0.0001 |
|  |  |  |  |  |  |  |  |  |  | A01~B48~DR12 | 0.0001 |
|  |  |  |  |  |  |  |  |  |  | A29~B13~DR03 | 0.0001 |
|  |  |  |  |  |  |  |  |  |  | A01~B27~DR01 | 0.0001 |
|  |  |  |  |  |  |  |  |  |  | A32~B39~DR13 | 0.0001 |
|  |  |  |  |  |  |  |  |  |  | A30~B38~DR16 | 0.0001 |
|  |  |  |  |  |  |  |  |  |  | A31~B58~DR16 | 0.0001 |
|  |  |  |  |  |  |  |  |  |  | A32~B58~DR08 | 0.0001 |
|  |  |  |  |  |  |  |  |  |  | A24~B14~DR08 | 0.0001 |
|  |  |  |  |  |  |  |  |  |  | A31~B40~DR14 | 0.0001 |
|  |  |  |  |  |  |  |  |  |  | A23~B15~DR14 | 0.0001 |
|  |  |  |  |  |  |  |  |  |  | A01~B48~DR03 | 0.0001 |
|  |  |  |  |  |  |  |  |  |  | A01~B47~DR11 | 0.0001 |
|  |  |  |  |  |  |  |  |  |  | A32~B27~DR15 | 0.0001 |
|  |  |  |  |  |  |  |  |  |  | A25~B50~DR12 | 0.0001 |
|  |  |  |  |  |  |  |  |  |  | A30~B53~DR15 | 0.0001 |
|  |  |  |  |  |  |  |  |  |  | A33~B15~DR04 | 0.0001 |
|  |  |  |  |  |  |  |  |  |  | A66~B07~DR03 | 0.0001 |
|  |  |  |  |  |  |  |  |  |  | A01~B48~DR15 | 0.0001 |
|  |  |  |  |  |  |  |  |  |  | A23~B15~DR15 | 0.0001 |
|  |  |  |  |  |  |  |  |  |  | A23~B38~DR13 | 0.0001 |
|  |  |  |  |  |  |  |  |  |  | A68~B39~DR07 | 0.0001 |
|  |  |  |  |  |  |  |  |  |  | A68~B39~DR13 | 0.0001 |
|  |  |  |  |  |  |  |  |  |  | A66~B40~DR13 | 0.0001 |
|  |  |  |  |  |  |  |  |  |  | A30~B38~DR08 | 0.0001 |
|  |  |  |  |  |  |  |  |  |  | A30~B48~DR08 | 0.0001 |
|  |  |  |  |  |  |  |  |  |  | A32~B53~DR11 | 0.0001 |
|  |  |  |  |  |  |  |  |  |  | A33~B13~DR03 | 0.0001 |
|  |  |  |  |  |  |  |  |  |  | A03~B57~DR10 | 0.0001 |
|  |  |  |  |  |  |  |  |  |  | A23~B13~DR09 | 0.0001 |
|  |  |  |  |  |  |  |  |  |  | A30~B13~DR09 | 0.0001 |
|  |  |  |  |  |  |  |  |  |  | A03~B50~DR14 | 0.0001 |
|  |  |  |  |  |  |  |  |  |  | A38~B44~DR07 | 0.0001 |
|  |  |  |  |  |  |  |  |  |  | A25~B44~DR03 | 0.0001 |
|  |  |  |  |  |  |  |  |  |  | A01~B44~DR08 | 0.0001 |
|  |  |  |  |  |  |  |  |  |  | A01~B56~DR08 | 0.0001 |
|  |  |  |  |  |  |  |  |  |  | A26~B15~DR07 | 0.0001 |
|  |  |  |  |  |  |  |  |  |  | A23~B56~DR04 | 0.0001 |
|  |  |  |  |  |  |  |  |  |  | A03~B55~DR09 | 0.0001 |
|  |  |  |  |  |  |  |  |  |  | A26~B40~DR13 | 0.0001 |
|  |  |  |  |  |  |  |  |  |  | A29~B44~DR07 | 0.0001 |
|  |  |  |  |  |  |  |  |  |  | A33~B56~DR01 | 0.0001 |
|  |  |  |  |  |  |  |  |  |  | A33~B39~DR04 | 0.0001 |
|  |  |  |  |  |  |  |  |  |  | A33~B40~DR16 | 0.0001 |
|  |  |  |  |  |  |  |  |  |  | A01~B53~DR14 | 0.0001 |
|  |  |  |  |  |  |  |  |  |  | A11~B59~DR11 | 0.0001 |
|  |  |  |  |  |  |  |  |  |  | A31~B15~DR15 | 0.0001 |
|  |  |  |  |  |  |  |  |  |  | A11~B52~DR14 | 0.0001 |
|  |  |  |  |  |  |  |  |  |  | A29~B51~DR07 | 0.0001 |
|  |  |  |  |  |  |  |  |  |  | A01~B53~DR15 | 0.0001 |
|  |  |  |  |  |  |  |  |  |  | A22~B55~DR14 | 0.0001 |
|  |  |  |  |  |  |  |  |  |  | A33~B38~DR15 | 0.0001 |
|  |  |  |  |  |  |  |  |  |  | A30~B38~DR14 | 0.0001 |
|  |  |  |  |  |  |  |  |  |  | A30~B08~DR11 | 0.0001 |
|  |  |  |  |  |  |  |  |  |  | A69~B55~DR03 | 0.0001 |
|  |  |  |  |  |  |  |  |  |  | A26~B14~DR03 | 0.0001 |
|  |  |  |  |  |  |  |  |  |  | A29~B38~DR11 | 0.0001 |
|  |  |  |  |  |  |  |  |  |  | A23~B57~DR01 | 0.0001 |
|  |  |  |  |  |  |  |  |  |  | A11~B03~DR03 | 0.0001 |
|  |  |  |  |  |  |  |  |  |  | A02~B19~DR03 | 0.0001 |
|  |  |  |  |  |  |  |  |  |  | A30~B40~DR04 | 0.0001 |
|  |  |  |  |  |  |  |  |  |  | A32~B07~DR12 | 0.0001 |
|  |  |  |  |  |  |  |  |  |  | A30~B49~DR01 | 0.0001 |
|  |  |  |  |  |  |  |  |  |  | A23~B52~DR07 | 0.0001 |
|  |  |  |  |  |  |  |  |  |  | A68~B42~DR03 | 0.0001 |
|  |  |  |  |  |  |  |  |  |  | A25~B41~DR13 | 0.0001 |
|  |  |  |  |  |  |  |  |  |  | A25~B07~DR03 | 0.0001 |
|  |  |  |  |  |  |  |  |  |  | A30~B51~DR10 | 0.0001 |
|  |  |  |  |  |  |  |  |  |  | A26~B44~DR12 | 0.0001 |
|  |  |  |  |  |  |  |  |  |  | A25~B51~DR07 | 0.0001 |
|  |  |  |  |  |  |  |  |  |  | A26~B40~DR12 | 0.0001 |
|  |  |  |  |  |  |  |  |  |  | A24~B15~DR09 | 0.0001 |
|  |  |  |  |  |  |  |  |  |  | A31~B35~DR09 | 0.0001 |
|  |  |  |  |  |  |  |  |  |  | A30~B27~DR14 | 0.0001 |
|  |  |  |  |  |  |  |  |  |  | A68~B27~DR08 | 0.0001 |
|  |  |  |  |  |  |  |  |  |  | A26~B15~DR12 | 0.0001 |
|  |  |  |  |  |  |  |  |  |  | A68~B40~DR03 | 0.0001 |
|  |  |  |  |  |  |  |  |  |  | A26~B48~DR12 | 0.0001 |
|  |  |  |  |  |  |  |  |  |  | A36~B35~DR15 | 0.0001 |
|  |  |  |  |  |  |  |  |  |  | A24~B14~DR03 | 0.0001 |
|  |  |  |  |  |  |  |  |  |  | A69~B41~DR07 | 0.0001 |
|  |  |  |  |  |  |  |  |  |  | A68~B56~DR03 | 0.0001 |
|  |  |  |  |  |  |  |  |  |  | A69~B13~DR03 | 0.0001 |
|  |  |  |  |  |  |  |  |  |  | A33~B50~DR03 | 0.0001 |
|  |  |  |  |  |  |  |  |  |  | A02~B39~DR10 | 0.0001 |
|  |  |  |  |  |  |  |  |  |  | A01~B48~DR09 | 0.0001 |
|  |  |  |  |  |  |  |  |  |  | A01~B52~DR12 | 0.0001 |
|  |  |  |  |  |  |  |  |  |  | A01~B48~DR14 | 0.0001 |
|  |  |  |  |  |  |  |  |  |  | A11~B48~DR12 | 0.0001 |
|  |  |  |  |  |  |  |  |  |  | A03~B56~DR13 | 0.0001 |
|  |  |  |  |  |  |  |  |  |  | A68~B07~DR08 | 0.0001 |
|  |  |  |  |  |  |  |  |  |  | A68~B34~DR13 | 0.0001 |
|  |  |  |  |  |  |  |  |  |  | A68~B14~DR03 | 0.0001 |
|  |  |  |  |  |  |  |  |  |  | A32~B14~DR07 | 0.0001 |
|  |  |  |  |  |  |  |  |  |  | A68~B55~DR14 | 0.0001 |
|  |  |  |  |  |  |  |  |  |  | A31~B40~DR09 | 0.0001 |
|  |  |  |  |  |  |  |  |  |  | A26~B52~DR10 | 0.0001 |
|  |  |  |  |  |  |  |  |  |  | A24~B54~DR14 | 0.0001 |
|  |  |  |  |  |  |  |  |  |  | A01~B47~DR10 | 0.0001 |
|  |  |  |  |  |  |  |  |  |  | A30~B38~DR12 | 0.0001 |
|  |  |  |  |  |  |  |  |  |  | A69~B46~DR13 | 0.0001 |
|  |  |  |  |  |  |  |  |  |  | A30~B27~DR10 | 0.0001 |
|  |  |  |  |  |  |  |  |  |  | A03~B53~DR03 | 0.0001 |
|  |  |  |  |  |  |  |  |  |  | A30~B08~DR14 | 0.0001 |
|  |  |  |  |  |  |  |  |  |  | A29~B14~DR16 | 0.0001 |
|  |  |  |  |  |  |  |  |  |  | A29~B14~DR14 | 0.0001 |
|  |  |  |  |  |  |  |  |  |  | A26~B37~DR09 | 0.0001 |
|  |  |  |  |  |  |  |  |  |  | A66~B57~DR04 | 0.0001 |
|  |  |  |  |  |  |  |  |  |  | A66~B52~DR07 | 0.0001 |
|  |  |  |  |  |  |  |  |  |  | A66~B35~DR14 | 0.0001 |
|  |  |  |  |  |  |  |  |  |  | A68~B13~DR16 | 0.0001 |
|  |  |  |  |  |  |  |  |  |  | A31~B49~DR16 | 0.0001 |
|  |  |  |  |  |  |  |  |  |  | A02~B66~DR09 | 0.0001 |
|  |  |  |  |  |  |  |  |  |  | A66~B46~DR09 | 0.0001 |
|  |  |  |  |  |  |  |  |  |  | A25~B50~DR03 | 0.0001 |
|  |  |  |  |  |  |  |  |  |  | A26~B55~DR09 | 0.0001 |
|  |  |  |  |  |  |  |  |  |  | A69~B52~DR07 | 0.0001 |
|  |  |  |  |  |  |  |  |  |  | A24~B48~DR09 | 0.0001 |
|  |  |  |  |  |  |  |  |  |  | A69~B55~DR08 | 0.0001 |
|  |  |  |  |  |  |  |  |  |  | A33~B73~DR08 | 0.0001 |
|  |  |  |  |  |  |  |  |  |  | A29~B49~DR07 | 0.0001 |
|  |  |  |  |  |  |  |  |  |  | A02~B56~DR15 | 0.0001 |
|  |  |  |  |  |  |  |  |  |  | A02~B48~DR15 | 0.0001 |
|  |  |  |  |  |  |  |  |  |  | A31~B48~DR15 | 0.0001 |
|  |  |  |  |  |  |  |  |  |  | A68~B41~DR07 | 0.0001 |
|  |  |  |  |  |  |  |  |  |  | A68~B41~DR08 | 0.0001 |
|  |  |  |  |  |  |  |  |  |  | A24~B37~DR09 | 0.0001 |
|  |  |  |  |  |  |  |  |  |  | A31~B57~DR15 | 0.0001 |
|  |  |  |  |  |  |  |  |  |  | A11~B73~DR04 | 0.0001 |
|  |  |  |  |  |  |  |  |  |  | A23~B58~DR13 | 0.0001 |
|  |  |  |  |  |  |  |  |  |  | A31~B38~DR13 | 0.0001 |
|  |  |  |  |  |  |  |  |  |  | A33~B51~DR08 | 0.0001 |
|  |  |  |  |  |  |  |  |  |  | A29~B44~DR12 | 0.0001 |
|  |  |  |  |  |  |  |  |  |  | A69~B58~DR08 | 0.0001 |
|  |  |  |  |  |  |  |  |  |  | A30~B08~DR04 | 0.0001 |
|  |  |  |  |  |  |  |  |  |  | A02~B82~DR03 | 0.0001 |
|  |  |  |  |  |  |  |  |  |  | A74~B58~DR15 | 0.0001 |
|  |  |  |  |  |  |  |  |  |  | A23~B04~DR10 | 0.0001 |
|  |  |  |  |  |  |  |  |  |  | A69~B15~DR13 | 0.0001 |
|  |  |  |  |  |  |  |  |  |  | A33~B50~DR08 | 0.0001 |
|  |  |  |  |  |  |  |  |  |  | A03~B47~DR15 | 0.0001 |
|  |  |  |  |  |  |  |  |  |  | A01~B08~DR02 | 0.0001 |
|  |  |  |  |  |  |  |  |  |  | A31~B14~DR10 | 0.0001 |
|  |  |  |  |  |  |  |  |  |  | A29~B51~DR14 | 0.0001 |
|  |  |  |  |  |  |  |  |  |  | A32~B58~DR10 | 0.0001 |
|  |  |  |  |  |  |  |  |  |  | A25~B55~DR14 | 0.0001 |
|  |  |  |  |  |  |  |  |  |  | A23~B14~DR08 | 0.0001 |
|  |  |  |  |  |  |  |  |  |  | A68~B39~DR09 | 0.0001 |
|  |  |  |  |  |  |  |  |  |  | A69~B44~DR13 | 0.0001 |
|  |  |  |  |  |  |  |  |  |  | A25~B52~DR03 | 0.0001 |
|  |  |  |  |  |  |  |  |  |  | A23~B50~DR10 | 0.0001 |
|  |  |  |  |  |  |  |  |  |  | A30~B37~DR11 | 0.0001 |
|  |  |  |  |  |  |  |  |  |  | A31~B13~DR15 | 0.0001 |
|  |  |  |  |  |  |  |  |  |  | A01~B40~DR11 | 0.0000 |
|  |  |  |  |  |  |  |  |  |  | A01~B40~DR13 | 0.0000 |
|  |  |  |  |  |  |  |  |  |  | A02~B57~DR11 | 0.0000 |
|  |  |  |  |  |  |  |  |  |  | A03~B15~DR14 | 0.0000 |
|  |  |  |  |  |  |  |  |  |  | A03~B27~DR14 | 0.0000 |
|  |  |  |  |  |  |  |  |  |  | A24~B27~DR14 | 0.0000 |
|  |  |  |  |  |  |  |  |  |  | A26~B35~DR07 | 0.0000 |
|  |  |  |  |  |  |  |  |  |  | A02~B18~DR07 | 0.0000 |
|  |  |  |  |  |  |  |  |  |  | A29~B38~DR01 | 0.0000 |
|  |  |  |  |  |  |  |  |  |  | A29~B18~DR01 | 0.0000 |
|  |  |  |  |  |  |  |  |  |  | A02~B38~DR01 | 0.0000 |
|  |  |  |  |  |  |  |  |  |  | A29~B35~DR04 | 0.0000 |
|  |  |  |  |  |  |  |  |  |  | A01~B18~DR01 | 0.0000 |
|  |  |  |  |  |  |  |  |  |  | A02~B52~DR07 | 0.0000 |
|  |  |  |  |  |  |  |  |  |  | A03~B52~DR01 | 0.0000 |
|  |  |  |  |  |  |  |  |  |  | A31~B52~DR11 | 0.0000 |
|  |  |  |  |  |  |  |  |  |  | A31~B40~DR16 | 0.0000 |
|  |  |  |  |  |  |  |  |  |  | A02~B52~DR16 | 0.0000 |
|  |  |  |  |  |  |  |  |  |  | A31~B40~DR11 | 0.0000 |
|  |  |  |  |  |  |  |  |  |  | A31~B44~DR11 | 0.0000 |
|  |  |  |  |  |  |  |  |  |  | A31~B40~DR07 | 0.0000 |
|  |  |  |  |  |  |  |  |  |  | A68~B44~DR16 | 0.0000 |
|  |  |  |  |  |  |  |  |  |  | A68~B44~DR07 | 0.0000 |
|  |  |  |  |  |  |  |  |  |  | A02~B18~DR13 | 0.0000 |
|  |  |  |  |  |  |  |  |  |  | A11~B15~DR13 | 0.0000 |
|  |  |  |  |  |  |  |  |  |  | A24~B52~DR13 | 0.0000 |
|  |  |  |  |  |  |  |  |  |  | A02~B13~DR11 | 0.0000 |
|  |  |  |  |  |  |  |  |  |  | A30~B35~DR07 | 0.0000 |
|  |  |  |  |  |  |  |  |  |  | A66~B41~DR11 | 0.0000 |
|  |  |  |  |  |  |  |  |  |  | A66~B51~DR07 | 0.0000 |
|  |  |  |  |  |  |  |  |  |  | A66~B51~DR11 | 0.0000 |
|  |  |  |  |  |  |  |  |  |  | A66~B13~DR04 | 0.0000 |
|  |  |  |  |  |  |  |  |  |  | A66~B35~DR07 | 0.0000 |
|  |  |  |  |  |  |  |  |  |  | A66~B35~DR04 | 0.0000 |
|  |  |  |  |  |  |  |  |  |  | A66~B41~DR15 | 0.0000 |
|  |  |  |  |  |  |  |  |  |  | A66~B07~DR15 | 0.0000 |
|  |  |  |  |  |  |  |  |  |  | A66~B07~DR04 | 0.0000 |
|  |  |  |  |  |  |  |  |  |  | A26~B27~DR04 | 0.0000 |
|  |  |  |  |  |  |  |  |  |  | A03~B27~DR13 | 0.0000 |
|  |  |  |  |  |  |  |  |  |  | A03~B44~DR13 | 0.0000 |
|  |  |  |  |  |  |  |  |  |  | A26~B18~DR14 | 0.0000 |
|  |  |  |  |  |  |  |  |  |  | A68~B18~DR04 | 0.0000 |
|  |  |  |  |  |  |  |  |  |  | A68~B27~DR14 | 0.0000 |
|  |  |  |  |  |  |  |  |  |  | A26~B35~DR13 | 0.0000 |
|  |  |  |  |  |  |  |  |  |  | A03~B58~DR07 | 0.0000 |
|  |  |  |  |  |  |  |  |  |  | A03~B37~DR07 | 0.0000 |
|  |  |  |  |  |  |  |  |  |  | A23~B13~DR07 | 0.0000 |
|  |  |  |  |  |  |  |  |  |  | A23~B13~DR04 | 0.0000 |
|  |  |  |  |  |  |  |  |  |  | A23~B58~DR07 | 0.0000 |
|  |  |  |  |  |  |  |  |  |  | A03~B41~DR07 | 0.0000 |
|  |  |  |  |  |  |  |  |  |  | A23~B58~DR04 | 0.0000 |
|  |  |  |  |  |  |  |  |  |  | A23~B37~DR07 | 0.0000 |
|  |  |  |  |  |  |  |  |  |  | A02~B37~DR13 | 0.0000 |
|  |  |  |  |  |  |  |  |  |  | A32~B37~DR15 | 0.0000 |
|  |  |  |  |  |  |  |  |  |  | A02~B37~DR15 | 0.0000 |
|  |  |  |  |  |  |  |  |  |  | A32~B07~DR10 | 0.0000 |
|  |  |  |  |  |  |  |  |  |  | A32~B07~DR15 | 0.0000 |
|  |  |  |  |  |  |  |  |  |  | A32~B07~DR07 | 0.0000 |
|  |  |  |  |  |  |  |  |  |  | A32~B13~DR15 | 0.0000 |
|  |  |  |  |  |  |  |  |  |  | A02~B13~DR10 | 0.0000 |
|  |  |  |  |  |  |  |  |  |  | A32~B27~DR13 | 0.0000 |
|  |  |  |  |  |  |  |  |  |  | A11~B27~DR07 | 0.0000 |
|  |  |  |  |  |  |  |  |  |  | A11~B27~DR13 | 0.0000 |
|  |  |  |  |  |  |  |  |  |  | A29~B58~DR11 | 0.0000 |
|  |  |  |  |  |  |  |  |  |  | A29~B41~DR16 | 0.0000 |
|  |  |  |  |  |  |  |  |  |  | A29~B41~DR11 | 0.0000 |
|  |  |  |  |  |  |  |  |  |  | A03~B38~DR03 | 0.0000 |
|  |  |  |  |  |  |  |  |  |  | A68~B38~DR13 | 0.0000 |
|  |  |  |  |  |  |  |  |  |  | A24~B53~DR11 | 0.0000 |
|  |  |  |  |  |  |  |  |  |  | A11~B53~DR07 | 0.0000 |
|  |  |  |  |  |  |  |  |  |  | A24~B55~DR07 | 0.0000 |
|  |  |  |  |  |  |  |  |  |  | A11~B53~DR11 | 0.0000 |
|  |  |  |  |  |  |  |  |  |  | A24~B49~DR03 | 0.0000 |
|  |  |  |  |  |  |  |  |  |  | A24~B41~DR03 | 0.0000 |
|  |  |  |  |  |  |  |  |  |  | A24~B41~DR04 | 0.0000 |
|  |  |  |  |  |  |  |  |  |  | A33~B14~DR04 | 0.0000 |
|  |  |  |  |  |  |  |  |  |  | A03~B14~DR04 | 0.0000 |
|  |  |  |  |  |  |  |  |  |  | A33~B44~DR01 | 0.0000 |
|  |  |  |  |  |  |  |  |  |  | A33~B44~DR04 | 0.0000 |
|  |  |  |  |  |  |  |  |  |  | A01~B39~DR04 | 0.0000 |
|  |  |  |  |  |  |  |  |  |  | A01~B39~DR16 | 0.0000 |
|  |  |  |  |  |  |  |  |  |  | A01~B50~DR04 | 0.0000 |
|  |  |  |  |  |  |  |  |  |  | A01~B50~DR03 | 0.0000 |
|  |  |  |  |  |  |  |  |  |  | A02~B50~DR01 | 0.0000 |
|  |  |  |  |  |  |  |  |  |  | A23~B07~DR03 | 0.0000 |
|  |  |  |  |  |  |  |  |  |  | A23~B07~DR01 | 0.0000 |
|  |  |  |  |  |  |  |  |  |  | A23~B50~DR01 | 0.0000 |
|  |  |  |  |  |  |  |  |  |  | A11~B13~DR11 | 0.0000 |
|  |  |  |  |  |  |  |  |  |  | A68~B13~DR11 | 0.0000 |
|  |  |  |  |  |  |  |  |  |  | A11~B07~DR13 | 0.0000 |
|  |  |  |  |  |  |  |  |  |  | A11~B58~DR11 | 0.0000 |
|  |  |  |  |  |  |  |  |  |  | A68~B13~DR15 | 0.0000 |
|  |  |  |  |  |  |  |  |  |  | A24~B58~DR11 | 0.0000 |
|  |  |  |  |  |  |  |  |  |  | A24~B58~DR15 | 0.0000 |
|  |  |  |  |  |  |  |  |  |  | A26~B07~DR04 | 0.0000 |
|  |  |  |  |  |  |  |  |  |  | A29~B38~DR10 | 0.0000 |
|  |  |  |  |  |  |  |  |  |  | A26~B07~DR10 | 0.0000 |
|  |  |  |  |  |  |  |  |  |  | A29~B51~DR11 | 0.0000 |
|  |  |  |  |  |  |  |  |  |  | A29~B51~DR10 | 0.0000 |
|  |  |  |  |  |  |  |  |  |  | A01~B39~DR01 | 0.0000 |
|  |  |  |  |  |  |  |  |  |  | A01~B08~DR01 | 0.0000 |
|  |  |  |  |  |  |  |  |  |  | A02~B56~DR03 | 0.0000 |
|  |  |  |  |  |  |  |  |  |  | A01~B56~DR03 | 0.0000 |
|  |  |  |  |  |  |  |  |  |  | A01~B56~DR11 | 0.0000 |
|  |  |  |  |  |  |  |  |  |  | A32~B51~DR01 | 0.0000 |
|  |  |  |  |  |  |  |  |  |  | A01~B51~DR03 | 0.0000 |
|  |  |  |  |  |  |  |  |  |  | A02~B18~DR08 | 0.0000 |
|  |  |  |  |  |  |  |  |  |  | A68~B35~DR10 | 0.0000 |
|  |  |  |  |  |  |  |  |  |  | A68~B51~DR08 | 0.0000 |
|  |  |  |  |  |  |  |  |  |  | A68~B51~DR10 | 0.0000 |
|  |  |  |  |  |  |  |  |  |  | A33~B44~DR16 | 0.0000 |
|  |  |  |  |  |  |  |  |  |  | A01~B44~DR16 | 0.0000 |
|  |  |  |  |  |  |  |  |  |  | A33~B35~DR07 | 0.0000 |
|  |  |  |  |  |  |  |  |  |  | A11~B52~DR16 | 0.0000 |
|  |  |  |  |  |  |  |  |  |  | A03~B52~DR16 | 0.0000 |
|  |  |  |  |  |  |  |  |  |  | A33~B38~DR14 | 0.0000 |
|  |  |  |  |  |  |  |  |  |  | A33~B35~DR14 | 0.0000 |
|  |  |  |  |  |  |  |  |  |  | A33~B52~DR16 | 0.0000 |
|  |  |  |  |  |  |  |  |  |  | A03~B08~DR08 | 0.0000 |
|  |  |  |  |  |  |  |  |  |  | A02~B08~DR08 | 0.0000 |
|  |  |  |  |  |  |  |  |  |  | A24~B13~DR11 | 0.0000 |
|  |  |  |  |  |  |  |  |  |  | A69~B49~DR07 | 0.0000 |
|  |  |  |  |  |  |  |  |  |  | A69~B40~DR07 | 0.0000 |
|  |  |  |  |  |  |  |  |  |  | A24~B49~DR07 | 0.0000 |
|  |  |  |  |  |  |  |  |  |  | A69~B40~DR13 | 0.0000 |
|  |  |  |  |  |  |  |  |  |  | A30~B18~DR01 | 0.0000 |
|  |  |  |  |  |  |  |  |  |  | A69~B49~DR04 | 0.0000 |
|  |  |  |  |  |  |  |  |  |  | A69~B35~DR13 | 0.0000 |
|  |  |  |  |  |  |  |  |  |  | A02~B49~DR15 | 0.0000 |
|  |  |  |  |  |  |  |  |  |  | A29~B13~DR13 | 0.0000 |
|  |  |  |  |  |  |  |  |  |  | A29~B35~DR13 | 0.0000 |
|  |  |  |  |  |  |  |  |  |  | A26~B46~DR08 | 0.0000 |
|  |  |  |  |  |  |  |  |  |  | A02~B38~DR08 | 0.0000 |
|  |  |  |  |  |  |  |  |  |  | A26~B46~DR14 | 0.0000 |
|  |  |  |  |  |  |  |  |  |  | A02~B46~DR14 | 0.0000 |
|  |  |  |  |  |  |  |  |  |  | A26~B38~DR08 | 0.0000 |
|  |  |  |  |  |  |  |  |  |  | A32~B18~DR01 | 0.0000 |
|  |  |  |  |  |  |  |  |  |  | A32~B35~DR01 | 0.0000 |
|  |  |  |  |  |  |  |  |  |  | A26~B07~DR13 | 0.0000 |
|  |  |  |  |  |  |  |  |  |  | A01~B08~DR10 | 0.0000 |
|  |  |  |  |  |  |  |  |  |  | A11~B37~DR10 | 0.0000 |
|  |  |  |  |  |  |  |  |  |  | A11~B37~DR14 | 0.0000 |
|  |  |  |  |  |  |  |  |  |  | A11~B41~DR11 | 0.0000 |
|  |  |  |  |  |  |  |  |  |  | A68~B52~DR03 | 0.0000 |
|  |  |  |  |  |  |  |  |  |  | A11~B08~DR15 | 0.0000 |
|  |  |  |  |  |  |  |  |  |  | A26~B52~DR04 | 0.0000 |
|  |  |  |  |  |  |  |  |  |  | A11~B38~DR15 | 0.0000 |
|  |  |  |  |  |  |  |  |  |  | A69~B38~DR04 | 0.0000 |
|  |  |  |  |  |  |  |  |  |  | A69~B38~DR11 | 0.0000 |
|  |  |  |  |  |  |  |  |  |  | A69~B51~DR04 | 0.0000 |
|  |  |  |  |  |  |  |  |  |  | A32~B44~DR01 | 0.0000 |
|  |  |  |  |  |  |  |  |  |  | A33~B39~DR03 | 0.0000 |
|  |  |  |  |  |  |  |  |  |  | A33~B39~DR16 | 0.0000 |
|  |  |  |  |  |  |  |  |  |  | A01~B15~DR15 | 0.0000 |
|  |  |  |  |  |  |  |  |  |  | A01~B07~DR13 | 0.0000 |
|  |  |  |  |  |  |  |  |  |  | A01~B51~DR10 | 0.0000 |
|  |  |  |  |  |  |  |  |  |  | A24~B37~DR11 | 0.0000 |
|  |  |  |  |  |  |  |  |  |  | A24~B51~DR10 | 0.0000 |
|  |  |  |  |  |  |  |  |  |  | A11~B08~DR09 | 0.0000 |
|  |  |  |  |  |  |  |  |  |  | A02~B52~DR09 | 0.0000 |
|  |  |  |  |  |  |  |  |  |  | A11~B08~DR04 | 0.0000 |
|  |  |  |  |  |  |  |  |  |  | A02~B08~DR04 | 0.0000 |
|  |  |  |  |  |  |  |  |  |  | A11~B52~DR09 | 0.0000 |
|  |  |  |  |  |  |  |  |  |  | A29~B08~DR03 | 0.0000 |
|  |  |  |  |  |  |  |  |  |  | A02~B18~DR09 | 0.0000 |
|  |  |  |  |  |  |  |  |  |  | A29~B52~DR03 | 0.0000 |
|  |  |  |  |  |  |  |  |  |  | A29~B08~DR04 | 0.0000 |
|  |  |  |  |  |  |  |  |  |  | A68~B57~DR07 | 0.0000 |
|  |  |  |  |  |  |  |  |  |  | A68~B57~DR13 | 0.0000 |
|  |  |  |  |  |  |  |  |  |  | A02~B57~DR14 | 0.0000 |
|  |  |  |  |  |  |  |  |  |  | A32~B57~DR14 | 0.0000 |
|  |  |  |  |  |  |  |  |  |  | A32~B27~DR16 | 0.0000 |
|  |  |  |  |  |  |  |  |  |  | A24~B57~DR16 | 0.0000 |
|  |  |  |  |  |  |  |  |  |  | A32~B57~DR07 | 0.0000 |
|  |  |  |  |  |  |  |  |  |  | A02~B49~DR11 | 0.0000 |
|  |  |  |  |  |  |  |  |  |  | A23~B18~DR14 | 0.0000 |
|  |  |  |  |  |  |  |  |  |  | A02~B49~DR14 | 0.0000 |
|  |  |  |  |  |  |  |  |  |  | A23~B18~DR11 | 0.0000 |
|  |  |  |  |  |  |  |  |  |  | A02~B27~DR14 | 0.0000 |
|  |  |  |  |  |  |  |  |  |  | A02~B27~DR07 | 0.0000 |
|  |  |  |  |  |  |  |  |  |  | A01~B27~DR10 | 0.0000 |
|  |  |  |  |  |  |  |  |  |  | A03~B44~DR10 | 0.0000 |
|  |  |  |  |  |  |  |  |  |  | A03~B27~DR07 | 0.0000 |
|  |  |  |  |  |  |  |  |  |  | A03~B44~DR03 | 0.0000 |
|  |  |  |  |  |  |  |  |  |  | A11~B08~DR07 | 0.0000 |
|  |  |  |  |  |  |  |  |  |  | A03~B08~DR07 | 0.0000 |
|  |  |  |  |  |  |  |  |  |  | A11~B44~DR03 | 0.0000 |
|  |  |  |  |  |  |  |  |  |  | A23~B55~DR11 | 0.0000 |
|  |  |  |  |  |  |  |  |  |  | A03~B55~DR07 | 0.0000 |
|  |  |  |  |  |  |  |  |  |  | A01~B38~DR15 | 0.0000 |
|  |  |  |  |  |  |  |  |  |  | A24~B08~DR14 | 0.0000 |
|  |  |  |  |  |  |  |  |  |  | A03~B51~DR10 | 0.0000 |
|  |  |  |  |  |  |  |  |  |  | A24~B07~DR08 | 0.0000 |
|  |  |  |  |  |  |  |  |  |  | A24~B38~DR10 | 0.0000 |
|  |  |  |  |  |  |  |  |  |  | A24~B08~DR11 | 0.0000 |
|  |  |  |  |  |  |  |  |  |  | A68~B49~DR11 | 0.0000 |
|  |  |  |  |  |  |  |  |  |  | A68~B49~DR14 | 0.0000 |
|  |  |  |  |  |  |  |  |  |  | A23~B55~DR14 | 0.0000 |
|  |  |  |  |  |  |  |  |  |  | A24~B49~DR14 | 0.0000 |
|  |  |  |  |  |  |  |  |  |  | A23~B55~DR13 | 0.0000 |
|  |  |  |  |  |  |  |  |  |  | A23~B49~DR12 | 0.0000 |
|  |  |  |  |  |  |  |  |  |  | A23~B38~DR12 | 0.0000 |
|  |  |  |  |  |  |  |  |  |  | A68~B49~DR12 | 0.0000 |
|  |  |  |  |  |  |  |  |  |  | A11~B13~DR04 | 0.0000 |
|  |  |  |  |  |  |  |  |  |  | A30~B07~DR15 | 0.0000 |
|  |  |  |  |  |  |  |  |  |  | A11~B13~DR15 | 0.0000 |
|  |  |  |  |  |  |  |  |  |  | A30~B07~DR04 | 0.0000 |
|  |  |  |  |  |  |  |  |  |  | A11~B07~DR04 | 0.0000 |
|  |  |  |  |  |  |  |  |  |  | A26~B40~DR07 | 0.0000 |
|  |  |  |  |  |  |  |  |  |  | A24~B51~DR16 | 0.0000 |
|  |  |  |  |  |  |  |  |  |  | A26~B57~DR13 | 0.0000 |
|  |  |  |  |  |  |  |  |  |  | A68~B57~DR11 | 0.0000 |
|  |  |  |  |  |  |  |  |  |  | A03~B37~DR11 | 0.0000 |
|  |  |  |  |  |  |  |  |  |  | A24~B50~DR14 | 0.0000 |
|  |  |  |  |  |  |  |  |  |  | A32~B50~DR11 | 0.0000 |
|  |  |  |  |  |  |  |  |  |  | A32~B41~DR04 | 0.0000 |
|  |  |  |  |  |  |  |  |  |  | A32~B41~DR11 | 0.0000 |
|  |  |  |  |  |  |  |  |  |  | A03~B18~DR15 | 0.0000 |
|  |  |  |  |  |  |  |  |  |  | A25~B18~DR01 | 0.0000 |
|  |  |  |  |  |  |  |  |  |  | A25~B51~DR15 | 0.0000 |
|  |  |  |  |  |  |  |  |  |  | A25~B35~DR01 | 0.0000 |
|  |  |  |  |  |  |  |  |  |  | A32~B51~DR09 | 0.0000 |
|  |  |  |  |  |  |  |  |  |  | A32~B35~DR09 | 0.0000 |
|  |  |  |  |  |  |  |  |  |  | A01~B55~DR11 | 0.0000 |
|  |  |  |  |  |  |  |  |  |  | A24~B08~DR13 | 0.0000 |
|  |  |  |  |  |  |  |  |  |  | A01~B08~DR15 | 0.0000 |
|  |  |  |  |  |  |  |  |  |  | A03~B44~DR15 | 0.0000 |
|  |  |  |  |  |  |  |  |  |  | A03~B27~DR15 | 0.0000 |
|  |  |  |  |  |  |  |  |  |  | A25~B18~DR12 | 0.0000 |
|  |  |  |  |  |  |  |  |  |  | A25~B40~DR12 | 0.0000 |
|  |  |  |  |  |  |  |  |  |  | A25~B40~DR15 | 0.0000 |
|  |  |  |  |  |  |  |  |  |  | A11~B40~DR15 | 0.0000 |
|  |  |  |  |  |  |  |  |  |  | A25~B27~DR12 | 0.0000 |
|  |  |  |  |  |  |  |  |  |  | A03~B18~DR12 | 0.0000 |
|  |  |  |  |  |  |  |  |  |  | A11~B07~DR12 | 0.0000 |
|  |  |  |  |  |  |  |  |  |  | A02~B07~DR12 | 0.0000 |
|  |  |  |  |  |  |  |  |  |  | A23~B07~DR12 | 0.0000 |
|  |  |  |  |  |  |  |  |  |  | A23~B35~DR12 | 0.0000 |
|  |  |  |  |  |  |  |  |  |  | A23~B35~DR15 | 0.0000 |
|  |  |  |  |  |  |  |  |  |  | A30~B37~DR08 | 0.0000 |
|  |  |  |  |  |  |  |  |  |  | A30~B07~DR08 | 0.0000 |
|  |  |  |  |  |  |  |  |  |  | A02~B37~DR08 | 0.0000 |
|  |  |  |  |  |  |  |  |  |  | A32~B07~DR16 | 0.0000 |
|  |  |  |  |  |  |  |  |  |  | A29~B35~DR16 | 0.0000 |
|  |  |  |  |  |  |  |  |  |  | A32~B07~DR04 | 0.0000 |
|  |  |  |  |  |  |  |  |  |  | A29~B07~DR16 | 0.0000 |
|  |  |  |  |  |  |  |  |  |  | A33~B37~DR10 | 0.0000 |
|  |  |  |  |  |  |  |  |  |  | A01~B18~DR10 | 0.0000 |
|  |  |  |  |  |  |  |  |  |  | A33~B37~DR11 | 0.0000 |
|  |  |  |  |  |  |  |  |  |  | A33~B18~DR10 | 0.0000 |
|  |  |  |  |  |  |  |  |  |  | A02~B37~DR11 | 0.0000 |
|  |  |  |  |  |  |  |  |  |  | A32~B44~DR10 | 0.0000 |
|  |  |  |  |  |  |  |  |  |  | A02~B38~DR10 | 0.0000 |
|  |  |  |  |  |  |  |  |  |  | A32~B37~DR13 | 0.0000 |
|  |  |  |  |  |  |  |  |  |  | A32~B38~DR10 | 0.0000 |
|  |  |  |  |  |  |  |  |  |  | A01~B13~DR13 | 0.0000 |
|  |  |  |  |  |  |  |  |  |  | A68~B49~DR01 | 0.0000 |
|  |  |  |  |  |  |  |  |  |  | A23~B35~DR01 | 0.0000 |
|  |  |  |  |  |  |  |  |  |  | A02~B40~DR01 | 0.0000 |
|  |  |  |  |  |  |  |  |  |  | A23~B40~DR09 | 0.0000 |
|  |  |  |  |  |  |  |  |  |  | A02~B49~DR09 | 0.0000 |
|  |  |  |  |  |  |  |  |  |  | A23~B40~DR01 | 0.0000 |
|  |  |  |  |  |  |  |  |  |  | A24~B07~DR09 | 0.0000 |
|  |  |  |  |  |  |  |  |  |  | A24~B40~DR09 | 0.0000 |
|  |  |  |  |  |  |  |  |  |  | A11~B54~DR15 | 0.0000 |
|  |  |  |  |  |  |  |  |  |  | A11~B54~DR04 | 0.0000 |
|  |  |  |  |  |  |  |  |  |  | A02~B54~DR15 | 0.0000 |
|  |  |  |  |  |  |  |  |  |  | A03~B54~DR15 | 0.0000 |
|  |  |  |  |  |  |  |  |  |  | A03~B54~DR04 | 0.0000 |
|  |  |  |  |  |  |  |  |  |  | A33~B55~DR15 | 0.0000 |
|  |  |  |  |  |  |  |  |  |  | A02~B55~DR13 | 0.0000 |
|  |  |  |  |  |  |  |  |  |  | A33~B40~DR15 | 0.0000 |
|  |  |  |  |  |  |  |  |  |  | A03~B40~DR13 | 0.0000 |
|  |  |  |  |  |  |  |  |  |  | A33~B49~DR13 | 0.0000 |
|  |  |  |  |  |  |  |  |  |  | A03~B49~DR13 | 0.0000 |
|  |  |  |  |  |  |  |  |  |  | A33~B40~DR04 | 0.0000 |
|  |  |  |  |  |  |  |  |  |  | A33~B58~DR04 | 0.0000 |
|  |  |  |  |  |  |  |  |  |  | A03~B55~DR15 | 0.0000 |
|  |  |  |  |  |  |  |  |  |  | A32~B18~DR04 | 0.0000 |
|  |  |  |  |  |  |  |  |  |  | A32~B14~DR01 | 0.0000 |
|  |  |  |  |  |  |  |  |  |  | A32~B14~DR11 | 0.0000 |
|  |  |  |  |  |  |  |  |  |  | A29~B14~DR11 | 0.0000 |
|  |  |  |  |  |  |  |  |  |  | A32~B27~DR01 | 0.0000 |
|  |  |  |  |  |  |  |  |  |  | A32~B15~DR04 | 0.0000 |
|  |  |  |  |  |  |  |  |  |  | A68~B07~DR14 | 0.0000 |
|  |  |  |  |  |  |  |  |  |  | A24~B07~DR14 | 0.0000 |
|  |  |  |  |  |  |  |  |  |  | A32~B07~DR08 | 0.0000 |
|  |  |  |  |  |  |  |  |  |  | A32~B27~DR08 | 0.0000 |
|  |  |  |  |  |  |  |  |  |  | A68~B15~DR08 | 0.0000 |
|  |  |  |  |  |  |  |  |  |  | A68~B15~DR01 | 0.0000 |
|  |  |  |  |  |  |  |  |  |  | A03~B13~DR08 | 0.0000 |
|  |  |  |  |  |  |  |  |  |  | A02~B13~DR08 | 0.0000 |
|  |  |  |  |  |  |  |  |  |  | A26~B49~DR13 | 0.0000 |
|  |  |  |  |  |  |  |  |  |  | A26~B49~DR11 | 0.0000 |
|  |  |  |  |  |  |  |  |  |  | A24~B40~DR01 | 0.0000 |
|  |  |  |  |  |  |  |  |  |  | A68~B15~DR11 | 0.0000 |
|  |  |  |  |  |  |  |  |  |  | A03~B40~DR11 | 0.0000 |
|  |  |  |  |  |  |  |  |  |  | A68~B07~DR07 | 0.0000 |
|  |  |  |  |  |  |  |  |  |  | A68~B40~DR07 | 0.0000 |
|  |  |  |  |  |  |  |  |  |  | A11~B41~DR07 | 0.0000 |
|  |  |  |  |  |  |  |  |  |  | A11~B07~DR03 | 0.0000 |
|  |  |  |  |  |  |  |  |  |  | A11~B07~DR07 | 0.0000 |
|  |  |  |  |  |  |  |  |  |  | A03~B15~DR07 | 0.0000 |
|  |  |  |  |  |  |  |  |  |  | A03~B15~DR01 | 0.0000 |
|  |  |  |  |  |  |  |  |  |  | A24~B15~DR07 | 0.0000 |
|  |  |  |  |  |  |  |  |  |  | A68~B50~DR04 | 0.0000 |
|  |  |  |  |  |  |  |  |  |  | A68~B50~DR11 | 0.0000 |
|  |  |  |  |  |  |  |  |  |  | A24~B50~DR04 | 0.0000 |
|  |  |  |  |  |  |  |  |  |  | A32~B18~DR10 | 0.0000 |
|  |  |  |  |  |  |  |  |  |  | A24~B15~DR10 | 0.0000 |
|  |  |  |  |  |  |  |  |  |  | A24~B18~DR10 | 0.0000 |
|  |  |  |  |  |  |  |  |  |  | A68~B55~DR03 | 0.0000 |
|  |  |  |  |  |  |  |  |  |  | A03~B55~DR03 | 0.0000 |
|  |  |  |  |  |  |  |  |  |  | A03~B18~DR03 | 0.0000 |
|  |  |  |  |  |  |  |  |  |  | A32~B55~DR10 | 0.0000 |
|  |  |  |  |  |  |  |  |  |  | A03~B55~DR10 | 0.0000 |
|  |  |  |  |  |  |  |  |  |  | A32~B18~DR03 | 0.0000 |
|  |  |  |  |  |  |  |  |  |  | A32~B51~DR13 | 0.0000 |
|  |  |  |  |  |  |  |  |  |  | A32~B40~DR13 | 0.0000 |
|  |  |  |  |  |  |  |  |  |  | A32~B40~DR11 | 0.0000 |
|  |  |  |  |  |  |  |  |  |  | A03~B08~DR04 | 0.0000 |
|  |  |  |  |  |  |  |  |  |  | A23~B13~DR11 | 0.0000 |
|  |  |  |  |  |  |  |  |  |  | A02~B14~DR13 | 0.0000 |
|  |  |  |  |  |  |  |  |  |  | A02~B58~DR07 | 0.0000 |
|  |  |  |  |  |  |  |  |  |  | A03~B14~DR03 | 0.0000 |
|  |  |  |  |  |  |  |  |  |  | A33~B08~DR13 | 0.0000 |
|  |  |  |  |  |  |  |  |  |  | A02~B50~DR13 | 0.0000 |
|  |  |  |  |  |  |  |  |  |  | A29~B37~DR13 | 0.0000 |
|  |  |  |  |  |  |  |  |  |  | A68~B51~DR01 | 0.0000 |
|  |  |  |  |  |  |  |  |  |  | A33~B15~DR13 | 0.0000 |
|  |  |  |  |  |  |  |  |  |  | A24~B18~DR09 | 0.0000 |
|  |  |  |  |  |  |  |  |  |  | A23~B18~DR10 | 0.0000 |
|  |  |  |  |  |  |  |  |  |  | A30~B44~DR10 | 0.0000 |
|  |  |  |  |  |  |  |  |  |  | A23~B18~DR07 | 0.0000 |
|  |  |  |  |  |  |  |  |  |  | A30~B18~DR07 | 0.0000 |
|  |  |  |  |  |  |  |  |  |  | A23~B44~DR10 | 0.0000 |
|  |  |  |  |  |  |  |  |  |  | A30~B13~DR10 | 0.0000 |
|  |  |  |  |  |  |  |  |  |  | A24~B13~DR10 | 0.0000 |
|  |  |  |  |  |  |  |  |  |  | A32~B38~DR04 | 0.0000 |
|  |  |  |  |  |  |  |  |  |  | A01~B13~DR11 | 0.0000 |
|  |  |  |  |  |  |  |  |  |  | A30~B35~DR13 | 0.0000 |
|  |  |  |  |  |  |  |  |  |  | A02~B39~DR15 | 0.0000 |
|  |  |  |  |  |  |  |  |  |  | A32~B39~DR11 | 0.0000 |
|  |  |  |  |  |  |  |  |  |  | A32~B51~DR07 | 0.0000 |
|  |  |  |  |  |  |  |  |  |  | A32~B39~DR16 | 0.0000 |
|  |  |  |  |  |  |  |  |  |  | A24~B39~DR07 | 0.0000 |
|  |  |  |  |  |  |  |  |  |  | A32~B55~DR16 | 0.0000 |
|  |  |  |  |  |  |  |  |  |  | A32~B55~DR07 | 0.0000 |
|  |  |  |  |  |  |  |  |  |  | A01~B14~DR07 | 0.0000 |
|  |  |  |  |  |  |  |  |  |  | A01~B07~DR14 | 0.0000 |
|  |  |  |  |  |  |  |  |  |  | A01~B07~DR10 | 0.0000 |
|  |  |  |  |  |  |  |  |  |  | A24~B50~DR16 | 0.0000 |
|  |  |  |  |  |  |  |  |  |  | A24~B51~DR01 | 0.0000 |
|  |  |  |  |  |  |  |  |  |  | A11~B41~DR01 | 0.0000 |
|  |  |  |  |  |  |  |  |  |  | A11~B41~DR04 | 0.0000 |
|  |  |  |  |  |  |  |  |  |  | A11~B37~DR01 | 0.0000 |
|  |  |  |  |  |  |  |  |  |  | A29~B18~DR16 | 0.0000 |
|  |  |  |  |  |  |  |  |  |  | A29~B44~DR16 | 0.0000 |
|  |  |  |  |  |  |  |  |  |  | A33~B44~DR12 | 0.0000 |
|  |  |  |  |  |  |  |  |  |  | A02~B14~DR12 | 0.0000 |
|  |  |  |  |  |  |  |  |  |  | A33~B15~DR01 | 0.0000 |
|  |  |  |  |  |  |  |  |  |  | A69~B14~DR01 | 0.0000 |
|  |  |  |  |  |  |  |  |  |  | A69~B44~DR01 | 0.0000 |
|  |  |  |  |  |  |  |  |  |  | A01~B15~DR01 | 0.0000 |
|  |  |  |  |  |  |  |  |  |  | A69~B44~DR04 | 0.0000 |
|  |  |  |  |  |  |  |  |  |  | A01~B44~DR01 | 0.0000 |
|  |  |  |  |  |  |  |  |  |  | A69~B15~DR04 | 0.0000 |
|  |  |  |  |  |  |  |  |  |  | A69~B44~DR12 | 0.0000 |
|  |  |  |  |  |  |  |  |  |  | A02~B15~DR12 | 0.0000 |
|  |  |  |  |  |  |  |  |  |  | A69~B15~DR12 | 0.0000 |
|  |  |  |  |  |  |  |  |  |  | A26~B58~DR16 | 0.0000 |
|  |  |  |  |  |  |  |  |  |  | A03~B08~DR11 | 0.0000 |
|  |  |  |  |  |  |  |  |  |  | A68~B39~DR11 | 0.0000 |
|  |  |  |  |  |  |  |  |  |  | A31~B08~DR03 | 0.0000 |
|  |  |  |  |  |  |  |  |  |  | A01~B49~DR03 | 0.0000 |
|  |  |  |  |  |  |  |  |  |  | A31~B08~DR01 | 0.0000 |
|  |  |  |  |  |  |  |  |  |  | A31~B49~DR03 | 0.0000 |
|  |  |  |  |  |  |  |  |  |  | A32~B39~DR09 | 0.0000 |
|  |  |  |  |  |  |  |  |  |  | A32~B27~DR09 | 0.0000 |
|  |  |  |  |  |  |  |  |  |  | A26~B47~DR01 | 0.0000 |
|  |  |  |  |  |  |  |  |  |  | A02~B47~DR01 | 0.0000 |
|  |  |  |  |  |  |  |  |  |  | A26~B41~DR15 | 0.0000 |
|  |  |  |  |  |  |  |  |  |  | A02~B47~DR15 | 0.0000 |
|  |  |  |  |  |  |  |  |  |  | A26~B41~DR01 | 0.0000 |
|  |  |  |  |  |  |  |  |  |  | A24~B18~DR08 | 0.0000 |
|  |  |  |  |  |  |  |  |  |  | A24~B41~DR15 | 0.0000 |
|  |  |  |  |  |  |  |  |  |  | A24~B41~DR01 | 0.0000 |
|  |  |  |  |  |  |  |  |  |  | A26~B47~DR08 | 0.0000 |
|  |  |  |  |  |  |  |  |  |  | A26~B15~DR08 | 0.0000 |
|  |  |  |  |  |  |  |  |  |  | A02~B47~DR08 | 0.0000 |
|  |  |  |  |  |  |  |  |  |  | A24~B47~DR15 | 0.0000 |
|  |  |  |  |  |  |  |  |  |  | A26~B18~DR15 | 0.0000 |
|  |  |  |  |  |  |  |  |  |  | A03~B52~DR13 | 0.0000 |
|  |  |  |  |  |  |  |  |  |  | A31~B14~DR15 | 0.0000 |
|  |  |  |  |  |  |  |  |  |  | A31~B52~DR13 | 0.0000 |
|  |  |  |  |  |  |  |  |  |  | A26~B50~DR01 | 0.0000 |
|  |  |  |  |  |  |  |  |  |  | A23~B18~DR16 | 0.0000 |
|  |  |  |  |  |  |  |  |  |  | A29~B51~DR16 | 0.0000 |
|  |  |  |  |  |  |  |  |  |  | A23~B51~DR11 | 0.0000 |
|  |  |  |  |  |  |  |  |  |  | A11~B49~DR04 | 0.0000 |
|  |  |  |  |  |  |  |  |  |  | A23~B08~DR03 | 0.0000 |
|  |  |  |  |  |  |  |  |  |  | A29~B14~DR13 | 0.0000 |
|  |  |  |  |  |  |  |  |  |  | A24~B14~DR13 | 0.0000 |
|  |  |  |  |  |  |  |  |  |  | A24~B14~DR04 | 0.0000 |
|  |  |  |  |  |  |  |  |  |  | A29~B55~DR13 | 0.0000 |
|  |  |  |  |  |  |  |  |  |  | A29~B14~DR07 | 0.0000 |
|  |  |  |  |  |  |  |  |  |  | A31~B18~DR07 | 0.0000 |
|  |  |  |  |  |  |  |  |  |  | A31~B18~DR14 | 0.0000 |
|  |  |  |  |  |  |  |  |  |  | A31~B27~DR07 | 0.0000 |
|  |  |  |  |  |  |  |  |  |  | A33~B50~DR16 | 0.0000 |
|  |  |  |  |  |  |  |  |  |  | A23~B35~DR16 | 0.0000 |
|  |  |  |  |  |  |  |  |  |  | A33~B50~DR04 | 0.0000 |
|  |  |  |  |  |  |  |  |  |  | A33~B35~DR04 | 0.0000 |
|  |  |  |  |  |  |  |  |  |  | A03~B50~DR11 | 0.0000 |
|  |  |  |  |  |  |  |  |  |  | A23~B07~DR16 | 0.0000 |
|  |  |  |  |  |  |  |  |  |  | A03~B50~DR16 | 0.0000 |
|  |  |  |  |  |  |  |  |  |  | A23~B07~DR11 | 0.0000 |
|  |  |  |  |  |  |  |  |  |  | A26~B58~DR11 | 0.0000 |
|  |  |  |  |  |  |  |  |  |  | A26~B37~DR13 | 0.0000 |
|  |  |  |  |  |  |  |  |  |  | A26~B37~DR11 | 0.0000 |
|  |  |  |  |  |  |  |  |  |  | A33~B49~DR14 | 0.0000 |
|  |  |  |  |  |  |  |  |  |  | A01~B52~DR11 | 0.0000 |
|  |  |  |  |  |  |  |  |  |  | A33~B08~DR08 | 0.0000 |
|  |  |  |  |  |  |  |  |  |  | A01~B52~DR08 | 0.0000 |
|  |  |  |  |  |  |  |  |  |  | A33~B08~DR11 | 0.0000 |
|  |  |  |  |  |  |  |  |  |  | A33~B52~DR11 | 0.0000 |
|  |  |  |  |  |  |  |  |  |  | A03~B57~DR03 | 0.0000 |
|  |  |  |  |  |  |  |  |  |  | A26~B57~DR03 | 0.0000 |
|  |  |  |  |  |  |  |  |  |  | A26~B57~DR07 | 0.0000 |
|  |  |  |  |  |  |  |  |  |  | A29~B45~DR15 | 0.0000 |
|  |  |  |  |  |  |  |  |  |  | A29~B52~DR15 | 0.0000 |
|  |  |  |  |  |  |  |  |  |  | A24~B57~DR03 | 0.0000 |
|  |  |  |  |  |  |  |  |  |  | A23~B27~DR04 | 0.0000 |
|  |  |  |  |  |  |  |  |  |  | A23~B39~DR03 | 0.0000 |
|  |  |  |  |  |  |  |  |  |  | A31~B41~DR04 | 0.0000 |
|  |  |  |  |  |  |  |  |  |  | A23~B27~DR07 | 0.0000 |
|  |  |  |  |  |  |  |  |  |  | A31~B41~DR07 | 0.0000 |
|  |  |  |  |  |  |  |  |  |  | A23~B41~DR15 | 0.0000 |
|  |  |  |  |  |  |  |  |  |  | A31~B50~DR15 | 0.0000 |
|  |  |  |  |  |  |  |  |  |  | A24~B27~DR15 | 0.0000 |
|  |  |  |  |  |  |  |  |  |  | A31~B50~DR04 | 0.0000 |
|  |  |  |  |  |  |  |  |  |  | A31~B27~DR15 | 0.0000 |
|  |  |  |  |  |  |  |  |  |  | A24~B27~DR03 | 0.0000 |
|  |  |  |  |  |  |  |  |  |  | A24~B52~DR11 | 0.0000 |
|  |  |  |  |  |  |  |  |  |  | A68~B52~DR11 | 0.0000 |
|  |  |  |  |  |  |  |  |  |  | A26~B08~DR15 | 0.0000 |
|  |  |  |  |  |  |  |  |  |  | A02~B08~DR15 | 0.0000 |
|  |  |  |  |  |  |  |  |  |  | A02~B08~DR10 | 0.0000 |
|  |  |  |  |  |  |  |  |  |  | A68~B37~DR10 | 0.0000 |
|  |  |  |  |  |  |  |  |  |  | A68~B37~DR07 | 0.0000 |
|  |  |  |  |  |  |  |  |  |  | A24~B37~DR07 | 0.0000 |
|  |  |  |  |  |  |  |  |  |  | A11~B54~DR07 | 0.0000 |
|  |  |  |  |  |  |  |  |  |  | A68~B54~DR13 | 0.0000 |
|  |  |  |  |  |  |  |  |  |  | A68~B54~DR07 | 0.0000 |
|  |  |  |  |  |  |  |  |  |  | A02~B54~DR13 | 0.0000 |
|  |  |  |  |  |  |  |  |  |  | A02~B54~DR07 | 0.0000 |
|  |  |  |  |  |  |  |  |  |  | A11~B50~DR13 | 0.0000 |
|  |  |  |  |  |  |  |  |  |  | A03~B58~DR11 | 0.0000 |
|  |  |  |  |  |  |  |  |  |  | A03~B51~DR14 | 0.0000 |
|  |  |  |  |  |  |  |  |  |  | A02~B41~DR10 | 0.0000 |
|  |  |  |  |  |  |  |  |  |  | A02~B40~DR10 | 0.0000 |
|  |  |  |  |  |  |  |  |  |  | A24~B41~DR11 | 0.0000 |
|  |  |  |  |  |  |  |  |  |  | A29~B48~DR04 | 0.0000 |
|  |  |  |  |  |  |  |  |  |  | A29~B48~DR14 | 0.0000 |
|  |  |  |  |  |  |  |  |  |  | A03~B13~DR04 | 0.0000 |
|  |  |  |  |  |  |  |  |  |  | A02~B44~DR14 | 0.0000 |
|  |  |  |  |  |  |  |  |  |  | A03~B48~DR04 | 0.0000 |
|  |  |  |  |  |  |  |  |  |  | A03~B48~DR14 | 0.0000 |
|  |  |  |  |  |  |  |  |  |  | A11~B08~DR11 | 0.0000 |
|  |  |  |  |  |  |  |  |  |  | A23~B14~DR11 | 0.0000 |
|  |  |  |  |  |  |  |  |  |  | A11~B14~DR11 | 0.0000 |
|  |  |  |  |  |  |  |  |  |  | A02~B48~DR11 | 0.0000 |
|  |  |  |  |  |  |  |  |  |  | A24~B07~DR12 | 0.0000 |
|  |  |  |  |  |  |  |  |  |  | A03~B35~DR12 | 0.0000 |
|  |  |  |  |  |  |  |  |  |  | A26~B13~DR01 | 0.0000 |
|  |  |  |  |  |  |  |  |  |  | A26~B35~DR12 | 0.0000 |
|  |  |  |  |  |  |  |  |  |  | A03~B13~DR12 | 0.0000 |
|  |  |  |  |  |  |  |  |  |  | A24~B07~DR16 | 0.0000 |
|  |  |  |  |  |  |  |  |  |  | A33~B14~DR16 | 0.0000 |
|  |  |  |  |  |  |  |  |  |  | A02~B14~DR16 | 0.0000 |
|  |  |  |  |  |  |  |  |  |  | A23~B27~DR01 | 0.0000 |
|  |  |  |  |  |  |  |  |  |  | A33~B27~DR10 | 0.0000 |
|  |  |  |  |  |  |  |  |  |  | A33~B27~DR01 | 0.0000 |
|  |  |  |  |  |  |  |  |  |  | A11~B39~DR13 | 0.0000 |
|  |  |  |  |  |  |  |  |  |  | A24~B27~DR01 | 0.0000 |
|  |  |  |  |  |  |  |  |  |  | A03~B13~DR13 | 0.0000 |
|  |  |  |  |  |  |  |  |  |  | A02~B13~DR16 | 0.0000 |
|  |  |  |  |  |  |  |  |  |  | A01~B41~DR11 | 0.0000 |
|  |  |  |  |  |  |  |  |  |  | A30~B27~DR04 | 0.0000 |
|  |  |  |  |  |  |  |  |  |  | A30~B27~DR11 | 0.0000 |
|  |  |  |  |  |  |  |  |  |  | A31~B40~DR01 | 0.0000 |
|  |  |  |  |  |  |  |  |  |  | A11~B40~DR01 | 0.0000 |
|  |  |  |  |  |  |  |  |  |  | A31~B27~DR01 | 0.0000 |
|  |  |  |  |  |  |  |  |  |  | A29~B50~DR07 | 0.0000 |
|  |  |  |  |  |  |  |  |  |  | A31~B07~DR10 | 0.0000 |
|  |  |  |  |  |  |  |  |  |  | A29~B50~DR10 | 0.0000 |
|  |  |  |  |  |  |  |  |  |  | A31~B07~DR07 | 0.0000 |
|  |  |  |  |  |  |  |  |  |  | A31~B50~DR10 | 0.0000 |
|  |  |  |  |  |  |  |  |  |  | A29~B41~DR03 | 0.0000 |
|  |  |  |  |  |  |  |  |  |  | A29~B41~DR10 | 0.0000 |
|  |  |  |  |  |  |  |  |  |  | A31~B41~DR03 | 0.0000 |
|  |  |  |  |  |  |  |  |  |  | A31~B50~DR03 | 0.0000 |
|  |  |  |  |  |  |  |  |  |  | A01~B44~DR10 | 0.0000 |
|  |  |  |  |  |  |  |  |  |  | A11~B37~DR15 | 0.0000 |
|  |  |  |  |  |  |  |  |  |  | A11~B44~DR10 | 0.0000 |
|  |  |  |  |  |  |  |  |  |  | A26~B39~DR03 | 0.0000 |
|  |  |  |  |  |  |  |  |  |  | A29~B51~DR15 | 0.0000 |
|  |  |  |  |  |  |  |  |  |  | A29~B51~DR04 | 0.0000 |
|  |  |  |  |  |  |  |  |  |  | A26~B15~DR04 | 0.0000 |
|  |  |  |  |  |  |  |  |  |  | A26~B44~DR10 | 0.0000 |
|  |  |  |  |  |  |  |  |  |  | A32~B08~DR10 | 0.0000 |
|  |  |  |  |  |  |  |  |  |  | A32~B08~DR15 | 0.0000 |
|  |  |  |  |  |  |  |  |  |  | A26~B44~DR03 | 0.0000 |
|  |  |  |  |  |  |  |  |  |  | A11~B50~DR15 | 0.0000 |
|  |  |  |  |  |  |  |  |  |  | A03~B41~DR15 | 0.0000 |
|  |  |  |  |  |  |  |  |  |  | A66~B07~DR13 | 0.0000 |
|  |  |  |  |  |  |  |  |  |  | A33~B08~DR01 | 0.0000 |
|  |  |  |  |  |  |  |  |  |  | A11~B14~DR16 | 0.0000 |
|  |  |  |  |  |  |  |  |  |  | A11~B08~DR16 | 0.0000 |
|  |  |  |  |  |  |  |  |  |  | A02~B08~DR16 | 0.0000 |
|  |  |  |  |  |  |  |  |  |  | A01~B08~DR04 | 0.0000 |
|  |  |  |  |  |  |  |  |  |  | A11~B51~DR16 | 0.0000 |
|  |  |  |  |  |  |  |  |  |  | A11~B49~DR16 | 0.0000 |
|  |  |  |  |  |  |  |  |  |  | A23~B51~DR09 | 0.0000 |
|  |  |  |  |  |  |  |  |  |  | A11~B49~DR09 | 0.0000 |
|  |  |  |  |  |  |  |  |  |  | A11~B55~DR09 | 0.0000 |
|  |  |  |  |  |  |  |  |  |  | A02~B55~DR09 | 0.0000 |
|  |  |  |  |  |  |  |  |  |  | A02~B38~DR07 | 0.0000 |
|  |  |  |  |  |  |  |  |  |  | A32~B38~DR07 | 0.0000 |
|  |  |  |  |  |  |  |  |  |  | A24~B38~DR16 | 0.0000 |
|  |  |  |  |  |  |  |  |  |  | A01~B38~DR16 | 0.0000 |
|  |  |  |  |  |  |  |  |  |  | A24~B44~DR07 | 0.0000 |
|  |  |  |  |  |  |  |  |  |  | A24~B38~DR07 | 0.0000 |
|  |  |  |  |  |  |  |  |  |  | A01~B38~DR07 | 0.0000 |
|  |  |  |  |  |  |  |  |  |  | A01~B38~DR10 | 0.0000 |
|  |  |  |  |  |  |  |  |  |  | A26~B50~DR14 | 0.0000 |
|  |  |  |  |  |  |  |  |  |  | A01~B50~DR14 | 0.0000 |
|  |  |  |  |  |  |  |  |  |  | A26~B40~DR14 | 0.0000 |
|  |  |  |  |  |  |  |  |  |  | A03~B15~DR10 | 0.0000 |
|  |  |  |  |  |  |  |  |  |  | A24~B14~DR11 | 0.0000 |
|  |  |  |  |  |  |  |  |  |  | A24~B14~DR07 | 0.0000 |
|  |  |  |  |  |  |  |  |  |  | A03~B14~DR07 | 0.0000 |
|  |  |  |  |  |  |  |  |  |  | A26~B48~DR11 | 0.0000 |
|  |  |  |  |  |  |  |  |  |  | A01~B48~DR11 | 0.0000 |
|  |  |  |  |  |  |  |  |  |  | A26~B14~DR04 | 0.0000 |
|  |  |  |  |  |  |  |  |  |  | A33~B48~DR01 | 0.0000 |
|  |  |  |  |  |  |  |  |  |  | A26~B14~DR01 | 0.0000 |
|  |  |  |  |  |  |  |  |  |  | A33~B48~DR04 | 0.0000 |
|  |  |  |  |  |  |  |  |  |  | A03~B41~DR16 | 0.0000 |
|  |  |  |  |  |  |  |  |  |  | A24~B08~DR04 | 0.0000 |
|  |  |  |  |  |  |  |  |  |  | A36~B18~DR04 | 0.0000 |
|  |  |  |  |  |  |  |  |  |  | A36~B51~DR08 | 0.0000 |
|  |  |  |  |  |  |  |  |  |  | A36~B51~DR04 | 0.0000 |
|  |  |  |  |  |  |  |  |  |  | A29~B18~DR08 | 0.0000 |
|  |  |  |  |  |  |  |  |  |  | A11~B58~DR08 | 0.0000 |
|  |  |  |  |  |  |  |  |  |  | A29~B51~DR08 | 0.0000 |
|  |  |  |  |  |  |  |  |  |  | A11~B58~DR04 | 0.0000 |
|  |  |  |  |  |  |  |  |  |  | A11~B51~DR08 | 0.0000 |
|  |  |  |  |  |  |  |  |  |  | A24~B39~DR03 | 0.0000 |
|  |  |  |  |  |  |  |  |  |  | A24~B39~DR01 | 0.0000 |
|  |  |  |  |  |  |  |  |  |  | A30~B53~DR16 | 0.0000 |
|  |  |  |  |  |  |  |  |  |  | A30~B55~DR13 | 0.0000 |
|  |  |  |  |  |  |  |  |  |  | A32~B53~DR16 | 0.0000 |
|  |  |  |  |  |  |  |  |  |  | A31~B13~DR07 | 0.0000 |
|  |  |  |  |  |  |  |  |  |  | A24~B57~DR14 | 0.0000 |
|  |  |  |  |  |  |  |  |  |  | A32~B49~DR03 | 0.0000 |
|  |  |  |  |  |  |  |  |  |  | A30~B49~DR03 | 0.0000 |
|  |  |  |  |  |  |  |  |  |  | A32~B44~DR03 | 0.0000 |
|  |  |  |  |  |  |  |  |  |  | A32~B44~DR15 | 0.0000 |
|  |  |  |  |  |  |  |  |  |  | A68~B44~DR10 | 0.0000 |
|  |  |  |  |  |  |  |  |  |  | A02~B15~DR10 | 0.0000 |
|  |  |  |  |  |  |  |  |  |  | A68~B15~DR03 | 0.0000 |
|  |  |  |  |  |  |  |  |  |  | A32~B15~DR03 | 0.0000 |
|  |  |  |  |  |  |  |  |  |  | A68~B15~DR13 | 0.0000 |
|  |  |  |  |  |  |  |  |  |  | A30~B15~DR10 | 0.0000 |
|  |  |  |  |  |  |  |  |  |  | A68~B49~DR10 | 0.0000 |
|  |  |  |  |  |  |  |  |  |  | A11~B50~DR04 | 0.0000 |
|  |  |  |  |  |  |  |  |  |  | A11~B49~DR08 | 0.0000 |
|  |  |  |  |  |  |  |  |  |  | A24~B49~DR08 | 0.0000 |
|  |  |  |  |  |  |  |  |  |  | A01~B58~DR15 | 0.0000 |
|  |  |  |  |  |  |  |  |  |  | A33~B52~DR13 | 0.0000 |
|  |  |  |  |  |  |  |  |  |  | A33~B51~DR14 | 0.0000 |
|  |  |  |  |  |  |  |  |  |  | A33~B51~DR13 | 0.0000 |
|  |  |  |  |  |  |  |  |  |  | A01~B51~DR14 | 0.0000 |
|  |  |  |  |  |  |  |  |  |  | A02~B08~DR14 | 0.0000 |
|  |  |  |  |  |  |  |  |  |  | A23~B18~DR04 | 0.0000 |
|  |  |  |  |  |  |  |  |  |  | A25~B35~DR11 | 0.0000 |
|  |  |  |  |  |  |  |  |  |  | A23~B15~DR04 | 0.0000 |
|  |  |  |  |  |  |  |  |  |  | A01~B13~DR03 | 0.0000 |
|  |  |  |  |  |  |  |  |  |  | A30~B49~DR16 | 0.0000 |
|  |  |  |  |  |  |  |  |  |  | A02~B49~DR16 | 0.0000 |
|  |  |  |  |  |  |  |  |  |  | A30~B49~DR14 | 0.0000 |
|  |  |  |  |  |  |  |  |  |  | A03~B49~DR14 | 0.0000 |
|  |  |  |  |  |  |  |  |  |  | A26~B55~DR01 | 0.0000 |
|  |  |  |  |  |  |  |  |  |  | A01~B40~DR08 | 0.0000 |
|  |  |  |  |  |  |  |  |  |  | A23~B41~DR11 | 0.0000 |
|  |  |  |  |  |  |  |  |  |  | A23~B40~DR08 | 0.0000 |
|  |  |  |  |  |  |  |  |  |  | A11~B49~DR13 | 0.0000 |
|  |  |  |  |  |  |  |  |  |  | A29~B49~DR11 | 0.0000 |
|  |  |  |  |  |  |  |  |  |  | A29~B49~DR15 | 0.0000 |
|  |  |  |  |  |  |  |  |  |  | A29~B41~DR04 | 0.0000 |
|  |  |  |  |  |  |  |  |  |  | A11~B41~DR15 | 0.0000 |
|  |  |  |  |  |  |  |  |  |  | A26~B50~DR13 | 0.0000 |
|  |  |  |  |  |  |  |  |  |  | A68~B51~DR12 | 0.0000 |
|  |  |  |  |  |  |  |  |  |  | A68~B35~DR12 | 0.0000 |
|  |  |  |  |  |  |  |  |  |  | A02~B55~DR03 | 0.0000 |
|  |  |  |  |  |  |  |  |  |  | A26~B55~DR10 | 0.0000 |
|  |  |  |  |  |  |  |  |  |  | A11~B52~DR01 | 0.0000 |
|  |  |  |  |  |  |  |  |  |  | A11~B14~DR15 | 0.0000 |
|  |  |  |  |  |  |  |  |  |  | A33~B52~DR01 | 0.0000 |
|  |  |  |  |  |  |  |  |  |  | A01~B14~DR01 | 0.0000 |
|  |  |  |  |  |  |  |  |  |  | A01~B14~DR03 | 0.0000 |
|  |  |  |  |  |  |  |  |  |  | A31~B15~DR01 | 0.0000 |
|  |  |  |  |  |  |  |  |  |  | A33~B14~DR14 | 0.0000 |
|  |  |  |  |  |  |  |  |  |  | A11~B39~DR07 | 0.0000 |
|  |  |  |  |  |  |  |  |  |  | A24~B52~DR07 | 0.0000 |
|  |  |  |  |  |  |  |  |  |  | A11~B39~DR11 | 0.0000 |
|  |  |  |  |  |  |  |  |  |  | A24~B39~DR15 | 0.0000 |
|  |  |  |  |  |  |  |  |  |  | A33~B44~DR14 | 0.0000 |
|  |  |  |  |  |  |  |  |  |  | A11~B44~DR13 | 0.0000 |
|  |  |  |  |  |  |  |  |  |  | A66~B35~DR11 | 0.0000 |
|  |  |  |  |  |  |  |  |  |  | A66~B14~DR13 | 0.0000 |
|  |  |  |  |  |  |  |  |  |  | A33~B41~DR01 | 0.0000 |
|  |  |  |  |  |  |  |  |  |  | A66~B14~DR01 | 0.0000 |
|  |  |  |  |  |  |  |  |  |  | A33~B41~DR13 | 0.0000 |
|  |  |  |  |  |  |  |  |  |  | A66~B35~DR15 | 0.0000 |
|  |  |  |  |  |  |  |  |  |  | A66~B35~DR13 | 0.0000 |
|  |  |  |  |  |  |  |  |  |  | A11~B14~DR13 | 0.0000 |
|  |  |  |  |  |  |  |  |  |  | A03~B14~DR14 | 0.0000 |
|  |  |  |  |  |  |  |  |  |  | A01~B49~DR15 | 0.0000 |
|  |  |  |  |  |  |  |  |  |  | A01~B57~DR09 | 0.0000 |
|  |  |  |  |  |  |  |  |  |  | A01~B27~DR14 | 0.0000 |
|  |  |  |  |  |  |  |  |  |  | A03~B57~DR04 | 0.0000 |
|  |  |  |  |  |  |  |  |  |  | A30~B27~DR01 | 0.0000 |
|  |  |  |  |  |  |  |  |  |  | A11~B07~DR01 | 0.0000 |
|  |  |  |  |  |  |  |  |  |  | A31~B18~DR11 | 0.0000 |
|  |  |  |  |  |  |  |  |  |  | A31~B59~DR11 | 0.0000 |
|  |  |  |  |  |  |  |  |  |  | A03~B59~DR11 | 0.0000 |
|  |  |  |  |  |  |  |  |  |  | A03~B59~DR15 | 0.0000 |
|  |  |  |  |  |  |  |  |  |  | A30~B38~DR04 | 0.0000 |
|  |  |  |  |  |  |  |  |  |  | A02~B37~DR14 | 0.0000 |
|  |  |  |  |  |  |  |  |  |  | A01~B44~DR14 | 0.0000 |
|  |  |  |  |  |  |  |  |  |  | A24~B57~DR11 | 0.0000 |
|  |  |  |  |  |  |  |  |  |  | A01~B57~DR10 | 0.0000 |
|  |  |  |  |  |  |  |  |  |  | A26~B37~DR03 | 0.0000 |
|  |  |  |  |  |  |  |  |  |  | A26~B37~DR10 | 0.0000 |
|  |  |  |  |  |  |  |  |  |  | A69~B51~DR03 | 0.0000 |
|  |  |  |  |  |  |  |  |  |  | A24~B15~DR03 | 0.0000 |
|  |  |  |  |  |  |  |  |  |  | A69~B51~DR15 | 0.0000 |
|  |  |  |  |  |  |  |  |  |  | A69~B15~DR15 | 0.0000 |
|  |  |  |  |  |  |  |  |  |  | A69~B15~DR11 | 0.0000 |
|  |  |  |  |  |  |  |  |  |  | A69~B40~DR03 | 0.0000 |
|  |  |  |  |  |  |  |  |  |  | A69~B40~DR11 | 0.0000 |
|  |  |  |  |  |  |  |  |  |  | A11~B40~DR03 | 0.0000 |
|  |  |  |  |  |  |  |  |  |  | A11~B15~DR12 | 0.0000 |
|  |  |  |  |  |  |  |  |  |  | A11~B15~DR03 | 0.0000 |
|  |  |  |  |  |  |  |  |  |  | A69~B40~DR12 | 0.0000 |
|  |  |  |  |  |  |  |  |  |  | A24~B40~DR15 | 0.0000 |
|  |  |  |  |  |  |  |  |  |  | A01~B18~DR16 | 0.0000 |
|  |  |  |  |  |  |  |  |  |  | A33~B07~DR11 | 0.0000 |
|  |  |  |  |  |  |  |  |  |  | A33~B18~DR16 | 0.0000 |
|  |  |  |  |  |  |  |  |  |  | A31~B07~DR11 | 0.0000 |
|  |  |  |  |  |  |  |  |  |  | A01~B07~DR01 | 0.0000 |
|  |  |  |  |  |  |  |  |  |  | A32~B44~DR13 | 0.0000 |
|  |  |  |  |  |  |  |  |  |  | A68~B49~DR16 | 0.0000 |
|  |  |  |  |  |  |  |  |  |  | A11~B38~DR14 | 0.0000 |
|  |  |  |  |  |  |  |  |  |  | A69~B13~DR01 | 0.0000 |
|  |  |  |  |  |  |  |  |  |  | A69~B35~DR01 | 0.0000 |
|  |  |  |  |  |  |  |  |  |  | A24~B51~DR12 | 0.0000 |
|  |  |  |  |  |  |  |  |  |  | A01~B35~DR09 | 0.0000 |
|  |  |  |  |  |  |  |  |  |  | A31~B50~DR12 | 0.0000 |
|  |  |  |  |  |  |  |  |  |  | A03~B51~DR12 | 0.0000 |
|  |  |  |  |  |  |  |  |  |  | A03~B50~DR12 | 0.0000 |
|  |  |  |  |  |  |  |  |  |  | A31~B51~DR07 | 0.0000 |
|  |  |  |  |  |  |  |  |  |  | A31~B51~DR10 | 0.0000 |
|  |  |  |  |  |  |  |  |  |  | A03~B37~DR15 | 0.0000 |
|  |  |  |  |  |  |  |  |  |  | A11~B13~DR13 | 0.0000 |
|  |  |  |  |  |  |  |  |  |  | A30~B07~DR13 | 0.0000 |
|  |  |  |  |  |  |  |  |  |  | A26~B44~DR07 | 0.0000 |
|  |  |  |  |  |  |  |  |  |  | A23~B35~DR13 | 0.0000 |
|  |  |  |  |  |  |  |  |  |  | A02~B13~DR01 | 0.0000 |
|  |  |  |  |  |  |  |  |  |  | A33~B50~DR11 | 0.0000 |
|  |  |  |  |  |  |  |  |  |  | A33~B18~DR14 | 0.0000 |
|  |  |  |  |  |  |  |  |  |  | A33~B41~DR04 | 0.0000 |
|  |  |  |  |  |  |  |  |  |  | A33~B41~DR11 | 0.0000 |
|  |  |  |  |  |  |  |  |  |  | A01~B13~DR08 | 0.0000 |
|  |  |  |  |  |  |  |  |  |  | A01~B45~DR13 | 0.0000 |
|  |  |  |  |  |  |  |  |  |  | A01~B45~DR08 | 0.0000 |
|  |  |  |  |  |  |  |  |  |  | A01~B13~DR01 | 0.0000 |
|  |  |  |  |  |  |  |  |  |  | A33~B18~DR01 | 0.0000 |
|  |  |  |  |  |  |  |  |  |  | A24~B38~DR15 | 0.0000 |
|  |  |  |  |  |  |  |  |  |  | A26~B15~DR14 | 0.0000 |
|  |  |  |  |  |  |  |  |  |  | A24~B49~DR09 | 0.0000 |
|  |  |  |  |  |  |  |  |  |  | A24~B38~DR09 | 0.0000 |
|  |  |  |  |  |  |  |  |  |  | A03~B49~DR09 | 0.0000 |
|  |  |  |  |  |  |  |  |  |  | A03~B15~DR15 | 0.0000 |
|  |  |  |  |  |  |  |  |  |  | A03~B38~DR15 | 0.0000 |
|  |  |  |  |  |  |  |  |  |  | A23~B18~DR15 | 0.0000 |
|  |  |  |  |  |  |  |  |  |  | A24~B49~DR15 | 0.0000 |
|  |  |  |  |  |  |  |  |  |  | A11~B38~DR11 | 0.0000 |
|  |  |  |  |  |  |  |  |  |  | A11~B18~DR03 | 0.0000 |
|  |  |  |  |  |  |  |  |  |  | A24~B44~DR12 | 0.0000 |
|  |  |  |  |  |  |  |  |  |  | A32~B58~DR03 | 0.0000 |
|  |  |  |  |  |  |  |  |  |  | A32~B58~DR12 | 0.0000 |
|  |  |  |  |  |  |  |  |  |  | A24~B58~DR12 | 0.0000 |
|  |  |  |  |  |  |  |  |  |  | A32~B08~DR12 | 0.0000 |
|  |  |  |  |  |  |  |  |  |  | A24~B08~DR12 | 0.0000 |
|  |  |  |  |  |  |  |  |  |  | A33~B58~DR12 | 0.0000 |
|  |  |  |  |  |  |  |  |  |  | A23~B58~DR15 | 0.0000 |
|  |  |  |  |  |  |  |  |  |  | A33~B07~DR03 | 0.0000 |
|  |  |  |  |  |  |  |  |  |  | A23~B58~DR03 | 0.0000 |
|  |  |  |  |  |  |  |  |  |  | A33~B07~DR15 | 0.0000 |
|  |  |  |  |  |  |  |  |  |  | A31~B57~DR11 | 0.0000 |
|  |  |  |  |  |  |  |  |  |  | A11~B57~DR01 | 0.0000 |
|  |  |  |  |  |  |  |  |  |  | A31~B18~DR01 | 0.0000 |
|  |  |  |  |  |  |  |  |  |  | A11~B57~DR11 | 0.0000 |
|  |  |  |  |  |  |  |  |  |  | A30~B35~DR12 | 0.0000 |
|  |  |  |  |  |  |  |  |  |  | A30~B13~DR03 | 0.0000 |
|  |  |  |  |  |  |  |  |  |  | A02~B41~DR14 | 0.0000 |
|  |  |  |  |  |  |  |  |  |  | A24~B41~DR14 | 0.0000 |
|  |  |  |  |  |  |  |  |  |  | A30~B08~DR16 | 0.0000 |
|  |  |  |  |  |  |  |  |  |  | A30~B55~DR03 | 0.0000 |
|  |  |  |  |  |  |  |  |  |  | A26~B44~DR16 | 0.0000 |
|  |  |  |  |  |  |  |  |  |  | A24~B48~DR14 | 0.0000 |
|  |  |  |  |  |  |  |  |  |  | A26~B48~DR13 | 0.0000 |
|  |  |  |  |  |  |  |  |  |  | A24~B48~DR16 | 0.0000 |
|  |  |  |  |  |  |  |  |  |  | A26~B27~DR14 | 0.0000 |
|  |  |  |  |  |  |  |  |  |  | A26~B35~DR15 | 0.0000 |
|  |  |  |  |  |  |  |  |  |  | A33~B35~DR03 | 0.0000 |
|  |  |  |  |  |  |  |  |  |  | A33~B27~DR15 | 0.0000 |
|  |  |  |  |  |  |  |  |  |  | A33~B27~DR13 | 0.0000 |
|  |  |  |  |  |  |  |  |  |  | A03~B15~DR09 | 0.0000 |
|  |  |  |  |  |  |  |  |  |  | A01~B40~DR03 | 0.0000 |
|  |  |  |  |  |  |  |  |  |  | A01~B07~DR04 | 0.0000 |
|  |  |  |  |  |  |  |  |  |  | A03~B37~DR08 | 0.0000 |
|  |  |  |  |  |  |  |  |  |  | A24~B58~DR10 | 0.0000 |
|  |  |  |  |  |  |  |  |  |  | A03~B58~DR10 | 0.0000 |
|  |  |  |  |  |  |  |  |  |  | A24~B37~DR08 | 0.0000 |
|  |  |  |  |  |  |  |  |  |  | A01~B37~DR08 | 0.0000 |
|  |  |  |  |  |  |  |  |  |  | A01~B58~DR10 | 0.0000 |
|  |  |  |  |  |  |  |  |  |  | A01~B58~DR08 | 0.0000 |
|  |  |  |  |  |  |  |  |  |  | A24~B58~DR09 | 0.0000 |
|  |  |  |  |  |  |  |  |  |  | A23~B49~DR08 | 0.0000 |
|  |  |  |  |  |  |  |  |  |  | A23~B58~DR09 | 0.0000 |
|  |  |  |  |  |  |  |  |  |  | A23~B58~DR08 | 0.0000 |
|  |  |  |  |  |  |  |  |  |  | A02~B37~DR16 | 0.0000 |
|  |  |  |  |  |  |  |  |  |  | A29~B07~DR08 | 0.0000 |
|  |  |  |  |  |  |  |  |  |  | A29~B35~DR08 | 0.0000 |
|  |  |  |  |  |  |  |  |  |  | A29~B35~DR10 | 0.0000 |
|  |  |  |  |  |  |  |  |  |  | A23~B35~DR08 | 0.0000 |
|  |  |  |  |  |  |  |  |  |  | A02~B50~DR08 | 0.0000 |
|  |  |  |  |  |  |  |  |  |  | A31~B44~DR14 | 0.0000 |
|  |  |  |  |  |  |  |  |  |  | A11~B14~DR04 | 0.0000 |
|  |  |  |  |  |  |  |  |  |  | A30~B44~DR04 | 0.0000 |
|  |  |  |  |  |  |  |  |  |  | A30~B14~DR01 | 0.0000 |
|  |  |  |  |  |  |  |  |  |  | A30~B58~DR01 | 0.0000 |
|  |  |  |  |  |  |  |  |  |  | A25~B07~DR15 | 0.0000 |
|  |  |  |  |  |  |  |  |  |  | A25~B07~DR13 | 0.0000 |
|  |  |  |  |  |  |  |  |  |  | A01~B13~DR14 | 0.0000 |
|  |  |  |  |  |  |  |  |  |  | A25~B13~DR13 | 0.0000 |
|  |  |  |  |  |  |  |  |  |  | A25~B35~DR07 | 0.0000 |
|  |  |  |  |  |  |  |  |  |  | A32~B40~DR07 | 0.0000 |
|  |  |  |  |  |  |  |  |  |  | A32~B40~DR14 | 0.0000 |
|  |  |  |  |  |  |  |  |  |  | A24~B38~DR01 | 0.0000 |
|  |  |  |  |  |  |  |  |  |  | A23~B55~DR15 | 0.0000 |
|  |  |  |  |  |  |  |  |  |  | A23~B55~DR04 | 0.0000 |
|  |  |  |  |  |  |  |  |  |  | A23~B51~DR15 | 0.0000 |
|  |  |  |  |  |  |  |  |  |  | A02~B14~DR14 | 0.0000 |
|  |  |  |  |  |  |  |  |  |  | A68~B51~DR03 | 0.0000 |
|  |  |  |  |  |  |  |  |  |  | A26~B51~DR03 | 0.0000 |
|  |  |  |  |  |  |  |  |  |  | A26~B50~DR04 | 0.0000 |
|  |  |  |  |  |  |  |  |  |  | A11~B15~DR04 | 0.0000 |
|  |  |  |  |  |  |  |  |  |  | A30~B53~DR04 | 0.0000 |
|  |  |  |  |  |  |  |  |  |  | A11~B53~DR04 | 0.0000 |
|  |  |  |  |  |  |  |  |  |  | A11~B53~DR03 | 0.0000 |
|  |  |  |  |  |  |  |  |  |  | A26~B57~DR11 | 0.0000 |
|  |  |  |  |  |  |  |  |  |  | A32~B14~DR13 | 0.0000 |
|  |  |  |  |  |  |  |  |  |  | A32~B14~DR15 | 0.0000 |
|  |  |  |  |  |  |  |  |  |  | A30~B50~DR04 | 0.0000 |
|  |  |  |  |  |  |  |  |  |  | A30~B15~DR04 | 0.0000 |
|  |  |  |  |  |  |  |  |  |  | A33~B51~DR03 | 0.0000 |
|  |  |  |  |  |  |  |  |  |  | A30~B51~DR03 | 0.0000 |
|  |  |  |  |  |  |  |  |  |  | A02~B56~DR13 | 0.0000 |
|  |  |  |  |  |  |  |  |  |  | A68~B56~DR11 | 0.0000 |
|  |  |  |  |  |  |  |  |  |  | A26~B41~DR07 | 0.0000 |
|  |  |  |  |  |  |  |  |  |  | A25~B55~DR11 | 0.0000 |
|  |  |  |  |  |  |  |  |  |  | A25~B44~DR11 | 0.0000 |
|  |  |  |  |  |  |  |  |  |  | A25~B41~DR07 | 0.0000 |
|  |  |  |  |  |  |  |  |  |  | A31~B18~DR03 | 0.0000 |
|  |  |  |  |  |  |  |  |  |  | A01~B50~DR15 | 0.0000 |
|  |  |  |  |  |  |  |  |  |  | A02~B37~DR07 | 0.0000 |
|  |  |  |  |  |  |  |  |  |  | A11~B37~DR11 | 0.0000 |
|  |  |  |  |  |  |  |  |  |  | A11~B18~DR16 | 0.0000 |
|  |  |  |  |  |  |  |  |  |  | A26~B18~DR16 | 0.0000 |
|  |  |  |  |  |  |  |  |  |  | A11~B41~DR14 | 0.0000 |
|  |  |  |  |  |  |  |  |  |  | A23~B45~DR11 | 0.0000 |
|  |  |  |  |  |  |  |  |  |  | A01~B45~DR11 | 0.0000 |
|  |  |  |  |  |  |  |  |  |  | A68~B39~DR12 | 0.0000 |
|  |  |  |  |  |  |  |  |  |  | A30~B39~DR04 | 0.0000 |
|  |  |  |  |  |  |  |  |  |  | A66~B18~DR04 | 0.0000 |
|  |  |  |  |  |  |  |  |  |  | A66~B51~DR16 | 0.0000 |
|  |  |  |  |  |  |  |  |  |  | A66~B51~DR04 | 0.0000 |
|  |  |  |  |  |  |  |  |  |  | A30~B18~DR16 | 0.0000 |
|  |  |  |  |  |  |  |  |  |  | A66~B41~DR12 | 0.0000 |
|  |  |  |  |  |  |  |  |  |  | A66~B41~DR16 | 0.0000 |
|  |  |  |  |  |  |  |  |  |  | A30~B41~DR16 | 0.0000 |
|  |  |  |  |  |  |  |  |  |  | A66~B18~DR12 | 0.0000 |
|  |  |  |  |  |  |  |  |  |  | A30~B51~DR12 | 0.0000 |
|  |  |  |  |  |  |  |  |  |  | A24~B41~DR12 | 0.0000 |
|  |  |  |  |  |  |  |  |  |  | A23~B27~DR03 | 0.0000 |
|  |  |  |  |  |  |  |  |  |  | A23~B27~DR16 | 0.0000 |
|  |  |  |  |  |  |  |  |  |  | A24~B59~DR13 | 0.0000 |
|  |  |  |  |  |  |  |  |  |  | A03~B39~DR15 | 0.0000 |
|  |  |  |  |  |  |  |  |  |  | A24~B52~DR16 | 0.0000 |
|  |  |  |  |  |  |  |  |  |  | A24~B13~DR04 | 0.0000 |
|  |  |  |  |  |  |  |  |  |  | A24~B13~DR14 | 0.0000 |
|  |  |  |  |  |  |  |  |  |  | A02~B13~DR14 | 0.0000 |
|  |  |  |  |  |  |  |  |  |  | A24~B44~DR08 | 0.0000 |
|  |  |  |  |  |  |  |  |  |  | A03~B44~DR08 | 0.0000 |
|  |  |  |  |  |  |  |  |  |  | A03~B13~DR10 | 0.0000 |
|  |  |  |  |  |  |  |  |  |  | A26~B18~DR01 | 0.0000 |
|  |  |  |  |  |  |  |  |  |  | A26~B51~DR01 | 0.0000 |
|  |  |  |  |  |  |  |  |  |  | A33~B38~DR11 | 0.0000 |
|  |  |  |  |  |  |  |  |  |  | A11~B38~DR13 | 0.0000 |
|  |  |  |  |  |  |  |  |  |  | A02~B57~DR01 | 0.0000 |
|  |  |  |  |  |  |  |  |  |  | A03~B40~DR15 | 0.0000 |
|  |  |  |  |  |  |  |  |  |  | A25~B15~DR04 | 0.0000 |
|  |  |  |  |  |  |  |  |  |  | A02~B07~DR14 | 0.0000 |
|  |  |  |  |  |  |  |  |  |  | A25~B52~DR04 | 0.0000 |
|  |  |  |  |  |  |  |  |  |  | A03~B50~DR08 | 0.0000 |
|  |  |  |  |  |  |  |  |  |  | A03~B38~DR07 | 0.0000 |
|  |  |  |  |  |  |  |  |  |  | A03~B52~DR07 | 0.0000 |
|  |  |  |  |  |  |  |  |  |  | A33~B41~DR03 | 0.0000 |
|  |  |  |  |  |  |  |  |  |  | A68~B41~DR03 | 0.0000 |
|  |  |  |  |  |  |  |  |  |  | A33~B27~DR14 | 0.0000 |
|  |  |  |  |  |  |  |  |  |  | A11~B27~DR14 | 0.0000 |
|  |  |  |  |  |  |  |  |  |  | A33~B35~DR08 | 0.0000 |
|  |  |  |  |  |  |  |  |  |  | A11~B27~DR08 | 0.0000 |
|  |  |  |  |  |  |  |  |  |  | A68~B08~DR13 | 0.0000 |
|  |  |  |  |  |  |  |  |  |  | A68~B49~DR03 | 0.0000 |
|  |  |  |  |  |  |  |  |  |  | A01~B18~DR13 | 0.0000 |
|  |  |  |  |  |  |  |  |  |  | A68~B27~DR15 | 0.0000 |
|  |  |  |  |  |  |  |  |  |  | A01~B27~DR15 | 0.0000 |
|  |  |  |  |  |  |  |  |  |  | A01~B27~DR03 | 0.0000 |
|  |  |  |  |  |  |  |  |  |  | A29~B08~DR11 | 0.0000 |
|  |  |  |  |  |  |  |  |  |  | A33~B55~DR03 | 0.0000 |
|  |  |  |  |  |  |  |  |  |  | A11~B55~DR03 | 0.0000 |
|  |  |  |  |  |  |  |  |  |  | A03~B27~DR01 | 0.0000 |
|  |  |  |  |  |  |  |  |  |  | A03~B58~DR01 | 0.0000 |
|  |  |  |  |  |  |  |  |  |  | A33~B27~DR12 | 0.0000 |
|  |  |  |  |  |  |  |  |  |  | A03~B58~DR12 | 0.0000 |
|  |  |  |  |  |  |  |  |  |  | A23~B50~DR13 | 0.0000 |
|  |  |  |  |  |  |  |  |  |  | A23~B51~DR03 | 0.0000 |
|  |  |  |  |  |  |  |  |  |  | A01~B07~DR07 | 0.0000 |
|  |  |  |  |  |  |  |  |  |  | A02~B57~DR15 | 0.0000 |
|  |  |  |  |  |  |  |  |  |  | A31~B13~DR14 | 0.0000 |
|  |  |  |  |  |  |  |  |  |  | A31~B48~DR07 | 0.0000 |
|  |  |  |  |  |  |  |  |  |  | A03~B48~DR07 | 0.0000 |
|  |  |  |  |  |  |  |  |  |  | A02~B37~DR03 | 0.0000 |
|  |  |  |  |  |  |  |  |  |  | A24~B37~DR04 | 0.0000 |
|  |  |  |  |  |  |  |  |  |  | A23~B37~DR01 | 0.0000 |
|  |  |  |  |  |  |  |  |  |  | A23~B37~DR11 | 0.0000 |
|  |  |  |  |  |  |  |  |  |  | A02~B59~DR04 | 0.0000 |
|  |  |  |  |  |  |  |  |  |  | A02~B59~DR14 | 0.0000 |
|  |  |  |  |  |  |  |  |  |  | A01~B59~DR04 | 0.0000 |
|  |  |  |  |  |  |  |  |  |  | A11~B53~DR13 | 0.0000 |
|  |  |  |  |  |  |  |  |  |  | A02~B53~DR14 | 0.0000 |
|  |  |  |  |  |  |  |  |  |  | A02~B37~DR01 | 0.0000 |
|  |  |  |  |  |  |  |  |  |  | A24~B44~DR10 | 0.0000 |
|  |  |  |  |  |  |  |  |  |  | A29~B51~DR13 | 0.0000 |
|  |  |  |  |  |  |  |  |  |  | A03~B40~DR12 | 0.0000 |
|  |  |  |  |  |  |  |  |  |  | A02~B08~DR12 | 0.0000 |
|  |  |  |  |  |  |  |  |  |  | A03~B49~DR12 | 0.0000 |
|  |  |  |  |  |  |  |  |  |  | A02~B49~DR12 | 0.0000 |
|  |  |  |  |  |  |  |  |  |  | A01~B18~DR03 | 0.0000 |
|  |  |  |  |  |  |  |  |  |  | A26~B08~DR11 | 0.0000 |
|  |  |  |  |  |  |  |  |  |  | A32~B55~DR04 | 0.0000 |
|  |  |  |  |  |  |  |  |  |  | A32~B08~DR13 | 0.0000 |
|  |  |  |  |  |  |  |  |  |  | A26~B08~DR13 | 0.0000 |
|  |  |  |  |  |  |  |  |  |  | A11~B08~DR13 | 0.0000 |
|  |  |  |  |  |  |  |  |  |  | A01~B52~DR03 | 0.0000 |
|  |  |  |  |  |  |  |  |  |  | A02~B50~DR10 | 0.0000 |
|  |  |  |  |  |  |  |  |  |  | A26~B39~DR14 | 0.0000 |
|  |  |  |  |  |  |  |  |  |  | A24~B39~DR14 | 0.0000 |
|  |  |  |  |  |  |  |  |  |  | A24~B39~DR13 | 0.0000 |
|  |  |  |  |  |  |  |  |  |  | A29~B38~DR15 | 0.0000 |
|  |  |  |  |  |  |  |  |  |  | A23~B08~DR15 | 0.0000 |
|  |  |  |  |  |  |  |  |  |  | A23~B52~DR03 | 0.0000 |
|  |  |  |  |  |  |  |  |  |  | A26~B07~DR01 | 0.0000 |
|  |  |  |  |  |  |  |  |  |  | A69~B18~DR04 | 0.0000 |
|  |  |  |  |  |  |  |  |  |  | A69~B18~DR11 | 0.0000 |
|  |  |  |  |  |  |  |  |  |  | A26~B07~DR08 | 0.0000 |
|  |  |  |  |  |  |  |  |  |  | A25~B14~DR15 | 0.0000 |
|  |  |  |  |  |  |  |  |  |  | A25~B14~DR01 | 0.0000 |
|  |  |  |  |  |  |  |  |  |  | A25~B07~DR04 | 0.0000 |
|  |  |  |  |  |  |  |  |  |  | A25~B27~DR15 | 0.0000 |
|  |  |  |  |  |  |  |  |  |  | A25~B18~DR07 | 0.0000 |
|  |  |  |  |  |  |  |  |  |  | A23~B40~DR04 | 0.0000 |
|  |  |  |  |  |  |  |  |  |  | A23~B39~DR16 | 0.0000 |
|  |  |  |  |  |  |  |  |  |  | A23~B39~DR07 | 0.0000 |
|  |  |  |  |  |  |  |  |  |  | A23~B44~DR16 | 0.0000 |
|  |  |  |  |  |  |  |  |  |  | A23~B56~DR15 | 0.0000 |
|  |  |  |  |  |  |  |  |  |  | A23~B56~DR07 | 0.0000 |
|  |  |  |  |  |  |  |  |  |  | A03~B56~DR07 | 0.0000 |
|  |  |  |  |  |  |  |  |  |  | A26~B07~DR03 | 0.0000 |
|  |  |  |  |  |  |  |  |  |  | A26~B15~DR03 | 0.0000 |
|  |  |  |  |  |  |  |  |  |  | A25~B27~DR11 | 0.0000 |
|  |  |  |  |  |  |  |  |  |  | A24~B57~DR04 | 0.0000 |
|  |  |  |  |  |  |  |  |  |  | A11~B56~DR04 | 0.0000 |
|  |  |  |  |  |  |  |  |  |  | A01~B27~DR12 | 0.0000 |
|  |  |  |  |  |  |  |  |  |  | A01~B08~DR12 | 0.0000 |
|  |  |  |  |  |  |  |  |  |  | A24~B13~DR01 | 0.0000 |
|  |  |  |  |  |  |  |  |  |  | A26~B07~DR11 | 0.0000 |
|  |  |  |  |  |  |  |  |  |  | A26~B55~DR07 | 0.0000 |
|  |  |  |  |  |  |  |  |  |  | A26~B58~DR07 | 0.0000 |
|  |  |  |  |  |  |  |  |  |  | A01~B55~DR07 | 0.0000 |
|  |  |  |  |  |  |  |  |  |  | A26~B58~DR04 | 0.0000 |
|  |  |  |  |  |  |  |  |  |  | A32~B13~DR14 | 0.0000 |
|  |  |  |  |  |  |  |  |  |  | A32~B13~DR11 | 0.0000 |
|  |  |  |  |  |  |  |  |  |  | A11~B14~DR07 | 0.0000 |
|  |  |  |  |  |  |  |  |  |  | A11~B47~DR07 | 0.0000 |
|  |  |  |  |  |  |  |  |  |  | A11~B47~DR13 | 0.0000 |
|  |  |  |  |  |  |  |  |  |  | A68~B47~DR07 | 0.0000 |
|  |  |  |  |  |  |  |  |  |  | A29~B14~DR15 | 0.0000 |
|  |  |  |  |  |  |  |  |  |  | A29~B40~DR15 | 0.0000 |
|  |  |  |  |  |  |  |  |  |  | A29~B40~DR01 | 0.0000 |
|  |  |  |  |  |  |  |  |  |  | A29~B51~DR03 | 0.0000 |
|  |  |  |  |  |  |  |  |  |  | A03~B38~DR10 | 0.0000 |
|  |  |  |  |  |  |  |  |  |  | A01~B54~DR14 | 0.0000 |
|  |  |  |  |  |  |  |  |  |  | A01~B54~DR15 | 0.0000 |
|  |  |  |  |  |  |  |  |  |  | A68~B52~DR08 | 0.0000 |
|  |  |  |  |  |  |  |  |  |  | A68~B52~DR01 | 0.0000 |
|  |  |  |  |  |  |  |  |  |  | A68~B58~DR01 | 0.0000 |
|  |  |  |  |  |  |  |  |  |  | A11~B58~DR16 | 0.0000 |
|  |  |  |  |  |  |  |  |  |  | A03~B58~DR16 | 0.0000 |
|  |  |  |  |  |  |  |  |  |  | A11~B44~DR01 | 0.0000 |
|  |  |  |  |  |  |  |  |  |  | A01~B52~DR16 | 0.0000 |
|  |  |  |  |  |  |  |  |  |  | A23~B40~DR14 | 0.0000 |
|  |  |  |  |  |  |  |  |  |  | A23~B40~DR13 | 0.0000 |
|  |  |  |  |  |  |  |  |  |  | A02~B27~DR12 | 0.0000 |
|  |  |  |  |  |  |  |  |  |  | A68~B14~DR01 | 0.0000 |
|  |  |  |  |  |  |  |  |  |  | A68~B14~DR04 | 0.0000 |
|  |  |  |  |  |  |  |  |  |  | A25~B07~DR07 | 0.0000 |
|  |  |  |  |  |  |  |  |  |  | A25~B44~DR15 | 0.0000 |
|  |  |  |  |  |  |  |  |  |  | A32~B41~DR01 | 0.0000 |
|  |  |  |  |  |  |  |  |  |  | A23~B41~DR01 | 0.0000 |
|  |  |  |  |  |  |  |  |  |  | A23~B07~DR04 | 0.0000 |
|  |  |  |  |  |  |  |  |  |  | A24~B44~DR09 | 0.0000 |
|  |  |  |  |  |  |  |  |  |  | A23~B44~DR09 | 0.0000 |
|  |  |  |  |  |  |  |  |  |  | A32~B08~DR11 | 0.0000 |
|  |  |  |  |  |  |  |  |  |  | A32~B38~DR03 | 0.0000 |
|  |  |  |  |  |  |  |  |  |  | A03~B08~DR16 | 0.0000 |
|  |  |  |  |  |  |  |  |  |  | A03~B13~DR03 | 0.0000 |
|  |  |  |  |  |  |  |  |  |  | A03~B49~DR07 | 0.0000 |
|  |  |  |  |  |  |  |  |  |  | A30~B49~DR07 | 0.0000 |
|  |  |  |  |  |  |  |  |  |  | A02~B53~DR01 | 0.0000 |
|  |  |  |  |  |  |  |  |  |  | A02~B53~DR03 | 0.0000 |
|  |  |  |  |  |  |  |  |  |  | A68~B08~DR01 | 0.0000 |
|  |  |  |  |  |  |  |  |  |  | A68~B53~DR03 | 0.0000 |
|  |  |  |  |  |  |  |  |  |  | A26~B08~DR16 | 0.0000 |
|  |  |  |  |  |  |  |  |  |  | A29~B55~DR11 | 0.0000 |
|  |  |  |  |  |  |  |  |  |  | A30~B35~DR15 | 0.0000 |
|  |  |  |  |  |  |  |  |  |  | A32~B13~DR01 | 0.0000 |
|  |  |  |  |  |  |  |  |  |  | A26~B58~DR14 | 0.0000 |
|  |  |  |  |  |  |  |  |  |  | A33~B38~DR04 | 0.0000 |
|  |  |  |  |  |  |  |  |  |  | A26~B13~DR14 | 0.0000 |
|  |  |  |  |  |  |  |  |  |  | A32~B39~DR15 | 0.0000 |
|  |  |  |  |  |  |  |  |  |  | A01~B27~DR16 | 0.0000 |
|  |  |  |  |  |  |  |  |  |  | A01~B41~DR16 | 0.0000 |
|  |  |  |  |  |  |  |  |  |  | A01~B40~DR16 | 0.0000 |
|  |  |  |  |  |  |  |  |  |  | A68~B38~DR14 | 0.0000 |
|  |  |  |  |  |  |  |  |  |  | A26~B57~DR15 | 0.0000 |
|  |  |  |  |  |  |  |  |  |  | A34~B50~DR07 | 0.0000 |
|  |  |  |  |  |  |  |  |  |  | A34~B07~DR03 | 0.0000 |
|  |  |  |  |  |  |  |  |  |  | A34~B07~DR07 | 0.0000 |
|  |  |  |  |  |  |  |  |  |  | A34~B44~DR03 | 0.0000 |
|  |  |  |  |  |  |  |  |  |  | A32~B44~DR09 | 0.0000 |
|  |  |  |  |  |  |  |  |  |  | A32~B40~DR09 | 0.0000 |
|  |  |  |  |  |  |  |  |  |  | A02~B55~DR08 | 0.0000 |
|  |  |  |  |  |  |  |  |  |  | A03~B18~DR08 | 0.0000 |
|  |  |  |  |  |  |  |  |  |  | A03~B55~DR08 | 0.0000 |
|  |  |  |  |  |  |  |  |  |  | A02~B52~DR03 | 0.0000 |
|  |  |  |  |  |  |  |  |  |  | A29~B35~DR14 | 0.0000 |
|  |  |  |  |  |  |  |  |  |  | A11~B58~DR14 | 0.0000 |
|  |  |  |  |  |  |  |  |  |  | A29~B58~DR01 | 0.0000 |
|  |  |  |  |  |  |  |  |  |  | A11~B38~DR01 | 0.0000 |
|  |  |  |  |  |  |  |  |  |  | A26~B58~DR01 | 0.0000 |
|  |  |  |  |  |  |  |  |  |  | A32~B13~DR04 | 0.0000 |
|  |  |  |  |  |  |  |  |  |  | A31~B41~DR15 | 0.0000 |
|  |  |  |  |  |  |  |  |  |  | A31~B52~DR07 | 0.0000 |
|  |  |  |  |  |  |  |  |  |  | A33~B53~DR01 | 0.0000 |
|  |  |  |  |  |  |  |  |  |  | A68~B53~DR08 | 0.0000 |
|  |  |  |  |  |  |  |  |  |  | A29~B53~DR01 | 0.0000 |
|  |  |  |  |  |  |  |  |  |  | A29~B53~DR08 | 0.0000 |
|  |  |  |  |  |  |  |  |  |  | A29~B15~DR10 | 0.0000 |
|  |  |  |  |  |  |  |  |  |  | A29~B15~DR16 | 0.0000 |
|  |  |  |  |  |  |  |  |  |  | A32~B50~DR16 | 0.0000 |
|  |  |  |  |  |  |  |  |  |  | A32~B37~DR14 | 0.0000 |
|  |  |  |  |  |  |  |  |  |  | A24~B37~DR14 | 0.0000 |
|  |  |  |  |  |  |  |  |  |  | A32~B44~DR16 | 0.0000 |
|  |  |  |  |  |  |  |  |  |  | A32~B18~DR13 | 0.0000 |
|  |  |  |  |  |  |  |  |  |  | A31~B55~DR04 | 0.0000 |
|  |  |  |  |  |  |  |  |  |  | A31~B55~DR14 | 0.0000 |
|  |  |  |  |  |  |  |  |  |  | A03~B55~DR12 | 0.0000 |
|  |  |  |  |  |  |  |  |  |  | A01~B18~DR07 | 0.0000 |
|  |  |  |  |  |  |  |  |  |  | A33~B18~DR13 | 0.0000 |
|  |  |  |  |  |  |  |  |  |  | A30~B18~DR13 | 0.0000 |
|  |  |  |  |  |  |  |  |  |  | A23~B37~DR10 | 0.0000 |
|  |  |  |  |  |  |  |  |  |  | A23~B37~DR13 | 0.0000 |
|  |  |  |  |  |  |  |  |  |  | A01~B55~DR15 | 0.0000 |
|  |  |  |  |  |  |  |  |  |  | A26~B58~DR08 | 0.0000 |
|  |  |  |  |  |  |  |  |  |  | A26~B14~DR14 | 0.0000 |
|  |  |  |  |  |  |  |  |  |  | A66~B07~DR16 | 0.0000 |
|  |  |  |  |  |  |  |  |  |  | A66~B18~DR15 | 0.0000 |
|  |  |  |  |  |  |  |  |  |  | A02~B57~DR03 | 0.0000 |
|  |  |  |  |  |  |  |  |  |  | A32~B18~DR07 | 0.0000 |
|  |  |  |  |  |  |  |  |  |  | A32~B55~DR15 | 0.0000 |
|  |  |  |  |  |  |  |  |  |  | A29~B37~DR11 | 0.0000 |
|  |  |  |  |  |  |  |  |  |  | A29~B37~DR16 | 0.0000 |
|  |  |  |  |  |  |  |  |  |  | A03~B58~DR14 | 0.0000 |
|  |  |  |  |  |  |  |  |  |  | A29~B18~DR14 | 0.0000 |
|  |  |  |  |  |  |  |  |  |  | A26~B07~DR14 | 0.0000 |
|  |  |  |  |  |  |  |  |  |  | A26~B40~DR15 | 0.0000 |
|  |  |  |  |  |  |  |  |  |  | A23~B57~DR15 | 0.0000 |
|  |  |  |  |  |  |  |  |  |  | A23~B57~DR11 | 0.0000 |
|  |  |  |  |  |  |  |  |  |  | A25~B08~DR11 | 0.0000 |
|  |  |  |  |  |  |  |  |  |  | A23~B08~DR11 | 0.0000 |
|  |  |  |  |  |  |  |  |  |  | A25~B49~DR03 | 0.0000 |
|  |  |  |  |  |  |  |  |  |  | A25~B49~DR11 | 0.0000 |
|  |  |  |  |  |  |  |  |  |  | A24~B57~DR15 | 0.0000 |
|  |  |  |  |  |  |  |  |  |  | A26~B73~DR04 | 0.0000 |
|  |  |  |  |  |  |  |  |  |  | A33~B73~DR03 | 0.0000 |
|  |  |  |  |  |  |  |  |  |  | A02~B73~DR03 | 0.0000 |
|  |  |  |  |  |  |  |  |  |  | A26~B14~DR07 | 0.0000 |
|  |  |  |  |  |  |  |  |  |  | A33~B73~DR15 | 0.0000 |
|  |  |  |  |  |  |  |  |  |  | A31~B48~DR11 | 0.0000 |
|  |  |  |  |  |  |  |  |  |  | A03~B48~DR11 | 0.0000 |
|  |  |  |  |  |  |  |  |  |  | A03~B48~DR12 | 0.0000 |
|  |  |  |  |  |  |  |  |  |  | A03~B37~DR16 | 0.0000 |
|  |  |  |  |  |  |  |  |  |  | A66~B55~DR04 | 0.0000 |
|  |  |  |  |  |  |  |  |  |  | A68~B41~DR16 | 0.0000 |
|  |  |  |  |  |  |  |  |  |  | A66~B18~DR13 | 0.0000 |
|  |  |  |  |  |  |  |  |  |  | A32~B52~DR16 | 0.0000 |
|  |  |  |  |  |  |  |  |  |  | A68~B52~DR04 | 0.0000 |
|  |  |  |  |  |  |  |  |  |  | A68~B52~DR16 | 0.0000 |
|  |  |  |  |  |  |  |  |  |  | A69~B35~DR07 | 0.0000 |
|  |  |  |  |  |  |  |  |  |  | A74~B50~DR11 | 0.0000 |
|  |  |  |  |  |  |  |  |  |  | A69~B50~DR11 | 0.0000 |
|  |  |  |  |  |  |  |  |  |  | A74~B35~DR07 | 0.0000 |
|  |  |  |  |  |  |  |  |  |  | A69~B50~DR07 | 0.0000 |
|  |  |  |  |  |  |  |  |  |  | A74~B35~DR11 | 0.0000 |
|  |  |  |  |  |  |  |  |  |  | A74~B51~DR07 | 0.0000 |
|  |  |  |  |  |  |  |  |  |  | A68~B07~DR13 | 0.0000 |
|  |  |  |  |  |  |  |  |  |  | A23~B51~DR08 | 0.0000 |
|  |  |  |  |  |  |  |  |  |  | A11~B40~DR16 | 0.0000 |
|  |  |  |  |  |  |  |  |  |  | A32~B40~DR15 | 0.0000 |
|  |  |  |  |  |  |  |  |  |  | A03~B49~DR16 | 0.0000 |
|  |  |  |  |  |  |  |  |  |  | A03~B03~DR04 | 0.0000 |
|  |  |  |  |  |  |  |  |  |  | A24~B24~DR16 | 0.0000 |
|  |  |  |  |  |  |  |  |  |  | A03~B03~DR16 | 0.0000 |
|  |  |  |  |  |  |  |  |  |  | A24~B24~DR04 | 0.0000 |
|  |  |  |  |  |  |  |  |  |  | A03~B24~DR04 | 0.0000 |
|  |  |  |  |  |  |  |  |  |  | A24~B03~DR16 | 0.0000 |
|  |  |  |  |  |  |  |  |  |  | A03~B24~DR16 | 0.0000 |
|  |  |  |  |  |  |  |  |  |  | A24~B03~DR04 | 0.0000 |
|  |  |  |  |  |  |  |  |  |  | A32~B44~DR14 | 0.0000 |
|  |  |  |  |  |  |  |  |  |  | A11~B51~DR10 | 0.0000 |
|  |  |  |  |  |  |  |  |  |  | A11~B40~DR10 | 0.0000 |
|  |  |  |  |  |  |  |  |  |  | A01~B48~DR08 | 0.0000 |
|  |  |  |  |  |  |  |  |  |  | A24~B58~DR04 | 0.0000 |
|  |  |  |  |  |  |  |  |  |  | A03~B13~DR14 | 0.0000 |
|  |  |  |  |  |  |  |  |  |  | A68~B57~DR01 | 0.0000 |
|  |  |  |  |  |  |  |  |  |  | A68~B57~DR03 | 0.0000 |
|  |  |  |  |  |  |  |  |  |  | A68~B38~DR01 | 0.0000 |
|  |  |  |  |  |  |  |  |  |  | A68~B41~DR14 | 0.0000 |
|  |  |  |  |  |  |  |  |  |  | A29~B50~DR03 | 0.0000 |
|  |  |  |  |  |  |  |  |  |  | A29~B50~DR04 | 0.0000 |
|  |  |  |  |  |  |  |  |  |  | A29~B38~DR03 | 0.0000 |
|  |  |  |  |  |  |  |  |  |  | A25~B38~DR15 | 0.0000 |
|  |  |  |  |  |  |  |  |  |  | A25~B38~DR13 | 0.0000 |
|  |  |  |  |  |  |  |  |  |  | A33~B55~DR14 | 0.0000 |
|  |  |  |  |  |  |  |  |  |  | A31~B51~DR16 | 0.0000 |
|  |  |  |  |  |  |  |  |  |  | A68~B07~DR10 | 0.0000 |
|  |  |  |  |  |  |  |  |  |  | A32~B50~DR03 | 0.0000 |
|  |  |  |  |  |  |  |  |  |  | A03~B53~DR11 | 0.0000 |
|  |  |  |  |  |  |  |  |  |  | A03~B53~DR13 | 0.0000 |
|  |  |  |  |  |  |  |  |  |  | A68~B53~DR16 | 0.0000 |
|  |  |  |  |  |  |  |  |  |  | A02~B53~DR11 | 0.0000 |
|  |  |  |  |  |  |  |  |  |  | A68~B27~DR16 | 0.0000 |
|  |  |  |  |  |  |  |  |  |  | A02~B53~DR16 | 0.0000 |
|  |  |  |  |  |  |  |  |  |  | A03~B27~DR16 | 0.0000 |
|  |  |  |  |  |  |  |  |  |  | A02~B58~DR12 | 0.0000 |
|  |  |  |  |  |  |  |  |  |  | A31~B58~DR12 | 0.0000 |
|  |  |  |  |  |  |  |  |  |  | A31~B58~DR04 | 0.0000 |
|  |  |  |  |  |  |  |  |  |  | A25~B14~DR08 | 0.0000 |
|  |  |  |  |  |  |  |  |  |  | A25~B49~DR08 | 0.0000 |
|  |  |  |  |  |  |  |  |  |  | A01~B50~DR11 | 0.0000 |
|  |  |  |  |  |  |  |  |  |  | A24~B18~DR12 | 0.0000 |
|  |  |  |  |  |  |  |  |  |  | A32~B39~DR12 | 0.0000 |
|  |  |  |  |  |  |  |  |  |  | A32~B35~DR12 | 0.0000 |
|  |  |  |  |  |  |  |  |  |  | A02~B58~DR15 | 0.0000 |
|  |  |  |  |  |  |  |  |  |  | A23~B47~DR13 | 0.0000 |
|  |  |  |  |  |  |  |  |  |  | A23~B47~DR11 | 0.0000 |
|  |  |  |  |  |  |  |  |  |  | A24~B47~DR11 | 0.0000 |
|  |  |  |  |  |  |  |  |  |  | A24~B50~DR13 | 0.0000 |
|  |  |  |  |  |  |  |  |  |  | A11~B47~DR11 | 0.0000 |
|  |  |  |  |  |  |  |  |  |  | A23~B51~DR14 | 0.0000 |
|  |  |  |  |  |  |  |  |  |  | A24~B53~DR14 | 0.0000 |
|  |  |  |  |  |  |  |  |  |  | A24~B53~DR04 | 0.0000 |
|  |  |  |  |  |  |  |  |  |  | A30~B37~DR13 | 0.0000 |
|  |  |  |  |  |  |  |  |  |  | A30~B39~DR08 | 0.0000 |
|  |  |  |  |  |  |  |  |  |  | A24~B37~DR15 | 0.0000 |
|  |  |  |  |  |  |  |  |  |  | A26~B52~DR11 | 0.0000 |
|  |  |  |  |  |  |  |  |  |  | A03~B52~DR03 | 0.0000 |
|  |  |  |  |  |  |  |  |  |  | A30~B41~DR14 | 0.0000 |
|  |  |  |  |  |  |  |  |  |  | A26~B14~DR13 | 0.0000 |
|  |  |  |  |  |  |  |  |  |  | A33~B38~DR01 | 0.0000 |
|  |  |  |  |  |  |  |  |  |  | A30~B44~DR01 | 0.0000 |
|  |  |  |  |  |  |  |  |  |  | A11~B51~DR12 | 0.0000 |
|  |  |  |  |  |  |  |  |  |  | A02~B52~DR12 | 0.0000 |
|  |  |  |  |  |  |  |  |  |  | A68~B50~DR15 | 0.0000 |
|  |  |  |  |  |  |  |  |  |  | A68~B07~DR04 | 0.0000 |
|  |  |  |  |  |  |  |  |  |  | A23~B44~DR03 | 0.0000 |
|  |  |  |  |  |  |  |  |  |  | A68~B44~DR08 | 0.0000 |
|  |  |  |  |  |  |  |  |  |  | A32~B50~DR12 | 0.0000 |
|  |  |  |  |  |  |  |  |  |  | A30~B49~DR08 | 0.0000 |
|  |  |  |  |  |  |  |  |  |  | A30~B35~DR08 | 0.0000 |
|  |  |  |  |  |  |  |  |  |  | A02~B49~DR08 | 0.0000 |
|  |  |  |  |  |  |  |  |  |  | A29~B39~DR13 | 0.0000 |
|  |  |  |  |  |  |  |  |  |  | A29~B15~DR14 | 0.0000 |
|  |  |  |  |  |  |  |  |  |  | A03~B39~DR14 | 0.0000 |
|  |  |  |  |  |  |  |  |  |  | A29~B15~DR13 | 0.0000 |
|  |  |  |  |  |  |  |  |  |  | A31~B44~DR15 | 0.0000 |
|  |  |  |  |  |  |  |  |  |  | A31~B15~DR03 | 0.0000 |
|  |  |  |  |  |  |  |  |  |  | A31~B15~DR14 | 0.0000 |
|  |  |  |  |  |  |  |  |  |  | A31~B44~DR13 | 0.0000 |
|  |  |  |  |  |  |  |  |  |  | A31~B15~DR16 | 0.0000 |
|  |  |  |  |  |  |  |  |  |  | A69~B44~DR11 | 0.0000 |
|  |  |  |  |  |  |  |  |  |  | A69~B35~DR15 | 0.0000 |
|  |  |  |  |  |  |  |  |  |  | A30~B35~DR09 | 0.0000 |
|  |  |  |  |  |  |  |  |  |  | A03~B40~DR09 | 0.0000 |
|  |  |  |  |  |  |  |  |  |  | A30~B40~DR16 | 0.0000 |
|  |  |  |  |  |  |  |  |  |  | A30~B40~DR14 | 0.0000 |
|  |  |  |  |  |  |  |  |  |  | A23~B38~DR07 | 0.0000 |
|  |  |  |  |  |  |  |  |  |  | A11~B13~DR01 | 0.0000 |
|  |  |  |  |  |  |  |  |  |  | A68~B37~DR04 | 0.0000 |
|  |  |  |  |  |  |  |  |  |  | A29~B37~DR04 | 0.0000 |
|  |  |  |  |  |  |  |  |  |  | A68~B50~DR09 | 0.0000 |
|  |  |  |  |  |  |  |  |  |  | A01~B53~DR11 | 0.0000 |
|  |  |  |  |  |  |  |  |  |  | A01~B53~DR07 | 0.0000 |
|  |  |  |  |  |  |  |  |  |  | A68~B44~DR12 | 0.0000 |
|  |  |  |  |  |  |  |  |  |  | A68~B40~DR16 | 0.0000 |
|  |  |  |  |  |  |  |  |  |  | A68~B40~DR15 | 0.0000 |
|  |  |  |  |  |  |  |  |  |  | A25~B18~DR14 | 0.0000 |
|  |  |  |  |  |  |  |  |  |  | A25~B40~DR14 | 0.0000 |
|  |  |  |  |  |  |  |  |  |  | A29~B08~DR15 | 0.0000 |
|  |  |  |  |  |  |  |  |  |  | A24~B41~DR10 | 0.0000 |
|  |  |  |  |  |  |  |  |  |  | A30~B55~DR07 | 0.0000 |
|  |  |  |  |  |  |  |  |  |  | A30~B40~DR07 | 0.0000 |
|  |  |  |  |  |  |  |  |  |  | A03~B57~DR15 | 0.0000 |
|  |  |  |  |  |  |  |  |  |  | A03~B57~DR11 | 0.0000 |
|  |  |  |  |  |  |  |  |  |  | A03~B39~DR07 | 0.0000 |
|  |  |  |  |  |  |  |  |  |  | A29~B40~DR13 | 0.0000 |
|  |  |  |  |  |  |  |  |  |  | A29~B18~DR13 | 0.0000 |
|  |  |  |  |  |  |  |  |  |  | A32~B07~DR03 | 0.0000 |
|  |  |  |  |  |  |  |  |  |  | A24~B18~DR03 | 0.0000 |
|  |  |  |  |  |  |  |  |  |  | A32~B37~DR01 | 0.0000 |
|  |  |  |  |  |  |  |  |  |  | A33~B37~DR01 | 0.0000 |
|  |  |  |  |  |  |  |  |  |  | A29~B15~DR04 | 0.0000 |
|  |  |  |  |  |  |  |  |  |  | A29~B07~DR01 | 0.0000 |
|  |  |  |  |  |  |  |  |  |  | A33~B07~DR04 | 0.0000 |
|  |  |  |  |  |  |  |  |  |  | A66~B41~DR03 | 0.0000 |
|  |  |  |  |  |  |  |  |  |  | A66~B51~DR13 | 0.0000 |
|  |  |  |  |  |  |  |  |  |  | A66~B07~DR07 | 0.0000 |
|  |  |  |  |  |  |  |  |  |  | A66~B13~DR13 | 0.0000 |
|  |  |  |  |  |  |  |  |  |  | A32~B13~DR13 | 0.0000 |
|  |  |  |  |  |  |  |  |  |  | A24~B08~DR01 | 0.0000 |
|  |  |  |  |  |  |  |  |  |  | A69~B08~DR11 | 0.0000 |
|  |  |  |  |  |  |  |  |  |  | A69~B08~DR03 | 0.0000 |
|  |  |  |  |  |  |  |  |  |  | A32~B51~DR03 | 0.0000 |
|  |  |  |  |  |  |  |  |  |  | A32~B08~DR01 | 0.0000 |
|  |  |  |  |  |  |  |  |  |  | A01~B39~DR15 | 0.0000 |
|  |  |  |  |  |  |  |  |  |  | A01~B39~DR08 | 0.0000 |
|  |  |  |  |  |  |  |  |  |  | A68~B55~DR11 | 0.0000 |
|  |  |  |  |  |  |  |  |  |  | A33~B27~DR16 | 0.0000 |
|  |  |  |  |  |  |  |  |  |  | A03~B14~DR16 | 0.0000 |
|  |  |  |  |  |  |  |  |  |  | A11~B50~DR16 | 0.0000 |
|  |  |  |  |  |  |  |  |  |  | A11~B27~DR16 | 0.0000 |
|  |  |  |  |  |  |  |  |  |  | A26~B50~DR16 | 0.0000 |
|  |  |  |  |  |  |  |  |  |  | A25~B18~DR16 | 0.0000 |
|  |  |  |  |  |  |  |  |  |  | A26~B55~DR15 | 0.0000 |
|  |  |  |  |  |  |  |  |  |  | A03~B13~DR16 | 0.0000 |
|  |  |  |  |  |  |  |  |  |  | A11~B40~DR13 | 0.0000 |
|  |  |  |  |  |  |  |  |  |  | A01~B38~DR01 | 0.0000 |
|  |  |  |  |  |  |  |  |  |  | A23~B38~DR01 | 0.0000 |
|  |  |  |  |  |  |  |  |  |  | A23~B38~DR14 | 0.0000 |
|  |  |  |  |  |  |  |  |  |  | A68~B18~DR07 | 0.0000 |
|  |  |  |  |  |  |  |  |  |  | A23~B41~DR14 | 0.0000 |
|  |  |  |  |  |  |  |  |  |  | A03~B41~DR14 | 0.0000 |
|  |  |  |  |  |  |  |  |  |  | A03~B41~DR01 | 0.0000 |
|  |  |  |  |  |  |  |  |  |  | A33~B49~DR11 | 0.0000 |
|  |  |  |  |  |  |  |  |  |  | A23~B50~DR14 | 0.0000 |
|  |  |  |  |  |  |  |  |  |  | A23~B40~DR07 | 0.0000 |
|  |  |  |  |  |  |  |  |  |  | A30~B07~DR14 | 0.0000 |
|  |  |  |  |  |  |  |  |  |  | A11~B55~DR08 | 0.0000 |
|  |  |  |  |  |  |  |  |  |  | A11~B44~DR08 | 0.0000 |
|  |  |  |  |  |  |  |  |  |  | A26~B55~DR08 | 0.0000 |
|  |  |  |  |  |  |  |  |  |  | A01~B39~DR13 | 0.0000 |
|  |  |  |  |  |  |  |  |  |  | A25~B44~DR01 | 0.0000 |
|  |  |  |  |  |  |  |  |  |  | A25~B35~DR16 | 0.0000 |
|  |  |  |  |  |  |  |  |  |  | A03~B50~DR10 | 0.0000 |
|  |  |  |  |  |  |  |  |  |  | A29~B13~DR08 | 0.0000 |
|  |  |  |  |  |  |  |  |  |  | A23~B45~DR15 | 0.0000 |
|  |  |  |  |  |  |  |  |  |  | A26~B50~DR15 | 0.0000 |
|  |  |  |  |  |  |  |  |  |  | A23~B45~DR13 | 0.0000 |
|  |  |  |  |  |  |  |  |  |  | A26~B45~DR15 | 0.0000 |
|  |  |  |  |  |  |  |  |  |  | A02~B52~DR10 | 0.0000 |
|  |  |  |  |  |  |  |  |  |  | A24~B41~DR16 | 0.0000 |
|  |  |  |  |  |  |  |  |  |  | A01~B51~DR12 | 0.0000 |
|  |  |  |  |  |  |  |  |  |  | A01~B18~DR12 | 0.0000 |
|  |  |  |  |  |  |  |  |  |  | A33~B18~DR12 | 0.0000 |
|  |  |  |  |  |  |  |  |  |  | A68~B08~DR10 | 0.0000 |
|  |  |  |  |  |  |  |  |  |  | A68~B18~DR03 | 0.0000 |
|  |  |  |  |  |  |  |  |  |  | A31~B18~DR12 | 0.0000 |
|  |  |  |  |  |  |  |  |  |  | A33~B39~DR01 | 0.0000 |
|  |  |  |  |  |  |  |  |  |  | A31~B07~DR04 | 0.0000 |
|  |  |  |  |  |  |  |  |  |  | A31~B07~DR13 | 0.0000 |
|  |  |  |  |  |  |  |  |  |  | A26~B50~DR03 | 0.0000 |
|  |  |  |  |  |  |  |  |  |  | A32~B49~DR10 | 0.0000 |
|  |  |  |  |  |  |  |  |  |  | A32~B49~DR11 | 0.0000 |
|  |  |  |  |  |  |  |  |  |  | A23~B49~DR10 | 0.0000 |
|  |  |  |  |  |  |  |  |  |  | A68~B49~DR15 | 0.0000 |
|  |  |  |  |  |  |  |  |  |  | A01~B51~DR09 | 0.0000 |
|  |  |  |  |  |  |  |  |  |  | A01~B08~DR09 | 0.0000 |
|  |  |  |  |  |  |  |  |  |  | A26~B08~DR01 | 0.0000 |
|  |  |  |  |  |  |  |  |  |  | A29~B08~DR13 | 0.0000 |
|  |  |  |  |  |  |  |  |  |  | A29~B44~DR03 | 0.0000 |
|  |  |  |  |  |  |  |  |  |  | A03~B38~DR16 | 0.0000 |
|  |  |  |  |  |  |  |  |  |  | A24~B08~DR16 | 0.0000 |
|  |  |  |  |  |  |  |  |  |  | A26~B08~DR14 | 0.0000 |
|  |  |  |  |  |  |  |  |  |  | A32~B08~DR14 | 0.0000 |
|  |  |  |  |  |  |  |  |  |  | A29~B44~DR15 | 0.0000 |
|  |  |  |  |  |  |  |  |  |  | A32~B18~DR08 | 0.0000 |
|  |  |  |  |  |  |  |  |  |  | A30~B51~DR08 | 0.0000 |
|  |  |  |  |  |  |  |  |  |  | A32~B18~DR12 | 0.0000 |
|  |  |  |  |  |  |  |  |  |  | A30~B18~DR08 | 0.0000 |
|  |  |  |  |  |  |  |  |  |  | A30~B18~DR04 | 0.0000 |
|  |  |  |  |  |  |  |  |  |  | A30~B44~DR12 | 0.0000 |
|  |  |  |  |  |  |  |  |  |  | A11~B08~DR14 | 0.0000 |
|  |  |  |  |  |  |  |  |  |  | A69~B08~DR04 | 0.0000 |
|  |  |  |  |  |  |  |  |  |  | A69~B37~DR04 | 0.0000 |
|  |  |  |  |  |  |  |  |  |  | A69~B37~DR13 | 0.0000 |
|  |  |  |  |  |  |  |  |  |  | A02~B48~DR01 | 0.0000 |
|  |  |  |  |  |  |  |  |  |  | A01~B48~DR01 | 0.0000 |
|  |  |  |  |  |  |  |  |  |  | A11~B55~DR12 | 0.0000 |
|  |  |  |  |  |  |  |  |  |  | A11~B44~DR12 | 0.0000 |
|  |  |  |  |  |  |  |  |  |  | A02~B55~DR12 | 0.0000 |
|  |  |  |  |  |  |  |  |  |  | A11~B49~DR07 | 0.0000 |
|  |  |  |  |  |  |  |  |  |  | A32~B52~DR14 | 0.0000 |
|  |  |  |  |  |  |  |  |  |  | A32~B15~DR01 | 0.0000 |
|  |  |  |  |  |  |  |  |  |  | A68~B08~DR04 | 0.0000 |
|  |  |  |  |  |  |  |  |  |  | A01~B15~DR08 | 0.0000 |
|  |  |  |  |  |  |  |  |  |  | A32~B37~DR12 | 0.0000 |
|  |  |  |  |  |  |  |  |  |  | A32~B37~DR16 | 0.0000 |
|  |  |  |  |  |  |  |  |  |  | A31~B49~DR11 | 0.0000 |
|  |  |  |  |  |  |  |  |  |  | A29~B57~DR04 | 0.0000 |
|  |  |  |  |  |  |  |  |  |  | A29~B57~DR11 | 0.0000 |
|  |  |  |  |  |  |  |  |  |  | A23~B15~DR11 | 0.0000 |
|  |  |  |  |  |  |  |  |  |  | A32~B49~DR16 | 0.0000 |
|  |  |  |  |  |  |  |  |  |  | A01~B49~DR16 | 0.0000 |
|  |  |  |  |  |  |  |  |  |  | A30~B57~DR13 | 0.0000 |
|  |  |  |  |  |  |  |  |  |  | A31~B18~DR04 | 0.0000 |
|  |  |  |  |  |  |  |  |  |  | A26~B13~DR13 | 0.0000 |
|  |  |  |  |  |  |  |  |  |  | A31~B38~DR15 | 0.0000 |
|  |  |  |  |  |  |  |  |  |  | A30~B58~DR16 | 0.0000 |
|  |  |  |  |  |  |  |  |  |  | A31~B38~DR16 | 0.0000 |
|  |  |  |  |  |  |  |  |  |  | A30~B58~DR15 | 0.0000 |
|  |  |  |  |  |  |  |  |  |  | A31~B58~DR15 | 0.0000 |
|  |  |  |  |  |  |  |  |  |  | A11~B48~DR03 | 0.0000 |
|  |  |  |  |  |  |  |  |  |  | A11~B48~DR04 | 0.0000 |
|  |  |  |  |  |  |  |  |  |  | A24~B48~DR03 | 0.0000 |
|  |  |  |  |  |  |  |  |  |  | A30~B07~DR07 | 0.0000 |
|  |  |  |  |  |  |  |  |  |  | A32~B58~DR01 | 0.0000 |
|  |  |  |  |  |  |  |  |  |  | A32~B58~DR04 | 0.0000 |
|  |  |  |  |  |  |  |  |  |  | A26~B41~DR04 | 0.0000 |
|  |  |  |  |  |  |  |  |  |  | A66~B39~DR10 | 0.0000 |
|  |  |  |  |  |  |  |  |  |  | A26~B41~DR10 | 0.0000 |
|  |  |  |  |  |  |  |  |  |  | A66~B39~DR04 | 0.0000 |
|  |  |  |  |  |  |  |  |  |  | A26~B39~DR10 | 0.0000 |
|  |  |  |  |  |  |  |  |  |  | A01~B52~DR10 | 0.0000 |
|  |  |  |  |  |  |  |  |  |  | A01~B41~DR01 | 0.0000 |
|  |  |  |  |  |  |  |  |  |  | A66~B52~DR10 | 0.0000 |
|  |  |  |  |  |  |  |  |  |  | A66~B52~DR01 | 0.0000 |
|  |  |  |  |  |  |  |  |  |  | A26~B39~DR13 | 0.0000 |
|  |  |  |  |  |  |  |  |  |  | A33~B38~DR03 | 0.0000 |
|  |  |  |  |  |  |  |  |  |  | A33~B27~DR04 | 0.0000 |
|  |  |  |  |  |  |  |  |  |  | A01~B27~DR08 | 0.0000 |
|  |  |  |  |  |  |  |  |  |  | A01~B18~DR08 | 0.0000 |
|  |  |  |  |  |  |  |  |  |  | A30~B51~DR09 | 0.0000 |
|  |  |  |  |  |  |  |  |  |  | A68~B13~DR04 | 0.0000 |
|  |  |  |  |  |  |  |  |  |  | A23~B48~DR07 | 0.0000 |
|  |  |  |  |  |  |  |  |  |  | A23~B48~DR11 | 0.0000 |
|  |  |  |  |  |  |  |  |  |  | A24~B48~DR07 | 0.0000 |
|  |  |  |  |  |  |  |  |  |  | A69~B07~DR07 | 0.0000 |
|  |  |  |  |  |  |  |  |  |  | A69~B57~DR11 | 0.0000 |
|  |  |  |  |  |  |  |  |  |  | A69~B13~DR07 | 0.0000 |
|  |  |  |  |  |  |  |  |  |  | A69~B13~DR11 | 0.0000 |
|  |  |  |  |  |  |  |  |  |  | A29~B13~DR01 | 0.0000 |
|  |  |  |  |  |  |  |  |  |  | A25~B13~DR04 | 0.0000 |
|  |  |  |  |  |  |  |  |  |  | A25~B35~DR04 | 0.0000 |
|  |  |  |  |  |  |  |  |  |  | A11~B54~DR11 | 0.0000 |
|  |  |  |  |  |  |  |  |  |  | A01~B54~DR11 | 0.0000 |
|  |  |  |  |  |  |  |  |  |  | A01~B54~DR13 | 0.0000 |
|  |  |  |  |  |  |  |  |  |  | A25~B51~DR04 | 0.0000 |
|  |  |  |  |  |  |  |  |  |  | A26~B56~DR10 | 0.0000 |
|  |  |  |  |  |  |  |  |  |  | A01~B56~DR10 | 0.0000 |
|  |  |  |  |  |  |  |  |  |  | A26~B37~DR01 | 0.0000 |
|  |  |  |  |  |  |  |  |  |  | A26~B56~DR04 | 0.0000 |
|  |  |  |  |  |  |  |  |  |  | A33~B18~DR04 | 0.0000 |
|  |  |  |  |  |  |  |  |  |  | A02~B14~DR08 | 0.0000 |
|  |  |  |  |  |  |  |  |  |  | A01~B07~DR08 | 0.0000 |
|  |  |  |  |  |  |  |  |  |  | A01~B14~DR08 | 0.0000 |
|  |  |  |  |  |  |  |  |  |  | A24~B14~DR15 | 0.0000 |
|  |  |  |  |  |  |  |  |  |  | A68~B27~DR01 | 0.0000 |
|  |  |  |  |  |  |  |  |  |  | A03~B49~DR08 | 0.0000 |
|  |  |  |  |  |  |  |  |  |  | A01~B49~DR08 | 0.0000 |
|  |  |  |  |  |  |  |  |  |  | A32~B35~DR08 | 0.0000 |
|  |  |  |  |  |  |  |  |  |  | A31~B49~DR04 | 0.0000 |
|  |  |  |  |  |  |  |  |  |  | A01~B47~DR13 | 0.0000 |
|  |  |  |  |  |  |  |  |  |  | A23~B14~DR16 | 0.0000 |
|  |  |  |  |  |  |  |  |  |  | A31~B15~DR10 | 0.0000 |
|  |  |  |  |  |  |  |  |  |  | A01~B55~DR01 | 0.0000 |
|  |  |  |  |  |  |  |  |  |  | A25~B50~DR13 | 0.0000 |
|  |  |  |  |  |  |  |  |  |  | A25~B51~DR12 | 0.0000 |
|  |  |  |  |  |  |  |  |  |  | A02~B50~DR12 | 0.0000 |
|  |  |  |  |  |  |  |  |  |  | A30~B52~DR15 | 0.0000 |
|  |  |  |  |  |  |  |  |  |  | A24~B53~DR15 | 0.0000 |
|  |  |  |  |  |  |  |  |  |  | A30~B52~DR11 | 0.0000 |
|  |  |  |  |  |  |  |  |  |  | A11~B50~DR14 | 0.0000 |
|  |  |  |  |  |  |  |  |  |  | A25~B50~DR04 | 0.0000 |
|  |  |  |  |  |  |  |  |  |  | A26~B15~DR01 | 0.0000 |
|  |  |  |  |  |  |  |  |  |  | A23~B41~DR10 | 0.0000 |
|  |  |  |  |  |  |  |  |  |  | A23~B35~DR10 | 0.0000 |
|  |  |  |  |  |  |  |  |  |  | A01~B15~DR10 | 0.0000 |
|  |  |  |  |  |  |  |  |  |  | A11~B15~DR10 | 0.0000 |
|  |  |  |  |  |  |  |  |  |  | A01~B15~DR16 | 0.0000 |
|  |  |  |  |  |  |  |  |  |  | A02~B45~DR11 | 0.0000 |
|  |  |  |  |  |  |  |  |  |  | A11~B57~DR15 | 0.0000 |
|  |  |  |  |  |  |  |  |  |  | A02~B57~DR12 | 0.0000 |
|  |  |  |  |  |  |  |  |  |  | A24~B13~DR12 | 0.0000 |
|  |  |  |  |  |  |  |  |  |  | A32~B38~DR01 | 0.0000 |
|  |  |  |  |  |  |  |  |  |  | A69~B51~DR14 | 0.0000 |
|  |  |  |  |  |  |  |  |  |  | A69~B40~DR14 | 0.0000 |
|  |  |  |  |  |  |  |  |  |  | A23~B55~DR01 | 0.0000 |
|  |  |  |  |  |  |  |  |  |  | A32~B15~DR14 | 0.0000 |
|  |  |  |  |  |  |  |  |  |  | A66~B08~DR03 | 0.0000 |
|  |  |  |  |  |  |  |  |  |  | A01~B57~DR12 | 0.0000 |
|  |  |  |  |  |  |  |  |  |  | A24~B48~DR15 | 0.0000 |
|  |  |  |  |  |  |  |  |  |  | A24~B57~DR12 | 0.0000 |
|  |  |  |  |  |  |  |  |  |  | A03~B44~DR12 | 0.0000 |
|  |  |  |  |  |  |  |  |  |  | A03~B48~DR16 | 0.0000 |
|  |  |  |  |  |  |  |  |  |  | A03~B57~DR16 | 0.0000 |
|  |  |  |  |  |  |  |  |  |  | A68~B38~DR08 | 0.0000 |
|  |  |  |  |  |  |  |  |  |  | A23~B15~DR07 | 0.0000 |
|  |  |  |  |  |  |  |  |  |  | A68~B38~DR07 | 0.0000 |
|  |  |  |  |  |  |  |  |  |  | A33~B41~DR15 | 0.0000 |
|  |  |  |  |  |  |  |  |  |  | A29~B51~DR09 | 0.0000 |
|  |  |  |  |  |  |  |  |  |  | A29~B44~DR09 | 0.0000 |
|  |  |  |  |  |  |  |  |  |  | A26~B51~DR09 | 0.0000 |
|  |  |  |  |  |  |  |  |  |  | A26~B35~DR09 | 0.0000 |
|  |  |  |  |  |  |  |  |  |  | A03~B44~DR09 | 0.0000 |
|  |  |  |  |  |  |  |  |  |  | A03~B27~DR09 | 0.0000 |
|  |  |  |  |  |  |  |  |  |  | A01~B40~DR01 | 0.0000 |
|  |  |  |  |  |  |  |  |  |  | A01~B52~DR14 | 0.0000 |
|  |  |  |  |  |  |  |  |  |  | A66~B41~DR09 | 0.0000 |
|  |  |  |  |  |  |  |  |  |  | A66~B40~DR09 | 0.0000 |
|  |  |  |  |  |  |  |  |  |  | A68~B41~DR09 | 0.0000 |
|  |  |  |  |  |  |  |  |  |  | A66~B40~DR11 | 0.0000 |
|  |  |  |  |  |  |  |  |  |  | A69~B45~DR13 | 0.0000 |
|  |  |  |  |  |  |  |  |  |  | A66~B45~DR11 | 0.0000 |
|  |  |  |  |  |  |  |  |  |  | A66~B45~DR13 | 0.0000 |
|  |  |  |  |  |  |  |  |  |  | A69~B45~DR14 | 0.0000 |
|  |  |  |  |  |  |  |  |  |  | A68~B45~DR11 | 0.0000 |
|  |  |  |  |  |  |  |  |  |  | A69~B35~DR14 | 0.0000 |
|  |  |  |  |  |  |  |  |  |  | A68~B45~DR14 | 0.0000 |
|  |  |  |  |  |  |  |  |  |  | A26~B27~DR13 | 0.0000 |
|  |  |  |  |  |  |  |  |  |  | A11~B50~DR01 | 0.0000 |
|  |  |  |  |  |  |  |  |  |  | A66~B48~DR04 | 0.0000 |
|  |  |  |  |  |  |  |  |  |  | A66~B48~DR13 | 0.0000 |
|  |  |  |  |  |  |  |  |  |  | A02~B48~DR13 | 0.0000 |
|  |  |  |  |  |  |  |  |  |  | A66~B41~DR08 | 0.0000 |
|  |  |  |  |  |  |  |  |  |  | A30~B41~DR13 | 0.0000 |
|  |  |  |  |  |  |  |  |  |  | A66~B38~DR08 | 0.0000 |
|  |  |  |  |  |  |  |  |  |  | A30~B41~DR08 | 0.0000 |
|  |  |  |  |  |  |  |  |  |  | A66~B38~DR13 | 0.0000 |
|  |  |  |  |  |  |  |  |  |  | A30~B48~DR04 | 0.0000 |
|  |  |  |  |  |  |  |  |  |  | A03~B48~DR08 | 0.0000 |
|  |  |  |  |  |  |  |  |  |  | A32~B38~DR08 | 0.0000 |
|  |  |  |  |  |  |  |  |  |  | A11~B38~DR08 | 0.0000 |
|  |  |  |  |  |  |  |  |  |  | A30~B38~DR11 | 0.0000 |
|  |  |  |  |  |  |  |  |  |  | A32~B53~DR03 | 0.0000 |
|  |  |  |  |  |  |  |  |  |  | A30~B38~DR03 | 0.0000 |
|  |  |  |  |  |  |  |  |  |  | A11~B53~DR15 | 0.0000 |
|  |  |  |  |  |  |  |  |  |  | A33~B13~DR11 | 0.0000 |
|  |  |  |  |  |  |  |  |  |  | A66~B39~DR16 | 0.0000 |
|  |  |  |  |  |  |  |  |  |  | A66~B39~DR13 | 0.0000 |
|  |  |  |  |  |  |  |  |  |  | A66~B55~DR16 | 0.0000 |
|  |  |  |  |  |  |  |  |  |  | A11~B13~DR03 | 0.0000 |
|  |  |  |  |  |  |  |  |  |  | A24~B54~DR15 | 0.0000 |
|  |  |  |  |  |  |  |  |  |  | A24~B54~DR04 | 0.0000 |
|  |  |  |  |  |  |  |  |  |  | A24~B49~DR12 | 0.0000 |
|  |  |  |  |  |  |  |  |  |  | A01~B49~DR12 | 0.0000 |
|  |  |  |  |  |  |  |  |  |  | A31~B35~DR15 | 0.0000 |
|  |  |  |  |  |  |  |  |  |  | A11~B37~DR04 | 0.0000 |
|  |  |  |  |  |  |  |  |  |  | A02~B57~DR10 | 0.0000 |
|  |  |  |  |  |  |  |  |  |  | A68~B48~DR08 | 0.0000 |
|  |  |  |  |  |  |  |  |  |  | A31~B35~DR08 | 0.0000 |
|  |  |  |  |  |  |  |  |  |  | A68~B48~DR04 | 0.0000 |
|  |  |  |  |  |  |  |  |  |  | A31~B48~DR08 | 0.0000 |
|  |  |  |  |  |  |  |  |  |  | A26~B14~DR11 | 0.0000 |
|  |  |  |  |  |  |  |  |  |  | A23~B13~DR03 | 0.0000 |
|  |  |  |  |  |  |  |  |  |  | A23~B35~DR09 | 0.0000 |
|  |  |  |  |  |  |  |  |  |  | A23~B14~DR03 | 0.0000 |
|  |  |  |  |  |  |  |  |  |  | A03~B13~DR09 | 0.0000 |
|  |  |  |  |  |  |  |  |  |  | A23~B14~DR15 | 0.0000 |
|  |  |  |  |  |  |  |  |  |  | A68~B38~DR10 | 0.0000 |
|  |  |  |  |  |  |  |  |  |  | A38~B51~DR07 | 0.0000 |
|  |  |  |  |  |  |  |  |  |  | A38~B51~DR13 | 0.0000 |
|  |  |  |  |  |  |  |  |  |  | A38~B44~DR13 | 0.0000 |
|  |  |  |  |  |  |  |  |  |  | A03~B07~DR09 | 0.0000 |
|  |  |  |  |  |  |  |  |  |  | A01~B07~DR09 | 0.0000 |
|  |  |  |  |  |  |  |  |  |  | A11~B35~DR09 | 0.0000 |
|  |  |  |  |  |  |  |  |  |  | A11~B07~DR16 | 0.0000 |
|  |  |  |  |  |  |  |  |  |  | A11~B07~DR14 | 0.0000 |
|  |  |  |  |  |  |  |  |  |  | A31~B44~DR03 | 0.0000 |
|  |  |  |  |  |  |  |  |  |  | A25~B51~DR11 | 0.0000 |
|  |  |  |  |  |  |  |  |  |  | A25~B18~DR03 | 0.0000 |
|  |  |  |  |  |  |  |  |  |  | A23~B56~DR08 | 0.0000 |
|  |  |  |  |  |  |  |  |  |  | A23~B56~DR01 | 0.0000 |
|  |  |  |  |  |  |  |  |  |  | A23~B44~DR08 | 0.0000 |
|  |  |  |  |  |  |  |  |  |  | A01~B56~DR04 | 0.0000 |
|  |  |  |  |  |  |  |  |  |  | A30~B44~DR15 | 0.0000 |
|  |  |  |  |  |  |  |  |  |  | A24~B58~DR16 | 0.0000 |
|  |  |  |  |  |  |  |  |  |  | A30~B51~DR14 | 0.0000 |
|  |  |  |  |  |  |  |  |  |  | A33~B50~DR07 | 0.0000 |
|  |  |  |  |  |  |  |  |  |  | A33~B50~DR13 | 0.0000 |
|  |  |  |  |  |  |  |  |  |  | A30~B07~DR03 | 0.0000 |
|  |  |  |  |  |  |  |  |  |  | A03~B54~DR14 | 0.0000 |
|  |  |  |  |  |  |  |  |  |  | A24~B58~DR07 | 0.0000 |
|  |  |  |  |  |  |  |  |  |  | A32~B56~DR04 | 0.0000 |
|  |  |  |  |  |  |  |  |  |  | A32~B56~DR12 | 0.0000 |
|  |  |  |  |  |  |  |  |  |  | A24~B56~DR12 | 0.0000 |
|  |  |  |  |  |  |  |  |  |  | A68~B35~DR09 | 0.0000 |
|  |  |  |  |  |  |  |  |  |  | A02~B52~DR08 | 0.0000 |
|  |  |  |  |  |  |  |  |  |  | A26~B52~DR13 | 0.0000 |
|  |  |  |  |  |  |  |  |  |  | A11~B08~DR08 | 0.0000 |
|  |  |  |  |  |  |  |  |  |  | A68~B08~DR11 | 0.0000 |
|  |  |  |  |  |  |  |  |  |  | A23~B14~DR13 | 0.0000 |
|  |  |  |  |  |  |  |  |  |  | A23~B14~DR04 | 0.0000 |
|  |  |  |  |  |  |  |  |  |  | A30~B55~DR04 | 0.0000 |
|  |  |  |  |  |  |  |  |  |  | A11~B07~DR08 | 0.0000 |
|  |  |  |  |  |  |  |  |  |  | A11~B49~DR14 | 0.0000 |
|  |  |  |  |  |  |  |  |  |  | A24~B50~DR08 | 0.0000 |
|  |  |  |  |  |  |  |  |  |  | A26~B56~DR07 | 0.0000 |
|  |  |  |  |  |  |  |  |  |  | A24~B56~DR07 | 0.0000 |
|  |  |  |  |  |  |  |  |  |  | A26~B50~DR08 | 0.0000 |
|  |  |  |  |  |  |  |  |  |  | A24~B56~DR08 | 0.0000 |
|  |  |  |  |  |  |  |  |  |  | A26~B56~DR03 | 0.0000 |
|  |  |  |  |  |  |  |  |  |  | A24~B56~DR03 | 0.0000 |
|  |  |  |  |  |  |  |  |  |  | A31~B55~DR11 | 0.0000 |
|  |  |  |  |  |  |  |  |  |  | A24~B39~DR09 | 0.0000 |
|  |  |  |  |  |  |  |  |  |  | A03~B39~DR09 | 0.0000 |
|  |  |  |  |  |  |  |  |  |  | A03~B52~DR09 | 0.0000 |
|  |  |  |  |  |  |  |  |  |  | A33~B39~DR09 | 0.0000 |
|  |  |  |  |  |  |  |  |  |  | A02~B14~DR09 | 0.0000 |
|  |  |  |  |  |  |  |  |  |  | A33~B14~DR09 | 0.0000 |
|  |  |  |  |  |  |  |  |  |  | A02~B73~DR08 | 0.0000 |
|  |  |  |  |  |  |  |  |  |  | A30~B51~DR07 | 0.0000 |
|  |  |  |  |  |  |  |  |  |  | A30~B40~DR15 | 0.0000 |
|  |  |  |  |  |  |  |  |  |  | A11~B40~DR09 | 0.0000 |
|  |  |  |  |  |  |  |  |  |  | A33~B49~DR16 | 0.0000 |
|  |  |  |  |  |  |  |  |  |  | A33~B15~DR16 | 0.0000 |
|  |  |  |  |  |  |  |  |  |  | A29~B40~DR11 | 0.0000 |
|  |  |  |  |  |  |  |  |  |  | A11~B07~DR10 | 0.0000 |
|  |  |  |  |  |  |  |  |  |  | A29~B40~DR10 | 0.0000 |
|  |  |  |  |  |  |  |  |  |  | A33~B40~DR01 | 0.0000 |
|  |  |  |  |  |  |  |  |  |  | A33~B40~DR11 | 0.0000 |
|  |  |  |  |  |  |  |  |  |  | A11~B56~DR07 | 0.0000 |
|  |  |  |  |  |  |  |  |  |  | A24~B56~DR15 | 0.0000 |
|  |  |  |  |  |  |  |  |  |  | A11~B57~DR07 | 0.0000 |
|  |  |  |  |  |  |  |  |  |  | A03~B08~DR14 | 0.0000 |
|  |  |  |  |  |  |  |  |  |  | A03~B39~DR01 | 0.0000 |
|  |  |  |  |  |  |  |  |  |  | A33~B56~DR04 | 0.0000 |
|  |  |  |  |  |  |  |  |  |  | A03~B56~DR04 | 0.0000 |
|  |  |  |  |  |  |  |  |  |  | A03~B56~DR01 | 0.0000 |
|  |  |  |  |  |  |  |  |  |  | A24~B08~DR09 | 0.0000 |
|  |  |  |  |  |  |  |  |  |  | A24~B52~DR09 | 0.0000 |
|  |  |  |  |  |  |  |  |  |  | A33~B08~DR16 | 0.0000 |
|  |  |  |  |  |  |  |  |  |  | A02~B48~DR16 | 0.0000 |
|  |  |  |  |  |  |  |  |  |  | A23~B40~DR16 | 0.0000 |
|  |  |  |  |  |  |  |  |  |  | A23~B53~DR14 | 0.0000 |
|  |  |  |  |  |  |  |  |  |  | A23~B53~DR11 | 0.0000 |
|  |  |  |  |  |  |  |  |  |  | A33~B48~DR11 | 0.0000 |
|  |  |  |  |  |  |  |  |  |  | A23~B48~DR16 | 0.0000 |
|  |  |  |  |  |  |  |  |  |  | A26~B13~DR03 | 0.0000 |
|  |  |  |  |  |  |  |  |  |  | A11~B59~DR03 | 0.0000 |
|  |  |  |  |  |  |  |  |  |  | A03~B59~DR03 | 0.0000 |
|  |  |  |  |  |  |  |  |  |  | A33~B49~DR01 | 0.0000 |
|  |  |  |  |  |  |  |  |  |  | A24~B40~DR10 | 0.0000 |
|  |  |  |  |  |  |  |  |  |  | A26~B40~DR10 | 0.0000 |
|  |  |  |  |  |  |  |  |  |  | A24~B08~DR10 | 0.0000 |
|  |  |  |  |  |  |  |  |  |  | A26~B18~DR07 | 0.0000 |
|  |  |  |  |  |  |  |  |  |  | A29~B08~DR10 | 0.0000 |
|  |  |  |  |  |  |  |  |  |  | A26~B18~DR10 | 0.0000 |
|  |  |  |  |  |  |  |  |  |  | A29~B08~DR07 | 0.0000 |
|  |  |  |  |  |  |  |  |  |  | A29~B18~DR10 | 0.0000 |
|  |  |  |  |  |  |  |  |  |  | A29~B40~DR07 | 0.0000 |
|  |  |  |  |  |  |  |  |  |  | A01~B41~DR14 | 0.0000 |
|  |  |  |  |  |  |  |  |  |  | A32~B55~DR11 | 0.0000 |
|  |  |  |  |  |  |  |  |  |  | A32~B41~DR07 | 0.0000 |
|  |  |  |  |  |  |  |  |  |  | A24~B55~DR10 | 0.0000 |
|  |  |  |  |  |  |  |  |  |  | A01~B55~DR10 | 0.0000 |
|  |  |  |  |  |  |  |  |  |  | A31~B51~DR09 | 0.0000 |
|  |  |  |  |  |  |  |  |  |  | A31~B41~DR01 | 0.0000 |
|  |  |  |  |  |  |  |  |  |  | A31~B41~DR13 | 0.0000 |
|  |  |  |  |  |  |  |  |  |  | A31~B51~DR01 | 0.0000 |
|  |  |  |  |  |  |  |  |  |  | A11~B56~DR10 | 0.0000 |
|  |  |  |  |  |  |  |  |  |  | A68~B56~DR01 | 0.0000 |
|  |  |  |  |  |  |  |  |  |  | A68~B56~DR10 | 0.0000 |
|  |  |  |  |  |  |  |  |  |  | A29~B38~DR08 | 0.0000 |
|  |  |  |  |  |  |  |  |  |  | A03~B57~DR01 | 0.0000 |
|  |  |  |  |  |  |  |  |  |  | A11~B58~DR07 | 0.0000 |
|  |  |  |  |  |  |  |  |  |  | A29~B58~DR07 | 0.0000 |
|  |  |  |  |  |  |  |  |  |  | A68~B37~DR03 | 0.0000 |
|  |  |  |  |  |  |  |  |  |  | A29~B52~DR13 | 0.0000 |
|  |  |  |  |  |  |  |  |  |  | A29~B58~DR13 | 0.0000 |
|  |  |  |  |  |  |  |  |  |  | A02~B45~DR07 | 0.0000 |
|  |  |  |  |  |  |  |  |  |  | A03~B07~DR12 | 0.0000 |
|  |  |  |  |  |  |  |  |  |  | A32~B08~DR07 | 0.0000 |
|  |  |  |  |  |  |  |  |  |  | A02~B53~DR15 | 0.0000 |
|  |  |  |  |  |  |  |  |  |  | A01~B53~DR01 | 0.0000 |
|  |  |  |  |  |  |  |  |  |  | A22~B08~DR03 | 0.0000 |
|  |  |  |  |  |  |  |  |  |  | A22~B08~DR14 | 0.0000 |
|  |  |  |  |  |  |  |  |  |  | A22~B55~DR03 | 0.0000 |
|  |  |  |  |  |  |  |  |  |  | A29~B55~DR14 | 0.0000 |
|  |  |  |  |  |  |  |  |  |  | A29~B41~DR14 | 0.0000 |
|  |  |  |  |  |  |  |  |  |  | A33~B08~DR15 | 0.0000 |
|  |  |  |  |  |  |  |  |  |  | A24~B27~DR10 | 0.0000 |
|  |  |  |  |  |  |  |  |  |  | A68~B39~DR01 | 0.0000 |
|  |  |  |  |  |  |  |  |  |  | A68~B14~DR16 | 0.0000 |
|  |  |  |  |  |  |  |  |  |  | A32~B49~DR14 | 0.0000 |
|  |  |  |  |  |  |  |  |  |  | A32~B49~DR15 | 0.0000 |
|  |  |  |  |  |  |  |  |  |  | A29~B49~DR13 | 0.0000 |
|  |  |  |  |  |  |  |  |  |  | A69~B35~DR03 | 0.0000 |
|  |  |  |  |  |  |  |  |  |  | A30~B15~DR03 | 0.0000 |
|  |  |  |  |  |  |  |  |  |  | A69~B15~DR14 | 0.0000 |
|  |  |  |  |  |  |  |  |  |  | A24~B49~DR10 | 0.0000 |
|  |  |  |  |  |  |  |  |  |  | A30~B41~DR10 | 0.0000 |
|  |  |  |  |  |  |  |  |  |  | A11~B41~DR10 | 0.0000 |
|  |  |  |  |  |  |  |  |  |  | A11~B49~DR03 | 0.0000 |
|  |  |  |  |  |  |  |  |  |  | A29~B49~DR03 | 0.0000 |
|  |  |  |  |  |  |  |  |  |  | A66~B35~DR16 | 0.0000 |
|  |  |  |  |  |  |  |  |  |  | A66~B18~DR11 | 0.0000 |
|  |  |  |  |  |  |  |  |  |  | A66~B52~DR15 | 0.0000 |
|  |  |  |  |  |  |  |  |  |  | A66~B52~DR16 | 0.0000 |
|  |  |  |  |  |  |  |  |  |  | A33~B48~DR03 | 0.0000 |
|  |  |  |  |  |  |  |  |  |  | A01~B48~DR16 | 0.0000 |
|  |  |  |  |  |  |  |  |  |  | A26~B07~DR16 | 0.0000 |
|  |  |  |  |  |  |  |  |  |  | A33~B48~DR15 | 0.0000 |
|  |  |  |  |  |  |  |  |  |  | A26~B48~DR15 | 0.0000 |
|  |  |  |  |  |  |  |  |  |  | A24~B37~DR12 | 0.0000 |
|  |  |  |  |  |  |  |  |  |  | A01~B37~DR12 | 0.0000 |
|  |  |  |  |  |  |  |  |  |  | A33~B51~DR12 | 0.0000 |
|  |  |  |  |  |  |  |  |  |  | A33~B51~DR16 | 0.0000 |
|  |  |  |  |  |  |  |  |  |  | A32~B38~DR16 | 0.0000 |
|  |  |  |  |  |  |  |  |  |  | A23~B52~DR13 | 0.0000 |
|  |  |  |  |  |  |  |  |  |  | A11~B49~DR10 | 0.0000 |
|  |  |  |  |  |  |  |  |  |  | A01~B39~DR03 | 0.0000 |
|  |  |  |  |  |  |  |  |  |  | A26~B57~DR16 | 0.0000 |
|  |  |  |  |  |  |  |  |  |  | A29~B40~DR04 | 0.0000 |
|  |  |  |  |  |  |  |  |  |  | A69~B55~DR14 | 0.0000 |
|  |  |  |  |  |  |  |  |  |  | A33~B45~DR11 | 0.0000 |
|  |  |  |  |  |  |  |  |  |  | A69~B14~DR15 | 0.0000 |
|  |  |  |  |  |  |  |  |  |  | A69~B14~DR11 | 0.0000 |
|  |  |  |  |  |  |  |  |  |  | A31~B45~DR11 | 0.0000 |
|  |  |  |  |  |  |  |  |  |  | A69~B35~DR16 | 0.0000 |
|  |  |  |  |  |  |  |  |  |  | A11~B14~DR03 | 0.0000 |
|  |  |  |  |  |  |  |  |  |  | A26~B14~DR15 | 0.0000 |
|  |  |  |  |  |  |  |  |  |  | A29~B51~DR01 | 0.0000 |
|  |  |  |  |  |  |  |  |  |  | A26~B57~DR01 | 0.0000 |
|  |  |  |  |  |  |  |  |  |  | A25~B52~DR16 | 0.0000 |
|  |  |  |  |  |  |  |  |  |  | A25~B51~DR16 | 0.0000 |
|  |  |  |  |  |  |  |  |  |  | A25~B52~DR10 | 0.0000 |
|  |  |  |  |  |  |  |  |  |  | A25~B51~DR10 | 0.0000 |
|  |  |  |  |  |  |  |  |  |  | A31~B50~DR09 | 0.0000 |
|  |  |  |  |  |  |  |  |  |  | A31~B50~DR16 | 0.0000 |
|  |  |  |  |  |  |  |  |  |  | A02~B50~DR09 | 0.0000 |
|  |  |  |  |  |  |  |  |  |  | A25~B52~DR13 | 0.0000 |
|  |  |  |  |  |  |  |  |  |  | A03~B58~DR13 | 0.0000 |
|  |  |  |  |  |  |  |  |  |  | A11~B37~DR09 | 0.0000 |
|  |  |  |  |  |  |  |  |  |  | A30~B39~DR11 | 0.0000 |
|  |  |  |  |  |  |  |  |  |  | A29~B39~DR08 | 0.0000 |
|  |  |  |  |  |  |  |  |  |  | A29~B39~DR01 | 0.0000 |
|  |  |  |  |  |  |  |  |  |  | A29~B14~DR08 | 0.0000 |
|  |  |  |  |  |  |  |  |  |  | A29~B39~DR04 | 0.0000 |
|  |  |  |  |  |  |  |  |  |  | A68~B52~DR14 | 0.0000 |
|  |  |  |  |  |  |  |  |  |  | A23~B39~DR08 | 0.0000 |
|  |  |  |  |  |  |  |  |  |  | A29~B49~DR01 | 0.0000 |
|  |  |  |  |  |  |  |  |  |  | A03~B15~DR03 | 0.0000 |
|  |  |  |  |  |  |  |  |  |  | A33~B39~DR13 | 0.0000 |
|  |  |  |  |  |  |  |  |  |  | A11~B03~DR13 | 0.0000 |
|  |  |  |  |  |  |  |  |  |  | A03~B03~DR13 | 0.0000 |
|  |  |  |  |  |  |  |  |  |  | A03~B03~DR03 | 0.0000 |
|  |  |  |  |  |  |  |  |  |  | A01~B19~DR03 | 0.0000 |
|  |  |  |  |  |  |  |  |  |  | A32~B13~DR08 | 0.0000 |
|  |  |  |  |  |  |  |  |  |  | A32~B13~DR16 | 0.0000 |
|  |  |  |  |  |  |  |  |  |  | A24~B13~DR16 | 0.0000 |
|  |  |  |  |  |  |  |  |  |  | A29~B40~DR14 | 0.0000 |
|  |  |  |  |  |  |  |  |  |  | A68~B53~DR15 | 0.0000 |
|  |  |  |  |  |  |  |  |  |  | A03~B08~DR10 | 0.0000 |
|  |  |  |  |  |  |  |  |  |  | A25~B44~DR04 | 0.0000 |
|  |  |  |  |  |  |  |  |  |  | A25~B27~DR16 | 0.0000 |
|  |  |  |  |  |  |  |  |  |  | A29~B27~DR04 | 0.0000 |
|  |  |  |  |  |  |  |  |  |  | A24~B48~DR01 | 0.0000 |
|  |  |  |  |  |  |  |  |  |  | A25~B57~DR07 | 0.0000 |
|  |  |  |  |  |  |  |  |  |  | A25~B57~DR04 | 0.0000 |
|  |  |  |  |  |  |  |  |  |  | A11~B38~DR03 | 0.0000 |
|  |  |  |  |  |  |  |  |  |  | A32~B13~DR03 | 0.0000 |
|  |  |  |  |  |  |  |  |  |  | A69~B08~DR15 | 0.0000 |
|  |  |  |  |  |  |  |  |  |  | A69~B18~DR03 | 0.0000 |
|  |  |  |  |  |  |  |  |  |  | A30~B57~DR07 | 0.0000 |
|  |  |  |  |  |  |  |  |  |  | A30~B57~DR04 | 0.0000 |
|  |  |  |  |  |  |  |  |  |  | A31~B40~DR15 | 0.0000 |
|  |  |  |  |  |  |  |  |  |  | A69~B40~DR15 | 0.0000 |
|  |  |  |  |  |  |  |  |  |  | A69~B40~DR04 | 0.0000 |
|  |  |  |  |  |  |  |  |  |  | A69~B44~DR15 | 0.0000 |
|  |  |  |  |  |  |  |  |  |  | A31~B48~DR01 | 0.0000 |
|  |  |  |  |  |  |  |  |  |  | A02~B48~DR03 | 0.0000 |
|  |  |  |  |  |  |  |  |  |  | A33~B13~DR04 | 0.0000 |
|  |  |  |  |  |  |  |  |  |  | A32~B15~DR07 | 0.0000 |
|  |  |  |  |  |  |  |  |  |  | A11~B13~DR16 | 0.0000 |
|  |  |  |  |  |  |  |  |  |  | A11~B57~DR03 | 0.0000 |
|  |  |  |  |  |  |  |  |  |  | A23~B08~DR07 | 0.0000 |
|  |  |  |  |  |  |  |  |  |  | A02~B41~DR12 | 0.0000 |
|  |  |  |  |  |  |  |  |  |  | A01~B49~DR10 | 0.0000 |
|  |  |  |  |  |  |  |  |  |  | A30~B08~DR10 | 0.0000 |
|  |  |  |  |  |  |  |  |  |  | A11~B49~DR01 | 0.0000 |
|  |  |  |  |  |  |  |  |  |  | A26~B41~DR14 | 0.0000 |
|  |  |  |  |  |  |  |  |  |  | A29~B44~DR08 | 0.0000 |
|  |  |  |  |  |  |  |  |  |  | A29~B58~DR10 | 0.0000 |
|  |  |  |  |  |  |  |  |  |  | A29~B37~DR08 | 0.0000 |
|  |  |  |  |  |  |  |  |  |  | A29~B37~DR10 | 0.0000 |
|  |  |  |  |  |  |  |  |  |  | A23~B15~DR16 | 0.0000 |
|  |  |  |  |  |  |  |  |  |  | A23~B52~DR14 | 0.0000 |
|  |  |  |  |  |  |  |  |  |  | A23~B52~DR11 | 0.0000 |
|  |  |  |  |  |  |  |  |  |  | A33~B55~DR04 | 0.0000 |
|  |  |  |  |  |  |  |  |  |  | A25~B53~DR12 | 0.0000 |
|  |  |  |  |  |  |  |  |  |  | A26~B18~DR12 | 0.0000 |
|  |  |  |  |  |  |  |  |  |  | A25~B53~DR15 | 0.0000 |
|  |  |  |  |  |  |  |  |  |  | A26~B53~DR15 | 0.0000 |
|  |  |  |  |  |  |  |  |  |  | A69~B18~DR14 | 0.0000 |
|  |  |  |  |  |  |  |  |  |  | A66~B56~DR01 | 0.0000 |
|  |  |  |  |  |  |  |  |  |  | A66~B56~DR13 | 0.0000 |
|  |  |  |  |  |  |  |  |  |  | A11~B56~DR13 | 0.0000 |
|  |  |  |  |  |  |  |  |  |  | A03~B52~DR08 | 0.0000 |
|  |  |  |  |  |  |  |  |  |  | A32~B42~DR03 | 0.0000 |
|  |  |  |  |  |  |  |  |  |  | A32~B42~DR11 | 0.0000 |
|  |  |  |  |  |  |  |  |  |  | A68~B42~DR11 | 0.0000 |
|  |  |  |  |  |  |  |  |  |  | A25~B41~DR04 | 0.0000 |
|  |  |  |  |  |  |  |  |  |  | A25~B44~DR13 | 0.0000 |
|  |  |  |  |  |  |  |  |  |  | A25~B41~DR03 | 0.0000 |
|  |  |  |  |  |  |  |  |  |  | A33~B07~DR07 | 0.0000 |
|  |  |  |  |  |  |  |  |  |  | A33~B07~DR13 | 0.0000 |
|  |  |  |  |  |  |  |  |  |  | A25~B35~DR03 | 0.0000 |
|  |  |  |  |  |  |  |  |  |  | A23~B13~DR16 | 0.0000 |
|  |  |  |  |  |  |  |  |  |  | A30~B13~DR14 | 0.0000 |
|  |  |  |  |  |  |  |  |  |  | A24~B27~DR08 | 0.0000 |
|  |  |  |  |  |  |  |  |  |  | A03~B27~DR08 | 0.0000 |
|  |  |  |  |  |  |  |  |  |  | A03~B50~DR13 | 0.0000 |
|  |  |  |  |  |  |  |  |  |  | A30~B44~DR13 | 0.0000 |
|  |  |  |  |  |  |  |  |  |  | A30~B44~DR03 | 0.0000 |
|  |  |  |  |  |  |  |  |  |  | A32~B52~DR09 | 0.0000 |
|  |  |  |  |  |  |  |  |  |  | A68~B52~DR09 | 0.0000 |
|  |  |  |  |  |  |  |  |  |  | A68~B14~DR13 | 0.0000 |
|  |  |  |  |  |  |  |  |  |  | A33~B40~DR09 | 0.0000 |
|  |  |  |  |  |  |  |  |  |  | A68~B14~DR09 | 0.0000 |
|  |  |  |  |  |  |  |  |  |  | A29~B50~DR01 | 0.0000 |
|  |  |  |  |  |  |  |  |  |  | A26~B50~DR12 | 0.0000 |
|  |  |  |  |  |  |  |  |  |  | A29~B15~DR01 | 0.0000 |
|  |  |  |  |  |  |  |  |  |  | A29~B15~DR11 | 0.0000 |
|  |  |  |  |  |  |  |  |  |  | A29~B14~DR03 | 0.0000 |
|  |  |  |  |  |  |  |  |  |  | A30~B08~DR08 | 0.0000 |
|  |  |  |  |  |  |  |  |  |  | A26~B08~DR08 | 0.0000 |
|  |  |  |  |  |  |  |  |  |  | A26~B13~DR08 | 0.0000 |
|  |  |  |  |  |  |  |  |  |  | A31~B07~DR15 | 0.0000 |
|  |  |  |  |  |  |  |  |  |  | A68~B53~DR14 | 0.0000 |
|  |  |  |  |  |  |  |  |  |  | A01~B53~DR13 | 0.0000 |
|  |  |  |  |  |  |  |  |  |  | A32~B27~DR03 | 0.0000 |
|  |  |  |  |  |  |  |  |  |  | A25~B50~DR07 | 0.0000 |
|  |  |  |  |  |  |  |  |  |  | A30~B41~DR15 | 0.0000 |
|  |  |  |  |  |  |  |  |  |  | A66~B13~DR11 | 0.0000 |
|  |  |  |  |  |  |  |  |  |  | A66~B49~DR11 | 0.0000 |
|  |  |  |  |  |  |  |  |  |  | A66~B07~DR11 | 0.0000 |
|  |  |  |  |  |  |  |  |  |  | A01~B13~DR15 | 0.0000 |
|  |  |  |  |  |  |  |  |  |  | A26~B27~DR08 | 0.0000 |
|  |  |  |  |  |  |  |  |  |  | A26~B58~DR15 | 0.0000 |
|  |  |  |  |  |  |  |  |  |  | A31~B18~DR13 | 0.0000 |
|  |  |  |  |  |  |  |  |  |  | A11~B55~DR01 | 0.0000 |
|  |  |  |  |  |  |  |  |  |  | A11~B14~DR14 | 0.0000 |
|  |  |  |  |  |  |  |  |  |  | A33~B55~DR01 | 0.0000 |
|  |  |  |  |  |  |  |  |  |  | A23~B38~DR03 | 0.0000 |
|  |  |  |  |  |  |  |  |  |  | A26~B49~DR03 | 0.0000 |
|  |  |  |  |  |  |  |  |  |  | A30~B27~DR15 | 0.0000 |
|  |  |  |  |  |  |  |  |  |  | A23~B27~DR15 | 0.0000 |
|  |  |  |  |  |  |  |  |  |  | A23~B27~DR13 | 0.0000 |
|  |  |  |  |  |  |  |  |  |  | A23~B18~DR03 | 0.0000 |
|  |  |  |  |  |  |  |  |  |  | A30~B27~DR03 | 0.0000 |
|  |  |  |  |  |  |  |  |  |  | A30~B15~DR11 | 0.0000 |
|  |  |  |  |  |  |  |  |  |  | A30~B50~DR14 | 0.0000 |
|  |  |  |  |  |  |  |  |  |  | A30~B50~DR11 | 0.0000 |
|  |  |  |  |  |  |  |  |  |  | A33~B57~DR04 | 0.0000 |
|  |  |  |  |  |  |  |  |  |  | A33~B57~DR11 | 0.0000 |
|  |  |  |  |  |  |  |  |  |  | A30~B37~DR14 | 0.0000 |
|  |  |  |  |  |  |  |  |  |  | A02~B37~DR04 | 0.0000 |
|  |  |  |  |  |  |  |  |  |  | A24~B14~DR14 | 0.0000 |
|  |  |  |  |  |  |  |  |  |  | A01~B44~DR09 | 0.0000 |
|  |  |  |  |  |  |  |  |  |  | A30~B49~DR09 | 0.0000 |
|  |  |  |  |  |  |  |  |  |  | A31~B49~DR13 | 0.0000 |
|  |  |  |  |  |  |  |  |  |  | A31~B49~DR09 | 0.0000 |
|  |  |  |  |  |  |  |  |  |  | A30~B41~DR01 | 0.0000 |
|  |  |  |  |  |  |  |  |  |  | A30~B18~DR14 | 0.0000 |
|  |  |  |  |  |  |  |  |  |  | A30~B57~DR14 | 0.0000 |
|  |  |  |  |  |  |  |  |  |  | A30~B57~DR11 | 0.0000 |
|  |  |  |  |  |  |  |  |  |  | A29~B49~DR04 | 0.0000 |
|  |  |  |  |  |  |  |  |  |  | A24~B73~DR04 | 0.0000 |
|  |  |  |  |  |  |  |  |  |  | A03~B57~DR09 | 0.0000 |
|  |  |  |  |  |  |  |  |  |  | A69~B07~DR09 | 0.0000 |
|  |  |  |  |  |  |  |  |  |  | A69~B07~DR15 | 0.0000 |
|  |  |  |  |  |  |  |  |  |  | A69~B18~DR09 | 0.0000 |
|  |  |  |  |  |  |  |  |  |  | A03~B73~DR04 | 0.0000 |
|  |  |  |  |  |  |  |  |  |  | A03~B73~DR11 | 0.0000 |
|  |  |  |  |  |  |  |  |  |  | A01~B73~DR11 | 0.0000 |
|  |  |  |  |  |  |  |  |  |  | A11~B55~DR10 | 0.0000 |
|  |  |  |  |  |  |  |  |  |  | A33~B58~DR09 | 0.0000 |
|  |  |  |  |  |  |  |  |  |  | A33~B07~DR09 | 0.0000 |
|  |  |  |  |  |  |  |  |  |  | A02~B58~DR09 | 0.0000 |
|  |  |  |  |  |  |  |  |  |  | A31~B08~DR04 | 0.0000 |
|  |  |  |  |  |  |  |  |  |  | A25~B55~DR13 | 0.0000 |
|  |  |  |  |  |  |  |  |  |  | A66~B15~DR13 | 0.0000 |
|  |  |  |  |  |  |  |  |  |  | A26~B15~DR16 | 0.0000 |
|  |  |  |  |  |  |  |  |  |  | A02~B46~DR13 | 0.0000 |
|  |  |  |  |  |  |  |  |  |  | A32~B15~DR12 | 0.0000 |
|  |  |  |  |  |  |  |  |  |  | A11~B39~DR14 | 0.0000 |
|  |  |  |  |  |  |  |  |  |  | A31~B39~DR07 | 0.0000 |
|  |  |  |  |  |  |  |  |  |  | A31~B15~DR07 | 0.0000 |
|  |  |  |  |  |  |  |  |  |  | A68~B40~DR08 | 0.0000 |
|  |  |  |  |  |  |  |  |  |  | A68~B50~DR16 | 0.0000 |
|  |  |  |  |  |  |  |  |  |  | A33~B48~DR12 | 0.0000 |
|  |  |  |  |  |  |  |  |  |  | A33~B48~DR07 | 0.0000 |
|  |  |  |  |  |  |  |  |  |  | A24~B49~DR16 | 0.0000 |
|  |  |  |  |  |  |  |  |  |  | A24~B46~DR09 | 0.0000 |
|  |  |  |  |  |  |  |  |  |  | A24~B46~DR16 | 0.0000 |
|  |  |  |  |  |  |  |  |  |  | A02~B46~DR16 | 0.0000 |
|  |  |  |  |  |  |  |  |  |  | A68~B13~DR14 | 0.0000 |
|  |  |  |  |  |  |  |  |  |  | A69~B41~DR01 | 0.0000 |
|  |  |  |  |  |  |  |  |  |  | A69~B18~DR01 | 0.0000 |
|  |  |  |  |  |  |  |  |  |  | A69~B18~DR07 | 0.0000 |
|  |  |  |  |  |  |  |  |  |  | A68~B55~DR01 | 0.0000 |
|  |  |  |  |  |  |  |  |  |  | A68~B18~DR01 | 0.0000 |
|  |  |  |  |  |  |  |  |  |  | A69~B13~DR13 | 0.0000 |
|  |  |  |  |  |  |  |  |  |  | A68~B13~DR13 | 0.0000 |
|  |  |  |  |  |  |  |  |  |  | A11~B15~DR09 | 0.0000 |
|  |  |  |  |  |  |  |  |  |  | A11~B39~DR10 | 0.0000 |
|  |  |  |  |  |  |  |  |  |  | A29~B55~DR07 | 0.0000 |
|  |  |  |  |  |  |  |  |  |  | A36~B55~DR03 | 0.0000 |
|  |  |  |  |  |  |  |  |  |  | A36~B55~DR15 | 0.0000 |
|  |  |  |  |  |  |  |  |  |  | A36~B52~DR03 | 0.0000 |
|  |  |  |  |  |  |  |  |  |  | A30~B08~DR12 | 0.0000 |
|  |  |  |  |  |  |  |  |  |  | A26~B08~DR12 | 0.0000 |
|  |  |  |  |  |  |  |  |  |  | A31~B57~DR03 | 0.0000 |
|  |  |  |  |  |  |  |  |  |  | A01~B13~DR12 | 0.0000 |
|  |  |  |  |  |  |  |  |  |  | A31~B48~DR09 | 0.0000 |
|  |  |  |  |  |  |  |  |  |  | A02~B48~DR09 | 0.0000 |
|  |  |  |  |  |  |  |  |  |  | A36~B08~DR03 | 0.0000 |
|  |  |  |  |  |  |  |  |  |  | A36~B08~DR15 | 0.0000 |
|  |  |  |  |  |  |  |  |  |  | A01~B50~DR12 | 0.0000 |
|  |  |  |  |  |  |  |  |  |  | A23~B52~DR12 | 0.0000 |
|  |  |  |  |  |  |  |  |  |  | A23~B50~DR12 | 0.0000 |
|  |  |  |  |  |  |  |  |  |  | A23~B15~DR03 | 0.0000 |
|  |  |  |  |  |  |  |  |  |  | A36~B52~DR13 | 0.0000 |
|  |  |  |  |  |  |  |  |  |  | A36~B44~DR15 | 0.0000 |
|  |  |  |  |  |  |  |  |  |  | A36~B44~DR13 | 0.0000 |
|  |  |  |  |  |  |  |  |  |  | A11~B48~DR14 | 0.0000 |
|  |  |  |  |  |  |  |  |  |  | A33~B48~DR14 | 0.0000 |
|  |  |  |  |  |  |  |  |  |  | A02~B38~DR12 | 0.0000 |
|  |  |  |  |  |  |  |  |  |  | A31~B39~DR09 | 0.0000 |
|  |  |  |  |  |  |  |  |  |  | A31~B39~DR03 | 0.0000 |
|  |  |  |  |  |  |  |  |  |  | A29~B50~DR13 | 0.0000 |
|  |  |  |  |  |  |  |  |  |  | A30~B08~DR07 | 0.0000 |
|  |  |  |  |  |  |  |  |  |  | A30~B50~DR13 | 0.0000 |
|  |  |  |  |  |  |  |  |  |  | A30~B50~DR03 | 0.0000 |
|  |  |  |  |  |  |  |  |  |  | A30~B55~DR01 | 0.0000 |
|  |  |  |  |  |  |  |  |  |  | A31~B27~DR16 | 0.0000 |
|  |  |  |  |  |  |  |  |  |  | A11~B57~DR16 | 0.0000 |
|  |  |  |  |  |  |  |  |  |  | A11~B41~DR16 | 0.0000 |
|  |  |  |  |  |  |  |  |  |  | A68~B55~DR16 | 0.0000 |
|  |  |  |  |  |  |  |  |  |  | A11~B48~DR16 | 0.0000 |
|  |  |  |  |  |  |  |  |  |  | A33~B58~DR07 | 0.0000 |
|  |  |  |  |  |  |  |  |  |  | A25~B15~DR13 | 0.0000 |
|  |  |  |  |  |  |  |  |  |  | A30~B44~DR16 | 0.0000 |
|  |  |  |  |  |  |  |  |  |  | A02~B56~DR04 | 0.0000 |
|  |  |  |  |  |  |  |  |  |  | A03~B38~DR01 | 0.0000 |
|  |  |  |  |  |  |  |  |  |  | A03~B56~DR11 | 0.0000 |
|  |  |  |  |  |  |  |  |  |  | A03~B18~DR09 | 0.0000 |
|  |  |  |  |  |  |  |  |  |  | A68~B58~DR13 | 0.0000 |
|  |  |  |  |  |  |  |  |  |  | A68~B58~DR11 | 0.0000 |
|  |  |  |  |  |  |  |  |  |  | A24~B37~DR03 | 0.0000 |
|  |  |  |  |  |  |  |  |  |  | A03~B37~DR03 | 0.0000 |
|  |  |  |  |  |  |  |  |  |  | A30~B18~DR15 | 0.0000 |
|  |  |  |  |  |  |  |  |  |  | A68~B34~DR11 | 0.0000 |
|  |  |  |  |  |  |  |  |  |  | A24~B34~DR11 | 0.0000 |
|  |  |  |  |  |  |  |  |  |  | A24~B34~DR13 | 0.0000 |
|  |  |  |  |  |  |  |  |  |  | A30~B18~DR09 | 0.0000 |
|  |  |  |  |  |  |  |  |  |  | A68~B51~DR09 | 0.0000 |
|  |  |  |  |  |  |  |  |  |  | A32~B32~DR04 | 0.0000 |
|  |  |  |  |  |  |  |  |  |  | A03~B32~DR11 | 0.0000 |
|  |  |  |  |  |  |  |  |  |  | A03~B32~DR04 | 0.0000 |
|  |  |  |  |  |  |  |  |  |  | A32~B56~DR11 | 0.0000 |
|  |  |  |  |  |  |  |  |  |  | A68~B14~DR11 | 0.0000 |
|  |  |  |  |  |  |  |  |  |  | A32~B14~DR03 | 0.0000 |
|  |  |  |  |  |  |  |  |  |  | A24~B56~DR16 | 0.0000 |
|  |  |  |  |  |  |  |  |  |  | A02~B56~DR16 | 0.0000 |
|  |  |  |  |  |  |  |  |  |  | A26~B37~DR15 | 0.0000 |
|  |  |  |  |  |  |  |  |  |  | A11~B41~DR12 | 0.0000 |
|  |  |  |  |  |  |  |  |  |  | A32~B50~DR01 | 0.0000 |
|  |  |  |  |  |  |  |  |  |  | A33~B50~DR01 | 0.0000 |
|  |  |  |  |  |  |  |  |  |  | A32~B50~DR13 | 0.0000 |
|  |  |  |  |  |  |  |  |  |  | A11~B14~DR12 | 0.0000 |
|  |  |  |  |  |  |  |  |  |  | A24~B14~DR10 | 0.0000 |
|  |  |  |  |  |  |  |  |  |  | A33~B52~DR10 | 0.0000 |
|  |  |  |  |  |  |  |  |  |  | A33~B52~DR03 | 0.0000 |
|  |  |  |  |  |  |  |  |  |  | A23~B52~DR08 | 0.0000 |
|  |  |  |  |  |  |  |  |  |  | A23~B52~DR01 | 0.0000 |
|  |  |  |  |  |  |  |  |  |  | A26~B44~DR09 | 0.0000 |
|  |  |  |  |  |  |  |  |  |  | A24~B39~DR12 | 0.0000 |
|  |  |  |  |  |  |  |  |  |  | A29~B45~DR16 | 0.0000 |
|  |  |  |  |  |  |  |  |  |  | A03~B45~DR16 | 0.0000 |
|  |  |  |  |  |  |  |  |  |  | A32~B57~DR11 | 0.0000 |
|  |  |  |  |  |  |  |  |  |  | A01~B49~DR09 | 0.0000 |
|  |  |  |  |  |  |  |  |  |  | A25~B51~DR14 | 0.0000 |
|  |  |  |  |  |  |  |  |  |  | A26~B55~DR16 | 0.0000 |
|  |  |  |  |  |  |  |  |  |  | A26~B18~DR08 | 0.0000 |
|  |  |  |  |  |  |  |  |  |  | A24~B57~DR01 | 0.0000 |
|  |  |  |  |  |  |  |  |  |  | A11~B39~DR01 | 0.0000 |
|  |  |  |  |  |  |  |  |  |  | A26~B52~DR01 | 0.0000 |
|  |  |  |  |  |  |  |  |  |  | A26~B52~DR07 | 0.0000 |
|  |  |  |  |  |  |  |  |  |  | A26~B57~DR14 | 0.0000 |
|  |  |  |  |  |  |  |  |  |  | A30~B55~DR11 | 0.0000 |
|  |  |  |  |  |  |  |  |  |  | A29~B27~DR10 | 0.0000 |
|  |  |  |  |  |  |  |  |  |  | A32~B52~DR10 | 0.0000 |
|  |  |  |  |  |  |  |  |  |  | A26~B49~DR10 | 0.0000 |
|  |  |  |  |  |  |  |  |  |  | A33~B58~DR08 | 0.0000 |
|  |  |  |  |  |  |  |  |  |  | A33~B40~DR08 | 0.0000 |
|  |  |  |  |  |  |  |  |  |  | A33~B40~DR03 | 0.0000 |
|  |  |  |  |  |  |  |  |  |  | A01~B58~DR14 | 0.0000 |
|  |  |  |  |  |  |  |  |  |  | A03~B47~DR10 | 0.0000 |
|  |  |  |  |  |  |  |  |  |  | A03~B47~DR14 | 0.0000 |
|  |  |  |  |  |  |  |  |  |  | A01~B47~DR14 | 0.0000 |
|  |  |  |  |  |  |  |  |  |  | A26~B40~DR03 | 0.0000 |
|  |  |  |  |  |  |  |  |  |  | A68~B50~DR13 | 0.0000 |
|  |  |  |  |  |  |  |  |  |  | A26~B38~DR12 | 0.0000 |
|  |  |  |  |  |  |  |  |  |  | A32~B52~DR01 | 0.0000 |
|  |  |  |  |  |  |  |  |  |  | A02~B45~DR15 | 0.0000 |
|  |  |  |  |  |  |  |  |  |  | A11~B45~DR13 | 0.0000 |
|  |  |  |  |  |  |  |  |  |  | A11~B45~DR11 | 0.0000 |
|  |  |  |  |  |  |  |  |  |  | A25~B55~DR04 | 0.0000 |
|  |  |  |  |  |  |  |  |  |  | A69~B46~DR12 | 0.0000 |
|  |  |  |  |  |  |  |  |  |  | A69~B18~DR12 | 0.0000 |
|  |  |  |  |  |  |  |  |  |  | A02~B46~DR12 | 0.0000 |
|  |  |  |  |  |  |  |  |  |  | A69~B18~DR13 | 0.0000 |
|  |  |  |  |  |  |  |  |  |  | A25~B13~DR15 | 0.0000 |
|  |  |  |  |  |  |  |  |  |  | A25~B50~DR15 | 0.0000 |
|  |  |  |  |  |  |  |  |  |  | A03~B58~DR15 | 0.0000 |
|  |  |  |  |  |  |  |  |  |  | A32~B53~DR04 | 0.0000 |
|  |  |  |  |  |  |  |  |  |  | A03~B53~DR04 | 0.0000 |
|  |  |  |  |  |  |  |  |  |  | A23~B39~DR15 | 0.0000 |
|  |  |  |  |  |  |  |  |  |  | A23~B39~DR04 | 0.0000 |
|  |  |  |  |  |  |  |  |  |  | A32~B40~DR08 | 0.0000 |
|  |  |  |  |  |  |  |  |  |  | A33~B15~DR11 | 0.0000 |
|  |  |  |  |  |  |  |  |  |  | A32~B58~DR07 | 0.0000 |
|  |  |  |  |  |  |  |  |  |  | A32~B58~DR11 | 0.0000 |
|  |  |  |  |  |  |  |  |  |  | A32~B37~DR07 | 0.0000 |
|  |  |  |  |  |  |  |  |  |  | A11~B39~DR12 | 0.0000 |
|  |  |  |  |  |  |  |  |  |  | A11~B27~DR12 | 0.0000 |
|  |  |  |  |  |  |  |  |  |  | A02~B45~DR03 | 0.0000 |
|  |  |  |  |  |  |  |  |  |  | A02~B45~DR16 | 0.0000 |
|  |  |  |  |  |  |  |  |  |  | A01~B45~DR16 | 0.0000 |
|  |  |  |  |  |  |  |  |  |  | A25~B55~DR15 | 0.0000 |
|  |  |  |  |  |  |  |  |  |  | A25~B13~DR12 | 0.0000 |
|  |  |  |  |  |  |  |  |  |  | A25~B35~DR12 | 0.0000 |
|  |  |  |  |  |  |  |  |  |  | A02~B13~DR12 | 0.0000 |
|  |  |  |  |  |  |  |  |  |  | A25~B07~DR01 | 0.0000 |
|  |  |  |  |  |  |  |  |  |  | A31~B37~DR04 | 0.0000 |
|  |  |  |  |  |  |  |  |  |  | A31~B37~DR16 | 0.0000 |
|  |  |  |  |  |  |  |  |  |  | A31~B27~DR08 | 0.0000 |
|  |  |  |  |  |  |  |  |  |  | A02~B54~DR03 | 0.0000 |
|  |  |  |  |  |  |  |  |  |  | A23~B53~DR03 | 0.0000 |
|  |  |  |  |  |  |  |  |  |  | A23~B51~DR07 | 0.0000 |
|  |  |  |  |  |  |  |  |  |  | A66~B44~DR03 | 0.0000 |
|  |  |  |  |  |  |  |  |  |  | A66~B44~DR13 | 0.0000 |
|  |  |  |  |  |  |  |  |  |  | A23~B57~DR07 | 0.0000 |
|  |  |  |  |  |  |  |  |  |  | A23~B08~DR01 | 0.0000 |
|  |  |  |  |  |  |  |  |  |  | A26~B41~DR16 | 0.0000 |
|  |  |  |  |  |  |  |  |  |  | A29~B38~DR16 | 0.0000 |
|  |  |  |  |  |  |  |  |  |  | A26~B14~DR16 | 0.0000 |
|  |  |  |  |  |  |  |  |  |  | A30~B38~DR01 | 0.0000 |
|  |  |  |  |  |  |  |  |  |  | A30~B15~DR01 | 0.0000 |
|  |  |  |  |  |  |  |  |  |  | A11~B38~DR16 | 0.0000 |
|  |  |  |  |  |  |  |  |  |  | A23~B38~DR16 | 0.0000 |
|  |  |  |  |  |  |  |  |  |  | A26~B40~DR01 | 0.0000 |
|  |  |  |  |  |  |  |  |  |  | A32~B57~DR01 | 0.0000 |
|  |  |  |  |  |  |  |  |  |  | A26~B51~DR12 | 0.0000 |
|  |  |  |  |  |  |  |  |  |  | A02~B37~DR09 | 0.0000 |
|  |  |  |  |  |  |  |  |  |  | A32~B37~DR04 | 0.0000 |
|  |  |  |  |  |  |  |  |  |  | A30~B58~DR13 | 0.0000 |
|  |  |  |  |  |  |  |  |  |  | A66~B52~DR04 | 0.0000 |
|  |  |  |  |  |  |  |  |  |  | A66~B57~DR15 | 0.0000 |
|  |  |  |  |  |  |  |  |  |  | A66~B13~DR15 | 0.0000 |
|  |  |  |  |  |  |  |  |  |  | A66~B18~DR07 | 0.0000 |
|  |  |  |  |  |  |  |  |  |  | A25~B52~DR07 | 0.0000 |
|  |  |  |  |  |  |  |  |  |  | A01~B39~DR07 | 0.0000 |
|  |  |  |  |  |  |  |  |  |  | A01~B48~DR07 | 0.0000 |
|  |  |  |  |  |  |  |  |  |  | A29~B18~DR03 | 0.0000 |
|  |  |  |  |  |  |  |  |  |  | A31~B08~DR13 | 0.0000 |
|  |  |  |  |  |  |  |  |  |  | A31~B14~DR03 | 0.0000 |
|  |  |  |  |  |  |  |  |  |  | A68~B41~DR04 | 0.0000 |
|  |  |  |  |  |  |  |  |  |  | A68~B27~DR03 | 0.0000 |
|  |  |  |  |  |  |  |  |  |  | A03~B39~DR03 | 0.0000 |
|  |  |  |  |  |  |  |  |  |  | A33~B41~DR10 | 0.0000 |
|  |  |  |  |  |  |  |  |  |  | A23~B08~DR08 | 0.0000 |
|  |  |  |  |  |  |  |  |  |  | A66~B08~DR01 | 0.0000 |
|  |  |  |  |  |  |  |  |  |  | A66~B08~DR14 | 0.0000 |
|  |  |  |  |  |  |  |  |  |  | A66~B13~DR01 | 0.0000 |
|  |  |  |  |  |  |  |  |  |  | A02~B56~DR11 | 0.0000 |
|  |  |  |  |  |  |  |  |  |  | A29~B39~DR16 | 0.0000 |
|  |  |  |  |  |  |  |  |  |  | A29~B52~DR08 | 0.0000 |
|  |  |  |  |  |  |  |  |  |  | A29~B52~DR07 | 0.0000 |
|  |  |  |  |  |  |  |  |  |  | A69~B13~DR14 | 0.0000 |
|  |  |  |  |  |  |  |  |  |  | A01~B13~DR16 | 0.0000 |
|  |  |  |  |  |  |  |  |  |  | A29~B58~DR03 | 0.0000 |
|  |  |  |  |  |  |  |  |  |  | A32~B83~DR04 | 0.0000 |
|  |  |  |  |  |  |  |  |  |  | A03~B83~DR13 | 0.0000 |
|  |  |  |  |  |  |  |  |  |  | A03~B83~DR04 | 0.0000 |
|  |  |  |  |  |  |  |  |  |  | A23~B44~DR12 | 0.0000 |
|  |  |  |  |  |  |  |  |  |  | A23~B27~DR12 | 0.0000 |
|  |  |  |  |  |  |  |  |  |  | A33~B50~DR15 | 0.0000 |
|  |  |  |  |  |  |  |  |  |  | A30~B50~DR12 | 0.0000 |
|  |  |  |  |  |  |  |  |  |  | A25~B49~DR07 | 0.0000 |
|  |  |  |  |  |  |  |  |  |  | A25~B49~DR13 | 0.0000 |
|  |  |  |  |  |  |  |  |  |  | A02~B57~DR08 | 0.0000 |
|  |  |  |  |  |  |  |  |  |  | A03~B57~DR08 | 0.0000 |
|  |  |  |  |  |  |  |  |  |  | A66~B49~DR10 | 0.0000 |
|  |  |  |  |  |  |  |  |  |  | A66~B49~DR07 | 0.0000 |
|  |  |  |  |  |  |  |  |  |  | A33~B27~DR03 | 0.0000 |
|  |  |  |  |  |  |  |  |  |  | A25~B15~DR11 | 0.0000 |
|  |  |  |  |  |  |  |  |  |  | A02~B58~DR10 | 0.0000 |
|  |  |  |  |  |  |  |  |  |  | A33~B41~DR07 | 0.0000 |
|  |  |  |  |  |  |  |  |  |  | A33~B52~DR07 | 0.0000 |
|  |  |  |  |  |  |  |  |  |  | A26~B49~DR16 | 0.0000 |
|  |  |  |  |  |  |  |  |  |  | A26~B49~DR07 | 0.0000 |
|  |  |  |  |  |  |  |  |  |  | A24~B38~DR12 | 0.0000 |
|  |  |  |  |  |  |  |  |  |  | A03~B15~DR12 | 0.0000 |
|  |  |  |  |  |  |  |  |  |  | A03~B38~DR12 | 0.0000 |
|  |  |  |  |  |  |  |  |  |  | A24~B53~DR03 | 0.0000 |
|  |  |  |  |  |  |  |  |  |  | A30~B53~DR14 | 0.0000 |
|  |  |  |  |  |  |  |  |  |  | A03~B14~DR08 | 0.0000 |
|  |  |  |  |  |  |  |  |  |  | A68~B58~DR04 | 0.0000 |
|  |  |  |  |  |  |  |  |  |  | A68~B58~DR03 | 0.0000 |
|  |  |  |  |  |  |  |  |  |  | A03~B14~DR12 | 0.0000 |
|  |  |  |  |  |  |  |  |  |  | A66~B57~DR16 | 0.0000 |
|  |  |  |  |  |  |  |  |  |  | A66~B57~DR09 | 0.0000 |
|  |  |  |  |  |  |  |  |  |  | A32~B57~DR09 | 0.0000 |
|  |  |  |  |  |  |  |  |  |  | A24~B66~DR09 | 0.0000 |
|  |  |  |  |  |  |  |  |  |  | A24~B66~DR14 | 0.0000 |
|  |  |  |  |  |  |  |  |  |  | A02~B66~DR14 | 0.0000 |
|  |  |  |  |  |  |  |  |  |  | A32~B08~DR16 | 0.0000 |
|  |  |  |  |  |  |  |  |  |  | A32~B57~DR03 | 0.0000 |
|  |  |  |  |  |  |  |  |  |  | A66~B44~DR09 | 0.0000 |
|  |  |  |  |  |  |  |  |  |  | A66~B44~DR04 | 0.0000 |
|  |  |  |  |  |  |  |  |  |  | A66~B46~DR04 | 0.0000 |
|  |  |  |  |  |  |  |  |  |  | A02~B46~DR04 | 0.0000 |
|  |  |  |  |  |  |  |  |  |  | A33~B35~DR09 | 0.0000 |
|  |  |  |  |  |  |  |  |  |  | A66~B14~DR09 | 0.0000 |
|  |  |  |  |  |  |  |  |  |  | A32~B52~DR12 | 0.0000 |
|  |  |  |  |  |  |  |  |  |  | A32~B40~DR12 | 0.0000 |
|  |  |  |  |  |  |  |  |  |  | A24~B52~DR12 | 0.0000 |
|  |  |  |  |  |  |  |  |  |  | A25~B50~DR01 | 0.0000 |
|  |  |  |  |  |  |  |  |  |  | A25~B51~DR03 | 0.0000 |
|  |  |  |  |  |  |  |  |  |  | A32~B58~DR13 | 0.0000 |
|  |  |  |  |  |  |  |  |  |  | A32~B27~DR10 | 0.0000 |
|  |  |  |  |  |  |  |  |  |  | A32~B32~DR03 | 0.0000 |
|  |  |  |  |  |  |  |  |  |  | A24~B32~DR11 | 0.0000 |
|  |  |  |  |  |  |  |  |  |  | A24~B32~DR03 | 0.0000 |
|  |  |  |  |  |  |  |  |  |  | A32~B49~DR07 | 0.0000 |
|  |  |  |  |  |  |  |  |  |  | A25~B38~DR11 | 0.0000 |
|  |  |  |  |  |  |  |  |  |  | A32~B58~DR14 | 0.0000 |
|  |  |  |  |  |  |  |  |  |  | A23~B58~DR11 | 0.0000 |
|  |  |  |  |  |  |  |  |  |  | A23~B58~DR01 | 0.0000 |
|  |  |  |  |  |  |  |  |  |  | A26~B37~DR04 | 0.0000 |
|  |  |  |  |  |  |  |  |  |  | A33~B18~DR03 | 0.0000 |
|  |  |  |  |  |  |  |  |  |  | A26~B38~DR09 | 0.0000 |
|  |  |  |  |  |  |  |  |  |  | A69~B52~DR11 | 0.0000 |
|  |  |  |  |  |  |  |  |  |  | A03~B54~DR13 | 0.0000 |
|  |  |  |  |  |  |  |  |  |  | A26~B52~DR08 | 0.0000 |
|  |  |  |  |  |  |  |  |  |  | A69~B55~DR01 | 0.0000 |
|  |  |  |  |  |  |  |  |  |  | A69~B51~DR08 | 0.0000 |
|  |  |  |  |  |  |  |  |  |  | A69~B51~DR01 | 0.0000 |
|  |  |  |  |  |  |  |  |  |  | A69~B41~DR15 | 0.0000 |
|  |  |  |  |  |  |  |  |  |  | A69~B41~DR04 | 0.0000 |
|  |  |  |  |  |  |  |  |  |  | A31~B27~DR13 | 0.0000 |
|  |  |  |  |  |  |  |  |  |  | A31~B27~DR10 | 0.0000 |
|  |  |  |  |  |  |  |  |  |  | A11~B18~DR10 | 0.0000 |
|  |  |  |  |  |  |  |  |  |  | A01~B38~DR08 | 0.0000 |
|  |  |  |  |  |  |  |  |  |  | A33~B38~DR08 | 0.0000 |
|  |  |  |  |  |  |  |  |  |  | A01~B73~DR08 | 0.0000 |
|  |  |  |  |  |  |  |  |  |  | A23~B15~DR01 | 0.0000 |
|  |  |  |  |  |  |  |  |  |  | A23~B56~DR11 | 0.0000 |
|  |  |  |  |  |  |  |  |  |  | A26~B27~DR03 | 0.0000 |
|  |  |  |  |  |  |  |  |  |  | A30~B14~DR03 | 0.0000 |
|  |  |  |  |  |  |  |  |  |  | A30~B14~DR13 | 0.0000 |
|  |  |  |  |  |  |  |  |  |  | A66~B08~DR13 | 0.0000 |
|  |  |  |  |  |  |  |  |  |  | A68~B08~DR14 | 0.0000 |
|  |  |  |  |  |  |  |  |  |  | A68~B08~DR09 | 0.0000 |
|  |  |  |  |  |  |  |  |  |  | A01~B55~DR09 | 0.0000 |
|  |  |  |  |  |  |  |  |  |  | A33~B57~DR01 | 0.0000 |
|  |  |  |  |  |  |  |  |  |  | A33~B57~DR13 | 0.0000 |
|  |  |  |  |  |  |  |  |  |  | A25~B50~DR11 | 0.0000 |
|  |  |  |  |  |  |  |  |  |  | A25~B40~DR07 | 0.0000 |
|  |  |  |  |  |  |  |  |  |  | A31~B57~DR04 | 0.0000 |
|  |  |  |  |  |  |  |  |  |  | A31~B57~DR13 | 0.0000 |
|  |  |  |  |  |  |  |  |  |  | A02~B48~DR07 | 0.0000 |
|  |  |  |  |  |  |  |  |  |  | A31~B27~DR03 | 0.0000 |
|  |  |  |  |  |  |  |  |  |  | A29~B55~DR01 | 0.0000 |
|  |  |  |  |  |  |  |  |  |  | A31~B55~DR13 | 0.0000 |
|  |  |  |  |  |  |  |  |  |  | A68~B08~DR07 | 0.0000 |
|  |  |  |  |  |  |  |  |  |  | A26~B50~DR09 | 0.0000 |
|  |  |  |  |  |  |  |  |  |  | A03~B50~DR09 | 0.0000 |
|  |  |  |  |  |  |  |  |  |  | A11~B18~DR12 | 0.0000 |
|  |  |  |  |  |  |  |  |  |  | A24~B13~DR09 | 0.0000 |
|  |  |  |  |  |  |  |  |  |  | A02~B13~DR09 | 0.0000 |
|  |  |  |  |  |  |  |  |  |  | A32~B57~DR13 | 0.0000 |
|  |  |  |  |  |  |  |  |  |  | A69~B57~DR13 | 0.0000 |
|  |  |  |  |  |  |  |  |  |  | A69~B07~DR13 | 0.0000 |
|  |  |  |  |  |  |  |  |  |  | A69~B58~DR03 | 0.0000 |
|  |  |  |  |  |  |  |  |  |  | A69~B58~DR07 | 0.0000 |
|  |  |  |  |  |  |  |  |  |  | A69~B57~DR03 | 0.0000 |
|  |  |  |  |  |  |  |  |  |  | A68~B41~DR11 | 0.0000 |
|  |  |  |  |  |  |  |  |  |  | A23~B37~DR04 | 0.0000 |
|  |  |  |  |  |  |  |  |  |  | A33~B47~DR08 | 0.0000 |
|  |  |  |  |  |  |  |  |  |  | A66~B14~DR08 | 0.0000 |
|  |  |  |  |  |  |  |  |  |  | A33~B47~DR13 | 0.0000 |
|  |  |  |  |  |  |  |  |  |  | A66~B47~DR13 | 0.0000 |
|  |  |  |  |  |  |  |  |  |  | A02~B48~DR08 | 0.0000 |
|  |  |  |  |  |  |  |  |  |  | A02~B41~DR08 | 0.0000 |
|  |  |  |  |  |  |  |  |  |  | A29~B53~DR11 | 0.0000 |
|  |  |  |  |  |  |  |  |  |  | A32~B45~DR13 | 0.0000 |
|  |  |  |  |  |  |  |  |  |  | A29~B53~DR13 | 0.0000 |
|  |  |  |  |  |  |  |  |  |  | A32~B45~DR11 | 0.0000 |
|  |  |  |  |  |  |  |  |  |  | A29~B45~DR13 | 0.0000 |
|  |  |  |  |  |  |  |  |  |  | A66~B50~DR13 | 0.0000 |
|  |  |  |  |  |  |  |  |  |  | A66~B50~DR15 | 0.0000 |
|  |  |  |  |  |  |  |  |  |  | A68~B50~DR14 | 0.0000 |
|  |  |  |  |  |  |  |  |  |  | A68~B15~DR14 | 0.0000 |
|  |  |  |  |  |  |  |  |  |  | A03~B08~DR09 | 0.0000 |
|  |  |  |  |  |  |  |  |  |  | A24~B27~DR09 | 0.0000 |
|  |  |  |  |  |  |  |  |  |  | A01~B37~DR09 | 0.0000 |
|  |  |  |  |  |  |  |  |  |  | A24~B45~DR04 | 0.0000 |
|  |  |  |  |  |  |  |  |  |  | A26~B27~DR09 | 0.0000 |
|  |  |  |  |  |  |  |  |  |  | A01~B50~DR09 | 0.0000 |
|  |  |  |  |  |  |  |  |  |  | A11~B37~DR07 | 0.0000 |
|  |  |  |  |  |  |  |  |  |  | A31~B38~DR07 | 0.0000 |
|  |  |  |  |  |  |  |  |  |  | A31~B55~DR07 | 0.0000 |
|  |  |  |  |  |  |  |  |  |  | A11~B73~DR15 | 0.0000 |
|  |  |  |  |  |  |  |  |  |  | A02~B73~DR15 | 0.0000 |
|  |  |  |  |  |  |  |  |  |  | A23~B58~DR14 | 0.0000 |
|  |  |  |  |  |  |  |  |  |  | A32~B41~DR15 | 0.0000 |
|  |  |  |  |  |  |  |  |  |  | A02~B73~DR11 | 0.0000 |
|  |  |  |  |  |  |  |  |  |  | A31~B27~DR11 | 0.0000 |
|  |  |  |  |  |  |  |  |  |  | A31~B39~DR16 | 0.0000 |
|  |  |  |  |  |  |  |  |  |  | A31~B39~DR11 | 0.0000 |
|  |  |  |  |  |  |  |  |  |  | A68~B50~DR01 | 0.0000 |
|  |  |  |  |  |  |  |  |  |  | A24~B14~DR09 | 0.0000 |
|  |  |  |  |  |  |  |  |  |  | A26~B52~DR09 | 0.0000 |
|  |  |  |  |  |  |  |  |  |  | A03~B41~DR09 | 0.0000 |
|  |  |  |  |  |  |  |  |  |  | A30~B27~DR16 | 0.0000 |
|  |  |  |  |  |  |  |  |  |  | A31~B38~DR03 | 0.0000 |
|  |  |  |  |  |  |  |  |  |  | A69~B18~DR16 | 0.0000 |
|  |  |  |  |  |  |  |  |  |  | A69~B50~DR16 | 0.0000 |
|  |  |  |  |  |  |  |  |  |  | A01~B50~DR16 | 0.0000 |
|  |  |  |  |  |  |  |  |  |  | A69~B51~DR07 | 0.0000 |
|  |  |  |  |  |  |  |  |  |  | A29~B44~DR01 | 0.0000 |
|  |  |  |  |  |  |  |  |  |  | A29~B35~DR12 | 0.0000 |
|  |  |  |  |  |  |  |  |  |  | A02~B46~DR11 | 0.0000 |
|  |  |  |  |  |  |  |  |  |  | A01~B46~DR11 | 0.0000 |
|  |  |  |  |  |  |  |  |  |  | A01~B46~DR09 | 0.0000 |
|  |  |  |  |  |  |  |  |  |  | A03~B46~DR09 | 0.0000 |
|  |  |  |  |  |  |  |  |  |  | A03~B46~DR14 | 0.0000 |
|  |  |  |  |  |  |  |  |  |  | A29~B52~DR10 | 0.0000 |
|  |  |  |  |  |  |  |  |  |  | A32~B39~DR03 | 0.0000 |
|  |  |  |  |  |  |  |  |  |  | A68~B27~DR07 | 0.0000 |
|  |  |  |  |  |  |  |  |  |  | A03~B52~DR10 | 0.0000 |
|  |  |  |  |  |  |  |  |  |  | A03~B14~DR10 | 0.0000 |
|  |  |  |  |  |  |  |  |  |  | A23~B40~DR03 | 0.0000 |
|  |  |  |  |  |  |  |  |  |  | A25~B52~DR11 | 0.0000 |
|  |  |  |  |  |  |  |  |  |  | A26~B37~DR14 | 0.0000 |
|  |  |  |  |  |  |  |  |  |  | A69~B58~DR16 | 0.0000 |
|  |  |  |  |  |  |  |  |  |  | A69~B44~DR08 | 0.0000 |
|  |  |  |  |  |  |  |  |  |  | A69~B44~DR16 | 0.0000 |
|  |  |  |  |  |  |  |  |  |  | A25~B27~DR03 | 0.0000 |
|  |  |  |  |  |  |  |  |  |  | A30~B55~DR08 | 0.0000 |
|  |  |  |  |  |  |  |  |  |  | A23~B14~DR07 | 0.0000 |
|  |  |  |  |  |  |  |  |  |  | A01~B53~DR03 | 0.0000 |
|  |  |  |  |  |  |  |  |  |  | A30~B08~DR15 | 0.0000 |
|  |  |  |  |  |  |  |  |  |  | A32~B15~DR08 | 0.0000 |
|  |  |  |  |  |  |  |  |  |  | A23~B07~DR13 | 0.0000 |
|  |  |  |  |  |  |  |  |  |  | A24~B46~DR08 | 0.0000 |
|  |  |  |  |  |  |  |  |  |  | A24~B46~DR04 | 0.0000 |
|  |  |  |  |  |  |  |  |  |  | A11~B37~DR16 | 0.0000 |
|  |  |  |  |  |  |  |  |  |  | A26~B39~DR07 | 0.0000 |
|  |  |  |  |  |  |  |  |  |  | A11~B39~DR09 | 0.0000 |
|  |  |  |  |  |  |  |  |  |  | A01~B38~DR12 | 0.0000 |
|  |  |  |  |  |  |  |  |  |  | A68~B07~DR12 | 0.0000 |
|  |  |  |  |  |  |  |  |  |  | A68~B14~DR15 | 0.0000 |
|  |  |  |  |  |  |  |  |  |  | A33~B13~DR14 | 0.0000 |
|  |  |  |  |  |  |  |  |  |  | A32~B46~DR08 | 0.0000 |
|  |  |  |  |  |  |  |  |  |  | A32~B46~DR14 | 0.0000 |
|  |  |  |  |  |  |  |  |  |  | A33~B58~DR10 | 0.0000 |
|  |  |  |  |  |  |  |  |  |  | A74~B07~DR15 | 0.0000 |
|  |  |  |  |  |  |  |  |  |  | A11~B52~DR10 | 0.0000 |
|  |  |  |  |  |  |  |  |  |  | A31~B41~DR16 | 0.0000 |
|  |  |  |  |  |  |  |  |  |  | A31~B55~DR12 | 0.0000 |
|  |  |  |  |  |  |  |  |  |  | A31~B08~DR16 | 0.0000 |
|  |  |  |  |  |  |  |  |  |  | A23~B14~DR14 | 0.0000 |
|  |  |  |  |  |  |  |  |  |  | A23~B04~DR11 | 0.0000 |
|  |  |  |  |  |  |  |  |  |  | A03~B04~DR11 | 0.0000 |
|  |  |  |  |  |  |  |  |  |  | A03~B04~DR10 | 0.0000 |
|  |  |  |  |  |  |  |  |  |  | A33~B27~DR11 | 0.0000 |
|  |  |  |  |  |  |  |  |  |  | A69~B07~DR03 | 0.0000 |
|  |  |  |  |  |  |  |  |  |  | A24~B53~DR01 | 0.0000 |
|  |  |  |  |  |  |  |  |  |  | A33~B53~DR13 | 0.0000 |
|  |  |  |  |  |  |  |  |  |  | A32~B14~DR08 | 0.0000 |
|  |  |  |  |  |  |  |  |  |  | A11~B14~DR08 | 0.0000 |
|  |  |  |  |  |  |  |  |  |  | A11~B50~DR08 | 0.0000 |
|  |  |  |  |  |  |  |  |  |  | A33~B15~DR14 | 0.0000 |
|  |  |  |  |  |  |  |  |  |  | A33~B39~DR07 | 0.0000 |
|  |  |  |  |  |  |  |  |  |  | A33~B13~DR08 | 0.0000 |
|  |  |  |  |  |  |  |  |  |  | A24~B55~DR12 | 0.0000 |
|  |  |  |  |  |  |  |  |  |  | A24~B14~DR12 | 0.0000 |
|  |  |  |  |  |  |  |  |  |  | A25~B44~DR12 | 0.0000 |
|  |  |  |  |  |  |  |  |  |  | A01~B07~DR02 | 0.0000 |
|  |  |  |  |  |  |  |  |  |  | A69~B38~DR07 | 0.0000 |
|  |  |  |  |  |  |  |  |  |  | A69~B49~DR15 | 0.0000 |
|  |  |  |  |  |  |  |  |  |  | A69~B49~DR11 | 0.0000 |
|  |  |  |  |  |  |  |  |  |  | A68~B52~DR07 | 0.0000 |
|  |  |  |  |  |  |  |  |  |  | A33~B39~DR11 | 0.0000 |
|  |  |  |  |  |  |  |  |  |  | A66~B07~DR10 | 0.0000 |
|  |  |  |  |  |  |  |  |  |  | A23~B39~DR01 | 0.0000 |
|  |  |  |  |  |  |  |  |  |  | A23~B39~DR13 | 0.0000 |
|  |  |  |  |  |  |  |  |  |  | A66~B44~DR01 | 0.0000 |
|  |  |  |  |  |  |  |  |  |  | A31~B14~DR11 | 0.0000 |
|  |  |  |  |  |  |  |  |  |  | A24~B50~DR10 | 0.0000 |
|  |  |  |  |  |  |  |  |  |  | A68~B46~DR08 | 0.0000 |
|  |  |  |  |  |  |  |  |  |  | A68~B46~DR14 | 0.0000 |
|  |  |  |  |  |  |  |  |  |  | A02~B46~DR03 | 0.0000 |
|  |  |  |  |  |  |  |  |  |  | A29~B07~DR14 | 0.0000 |
|  |  |  |  |  |  |  |  |  |  | A32~B58~DR16 | 0.0000 |
|  |  |  |  |  |  |  |  |  |  | A01~B14~DR15 | 0.0000 |
|  |  |  |  |  |  |  |  |  |  | A29~B41~DR13 | 0.0000 |
|  |  |  |  |  |  |  |  |  |  | A26~B13~DR11 | 0.0000 |
|  |  |  |  |  |  |  |  |  |  | A68~B38~DR16 | 0.0000 |
|  |  |  |  |  |  |  |  |  |  | A31~B40~DR10 | 0.0000 |
|  |  |  |  |  |  |  |  |  |  | A11~B56~DR11 | 0.0000 |
|  |  |  |  |  |  |  |  |  |  | A32~B50~DR14 | 0.0000 |
|  |  |  |  |  |  |  |  |  |  | A11~B18~DR08 | 0.0000 |
|  |  |  |  |  |  |  |  |  |  | A33~B51~DR07 | 0.0000 |
|  |  |  |  |  |  |  |  |  |  | A33~B57~DR15 | 0.0000 |
|  |  |  |  |  |  |  |  |  |  | A33~B57~DR07 | 0.0000 |
|  |  |  |  |  |  |  |  |  |  | A33~B49~DR07 | 0.0000 |
|  |  |  |  |  |  |  |  |  |  | A33~B57~DR03 | 0.0000 |
|  |  |  |  |  |  |  |  |  |  | A24~B55~DR09 | 0.0000 |
|  |  |  |  |  |  |  |  |  |  | A29~B52~DR01 | 0.0000 |
|  |  |  |  |  |  |  |  |  |  | A24~B58~DR01 | 0.0000 |
|  |  |  |  |  |  |  |  |  |  | A29~B45~DR04 | 0.0000 |
|  |  |  |  |  |  |  |  |  |  | A68~B15~DR07 | 0.0000 |
|  |  |  |  |  |  |  |  |  |  | A01~B45~DR07 | 0.0000 |
|  |  |  |  |  |  |  |  |  |  | A01~B39~DR14 | 0.0000 |
|  |  |  |  |  |  |  |  |  |  | A66~B18~DR01 | 0.0000 |
|  |  |  |  |  |  |  |  |  |  | A66~B49~DR04 | 0.0000 |
|  |  |  |  |  |  |  |  |  |  | A66~B38~DR01 | 0.0000 |
|  |  |  |  |  |  |  |  |  |  | A66~B38~DR04 | 0.0000 |
|  |  |  |  |  |  |  |  |  |  | A33~B13~DR13 | 0.0000 |
|  |  |  |  |  |  |  |  |  |  | A23~B18~DR13 | 0.0000 |
|  |  |  |  |  |  |  |  |  |  | A33~B07~DR10 | 0.0000 |
|  |  |  |  |  |  |  |  |  |  | A29~B14~DR10 | 0.0000 |
|  |  |  |  |  |  |  |  |  |  | A30~B37~DR03 | 0.0000 |
|  |  |  |  |  |  |  |  |  |  | A30~B37~DR01 | 0.0000 |
|  |  |  |  |  |  |  |  |  |  | A31~B13~DR01 | 0.0000 |
|  |  |  |  |  |  |  |  |  |  | A24~B45~DR15 | 0.0000 |
|  |  |  |  |  |  |  |  |  |  | A01~B39~DR12 | 0.0000 |
|  |  |  |  |  |  |  |  |  |  | A29~B27~DR15 | 0.0000 |
|  |  |  |  |  |  |  |  |  |  | A11~B50~DR12 | 0.0000 |
|  |  |  |  |  |  |  |  |  |  | sum: | 10.033 |

**Sheet 3.** HSCT candidates haplotype.

| **Name** | **Frequency** | **Name** | **Frequency** | **Name** | **Frequency** | **Name** | **Frequency** | **Name** | **Frequency** | **Name** | **Frequency** |
| --- | --- | --- | --- | --- | --- | --- | --- | --- | --- | --- | --- |
| A24~B35~C04 | 0.04436 | A24~B35~C04~DR11 | 0.01888 | A24~B35~C04~DR11~DQ03 | 0.03138 | A24~B35~C04~DR11~DQ03~DP04 | 0.017 | A24~B35~DR11 | 0.01979 | DR11~DQ03~DP04 | 0.13522 |
| A03~B35~C04 | 0.03037 | A23~B49~C07~DR11 | 0.01471 | A23~B49~C07~DR11~DQ03 | 0.01385 | A02~B35~C04~DR04~DQ03~DP02 | 0.01418 | A03~B35~DR11 | 0.01383 | DR04~DQ03~DP04 | 0.06251 |
| A23~B49~C07 | 0.02489 | A03~B35~C04~DR11 | 0.01373 | A02~B13~C06~DR07~DQ02 | 0.01316 | A23~B49~C07~DR11~DQ03~DP04 | 0.01282 | A02~B51~DR11 | 0.01231 | DR15~DQ06~DP04 | 0.05456 |
| A01~B35~C04 | 0.02118 | A02~B13~C06~DR07 | 0.01109 | A02~B35~C04~DR04~DQ03 | 0.01215 | A02~B51~C15~DR11~DQ03~DP04 | 0.0116 | A02~B35~DR04 | 0.01123 | DR03~DQ02~DP04 | 0.05063 |
| A11~B35~C04 | 0.01951 | A02~B35~C04~DR11 | 0.01108 | A03~B35~C04~DR01~DQ05 | 0.01111 | A11~B52~C12~DR15~DQ06~DP04 | 0.0116 | A23~B49~DR11 | 0.01091 | DR01~DQ05~DP04 | 0.03954 |
| A03~B07~C07 | 0.0189 | A01~B08~C07~DR03 | 0.01028 | A01~B08~C07~DR03~DQ02 | 0.01091 | A02~B13~C06~DR07~DQ02~DP04 | 0.01031 | A02~B50~DR07 | 0.01009 | DR14~DQ05~DP04 | 0.03902 |
| A02~B35~C04 | 0.01752 | A33~B14~C08~DR01 | 0.01024 | A03~B07~C07~DR15~DQ06 | 0.01059 | A02~B08~C07~DR03~DQ02~DP04 | 0.01031 | A33~B14~DR01 | 0.01006 | DR04~DQ03~DP02 | 0.03635 |
| A02~B50~C06 | 0.01531 | A24~B35~C04~DR04 | 0.01024 | A01~B35~C04~DR11~DQ03 | 0.01022 | A02~B50~C06~DR07~DQ02~DP04 | 0.00902 | A03~B07~DR15 | 0.00929 | DR13~DQ06~DP04 | 0.03531 |
| A24~B51~C15 | 0.01467 | A24~B51~C15~DR11 | 0.01004 | A33~B14~C08~DR01~DQ05 | 0.01012 | A24~B35~C04~DR11~DQ03~DP02 | 0.00902 | A02~B51~DR04 | 0.00902 | DR11~DQ03~DP02 | 0.03361 |
| A33~B14~C08 | 0.01346 | A03~B07~C07~DR15 | 0.00928 | A11~B52~C12~DR15~DQ06 | 0.01009 | A02~B35~C04~DR11~DQ03~DP04 | 0.00862 | A02~B44~DR11 | 0.00901 | DR16~DQ05~DP04 | 0.03122 |
| A02~B44~C05 | 0.01338 | A02~B18~C07~DR11 | 0.00872 | A02~B18~C07~DR11~DQ03 | 0.01006 | A26~B08~C07~DR03~DQ02~DP04 | 0.00773 | A01~B08~DR03 | 0.009 | DR13~DQ06~DP02 | 0.02999 |
| A02~B49~C07 | 0.01321 | A02~B50~C06~DR07 | 0.00872 | A26~B08~C07~DR03~DQ02 | 0.00903 | A03~B08~C07~DR03~DQ02~DP04 | 0.00773 | A24~B51~DR11 | 0.00894 | DR07~DQ02~DP04 | 0.02667 |
| A02~B13~C06 | 0.01294 | A24~B35~C04~DR13 | 0.00834 | A02~B50~C06~DR07~DQ02 | 0.00872 | A03~B35~C04~DR13~DQ06~DP04 | 0.00773 | A02~B51~DR13 | 0.00893 | DR15~DQ06~DP02 | 0.01745 |
| A02~B51~C15 | 0.01191 | A11~B52~C12~DR15 | 0.0079 | A03~B35~C04~DR13~DQ06 | 0.0081 | A03~B35~C04~DR04~DQ03~DP04 | 0.00773 | A03~B44~DR04 | 0.00881 | DR07~DQ02~DP17 | 0.01506 |
| A11~B52~C12 | 0.01125 | A26~B08~C07~DR03 | 0.00768 | A02~B51~C15~DR11~DQ03 | 0.00802 | A02~B07~C07~DR15~DQ06~DP04 | 0.00735 | A02~B18~DR11 | 0.00863 | DR07~DQ02~DP02 | 0.0131 |
| A26~B38~C12 | 0.0112 | A03~B35~C04~DR04 | 0.00721 | A03~B08~C07~DR03~DQ02 | 0.00796 | A03~B07~C07~DR15~DQ06~DP04 | 0.00688 | A24~B35~DR04 | 0.00849 | DR11~DQ03~DP03 | 0.0116 |
| A24~B07~C07 | 0.01049 | A01~B35~C04~DR11 | 0.00719 | A24~B35~C04~DR04~DQ03 | 0.00669 | A02~B35~C04~DR13~DQ06~DP02 | 0.00644 | A01~B51~DR11 | 0.0084 | DR13~DQ03~DP04 | 0.01068 |
| A02~B40~C03 | 0.00984 | A24~B18~C12~DR11 | 0.00715 | A03~B07~C07~DR11~DQ03 | 0.0066 | A01~B35~C06~DR11~DQ03~DP04 | 0.00644 | A24~B18~DR11 | 0.00813 | DR04~DQ03~DP03 | 0.0105 |
| A68~B35~C04 | 0.00947 | A03~B35~C04~DR13 | 0.0065 | A24~B07~C07~DR15~DQ06 | 0.00607 | A03~B18~C12~DR11~DQ03~DP04 | 0.00644 | A01~B35~DR11 | 0.00805 | DR14~DQ05~DP02 | 0.01002 |
| A30~B35~C04 | 0.00946 | A03~B08~C07~DR03 | 0.00647 | A26~B38~C12~DR11~DQ03 | 0.00607 | A33~B14~C08~DR01~DQ05~DP04 | 0.00644 | A03~B35~DR04 | 0.00788 | DR03~DQ02~DP02 | 0.00981 |
| A02~B51~C16 | 0.00938 | A02~B44~C05~DR04 | 0.00634 | A03~B35~C04~DR04~DQ03 | 0.00607 | A02~B49~C07~DR01~DQ05~DP04 | 0.00515 | A11~B52~DR15 | 0.00751 | DR10~DQ05~DP04 | 0.00932 |
| A02~B07~C07 | 0.00931 | A02~B51~C16~DR11 | 0.00599 | A02~B51~C16~DR11~DQ03 | 0.00607 | A26~B38~C12~DR03~DQ02~DP04 | 0.00515 | A02~B44~DR04 | 0.00735 | DR08~DQ03~DP04 | 0.0089 |
| A01~B08~C07 | 0.0091 | A11~B35~C04~DR01 | 0.00588 | A02~B44~C05~DR04~DQ03 | 0.00607 | A01~B18~C07~DR04~DQ03~DP04 | 0.00515 | A02~B07~DR15 | 0.00714 | DR03~DQ02~DP03 | 0.00838 |
| A01~B57~C06 | 0.00872 | A24~B07~C07~DR15 | 0.00567 | A03~B18~C12~DR11~DQ03 | 0.00607 | A03~B35~C04~DR01~DQ05~DP04 | 0.00515 | A02~B13~DR07 | 0.00712 | DR08~DQ04~DP04 | 0.00829 |
| A32~B35~C04 | 0.00871 | A30~B35~C04~DR04 | 0.00555 | A02~B35~C04~DR11~DQ03 | 0.00557 | A02~B44~C05~DR11~DQ03~DP04 | 0.00515 | A02~B51~DR14 | 0.00671 | DR11~DQ03~DP14 | 0.008 |
| A02~B38~C12 | 0.00852 | A02~B40~C03~DR13 | 0.00531 | A02~B35~C04~DR14~DQ05 | 0.00557 | A02~B41~C17~DR04~DQ03~DP04 | 0.00509 | A03~B35~DR13 | 0.00638 | DR12~DQ03~DP04 | 0.00799 |
| A30~B13~C06 | 0.00847 | A02~B49~C07~DR13 | 0.00506 | A11~B35~C04~DR14~DQ05 | 0.00506 | A24~B35~C04~DR11~DQ03~DP03 | 0.0043 | A24~B35~DR14 | 0.006 | DR01~DQ05~DP02 | 0.00739 |
| A02~B18~C07 | 0.00839 | A01~B52~C12~DR15 | 0.00469 | A30~B13~C06~DR07~DQ02 | 0.00506 | A02~B18~C12~DR11~DQ03~DP04 | 0.00387 | A32~B35~DR11 | 0.00593 | DR10~DQ05~DP02 | 0.00718 |
| A24~B18~C12 | 0.00831 | A03~B18~C12~DR11 | 0.00468 | A24~B52~C12~DR15~DQ06 | 0.00447 | A02~B51~C14~DR04~DQ03~DP04 | 0.00387 | A11~B35~DR01 | 0.00592 | DR13~DQ06~DP09 | 0.00708 |
| A24~B55~C01 | 0.00794 | A02~B38~C12~DR13 | 0.00452 | A24~B51~C16~DR11~DQ03 | 0.00421 | A01~B57~C06~DR11~DQ03~DP04 | 0.00387 | A03~B51~DR04 | 0.00562 | DR04~DQ04~DP04 | 0.00695 |
| A29~B07~C15 | 0.00792 | A02~B51~C14~DR04 | 0.00448 | A03~B35~C06~DR11~DQ03 | 0.00405 | A23~B49~C07~DR11~DQ03~DP02 | 0.00387 | A26~B38~DR11 | 0.00545 | DR11~DQ03~DP13 | 0.00694 |
| A01~B37~C06 | 0.00789 | A02~B35~C04~DR04 | 0.00442 | A01~B35~C06~DR07~DQ02 | 0.00405 | A24~B51~C16~DR11~DQ03~DP04 | 0.00387 | A26~B08~DR03 | 0.00541 | DR12~DQ03~DP02 | 0.00664 |
| A26~B08~C07 | 0.00784 | A24~B07~C07~DR04 | 0.00436 | A02~B35~C04~DR13~DQ06 | 0.00405 | A02~B51~C16~DR11~DQ03~DP04 | 0.00387 | A24~B51~DR04 | 0.00525 | DR11~DQ03~DP10 | 0.00647 |
| A02~B15~C03 | 0.00781 | A03~B07~C07~DR11 | 0.00435 | A02~B44~C16~DR04~DQ03 | 0.00405 | A24~B50~C06~DR11~DQ03~DP04 | 0.00387 | A25~B18~DR15 | 0.00504 | DR07~DQ02~DP03 | 0.00641 |
| A24~B18~C07 | 0.00732 | A11~B35~C04~DR15 | 0.00412 | A23~B49~C07~DR07~DQ03 | 0.00405 | A24~B18~C07~DR03~DQ02~DP03 | 0.00387 | A02~B35~DR08 | 0.00501 | DR16~DQ05~DP02 | 0.00573 |
| A03~B08~C07 | 0.00711 | A24~B18~C07~DR13 | 0.00396 | A11~B51~C15~DR04~DQ03 | 0.00405 | A29~B07~C15~DR13~DQ03~DP02 | 0.00387 | A02~B35~DR11 | 0.00501 | DR15~DQ05~DP02 | 0.00548 |
| A02~B27~C02 | 0.00682 | A01~B40~C15~DR14 | 0.00396 | A03~B44~C16~DR04~DQ03 | 0.00405 | A68~B51~C15~DR11~DQ03~DP04 | 0.00387 | A01~B52~DR15 | 0.00486 | DR08~DQ04~DP03 | 0.00538 |
| A02~B51~C14 | 0.00667 | A02~B49~C07~DR11 | 0.00396 | A24~B40~C03~DR04~DQ03 | 0.00405 | A02~B51~C16~DR04~DQ03~DP04 | 0.00387 | A03~B18~DR11 | 0.00483 | DR15~DQ06~DP03 | 0.0053 |
| A24~B50~C06 | 0.00613 | A01~B35~C06~DR07 | 0.00396 | A30~B13~C06~DR13~DQ06 | 0.00405 | A11~B35~C04~DR08~DQ04~DP04 | 0.00387 | A24~B55~DR13 | 0.00483 | DR15~DQ06~DP01 | 0.00442 |
| A03~B18~C12 | 0.00604 | A02~B44~C05~DR11 | 0.00396 | A01~B27~C07~DR04~DQ03 | 0.00405 | A11~B35~C04~DR14~DQ05~DP04 | 0.00387 | A24~B40~DR04 | 0.00482 | DR01~DQ05~DP03 | 0.00442 |
| A01~B49~C07 | 0.00578 | A68~B51~C15~DR11 | 0.00396 | A02~B49~C07~DR07~DQ02 | 0.00405 | A03~B35~C06~DR01~DQ05~DP04 | 0.00387 | A02~B35~DR14 | 0.00478 | DR03~DQ02~DP01 | 0.00439 |
| A01~B18~C07 | 0.00576 | A25~B18~C12~DR15 | 0.00396 | A02~B50~C06~DR03~DQ02 | 0.00398 | A24~B52~C12~DR15~DQ06~DP04 | 0.00387 | A24~B08~DR03 | 0.00471 | DR07~DQ03~DP02 | 0.00416 |
| A02~B52~C12 | 0.00575 | A30~B35~C06~DR07 | 0.00396 | A24~B18~C12~DR11~DQ03 | 0.0036 | A02~B18~C02~DR04~DQ03~DP04 | 0.00387 | A01~B13~DR07 | 0.00451 | DR04~DQ03~DP14 | 0.00406 |
| A01~B52~C12 | 0.00558 | A24~B38~C12~DR13 | 0.00396 | A02~B41~C17~DR11~DQ03 | 0.00336 | A03~B07~C07~DR15~DQ06~DP03 | 0.00343 | A01~B15~DR04 | 0.00441 | DR01~DQ05~DP09 | 0.00405 |
| A26~B55~C03 | 0.00555 | A24~B35~C04~DR01 | 0.00323 | A11~B35~C04~DR11~DQ03 | 0.00324 | A24~B35~C04~DR04~DQ03~DP04 | 0.00293 | A03~B35~DR01 | 0.00441 | DR11~DQ03~DP104 | 0.00405 |
| A03~B13~C06 | 0.0055 | A02~B49~C07~DR01 | 0.00321 | A24~B49~C07~DR15~DQ06 | 0.00319 | A11~B35~C04~DR11~DQ03~DP04 | 0.00284 | A03~B08~DR03 | 0.00434 | DR13~DQ03~DP03 | 0.0037 |
| A01~B51~C14 | 0.00539 | A02~B07~C07~DR04 | 0.00318 | A02~B08~C07~DR03~DQ02 | 0.00311 | A23~B49~C07~DR04~DQ03~DP04 | 0.00264 | A02~B44~DR01 | 0.00429 | DR16~DQ05~DP03 | 0.00358 |
| A01~B35~C06 | 0.00537 | A03~B35~C06~DR11 | 0.00317 | A24~B18~C07~DR13~DQ06 | 0.00304 | A03~B44~C01~DR04~DQ03~DP04 | 0.00258 | A11~B35~DR13 | 0.00418 | DR08~DQ04~DP02 | 0.00323 |
| A24~B51~C16 | 0.00531 | A23~B49~C07~DR15 | 0.00317 | A03~B44~C04~DR04~DQ03 | 0.00304 | A01~B18~C07~DR03~DQ02~DP09 | 0.00258 | A26~B51~DR11 | 0.00406 | DR15~DQ05~DP09 | 0.00304 |
| A68~B51~C15 | 0.005 | A24~B40~C15~DR04 | 0.00317 | A01~B40~C15~DR14~DQ05 | 0.00304 | A01~B35~C04~DR16~DQ05~DP04 | 0.00258 | A01~B40~DR14 | 0.00405 | DR09~DQ03~DP04 | 0.00304 |
| A11~B51~C15 | 0.00493 | A02~B27~C02~DR16 | 0.00317 | A01~B57~C06~DR03~DQ02 | 0.00304 | A03~B35~C06~DR07~DQ02~DP04 | 0.00258 | A02~B51~DR01 | 0.00398 | DR15~DQ05~DP13 | 0.00301 |
| A03~B55~C01 | 0.00477 | A01~B18~C07~DR04 | 0.00317 | A02~B41~C07~DR03~DQ02 | 0.00304 | A02~B41~C07~DR03~DQ02~DP04 | 0.00258 | A02~B35~DR13 | 0.00382 | DR01~DQ05~DP01 | 0.003 |
| A02~B41~C17 | 0.00475 | A23~B44~C04~DR07 | 0.00317 | A02~B49~C07~DR11~DQ03 | 0.00304 | A68~B51~C14~DR14~DQ05~DP04 | 0.00258 | A24~B51~DR13 | 0.00381 | DR04~DQ04~DP02 | 0.00294 |
| A25~B18~C12 | 0.00465 | A01~B51~C07~DR11 | 0.00317 | A02~B44~C02~DR16~DQ05 | 0.00304 | A02~B51~C15~DR14~DQ05~DP02 | 0.00258 | A30~B35~DR04 | 0.00371 | DR07~DQ03~DP04 | 0.00281 |
| A02~B39~C12 | 0.00464 | A03~B44~C16~DR04 | 0.00317 | A02~B44~C05~DR12~DQ03 | 0.00304 | A01~B50~C06~DR03~DQ02~DP04 | 0.00258 | A02~B44~DR07 | 0.00369 | DR14~DQ03~DP02 | 0.00266 |
| A23~B44~C04 | 0.00433 | A01~B57~C06~DR07 | 0.00317 | A29~B07~C15~DR04~DQ03 | 0.00304 | A01~B35~C04~DR13~DQ03~DP04 | 0.00258 | A11~B35~DR11 | 0.00358 | DR14~DQ05~DP05 | 0.00262 |
| A32~B18~C12 | 0.00425 | A30~B13~C06~DR13 | 0.00317 | A02~B07~C15~DR11~DQ03 | 0.00304 | A02~B35~C12~DR14~DQ05~DP02 | 0.00258 | A23~B49~DR15 | 0.00357 | DR03~DQ02~DP09 | 0.00256 |
| A24~B35~C07 | 0.00396 | A24~B40~C03~DR15 | 0.00316 | A26~B35~C04~DR11~DQ03 | 0.00304 | A24~B18~C07~DR15~DQ06~DP04 | 0.00258 | A24~B35~DR07 | 0.00356 | DR04~DQ04~DP03 | 0.00256 |
| A01~B40~C15 | 0.00396 | A11~B35~C04~DR04 | 0.00312 | A11~B35~C02~DR04~DQ03 | 0.00304 | A02~B35~C06~DR04~DQ03~DP02 | 0.00258 | A24~B07~DR15 | 0.00356 | DR13~DQ03~DP02 | 0.00244 |
| A26~B51~C14 | 0.00396 | A03~B13~C06~DR07 | 0.00304 | A02~B51~C02~DR01~DQ05 | 0.00304 | A02~B35~C06~DR07~DQ02~DP03 | 0.00258 | A02~B38~DR13 | 0.00356 | DR04~DQ03~DP05 | 0.00232 |
| A33~B58~C03 | 0.00396 | A01~B35~C04~DR14 | 0.00302 | A68~B35~C04~DR13~DQ06 | 0.00304 | A01~B07~C07~DR15~DQ06~DP04 | 0.00258 | A30~B13~DR07 | 0.00353 | DR16~DQ05~DP10 | 0.00232 |
| A24~B35~C12 | 0.00395 | A24~B51~C15~DR14 | 0.00298 | A03~B44~C14~DR04~DQ03 | 0.00304 | A26~B27~C01~DR01~DQ03~DP03 | 0.00258 | A02~B51~DR16 | 0.00351 | DR14~DQ05~DP03 | 0.00228 |
| A29~B58~C07 | 0.00393 | A11~B07~C07~DR15 | 0.00295 | A11~B35~C04~DR08~DQ04 | 0.00304 | A11~B35~C04~DR01~DQ05~DP09 | 0.00258 | A01~B57~DR07 | 0.0034 | DR01~DQ05~DP05 | 0.00227 |
| A03~B44~C02 | 0.00384 | A02~B07~C07~DR15 | 0.00293 | A30~B35~C04~DR04~DQ03 | 0.00304 | A11~B44~C04~DR07~DQ02~DP04 | 0.00258 | A26~B44~DR11 | 0.00339 | DR14~DQ05~DP17 | 0.00211 |
| A02~B35~C12 | 0.00381 | A02~B50~C06~DR03 | 0.00285 | A01~B37~C06~DR04~DQ05 | 0.00304 | A02~B35~C04~DR04~DQ05~DP04 | 0.00258 | A32~B51~DR11 | 0.00334 | DR01~DQ05~DP51 | 0.00208 |
| A01~B41~C17 | 0.0038 | A24~B55~C01~DR11 | 0.00283 | A01~B35~C04~DR04~DQ04 | 0.00304 | A11~B35~C02~DR04~DQ03~DP02 | 0.00258 | A11~B35~DR04 | 0.00331 | DR15~DQ06~DP15 | 0.00207 |
| A32~B51~C14 | 0.00375 | A02~B08~C07~DR03 | 0.00282 | A02~B40~C03~DR14~DQ05 | 0.00304 | A23~B15~C07~DR11~DQ03~DP02 | 0.00258 | A26~B38~DR14 | 0.00331 | DR11~DQ03~DP05 | 0.00206 |
| A11~B07~C07 | 0.00371 | A03~B52~C12~DR15 | 0.00275 | A32~B52~C12~DR15~DQ06 | 0.00304 | A24~B27~C01~DR03~DQ02~DP04 | 0.00258 | A02~B41~DR03 | 0.00318 | DR03~DQ03~DP04 | 0.00203 |
| A24~B40~C15 | 0.00367 | A24~B49~C07~DR11 | 0.00272 | A02~B40~C03~DR15~DQ06 | 0.00304 | A24~B48~C08~DR04~DQ03~DP02 | 0.00258 | A03~B35~DR07 | 0.00314 | DR07~DQ02~DP15 | 0.00202 |
| A24~B51~C14 | 0.00365 | A24~B50~C06~DR03 | 0.00263 | A33~B51~C14~DR11~DQ03 | 0.00304 | A33~B58~C03~DR11~DQ03~DP02 | 0.00258 | A11~B52~DR11 | 0.00313 | DR01~DQ05~DP17 | 0.00202 |
| A03~B44~C16 | 0.0036 | A23~B49~C07~DR13 | 0.00259 | A01~B15~C07~DR13~DQ06 | 0.00304 | A66~B41~C17~DR04~DQ04~DP04 | 0.00258 | A68~B35~DR13 | 0.00312 | DR07~DQ03~DP13 | 0.00202 |
| A03~B52~C12 | 0.00341 | A02~B52~C12~DR15 | 0.00258 | A01~B57~C06~DR07~DQ03 | 0.00304 | A30~B35~C06~DR07~DQ02~DP04 | 0.00258 | A11~B35~DR14 | 0.00299 | DR15~DQ06~DP23 | 0.00202 |
| A32~B52~C12 | 0.0034 | A02~B51~C15~DR11 | 0.00252 | A02~B38~C12~DR13~DQ06 | 0.00304 | A68~B35~C04~DR13~DQ06~DP04 | 0.00258 | A23~B49~DR13 | 0.00299 | DR07~DQ03~DP09 | 0.00202 |
| A11~B38~C12 | 0.00338 | A02~B41~C17~DR11 | 0.00251 | A02~B27~C02~DR16~DQ05 | 0.00304 | A02~B38~C12~DR11~DQ05~DP04 | 0.00258 | A02~B44~DR15 | 0.00293 | DR16~DQ05~DP23 | 0.00202 |
| A03~B35~C12 | 0.00334 | A02~B51~C15~DR04 | 0.00248 | A23~B44~C04~DR07~DQ02 | 0.00304 | A01~B40~C03~DR14~DQ04~DP03 | 0.00258 | A02~B08~DR03 | 0.00291 | DR10~DQ05~DP15 | 0.00202 |
| A26~B15~C07 | 0.0033 | A24~B51~C15~DR15 | 0.00246 | A02~B15~C03~DR15~DQ06 | 0.00304 | A30~B35~C04~DR04~DQ03~DP04 | 0.00258 | A02~B27~DR16 | 0.00291 | DR04~DQ03~DP47 | 0.00202 |
| A24~B40~C03 | 0.00327 | A24~B49~C07~DR15 | 0.00244 | A32~B35~C04~DR16~DQ05 | 0.00304 | A03~B52~C12~DR14~DQ04~DP04 | 0.00258 | A02~B40~DR04 | 0.00289 | DR09~DQ03~DP10 | 0.00202 |
| A02~B51~C02 | 0.00324 | A24~B35~C07~DR03 | 0.00238 | A11~B07~C07~DR15~DQ06 | 0.00304 | A32~B38~C12~DR14~DQ05~DP04 | 0.00258 | A29~B07~DR04 | 0.00279 | DR07~DQ02~DP105 | 0.00202 |
| A03~B49~C07 | 0.00324 | A26~B08~C07~DR10 | 0.00238 | A11~B35~C04~DR01~DQ05 | 0.00301 | A26~B15~C07~DR13~DQ03~DP02 | 0.00258 | A03~B07~DR04 | 0.00278 | DR04~DQ03~DP104 | 0.00202 |
| A02~B08~C07 | 0.0032 | A32~B18~C12~DR14 | 0.00238 | A24~B51~C15~DR15~DQ06 | 0.00263 | A03~B07~C07~DR04~DQ03~DP04 | 0.00258 | A24~B18~DR13 | 0.00278 | DR15~DQ05~DP03 | 0.00202 |
| A24~B08~C07 | 0.00319 | A01~B35~C04~DR16 | 0.00238 | A02~B51~C14~DR04~DQ03 | 0.00248 | A11~B55~C01~DR11~DQ03~DP04 | 0.00258 | A03~B55~DR13 | 0.00276 | DR13~DQ06~DP15 | 0.00198 |
| A66~B41~C17 | 0.00317 | A01~B37~C06~DR16 | 0.00238 | A68~B35~C04~DR07~DQ02 | 0.00241 | A11~B38~C12~DR04~DQ03~DP02 | 0.00258 | A01~B07~DR15 | 0.00267 | DR07~DQ02~DP06 | 0.00189 |
| A32~B40~C02 | 0.00317 | A02~B44~C05~DR01 | 0.00238 | A02~B51~C15~DR14~DQ05 | 0.0024 | A32~B35~C04~DR11~DQ03~DP02 | 0.00258 | A24~B35~DR03 | 0.00266 | DR15~DQ06~DP14 | 0.00171 |
| A02~B51~C07 | 0.00317 | A23~B49~C07~DR01 | 0.00238 | A24~B07~C07~DR11~DQ03 | 0.00204 | A11~B07~C07~DR03~DQ02~DP02 | 0.00258 | A24~B35~DR01 | 0.0026 | DR11~DQ03~DP06 | 0.00149 |
| A24~B48~C08 | 0.00317 | A01~B57~C06~DR03 | 0.00238 | A02~B35~C12~DR14~DQ05 | 0.00203 | A33~B35~C04~DR11~DQ03~DP03 | 0.00258 | A24~B18~DR14 | 0.00252 | DR15~DQ06~DP05 | 0.00143 |
| A26~B27~C01 | 0.00316 | A26~B38~C12~DR11 | 0.00238 | A24~B50~C06~DR11~DQ03 | 0.00202 | A02~B49~C07~DR10~DQ05~DP04 | 0.00258 | A24~B07~DR04 | 0.00245 | DR13~DQ06~DP05 | 0.00139 |
| A11~B49~C07 | 0.00315 | A02~B41~C07~DR03 | 0.00238 | A02~B39~C12~DR16~DQ05 | 0.00202 | A02~B40~C03~DR15~DQ06~DP04 | 0.00258 | A26~B51~DR13 | 0.00245 | DR10~DQ03~DP04 | 0.00136 |
| A02~B44~C02 | 0.00311 | A02~B50~C06~DR11 | 0.00238 | A24~B55~C01~DR13~DQ06 | 0.00202 | A26~B35~C12~DR04~DQ03~DP04 | 0.00258 | A03~B38~DR13 | 0.00243 | DR10~DQ05~DP10 | 0.00133 |
| A01~B15~C07 | 0.00302 | A02~B44~C02~DR16 | 0.00238 | A24~B13~C06~DR03~DQ02 | 0.00202 | A01~B35~C04~DR13~DQ06~DP02 | 0.00258 | A01~B35~DR16 | 0.00242 | DR15~DQ05~DP04 | 0.00132 |
| A03~B51~C15 | 0.00301 | A26~B27~C01~DR01 | 0.00238 | A03~B51~C01~DR04~DQ03 | 0.00202 | A02~B40~C03~DR13~DQ06~DP02 | 0.00258 | A02~B14~DR01 | 0.00236 | DR08~DQ03~DP03 | 0.0013 |
| A30~B51~C16 | 0.00286 | A02~B35~C04~DR13 | 0.00238 | A23~B44~C04~DR01~DQ05 | 0.00202 | A02~B44~C05~DR04~DQ03~DP03 | 0.00258 | A02~B41~DR15 | 0.00232 | DR08~DQ06~DP03 | 0.00124 |
| A26~B55~C01 | 0.00281 | A68~B35~C04~DR04 | 0.00238 | A03~B35~C06~DR07~DQ02 | 0.00202 | A02~B15~C03~DR07~DQ02~DP17 | 0.00258 | A11~B35~DR15 | 0.00232 | DR04~DQ03~DP01 | 0.0012 |
| A01~B44~C05 | 0.00268 | A02~B51~C16~DR04 | 0.00238 | A02~B44~C05~DR01~DQ05 | 0.00202 | A02~B27~C02~DR16~DQ05~DP02 | 0.00258 | A02~B27~DR04 | 0.00228 | DR07~DQ03~DP03 | 0.00118 |
| A02~B14~C08 | 0.00265 | A02~B44~C16~DR13 | 0.00238 | A68~B51~C14~DR14~DQ05 | 0.00202 | A68~B35~C04~DR07~DQ02~DP02 | 0.00258 | A32~B35~DR15 | 0.00226 | DR04~DQ03~DP13 | 0.00116 |
| A30~B49~C07 | 0.00257 | A02~B51~C02~DR01 | 0.00238 | A32~B35~C04~DR11~DQ03 | 0.00202 | A24~B08~C07~DR03~DQ03~DP04 | 0.00258 | A24~B15~DR13 | 0.00224 | DR11~DQ06~DP02 | 0.00115 |
| A24~B13~C06 | 0.00255 | A03~B49~C07~DR04 | 0.00238 | A33~B13~C08~DR01~DQ05 | 0.00202 | A24~B35~C04~DR12~DQ03~DP04 | 0.00258 | A01~B35~DR07 | 0.00223 | DR03~DQ02~DP66 | 0.00115 |
| A24~B56~C01 | 0.00254 | A26~B35~C04~DR13 | 0.00238 | A01~B44~C07~DR15~DQ06 | 0.00202 | A02~B15~C03~DR15~DQ06~DP02 | 0.00258 | A26~B15~DR13 | 0.00223 | DR04~DQ05~DP04 | 0.00108 |
| A32~B35~C12 | 0.00251 | A03~B44~C14~DR04 | 0.00238 | A01~B35~C07~DR04~DQ03 | 0.00202 | A24~B51~C15~DR14~DQ05~DP04 | 0.00258 | A26~B38~DR13 | 0.0022 | DR11~DQ03~DP17 | 0.00106 |
| A03~B44~C05 | 0.00244 | A11~B35~C04~DR08 | 0.00238 | A30~B49~C07~DR13~DQ06 | 0.00202 | A02~B51~C07~DR13~DQ06~DP02 | 0.00258 | A33~B58~DR03 | 0.0022 | DR11~DQ02~DP02 | 0.00104 |
| A02~B44~C07 | 0.00241 | A02~B51~C15~DR09 | 0.00238 | A02~B51~C07~DR13~DQ06 | 0.00202 | A24~B51~C16~DR13~DQ06~DP02 | 0.00258 | A02~B15~DR08 | 0.00219 | DR04~DQ02~DP04 | 0.00103 |
[truncated: 322,098 more chars]
